# Photocatalytic Minisci Approach to Chiral 2‑Hetaryl-1,2-aminoalcohols from β‑Hydroxy-α-amino Acid-Derived Redox Active Esters

**DOI:** 10.1021/acs.joc.5c02485

**Published:** 2025-11-05

**Authors:** Paula Oroz, Carmen Bretón, Miguel Torres, Iván Olagaray, Eduardo Sainz, Cristina M. Segovia, Alberto Avenoza, Jesús H. Busto, Francisco Corzana, Gonzalo Jiménez-Osés, Jesús M. Peregrina

**Affiliations:** † Departamento de Química, Instituto de Investigación en Química de la Universidad de La Rioja (IQUR), 16764 Universidad de La Rioja, C/Madre de Dios, 53, 26006 Logroño, La Rioja, Spain; ‡ Center for Cooperative Research in Biosciences (CIC bioGUNE), 73038 Basque Research and Technology Alliance (BRTA), 48160 Derio, Spain; § Ikerbasque, Basque Foundation for Science, 48013 Bilbao, Spain

## Abstract

Chiral 1,2-aminoalcohols are privileged motifs in bioactive molecules, yet their stereocontrolled synthesis remains a challenge. Here, we report a general, metal-free photoredox Minisci strategy that converts bicyclic *N*,*O*-acetals derived from β-hydroxy-α-amino acids into enantiopure 2-hetaryl-1,2-aminoalcohols with complete stereoretention. This diastereoselective radical α-hetarylation proceeds under visible light and mild conditions, tolerating diverse substitution patterns on both the heteroarene and the bicyclic scaffold. Combined experimental and quantum mechanical studies reveal that dispersion (CH/π) interactions between the incoming heteroarene and the bridgehead methyl group govern the unexpected facial selectivity in the C–C bond-forming step. The products serve as versatile precursors to medicinally relevant scaffolds, and the scalability of the process highlights its synthetic utility. This work establishes a broadly applicable platform for the stereocontrolled radical functionalization of amino acid derivatives.

## Graphical abstract

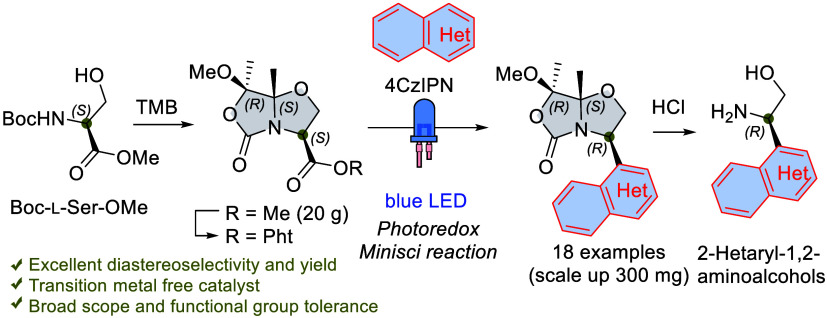

## Introduction

1,2-Aminoalcohols, which are ubiquitous in both natural and synthetic biologically active compounds, serve as versatile scaffolds in organic synthesis. They are widely used as building blocks, chiral auxiliaries, and catalysts—particularly the 2-substituted 2-aminoethanols.^1^ These motifs are crucial in medicinal chemistry for the development of drug candidates.^2^ Various methods have been developed to construct this scaffold through reactions involving both ionic^3^ and radical intermediates.^4^ Consequently, the development of stereoselective methods for the preparation of 1,2-aminoalcohols, especially chiral 2-(het)­aryl-2-aminoethanols, remains a highly active area of research in organic synthesis. Recently, a radical-based approach has been reported for the synthesis of 1-substituted 2-aminoethanols, utilizing chiral oxazolidine-carboxylic acid via Ni-electrocatalytic decarboxylative arylation.^5^

Given the established value of the Minisci reaction for C–H functionalization of heteroarenes, particularly in its photocatalytic variant,^6^ we envisioned a diastereoselective photoredox catalytic approach to synthesize enantioenriched 2-hetaryl-2-aminoethanols. This strategy involves a Minisci-type reaction between heteroarenes and prochiral cyclic *N*-Boc-protected α-amino radicals as nucleophiles. While enantioselective Minisci reactions have recently been explored using chiral acids,^7^ diastereoselective approaches remain relatively underdeveloped.

## Results and Discussion

### Identification of Prochiral Cyclic *N*-Protected α-Amino Radical Precursors

Inspired by the efficient generation of alkyl radicals in Minisci reactions through the combination of redox-active esters (RAEs) and photoredox catalysis,^8^ we have focused on bicyclic compound **4** as the starting RAE material (Scheme 1).

**Scheme 1:**
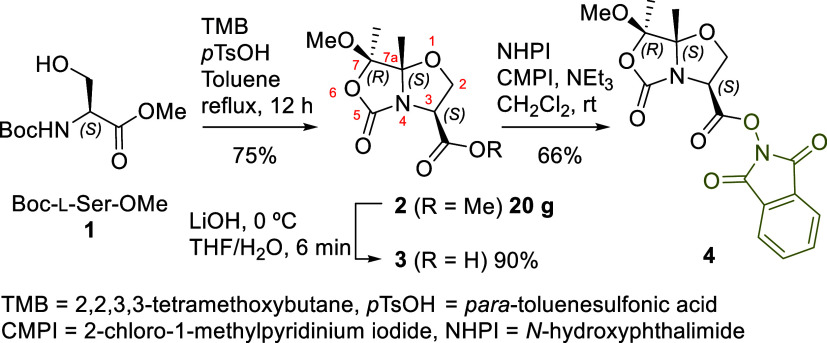
Synthesis of Redox Active Ester **4**

This compound **4** is derived from the chiral bicyclic serine equivalent **2**,^9,10^ which has demonstrated high efficiency in diastereoselective alkylations^9,10^ and serves as a precursor to chiral dehydroalanines, enabling access to a variety of unnatural amino acids.^11^ The bicyclic serine derivative **2** was synthesized on a 20 g scale with 99% enantiomeric excess (ee) from *N*-Boc-l-Ser-OMe **1**,^10^ following a recently improved protocol.^11^ This compound was then hydrolyzed using LiOH to afford acid **3**, which was coupled to *N*-hydroxyphthalimide (NHPI) employing 2-chloro-1-methylpyridinium iodide (CMPI) or propanephosphonic acid anhydride (T3P) as the coupling agent, yielding RAE **4** in a good yield (see Scheme 1 and Supporting Information).

Crucial factors influencing the success of the Minisci reaction include employing a photoactive catalyst with a suitable redox potential, which allows for the selective generation of α-aminoalkyl radicals while avoiding undesirable overoxidation. Additionally, the use of an acidic cocatalyst is fundamental to increase the electron-deficient character of *N*-heteroarenes.^6^ Consequently, RAE **4** was evaluated in this reaction with isoquinoline under previously reported conditions.^12–14^ To begin, we applied the protocol established by Shang and Fu,^12^ utilizing RAE **4** and isoquinoline in a diastereoselective Minisci reaction with an iridium-based photoredox catalyst (Cat-Ir) and a catalytic amount of racemic phosphoric acid (*rac-*PA), conducted at room temperature under blue LED irradiation. This approach Scheme 2, conditions **A** gave compound **5** in 53% yield after column chromatography. Drawing on further research by the same authors,^15^ we replaced the Brønsted acid (*rac-*PA) with the Lewis acid In­(OTf)_3_ as a cocatalyst. This modification provided a comparable efficiency, resulting in a 45% yield of compound **5** under otherwise identical conditions (Scheme 2, conditions **B**). Subsequently, we tested the photoinduced Minisci reaction developed by Molander and co-workers,^14a^ which offers a more straightforward strategy. This method utilizes NaHSO_4_ as a mild activating agent and relies on the formation of an electron donor–acceptor (EDA) complex between the heteroarene and the radical acceptor. To evaluate this, we conducted a catalyst-free Minisci reaction between RAE **4** and isoquinoline in DMF, irradiating the mixture with blue LEDs for 16 h. However, this EDA methodology^14^ resulted in a complex product mixture, with Minisci-type products accounting for less than 10% of the overall yield (Scheme 2, conditions **C**).

**Scheme 2:**
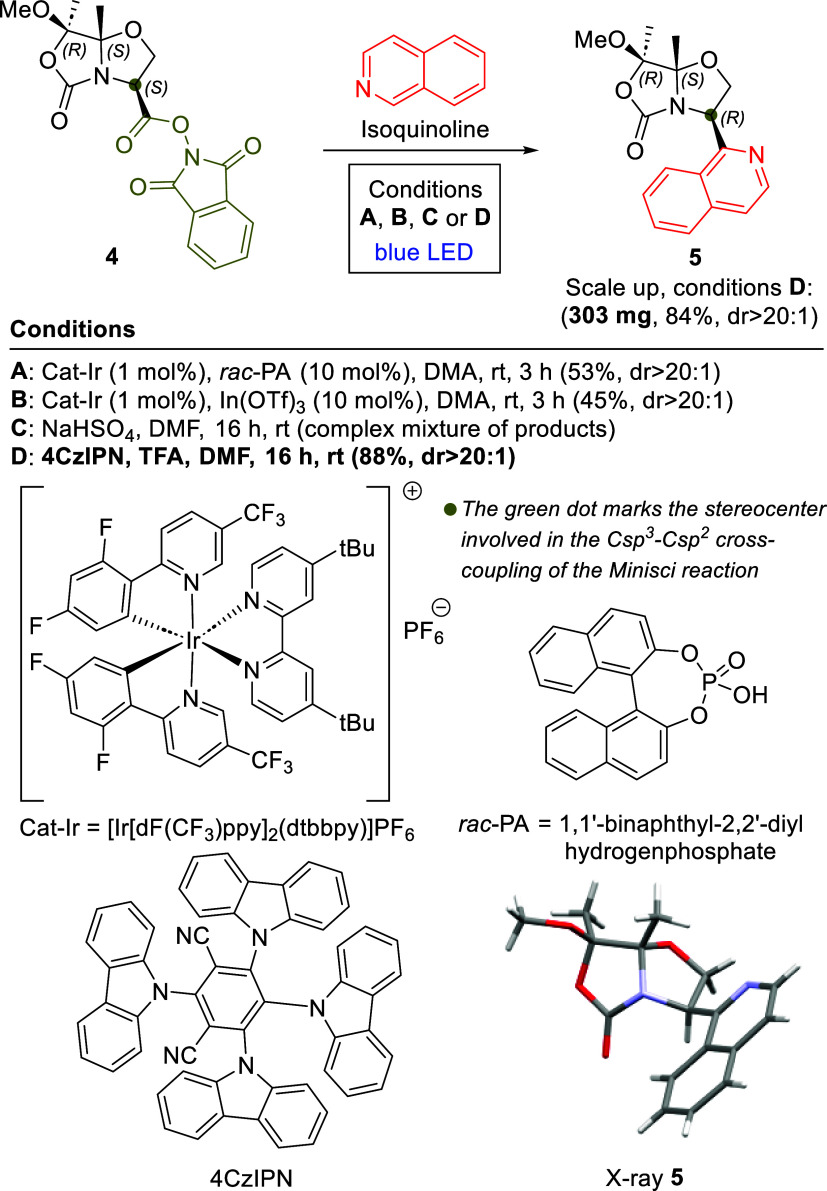
Minisci Reactions of RAE **4** to Obtain Derivative **5**

Finally, the optimal conditions were achieved using a methodology similar to that developed by Sherwood and co-workers,^13^ which involves the use of 1,2,3,5-tetrakis­(carbazol-9-yl)-4,6-dicyanobenzene (4CzIPN) as an organic photocatalyst and stoichiometric amounts of trifluoroacetic acid (TFA) as an acidic additive in DMF using visible light. These mild conditions led to the desired Minisci product **5** in 88% yield after column chromatography (Scheme 2, conditions **D**).

Switching the solvent to dichloromethane led to a marked drop in yield (<10%). Notably, these reactions proceeded under conditions **A**, **B**, and **D** with complete stereoselectivity, yielding exclusively compound **5** as a single diastereoisomer (dr >20:1 determined by NMR). In contrast, no product formation was observed in the absence of blue light or catalyst.

### Scope of Bicyclic RAEs Derived from Other *N*-Protected α-Amino Acids in the Minisci Reaction

Encouraged by these results, we further explored the scope of the Minisci reaction utilizing other chiral bicyclic RAEs derived from β-hydroxy-α-amino acids, including l-threonine (Thr) and (*S*)-α-methylserine (MeSer).^16^ RAE **6** was synthesized following the same protocol established for RAE **4**. RAE **8** was prepared from the chiral serine analog **2** via diastereoselective α-methylation,^9,10^ followed by ester hydrolysis and subsequent coupling to NHPI using propylphosphonic anhydride (T3P) (see Scheme 3 and Supporting Information). Consistent with the results observed for the bicyclic serine derivative, Minisci reactions between the Thr or MeSer analogs and isoquinoline under optimized conditions **D** afforded products **7** and **9**, respectively, in good yields and with complete diastereoselectivity (Scheme 3).

**Scheme 3:**
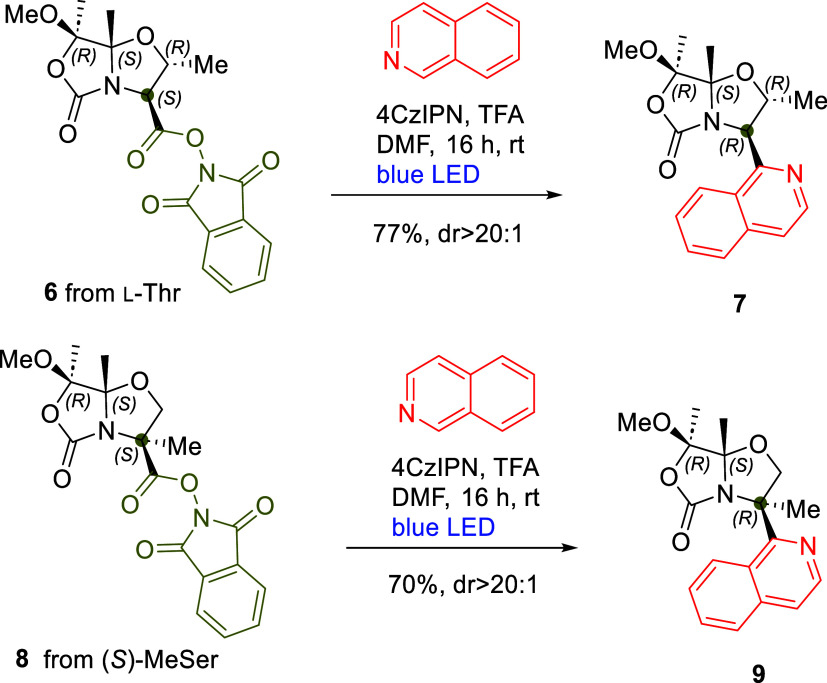
Minisci Reactions of RAEs **6** and **8** with Isoquinoline

The absolute configuration at carbon C3 in products **5**, **7**, and **9** was determined to be (*R*) in all cases by 2D-NOESY NMR experiments (Supporting Information) and was further confirmed by X-ray crystallography on compound **5** (Scheme 2). Consequently, the absolute configuration at this stereocenter was entirely retained throughout the Minisci reaction. The observed change in configuration assignment results solely from differences in the Cahn-Ingold-Prelog priority rules.

### Scope of Heteroarene in the Catalytic Visible Light-Induced Minisci Reactions of RAE 4

We also investigated the scope of the Minisci reaction of RAE **4** under optimized conditions **D** with various heteroarenes other than isoquinoline. Different substituted isoquinolines bearing both electron-donating and electron-withdrawing groups (5-methoxy-, 4-hydroxy-, and 5-, 6-, and 7-bromoisoquinolines), as well as quinoline and substituted quinolines (2- and 4-methyl, and 4-methoxyquinolines), were examined. Similarly, we extended this reaction to other benzo-fused heterocycles such as quinoxaline, benzo­[*d*]­thiazole, and phenanthridine. As a result, we obtained a series of 15 compounds (**5** and **10–22**) in high yields and with diastereoselectivities comparable to those observed with isoquinoline (see Scheme 4).

**Scheme 4:**
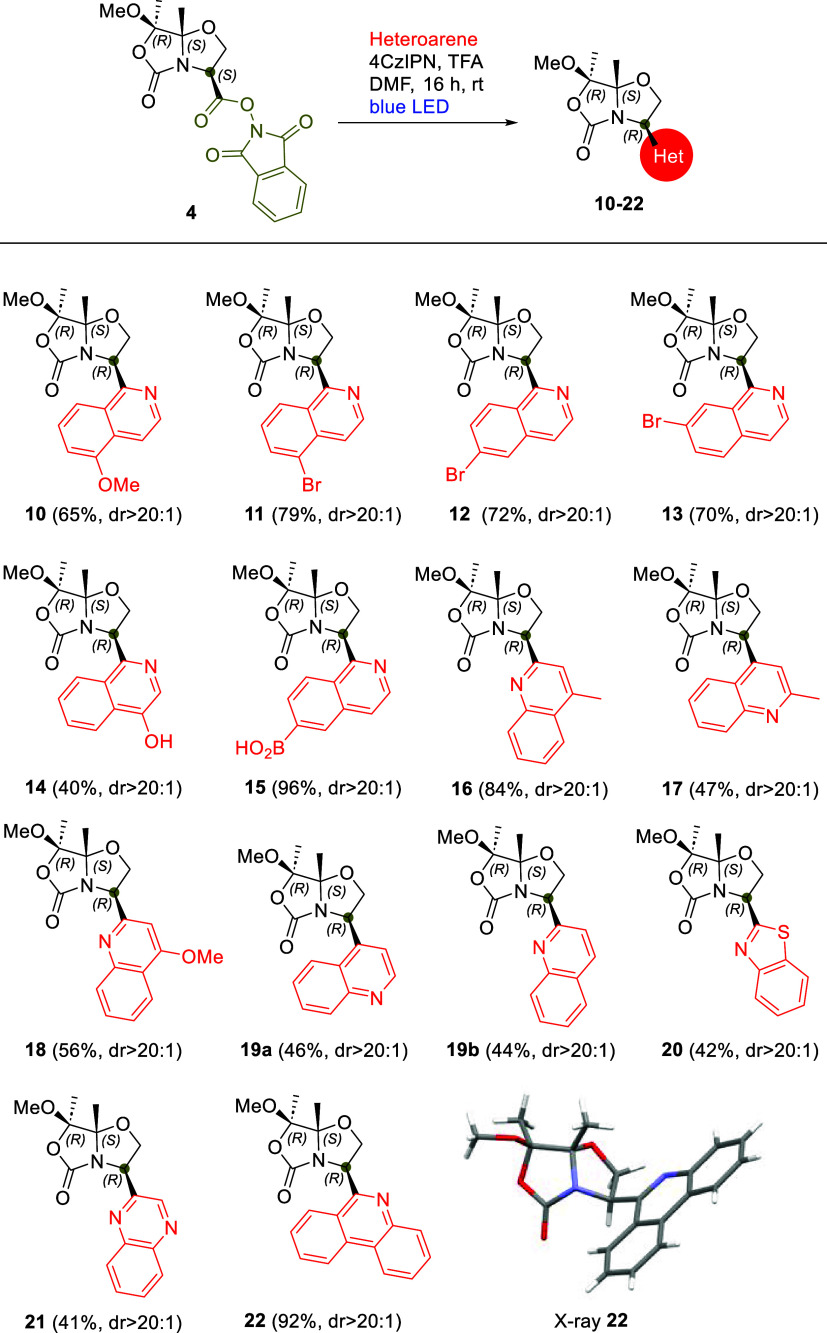
Scope of the Minisci Reactions of RAE **4**

In the case of quinoline, as expected, the reaction with RAE **4** was not regioselective, yielding a mixture of compounds **19a** and **19b** in a ratio of 48:52, respectively, which could be easily separated by chromatography. Interestingly, while the reaction between **4** and 4-hydroxyisoquinoline produced derivative **14** with moderate yield and high diastereoselectivity (Scheme 4), the isomers 3-hydroxyisoquinoline and 4-hydroxyquinoline did not react, likely because they exist predominantly as their keto tautomers, isoquinolin-3­(2*H*)-one and quinolin-4­(1*H*)-one, respectively. We also explored various pyridine derivatives, including pyridine, 3- and 4-acetylpyridine, 4-vinylpyridine, 4-phenylpyridine, pyrazine, pyrimidine, and pyridazine, without success.

### Synthetic Applications of the Catalytic Visible Light-Induced Minisci Reactions

To achieve milder reaction conditions, we carried out the Minisci reaction using RAE **4** and quinolinium hydrogen sulfate under the same conditions but without TFA. This approach produced a mixture of two compounds in a 30:70 ratio, both with high yield and excellent diastereoselectivity (dr >20:1). One product corresponded to derivative **19a**, while, interestingly, the other was a quinoline functionalized at both the 2- and 4-positions with two bicyclic amino alcohols (**19c**). This outcome highlights the increased reactivity of preformed stoichiometric quinoline-acid ion pairs. By employing an excess of RAE **4**, complete bis-Minisci coupling leading to compound **19c** was achieved (Scheme 5).

**Scheme 5:**
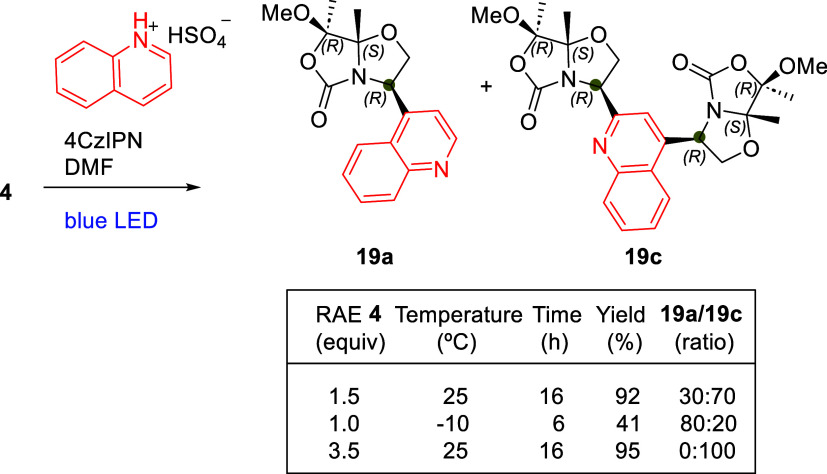
Minisci Reactions of RAE **4** with Quinolinium Hydrogen Sulfate to Obtain Mono- (**19a**) and Bis-Minisci Coupling Products (**19c**) (dr >20:1)

Finally, selected Minisci products **5**, **7**, **9**, and **16** were hydrolyzed with HCl at 60 °C to quantitatively afford the chiral 2-hetaryl-1,2-aminoalcohols **23**, **24**, **25**, and **26**, respectively (Scheme 6). To validate the viability of this synthetic process, we scaled up the process and obtained 217 mg (38% overall yield, 5 steps) of (*R*)-2-amino-2-(isoquinolin-1-yl)­ethan-1-ol hydrochloride **23** starting from 560 mg of Boc-l-Ser-OMe (**1**) (Supporting Information). To the best of our knowledge, the synthesis of commercially available salt-free compound **23** (CAS: 1212829–79–2) has not been previously reported.

**Scheme 6:**
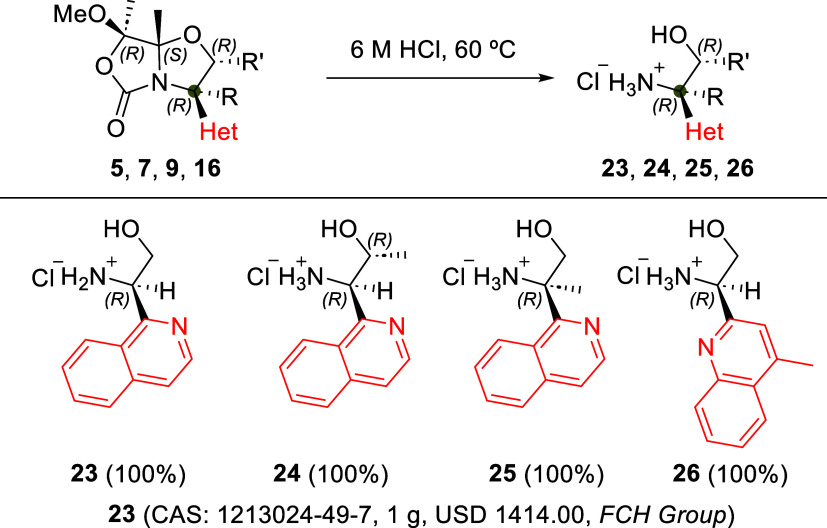
Synthesis of Chiral 2-Hetaryl-1,2-aminoalcohols

Remarkably, while preparing this manuscript, Kawamata and Baran reported an outstanding work^17^ on enantioselective 1,2-aminoalcohol synthesis via chemoselective Ni-electrocatalytic radical cross couplings, using the same RAE **4** derived from our bicyclic serine equivalent **2**, and a variety of coupling partners.

### Investigation of the Reaction Mechanism

To investigate the mechanism of this Minisci reaction, a radical trapping experiment was performed. To probe the formation of an alkyl radical intermediate, 2,2,6,6-tetramethyl-1-piperidinyloxy (TEMPO) was employed as a radical trapping agent in place of isoquinoline under standard conditions **D** (Scheme 7). The resulting TEMPO adduct **27** was successfully identified by HRMS and NMR analyses (see Supporting Information).

**Scheme 7:**
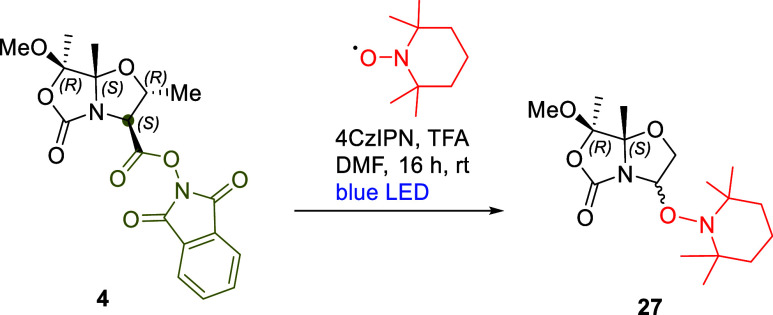
Reaction of RAE **4** with TEMPO

Based on the proposed mechanism for this Minisci-type reaction,^12^ (Scheme 8 and Supporting Information) and related studies on radicals generated by visible-light photocatalysis,^18^ the origin of the observed stereoselectivity was investigated through quantum mechanical calculations (Figure 1 and Supporting Information). The carboxyl radical **4B_** ***S***, proposed to form after irradiation of RAE **4**, undergoes spontaneous CO_2_ extrusion to yield the carbon-centered bicyclic radical **4C_** ***S*** in a highly exergonic initiation step (Δ*G* ≈ 29 kcal mol^–1^).

**Figure 1:**
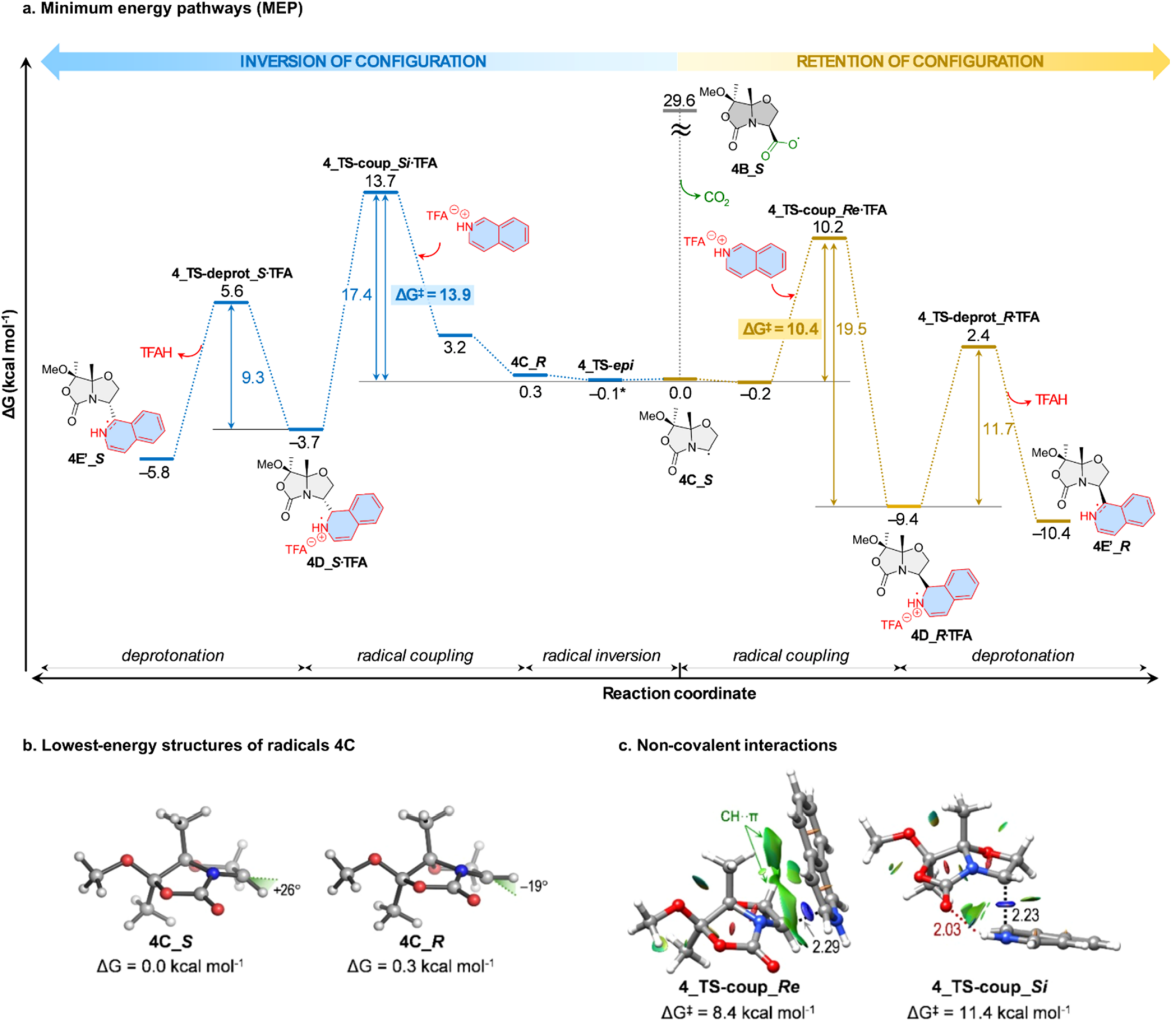
Computational study of the Minisci reaction. (a) Minimum energy pathways (MEP) calculated with PCM­(DMF)/M06–2X/6–311G­(d,p) for reactions originating from radical **4B_** ***S***. This species decarboxylates to form pyramidalized C-centered radicals in (*S*) and (*R*) configurations in a rapid equilibrium; each of those radicals then undergo diastereoselective radical coupling with the isoquinolinium-trifluoroacetate ion pair (**4_TS-coup**). The resulting intermediate adducts, **4D·TFA**, subsequently undergo internal deprotonation by TFA to yield quasi-aromatic radicals **4E**′. The bicyclic C-centered radical pyramidalized in (*S*) configuration (**4C_S**) has been arbitrarily assigned a free energy value (ΔG) of zero. (b, c) Lowest-energy structures calculated with PCM­(DMF)/M06–2X/6–311G­(d,p) for the intermediate radicals (**4C**) and C–C coupling transition states with isoquinolinium (**4_TS-coup**). Noncovalent interactions (NCI) between the radical and isoquinolinium coupling partners are shown as colored isosurfaces; (blue; strong attractive interactions, green; weak van der Waals interactions and red; strong repulsive interactions). Forming C–C bonds and hydrogen bonds are shown as black and red dashed lines, respectively. Distances are given in angstrom.

**Scheme 8:**
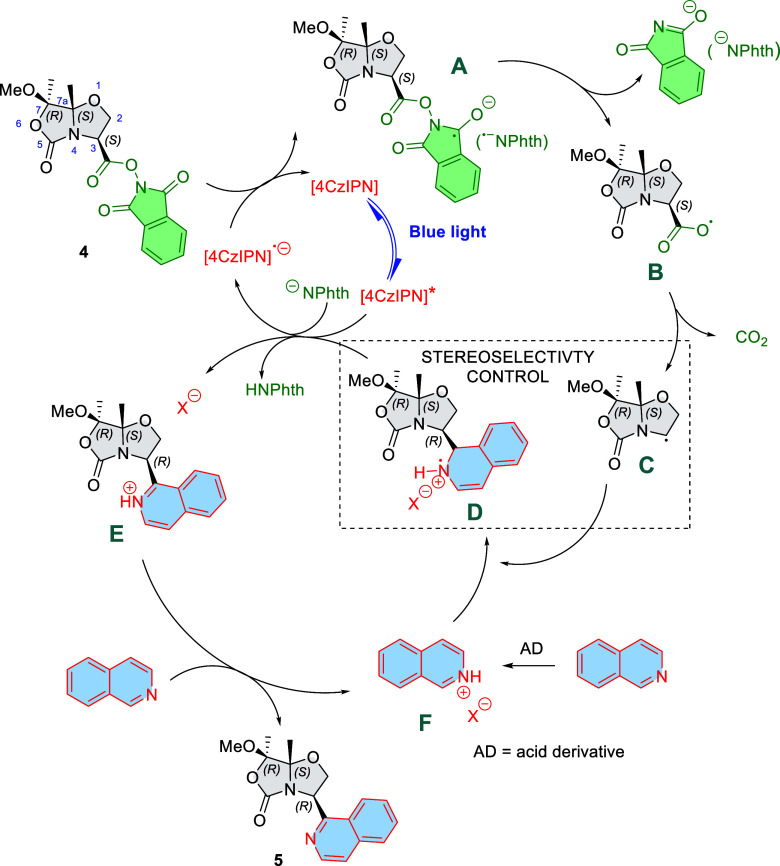
Proposed Reaction Mechanism for the 4CzIPN-Photocatalyzed Minisci Reaction of RAE **4** with Isoquinoline

Similar to analogous bicyclic enolates^9^ such C-radical is highly pyramidalized (+26°) and formally retains the initial (*S*) configuration; however, epimerization of this “chiral” radical toward **4C_** ***R***, which is isoenergetic (Δ*G* = 0.3 kcal mol^–1^; Figure 1b), was calculated to be exceedingly fast (i.e., barrierless), thus leading to a Curtin–Hammett scenario. This is a major difference regarding the source of stereoselectivity proposed for related anionic α-alkylation reactions.^9^ For the radical coupling and subsequent deprotonation of the Wheland-type intermediate with trifluoroacetate as a base, two electrophiles were evaluated: (i) cationic isoquinolinium, and (ii) neutral isoquinolinium-trifluoroacetate ion pair.

The minimum energy pathway calculated with the ion pair (Figure 1a) clearly shows that, unlike in previously reported chiral phosphoric acid catalyzed enantioselective Minisci reactions,^19^ the rate and stereoselectivity-determining step is the exergonic, irreversible radical coupling between **4C** and the activated heteroarene. Therefore, to simplify the discussion, we will hereafter refer only to this key step in the model reaction with cationic isoquinolinium. This radical C–C bond formation, unlike anionic α-alkylation,^9^ occurs predominantly on the concave face of the bicycle (*Re* in this case) (**4_TS-coup_** ***Re***: Δ*G* ^‡^ = 8.4 kcal mol^–1^ vs **4_TS-coup_** ***Si***: Δ*G* ^‡^ = 11.4 kcal mol^–1^) (Figure 1c).

While this result aligns well with the experimental observations, it was somewhat surprising given the expectation that steric hindrance from the bridgehead methyl group would block the concave face. Moreover, the computed hydrogen bond between the carbamate carbonyl of the substrate and the N–H group of the isoquinolinium would be expected to further favor attack on the opposite, convex (*Si*) face. Quite the opposite, the transition state (TS) leading to the experimental product (**4_TS-coup_** ***Re***) is much more stable; hence, we propose that dispersion interactions (i.e., CH/π) between the bridgehead methyl group of the substrate and positively charged, highly polarizable isoquinolinium dictate the correctly calculated preference for radical coupling on the concave face of the bicycle (Figure 1c and Supporting Information). Very similar energy profiles and structures were calculated for the reactions involving the C-radicals derived from RAE **6** and **8**. The presence of an additional methyl group at β or α positions either increases (ΔΔ*G*_Re–Si_^‡^ ≈ 6.7 kcal mol^–1^ for Thr-derived radical **6C**) or decreases (ΔΔ*G*_Re–Si_^‡^ ≈ 1.2 kcal mol^–1^ for MeSer-derived radical **8C**) the energy difference between the lowest-energy TS in each case, but the global trend and diastereoselectivity is preserved when all transition states are considered with Boltzmann weighting (see Supporting Information for further details).

To experimentally validate the calculated reaction mechanism, we conducted a kinetic isotope effect (KIE) study of the photoredox Minisci reaction between RAE **4** and isoquinoline, as well as fully deuterated isoquinoline, yielding products **5** and **5-D**, respectively (Figure 2). Initially, we carried out the reaction of RAE **4** with isoquinoline-d7 to characterize the new Minisci product **5-D**. Then, an intermolecular competition experiment was conducted with equimolecular amounts of isoquinoline and isoquinoline-d7 in a 5 mm diameter NMR tube using deuterated DMF as solvent, and the reaction progress was monitored at various time intervals. As a result, no appreciable kinetic isotope effect was observed within experimental error (KIE = 1.05), indicating that cleavage of the C–H/C–D bond associated with isoquinoline deprotonation does not contribute to the rate-determining step of the reaction. In fact, a theoretical primary KIE between 4.5 and 5.2 at 25 °C was calculated quantum mechanically from the ratio of vibrational partition functions of the isotopologues at the intermediate (**4D_** ***R*** **·TFA**) and deprotonation transition state (**4_TS-deprot_** ***R*** **·TFA**) levels, using the Eyring equation within the harmonic approximation and different vibrational frequency scaling factors (0.9–1.0).

**Figure 2:**
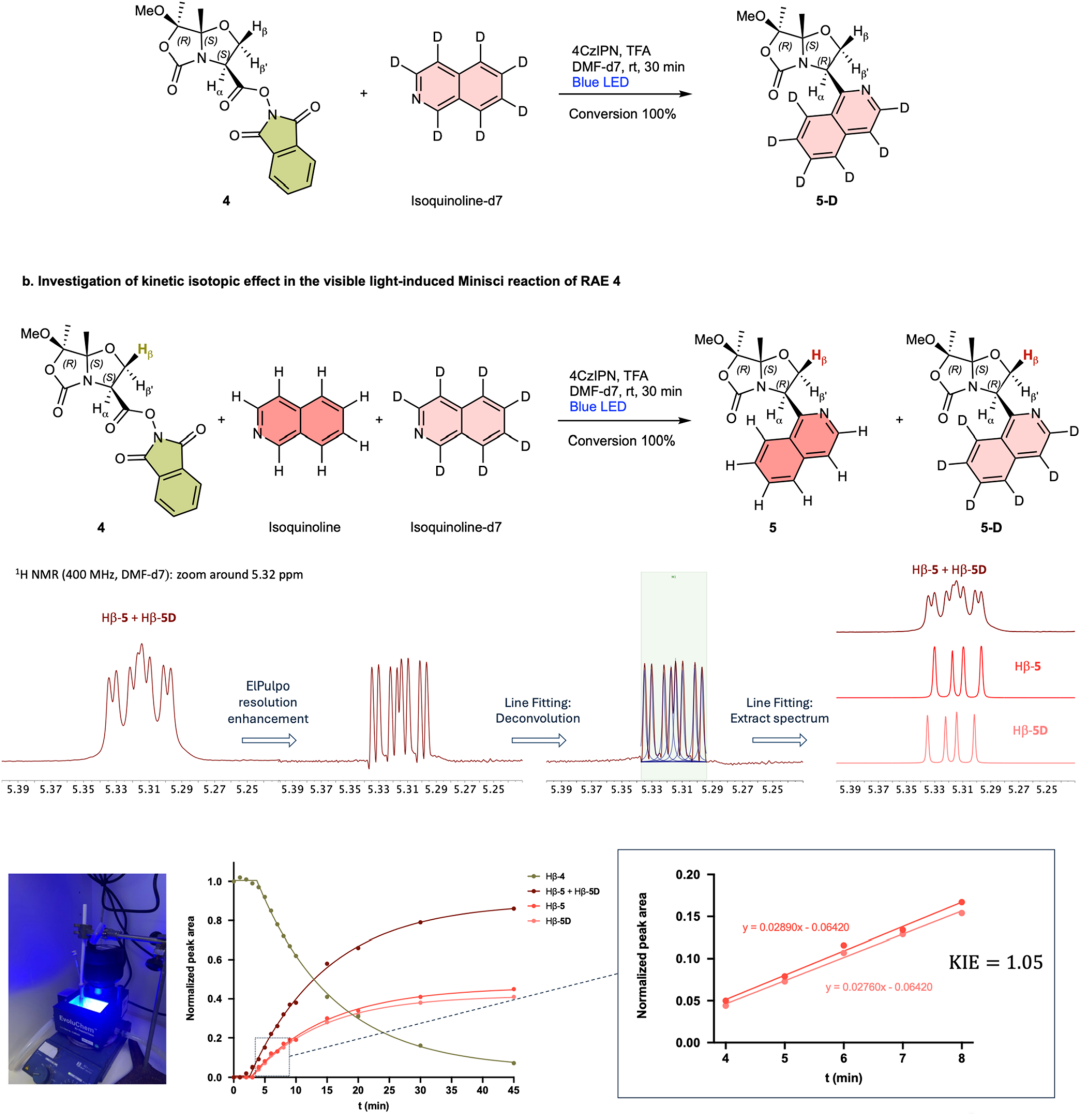
Kinetic study of the photoredox Minisci reaction. (a) Reaction of RAE **4** with fully deuterated isoquinoline to give compound **5-D**. (b) Kinetic isotope effect (KIE) determined from an intermolecular competition reaction of RAE **4** with equimolecular amounts of isoquinoline and isoquinoline-d7 to give products **5** and **5-D**, respectively. In the ^1^H NMR spectra of the products, all signals match in chemical shift except for the doublet of doublets corresponding to the Hβ protons marked in bold. These peaks could be integrated after deconvolution of the spectra with MestReNova software. The data points in the graphs represent the normalized peak area of the corresponding signals associated with the Hβ protons of compounds **4** (decay, in green), **5** (formation, in red), and **5-D** (formation, in light red) during the competition reaction described above, which was carried out in a 5 mm diameter NMR tube using deuterated DMF as solvent and monitored at various reaction times.

## Conclusion

We have developed a general synthetic protocol to access chiral 2-hetaryl-1,2-aminoalcohols via a metal-free diastereoselective photoredox Minisci-type reaction starting from bicyclic *N*,*O*-acetals derived from β-hydroxy-α-amino acids (Ser, Thr, and MeSer). The rigid structure of these substrates confers excellent stereochemical control during the photoredox radical reaction, as also demonstrated simultaneously by prominent laboratories.^17^ In contrast to ionic α-alkylation,^9,10^ the radical α-hetarylation selectively occurs on the concave face of the bicyclic scaffold, presumably directed by dispersion interactions between the incoming heteroarene and the bridgehead methyl group. This effectively replaces the original carboxyl group with a hetaryl group while maintaining complete stereoretention.

In summary, this mild Minisci reaction enables the incorporation of diverse heteroarenes bearing different substituents into the bicyclic scaffold, achieving complete stereoselectivity control over the newly formed bond. In nearly all cases, conversions reach 100%, with yields reported as percentages after purification by column chromatography. These compounds serve as valuable precursors to interesting chiral 2-hetaryl-1,2-aminoalcohols.

## Experimental Section

### General and Experimental Methods

Commercial reagents were used without further purification. Analytical thin layer chromatography (TLC) was performed on Macherey-Nagel precoated aluminum sheets with a 0.20 mm thickness of silica gel 60 with fluorescent indicator UV254. TLC plates were visualized with UV light and by staining with a potassium permanganate solution (0.75 g KMnO_4_, 5 g K_2_CO_3_, and 0.63 mL 10% NaOH in 100 mL water) or a ninhydrin solution (1.5 g ninhydrin in 100 mL of n-butanol and 3.0 mL acetic acid). Column chromatography was performed on silica gel (230–400 mesh). ^1^H and ^13^C­{^1^H} NMR spectra were measured with a 300 or 400 MHz spectrometer with TMS as the internal standard. Multiplicities are quoted as singlet (s), broad singlet (br s), doublet (d), doublet of doublets (dd), triplet (t), or multiplet (m). Spectra were assigned using COSY and HSQC experiments. The results of these experiments were processed with MestreNova software. High resolution electrospray mass (ESI) spectra were recorded on a microTOF spectrometer; accurate mass measurements were achieved by using sodium formate as an external reference. Light-promoted reactions have been carried out at 20 °C using a wavelength of 450 nm in an EvoluChem Photoreactor (by HepatoChem), equipped with a LED lamp (EvoluChem HCK1012–02–008, Batch: 190401–6, S/N: LED0000436). The power supply was AC200–240 V, the lamp power was 30 W and the relative irradiance was 55 mW/cm^2^. We used clear glass vials of volume 4 mL, thread for 13–425, O.D. × H × I.D. Fifteen mm × 45 mm × 8 mm (MERCK, 27111 Supelco). An external fan provides consistent temperature to the reaction.

### Coupling Carboxylic Acids with NHPI

Method 1: After dissolving *N*-hydroxyphthalimide (450 mg, 2.75 mmol, 1.0 equiv) in dichloromethane (1 mL), 2-chloro-1-methylpyridinium iodide (CMPI) (770 mg, 3 mmol, 1.1 equiv) and the corresponding carboxylic acid (3 mmol, 1.1 equiv) were added. Triethylamine (1.3 mL, 9.63 mmol, 3.5 equiv) was then added dropwise at room temperature, and the mixture was stirred for 2.5 h at room temperature. After completion of the reaction, the solvent was evaporated, the product was dissolved in EtOAc and washed with 10% citric acid solution (three times), 5% NaHCO_3_ solution (three times) and brine (three times). The final product was purified by flash column chromatography, using a small amount of silica and light pressure (hexane/ethyl acetate gradient from 100:0 to 1:1). Due to complications encountered during the reaction workup and product purification, an alternative methodology was explored, although it afforded somewhat lower yields. Method 2: In a sealed vial, *N*-hydroxyphthalimide (NHPI) (0.33 g, 2.0 equiv), the corresponding carboxylic acid (4.29 mmol, 1 equiv), propanephosphonic acid anhydride (T3P) (50% in EtOAc, 1.5 mL, 1.2 equiv) and triethylamine (0.35 mL, 1.2 equiv) were dissolved in 2-Me-THF (13.3 mL). The mixture was stirred at 60 °C for 16 h. After completion of the reaction (followed by TLC, hexane/EtOAc 4:1), 2-Me-THF (50 mL) was added to the mixture, and the organic phase was extracted with a 5% NaHCO_3_ solution (50 mL) and washed with brine (50 mL). The organic phase was dried over anhydrous Na_2_SO_4_, filtered, and concentrated under vacuum. The solvent was evaporated, and the crude product was purified by column chromatography (hexane/ethyl acetate gradient from 100:0 to 1:1).

### General Experimental Procedure for the Minisci Reaction Using D Conditions

A dried vial equipped with a Teflon septum and a magnetic stir bar was charged with 4CzIPN (1 mg, 0.01 mmol, 0.01 equiv), the corresponding *N*-heteroarene (0.10 mmol, 1.0 equiv), TFA (10 μL, 0.12 mmol, 1.2 equiv) and vacuum was created. Then anhydrous DMF (1 mL) was added to the vial, and it was irradiated with a blue LED (30 W, λ = 450 nm) for 16 h at room temperature. The corresponding activated ester (0.15 mmol, 1.5 equiv) was freshly synthesized and added in three portions (0.05 mmol each) every 2 h. The reaction was opened to air, diluted with CH_2_Cl_2_, poured into a separatory funnel containing a saturated NaHCO_3_ solution and it was checked that the pH was approximately 8. The organic layer was separated with water, dried with MgSO_4_, filtered and concentrated under vacuo. The crude mixture was purified by column chromatography (hexane/ethyl acetate gradient from 10:0 to 8:2) on silica gel to afford desired products. Only a single diastereomer has been detected in the reaction crudes by ^1^H NMR.

### Computational Details: Quantum Mechanical Calculations

Full geometry optimizations and transition structure (TS) searches were carried out with Gaussian 16 ^20^ using the M06–2X hybrid functional^21^ and 6–311G­(d,p) basis set with ultrafine integration grids. Bulk solvent effects in *N*,*N*-dimethylformamide (DMF) were considered implicitly through the IEF-PCM polarizable continuum model.^22^ The possibility of different conformations was taken into account for all structures. All stationary points were characterized by a frequency analysis performed at the same level used in the geometry optimizations from which thermal corrections were obtained at 298.15 K. The quasiharmonic approximation reported by Truhlar et al. was used to replace the harmonic oscillator approximation for the calculation of the vibrational contribution to enthalpy and entropy.^23^ Scaled frequencies were not considered. Mass-weighted intrinsic reaction coordinate (IRC) calculations were carried out using the Hessian-based predictor-corrector integrator scheme by Hratchian and Schlegel^24^ in order to ensure that the TSs indeed connected the appropriate reactants and products. Free energies calculated using the gas phase standard state concentration (1 atm = 1/24.5 M) were converted to reproduce the standard state concentration in solution (1 M) by subtracting or adding 1.89 kcal mol^–1^ for bimolecular additions and decompositions, respectively. Gibbs free energies (Δ*G*) were used for the discussion on the relative stabilities of the considered structures. The lowest energy conformer for each calculated stationary point (Supporting Figure S3) was considered in the discussion; all the computed structures can be obtained from authors upon request. Noncovalent-interactions (NCI) were calculated with NCIPLOT 4.^25^ Cartesian coordinates, electronic energies, entropies, enthalpies, Gibbs free energies, and lowest frequencies of the calculated structures are summarized in Supporting Table S5.

## Supplementary Material



## Data Availability

The data underlying this study are available in the published article, in its Supporting Information, and openly available in Zenodo at [https://zenodo.org/records/17242344].

## Notes

Experimental procedures, characterization data, copies of the NMR spectra, and computational data (PDF)
