# Supplementary material for: Photocatalytic Minisci Approach to Chiral 2‑Hetaryl-1,2-aminoalcohols from β‑Hydroxy-α-amino Acid-Derived Redox Active Esters
Source: J Org Chem. 2025 Nov 5;90(45):16238–48. doi: 10.1021/acs.joc.5c02485 (PMC12624844; doi:10.1021/acs.joc.5c02485)
Supplement: Supplementary file 1 [file jo5c02485_si_001.pdf]

Supporting Information

Photocatalytic Minisci Approach to Chiral 2-Hetaryl-1,2-Aminoalcohols from β-Hydroxy-α-Amino Acid-Derived Redox Active Esters

Paula Oroz,* Carmen Bretón, Miguel Torres, Iván Olagaray, Eduardo Sainz, Cristina M. Segovia, Alberto Avenoza, Jesús H. Busto, Francisco Corzana, Gonzalo Jiménez-Osés* and Jesús M. Peregrina*

Table of contents

1. General and experimental methods.......... S2

2. General experimental procedures to synthesize activated esters **4**, **6** and **8**.......... S4

3. Optimization of the Minisci reaction with bicyclic activated ester **4** and isoquinoline.......... S7

4. General experimental procedure for the Minisci reaction using D conditions.......... S7

5. Experimental procedure to synthesize compounds **19a** and **19c**.......... S16

6. General experimental procedure to synthesize aminoalcohols **23**, **24**, **25**, **26**.......... S17

7. Scale up of the synthetic procedure to obtain aminoalcohol **23** from
   Boc-L-Ser-OMe derivative **1**.......... S19

8. Radical trapping test with TEMPO.......... S20

9. X Ray analysis.......... S21

10. Proposed mechanism for the Minisci-type reaction.......... S22

11. NMR spectra.......... S24

12. NOESY experiments.......... S93

13. Quantum mechanical calculations.......... S95

14. Kinetic isotopic experiments.......... S117

15. References.......... S123

1. General and experimental methods

Commercial reagents were used without further purification. Analytical thin layer chromatography (TLC) was performed on Macherey-Nagel precoated aluminum sheets with a 0.20 mm thickness of silica gel 60 with fluorescent indicator UV254. TLC plates were visualized with UV light and by staining with a potassium permanganate solution (0.75 g $KMnO_4$, 5 g K_2CO_3, and 0.63 mL 10% NaOH in 100 mL water) or a ninhydrin solution (1.5 g ninhydrin in 100 mL of n-butanol and 3.0 mL acetic acid). Column chromatography was performed on silica gel (230−400 mesh). ^{1}H and ^{13}C{^{1}H} NMR spectra were measured with a 300 or 400 MHz spectrometer with TMS as the internal standard. Multiplicities are quoted as singlet (s), broad singlet (br s), doublet (d), doublet of doublets (dd), triplet (t), or multiplet (m). Spectra were assigned using COSY and HSQC experiments. The results of these experiments were processed with MestreNova software. High resolution electrospray mass (ESI) spectra were recorded on a microTOF spectrometer; accurate mass measurements were achieved by using sodium formate as an external reference.

Light-promoted reactions have been carried out in an EvoluChem PhotoRedOx Box TC™ (Temperature Controlled, by HepatoChem), equipped with a LED lamp (EvoluChem HCK1012-02-008, Batch 190401-6, S/N LED0000436). 20 ºC and wavelength 450 mm. The power supply is AC200-240V, the lamp power is 30 W and the relative irradiance is 55 mW/cm^2. Uses clear glass vials of volume 4 mL, thread for 13-425, O.D. × H × I.D. 15 mm × 45 mm × 8 mm (MERCK, 27111 Supelco). The temperature was controlled with an external chiller/heater module.

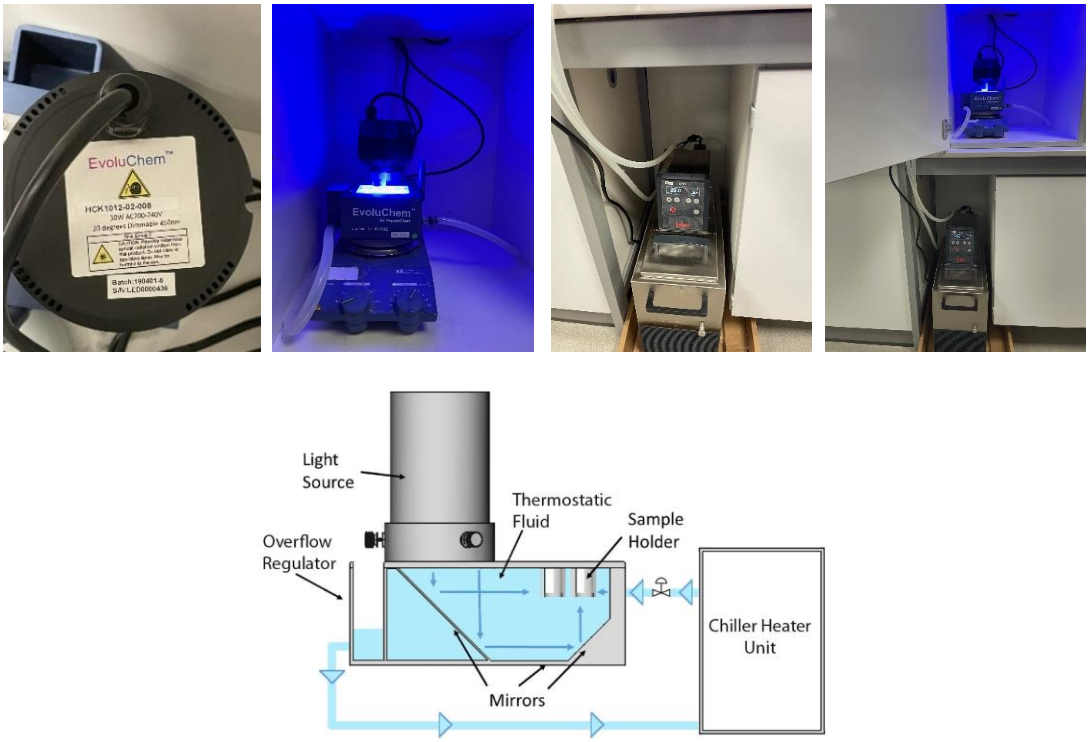

Figure S1. Top: Photoreactor equipped with a LED lamp and a chiller/heater module. Bottom: Schematic representation of the same setup.

HepatoChem
Reinventing Chemistry for Life Sciences

LIGHT MEASUREMENT (PREFORMED WITH 50% FILTER)

Description	Part Number	LED	Batch Number	S/N	Performed By	Date
EvoluChem450DX EU	HCK1012-02-008	CREE XPE	190401-6	LED0000436	SE	04-01-2019

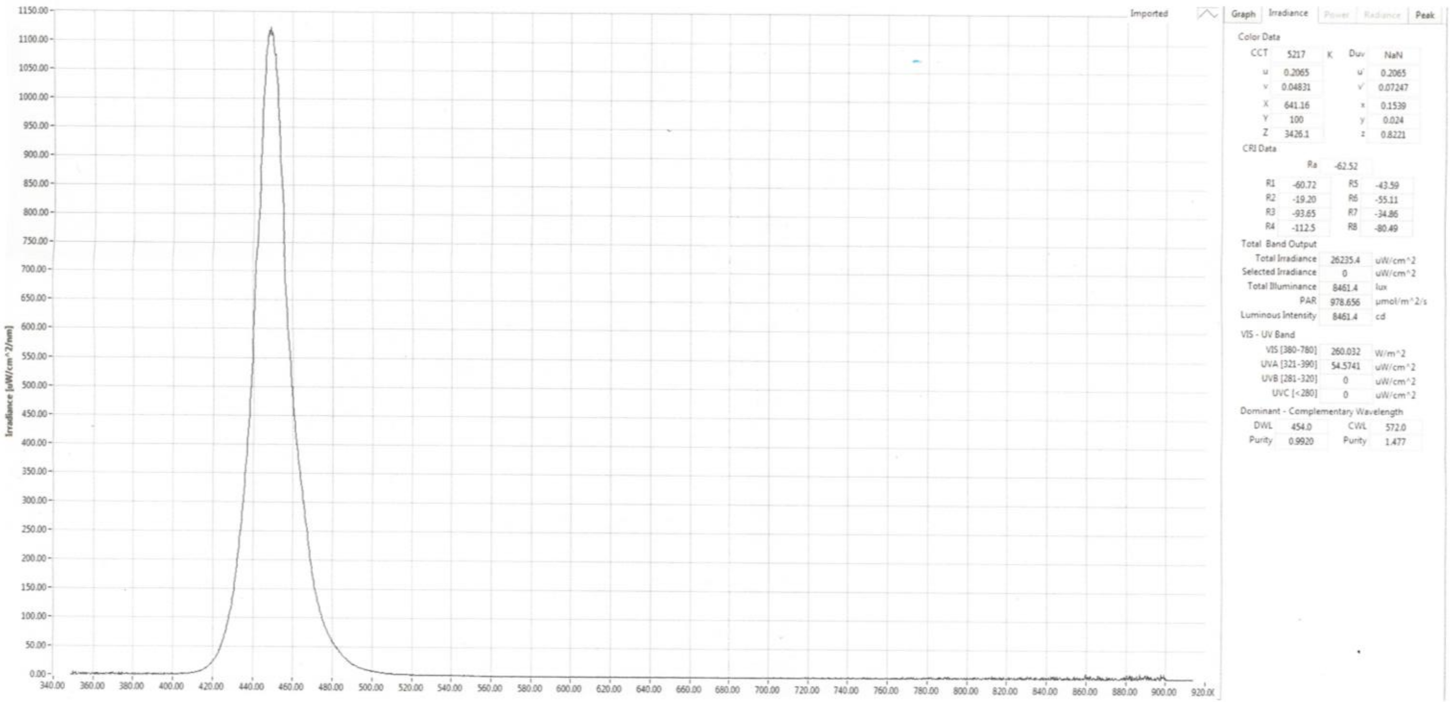

HepatoChem, Inc. 100 Cummings Center, Suite 451C; Beverly, MA 01915 http://www.hepatochem.com ; Tel: (617)-500-5285; Fax: (617)-274-0827

Graphic S1: Light measurement of the LED lamp used (EvoluChem HCK1012-02-008, Batch 190401-6, S/N LED0000436).

2. General experimental procedures to synthesize activated esters 4, 6 and 8.

Garner, P.; Park, J. M. *Org. Synth.* **1992**, *70*, 18

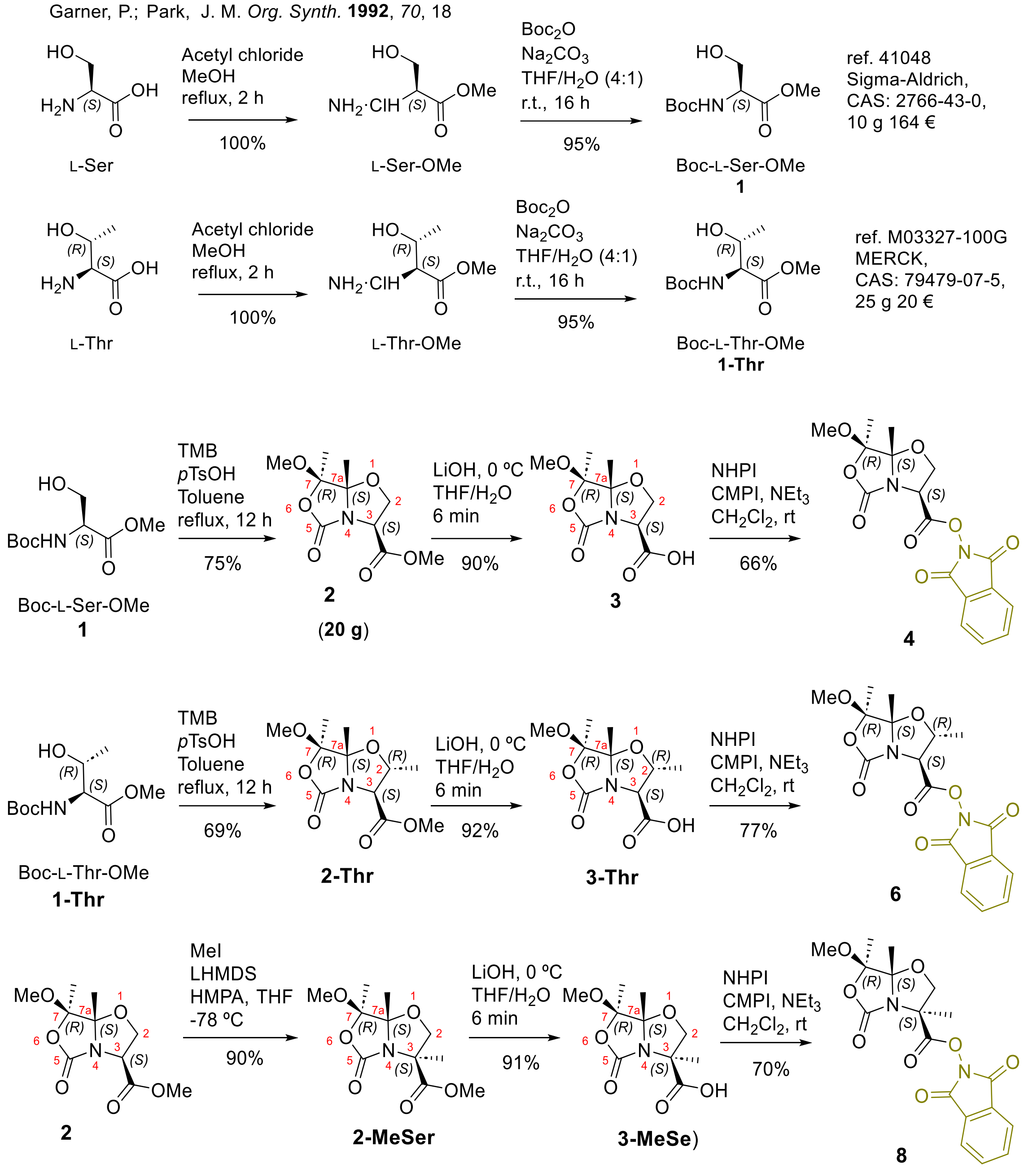

TMB = 2,2,3,3-tetramethoxybutane, CMPI = 2-chloro-1-methylpyridinium iodide, NHPI = *N*-hydroxyphtalimide
LHMDS = lithium hexamethyldisilazane (LHDMS), HMPA = hexamethylphosphotriamide

Scheme S1. Synthesis of RAEs **4**, **6** and **8**.

Method 1 for coupling carboxylic acid 4, 6 or 8 with NHPI:

After dissolving *N*-hydroxyphthalimide (450 mg, 2.75 mmol, 1.0 equiv) in dichloromethane (1 mL), 2-chloro-1-methylpyridinium iodide (CMPI) (770 mg, 3 mmol, 1.1 equiv) and the corresponding carboxylic acid (3 mmol, 1.1 equiv) were added. Triethylamine (1.3 mL, 9.63 mmol, 3.5 equiv) was then added dropwise at room temperature, and the mixture was stirred for 2.5 h at room temperature. After completion of the reaction, the solvent was evaporated, the product was dissolved in EtOAc, and washed with 10% citric acid solution (three times), 5% $NaHCO_3$ solution (three times) and brine (three times). The final product was purified by flash column chromatography, using a small amount of silica and light pressure (hexane/ethyl acetate gradient from 100:0 to 1:1).

Due to the complications encountered when performing the workup of the reaction and purifying the obtained products, an alternative methodology was sought, although the yields obtained were somewhat lower.

Method 2 for coupling carboxylic acid 4, 6 or 8 with NHPI:

In a sealed vial, *N*-hydroxyphthalimide (NHPI) (0.33 g, 2.0 equiv), the corresponding carboxylic acid (4.29 mmol, 1 equiv), propanephosphonic acid anhydride (T3P) (50% in EtOAc, 1.5 mL, 1.2 equiv) and triethylamine (0.35 mL, 1.2 equiv) were dissolved in 2-Me-THF (13.3 mL). The mixture was stirred at 60 °C for 16 h. After completion of the reaction (followed by TLC, hexane/EtOAc 4:1), 2-Me-THF (50 mL) was added to the mixture and the organic phase was extracted with a 5% $NaHCO_3$ solution (50 mL) and washed with brine (50 mL). The organic phase was dried over anhydrous Na_2SO_4, filtered, and concentrated under vacuum. The solvent was evaporated, and the crude product was purified by column chromatography (hexane/ethyl acetate gradient from 100:0 to 1:1).

1,3-Dioxoisoindolin-2-yl (3*S*,7*R*,7a*S*)-7-methoxy-7,7a-dimethyl-5-oxotetrahydro-5*H*-oxazolo[4,3-b]oxazole-3-carboxylate

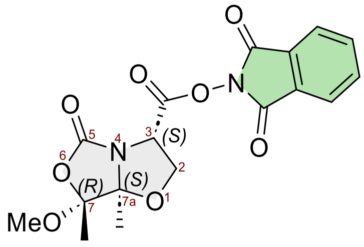

4

Product **4** (1 g, 2.7 mmol, 66% yield) was obtained from carboxylic acid **3** as a white solid (mp 91-96 °C), which was synthesized following a previously published method.[S3] $[\alpha]^{20}_{D}$ -97.5 (c 1.0, $CHCl_3$). HRMS (ESI) m/z: $[M + Na]^+$ Calcd for $C_{17}H_{16}N_2NaO_8$: 399.0799; Found 399.0801. ^1H NMR ($CDCl_3$, 400 MHz): δ 7.90 (dd, *J* = 5.5, 3.1 Hz, 2H, $2H^{Ar, Pht}$), 7.82 (dd, *J* = 5.5, 3.1 Hz, 2H, $2H^{Ar, Pht}$), 5.18 (dd, *J* = 9.1, 5.8 Hz, 1H, $C\underline{H}^{\alpha}$), 4.47 (t, *J* = 9.1 Hz, 1H, $1C\underline{H}_2^{\beta}$), 4.36 (dd, *J* = 9.0, 5.8 Hz, 1H, $C\underline{H}_2^{\beta}$), 3.48 (s, 3H, $OC\underline{H}_3$), 1.61 (s, 3H, $C\underline{H}_3^{7}$), 1.46 (s, 3H, $C\underline{H}_3^{7a}$). $^{13}C\{^1H\}$ NMR ($CDCl_3$, 100 MHz): δ 167.2, 161.5, 160.3 (4CO),

135.2 ($2C^{Ar, Pht}$), 128.9 ($2C^{*Pht}$), 124.3 ($2C^{Ar, Pht}$), 107.5 (C^{7}), 102.2 (C^{7a}), 66.7 (C^{β}), 58.4 (C^{α}), 51.3 ($O\underline{C}H_3$), 16.1 ($O\underline{C}H_3^{7a}$), 15.6 ($O\underline{C}H_3^{7}$).

1,3-Dioxoisoindolin-2-yl (2*R*,3*S*,7*R*,7a*S*)-7-methoxy-2,7,7a-trimethyl-5-oxotetrahydro-5*H*-oxazolo[4,3-b]oxazole-3-carboxylate

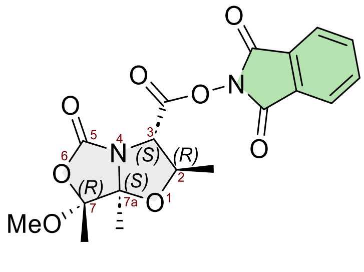

6

Product **6** (1.29 g, 3.3 mmol, 77% yield) was obtained from carboxylic acid **3-Thr** as a white solid (mp 87-92 °C), which was synthesized following a previously published method that involves *N,O*-bicyclic acetal formation of Boc-L-Thr-OMe followed by hydrolysis with LiOH.[S4] $[\alpha]^{20}_{D}$ -91.3 (c 1.0, $CHCl_3$). HRMS (ESI) m/z: $[M + Na]^+$ Calcd for $C_{18}H_{18}N_2NaO_8$: 413.0955; Found 413.0973. ^1H NMR ($CDCl_3$, 400 MHz): δ 7.88 (dd, *J* = 5.5, 3.1 Hz, 2H, $2H^{Ar, Pht}$), 7.80 (dd, *J* = 5.5, 3.1 Hz, 2H, $2H^{Ar, Pht}$), 4.63-4.62 (m, 2H, $C\underline{H}^{\alpha}$, $C\underline{H}_2^{\beta}$), 3.45 (s, 3H, $OC\underline{H}_3$), 1.60 (s, 3H, $C\underline{H}_3^{7}$), 1.52 (d, *J* = 5.9 Hz, 3H, $C\underline{H}_3^{2}$), 1.45 (s, 3H, $C\underline{H}_3^{7a}$). $^{13}C\{^1H\}$ NMR ($CDCl_3$, 100 MHz): δ 166.9, 161.5, 160.5 (4CO), 135.1 ($2C^{Ar, Pht}$), 128.8 ($2C^{*Pht}$), 124.2 ($2C^{Ar, Pht}$), 107.3 (C^{7}), 102.3 (C^{7a}), 76.6 (C^{β}), 65.1 (C^{α}), 51.1 ($O\underline{C}H_3$), 20.6 ($\underline{C}H_3^{2}$), 16.3 ($\underline{C}H_3^{7a}$), 15.7 ($\underline{C}H_3^{7}$).

1,3-Dioxoisoindolin-2-yl (3*S*,7*R*,7a*S*)-7-methoxy-3,7,7a-trimethyl-5-oxotetrahydro-5*H*-oxazolo[4,3-b]oxazole-3-carboxylate

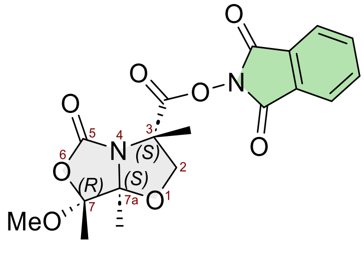

8

Product **8** (1.2 g, 3.0 mmol, 70% yield) was obtained from carboxylic acid **3-MeSer** as a white solid (mp 105-110 °C), which was synthesized following a previously published method that involves diastereoselective α-methylation of *N,O*-bicyclic serine equivalent **2** followed by hydrolysis with LiOH.[S3b] $[\alpha]^{20}_{D}$ -154.6 (c 1.0, $CHCl_3$). HRMS (ESI) m/z: $[M + Na]^+$ Calcd for $C_{18}H_{18}N_2NaO_8$: 413.0955; Found 413.0955. ^1H NMR ($CDCl_3$, 400 MHz): δ 7.89 (dd, *J* = 5.5, 3.1 Hz, 2H, $2H^{Ar, Pht}$), 7.80 (dd, *J* = 5.5, 3.1 Hz, 2H, $2H^{Ar, Pht}$), 4.72 (d, *J* = 9.1 Hz, 1H, $1C\underline{H}_2^{\beta}$), 3.98 (d, *J* = 9.1 Hz, 1H, $C\underline{H}_2^{\beta}$), 3.47 (s, 3H, $OC\underline{H}_3$), 2.06 (s, 3H, $C\underline{H}_3^{3}$), 1.57 (s, 3H, $C\underline{H}_3^{7}$), 1.49 (s, 3H, $C\underline{H}_3^{7a}$). $^{13}C\{^1H\}$ NMR ($CDCl_3$, 100 MHz): δ 169.9, 161.5,

155.1 (4CO), 135.1 (2C$^{Ar, Pht}$), 129.0 (2C*^{Pht}), 124.2 (2C$^{Ar, Pht}$), 106.8 (C^{7}), 103.3 (C^{7a}), 76.1 (C$^{\beta}$), 65.9 (C$^{\alpha}$), 51.5 (O<u>C</u>H$_3$), 20.9 (<u>C</u>H$_3$^{3}), 17.7 (<u>C</u>H$_3$^{7a}), 16.4 (<u>C</u>H$_3$^{7}).

3. Optimization of the Minisci reaction with bicyclic activated ester 4 and isoquinoline.

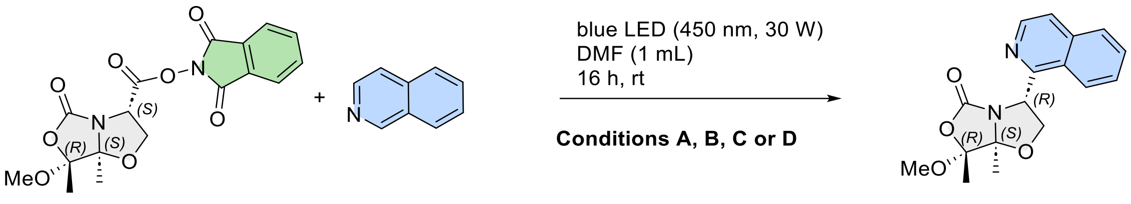

4
(0.15 mmol, 1.5 equiv)

Isoquinoline
(0.1 mmol, 1 equiv)

5

rac-PA: 1,1-Binaphtyl-2,2-diyl hydrogenphosphate
Cat-Ir: Ir[dF(CF_3)ppy]$_2$dtbbpyPF$_6$

Scheme S2. Minisci reaction between RAE **4** and isoquinoline.

Table S1. Reaction conditions for the synthesis of compound **5** and yields.

Procedure	Acid*	Catalyst*	Solvent	Yield
A	*rac*-PA (0.01 mmol, 0.1 equiv)	Cat-Ir (0.001 mmol, 0.01 equiv)	DMA (1 mL)	53%
B	In(OTf)$_3$ (0.01 mmol, 0.1 equiv)	Cat-Ir (0.001 mmol, 0.01 equiv)	DMA (1 mL)	45%
C	$NaHSO_4$ (0.10 mmol, 1.0 equiv)	None	DMF (1 mL)	<10%
D	TFA (0.12 mmol, 1.2 equiv)	4CzIPN (0.001 mmol, 0.01 equiv)	DMF (1 mL)	88%
E	TFA (0.12 mmol, 1.2 equiv)	4CzIPN (0.001 mmol, 0.01 equiv)	CH_2Cl_2 (1 mL)	<10%

*All acids and catalysts are commercially available. In particular, **Cat-Ir**: **[Ir[dF(CF_3)ppy]$_2$(dtbbpy)]PF$_6$** was purchased from TCI, CAS RN: 870987-63-6, Product Number: D5817, https://www.tcichemicals.com/OP/en/p/D5817, and **4CzIPN** from BLDpharm, CAS No. 1416881-52-1, Cat. No.: BD00787257, https://www.bldpharm.com/products/1416881-52-1.html

4. General experimental procedure for the Minisci reaction using D conditions.

A dried vial equipped with a Teflon septum and a magnetic stir bar was charged with 4CzIPN (1 mg, 0.01 mmol, 0.01 equiv), the corresponding *N*-heteroarene (0.10 mmol, 1.0 equiv), TFA (10 µL, 0.12 mmol, 1.2 equiv) and vacuum was created. Then anhydrous DMF (1 mL) was added to the vial, and it was irradiated with a blue LED (30 W, λ = 450 nm) for 16 h at room temperature. The corresponding

activated ester (0.15 mmol, 1.5 equiv) was **freshly synthesized** and added in three portions (0.05 mmol each) every 2 h. The reaction was opened to air, diluted with CH_2Cl_2, poured into a separatory funnel containing a saturated $NaHCO_3$ solution and it was checked that the pH was approximately 8. The organic layer was separated with water, dried with $MgSO_4$, filtered and concentrated under vacuo. The crude mixture was purified by column chromatography (hexane/ethyl acetate gradient from 10/0 to 8/2) on silica gel to afford desired products. Procedure based on that described in a previous work.[S2] Only a single diastereomer has been detected in the reaction crudes by ^{1}H NMR.

(3*R*,7*R*,7a*S*)-3-(Isoquinolin-1-yl)-7-methoxy-7,7a-dimethyltetrahydro-5*H*-oxazolo[4,3-b]oxazol-5-one

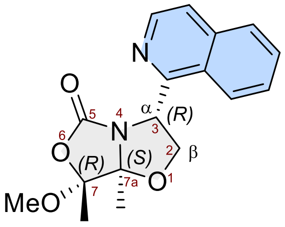

5

Following the above general procedure, the activated ester **4** was treated with isoquinoline affording product **5** as a white solid (41.5 mg, 0.132 mmol, 88% yield, mp 117-120 °C). $[\alpha]^{20}_{D}$ -92.5 (c 1.0, $CHCl_3$). HRMS (ESI) m/z: $[M + H]^+$ Calcd for $C_{17}H_{19}N_2O_4$: 315.1339; Found 315.1341. ^{1}H NMR ($CDCl_3$, 400 MHz): δ 8.86, 8.48, 7.85, 7.71, 7.64 (6H, H^{Ar}), 6.06 (dd, *J* = 8.1, 4.8 Hz, 1H, $C\underline{H}^{\alpha}$), 5.44 (dd, *J* = 8.2, 4.8 Hz, 1H, $1C\underline{H}_2^{\beta}$), 4.32 (t, *J* = 8.1 Hz, 1H, $1C\underline{H}_2^{\beta}$), 3.41 (s, 3H, $OC\underline{H}_3$), 1.64 (s, 3H, $C\underline{H}_3^{7}$), 1.21 (s, 3H, $C\underline{H}_3^{7a}$). $^{13}C\{^1H\}$ NMR ($CDCl_3$, 100 MHz): δ 162.0 (CO), 155.4 (C^{*Ar}), 140.8 (C^{*Ar}), 136.8 (C^{*Ar}), 130.4 (C^{Ar}), 128.1 (C^{Ar}), 127.8 (C^{Ar}), 127.4 (C^{Ar}), 125.6 (C^{Ar}), 121.6 (C^{Ar}), 107.4 (C^{7}), 101.4 (C^{7a}), 66.1 (C^{β}), 60.8 (C^{α}), 51.1 ($O\underline{C}H_3$), 17.6 ($\underline{C}H_3^{7a}$), 15.7 ($\underline{C}H_3^{7}$).

(2*R*,3*R*,7*R*,7a*S*)-3-(Isoquinolin-1-yl)-7-methoxy-2,7,7a-trimethyltetrahydro-5*H*-oxazolo[4,3-b]oxazol-5-one

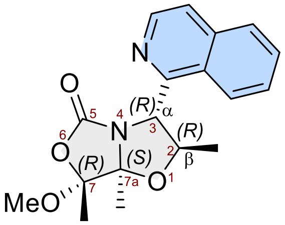

7

Following the above general procedure, the activated ester **6** was treated with isoquinoline affording product **7** as a colorless oil (37.9 mg, 0.116 mmol, 77% yield). $[\alpha]^{20}_{D}$ -87.4 (c 1.0, $CHCl_3$). HRMS (ESI) m/z: $[M + Na]^+$ Calcd for $C_{18}H_{20}N_2NaO_4$: 351.1315; Found 351.1322. ^{1}H NMR ($CDCl_3$, 400 MHz): δ 8.92, 8.48, 7.84, 7.68-7.72, 7.63 (6H, H^{Ar}), 5.90 (dd, *J* = 6.2, 4.8 Hz, 1H, $C\underline{H}^{\beta}$), 5.56 (d, *J* = 4.8 Hz, 1H, $C\underline{H}^{\alpha}$), 3.39 (s, 3H, $OC\underline{H}_3$), 1.66 (s, 3H, $C\underline{H}_3^{7}$), 1.46 (d, *J* = 4.8 Hz, 3H, $C\underline{H}_3^{2}$), 1.27 (s, 3H, $C\underline{H}_3^{7a}$). $^{13}C\{^1H\}$

NMR ($CDCl_3$, 100 MHz): δ 162.4 (CO), 155.4 (C^{*Ar}), 140.8 (C^{*Ar}), 136.7 (C^{*Ar}), 130.4 (C^{Ar}), 128.1 (C^{Ar}), 128.0 (C^{Ar}), 127.3 (C^{Ar}), 125.8 (C^{Ar}), 121.6 (C^{Ar}), 107.1 (C^{7}), 101.6 (C^{7a}), 74.5 (C^{β}), 67.5 (C^{α}), 51.0 ($O\underline{C}H_3$), 20.8 ($\underline{C}H_3{}^{2}$), 17.7 ($\underline{C}H_3{}^{7a}$), 15.8 ($\underline{C}H_3{}^{7}$).

(3*R*,7*R*,7a*S*)-3-(Isoquinolin-1-yl)-7-methoxy-3,7,7a-trimethyltetrahydro-5*H*-oxazolo[4,3-b]oxazol-5-one

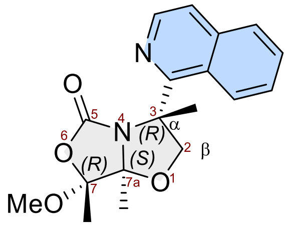

9

Following the above general procedure, the activated ester **8** was treated with isoquinoline affording product **9** as a colorless oil (34.5 mg, 0.105 mmol, 70% yield). $[\alpha]^{20}_{D}$ -170.0 (c 1.0, $CHCl_3$). HRMS (ESI) m/z: $[M + Na]^+$ Calcd for $C_{18}H_{20}N_2NaO_4$: 351.1315; Found 351.1319. ^1H NMR ($CDCl_3$, 400 MHz): δ 8.93, 8.43, 7.83, 7.59-7.70 (6H, H^{Ar}), 5.91 (d, *J* = 8.1 Hz, 1H, $C\underline{H}^{\beta}$), 3.82 (d, *J* = 8.1 Hz, 1H, $1C\underline{H}_2{}^{\beta}$), 3.41 (s, 3H, $OC\underline{H}_3$), 2.05 (s, 3H, $C\underline{H}_3{}^{3}$), 1.60 (s, 3H, $C\underline{H}_3{}^{7}$), 0.70 (s, 3H, $C\underline{H}_3{}^{7a}$). $^{13}C\{^1H\}$ NMR ($CDCl_3$, 100 MHz): δ 159.9 (CO), 156.5 (C^{*Ar}), 140.4 (C^{*Ar}), 137.5 (C^{*Ar}), 130.1 (C^{Ar}), 127.7 (C^{Ar}), 127.5 (C^{Ar}), 126.5 (C^{Ar}), 125.9 (C^{Ar}), 121.5 (C^{Ar}), 107.2 (C^{7}), 102.5 (C^{7a}), 76.9 (C^{β}), 69.8 (C^{α}), 51.4 ($O\underline{C}H_3$), 24.3 ($\underline{C}H_3{}^{3}$), 19.1 ($\underline{C}H_3{}^{7a}$), 16.6 ($\underline{C}H_3{}^{7}$).

(3*R*,7*R*,7a*S*)-7-methoxy-3-(5-methoxyisoquinolin-1-yl)-7,7a-dimethyltetrahydro-5*H*-oxazolo[4,3-b]oxazol-5-one

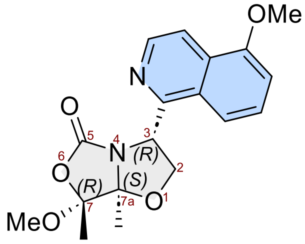

10

Following the above general procedure, the activated ester **4** was treated with 5-methoxyisoquinoline affording product **10** as a white solid (22.4 mg, 0.065 mmol, 65% yield, mp 47-50 °C). $[\alpha]^{20}_{D}$ -121.5 (c 1.0, $CHCl_3$). HRMS (ESI) m/z: $[M + Na]^+$ Calcd for $C_{18}H_{20}N_2NaO_5$ 367.1264; Found 367.1268. ^1H NMR ($CDCl_3$, 300 MHz): δ 8.50, 8.38, 8.07, 7.61, 7.06 (5H, H^{Ar}), 6.05 (dd, *J* = 8.00, 4.8 Hz, 1H, $C\underline{H}^{\alpha}$), 5.41 (dd, *J* = 8.2, 4.8 Hz, 1H, $1C\underline{H}_2{}^{\beta}$), 4.34 (t, *J* = 8.1 Hz, 1H, $1C\underline{H}_2{}^{\beta}$), 4.04 (s, 3H, $OC\underline{H}_3{}^{Ar}$), 3.43 (s, 3H, $OC\underline{H}_3$), 1.66 (s, 3H, $C\underline{H}_3{}^{7}$), 1.23 (s, 3H, $C\underline{H}_3{}^{7a}$). $^{13}C\{^1H\}$ NMR ($CDCl_3$, 100 MHz): δ 161.8 (CO), 154.9 (C^{*Ar}), 154.6 (C^{*Ar}), 140.4 (C^{Ar}), 129.3 (C^{*Ar}), 128.3 (C^{*Ar}), 128.0 (C^{Ar}), 117.1 (C^{Ar}), 115.7 (C^{Ar}), 107.6 (C^{Ar}), 107.3 (C^{7}), 101.3 (C^{7a}), 66.2 (C^{β}), 60.9 (C^{α}), 55.7 ($O\underline{C}H_3{}^{Ar}$), 50.9 ($O\underline{C}H_3$), 17.5 ($\underline{C}H_3{}^{7a}$), 15.6 ($\underline{C}H_3{}^{7}$).

(3*R*,7*R*,7a*S*)-3-(5-bromoisoquinolin-1-yl)-7-methoxy-7,7a-dimethyltetrahydro-5*H*-oxazolo[4,3-b]oxazol-5-one

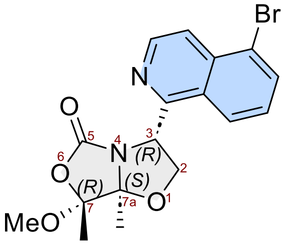

11

Following the above general procedure, the activated ester **4** was treated with 5-bromoisoquinoline affording product **11** as a white solid (31.1 mg, 0.079 mmol, 79% yield, mp 121-124 °C). $[\alpha]^{20}_{D}$ -154.6 (c 1.0, $CHCl_3$). HRMS (ESI) m/z: $[M + Na]^+$ Calcd for $C_{17}H_{17}BrN_2NaO_4$: 415.0264; Found 415.0266. ^1H NMR ($CDCl_3$, 300 MHz): δ 8.91, 8.61, 8.04, 7,57 (5H, H^{Ar}), 6.06 (dd, *J* = 8.0, 4.7 Hz, 1H, $C\underline{H}^{\alpha}$), 5.49 (dd, *J* = 8.3, 4.8 Hz, 1H, $1C\underline{H}_2^{\beta}$), 4.34 (t, *J* = 8.1 Hz, 1H, $1C\underline{H}_2^{\beta}$), 3.43 (s, 3H, $OC\underline{H}_3$), 1.66 (s, 3H, $C\underline{H}_3^{7}$), 1.20 (s, 3H, $C\underline{H}_3^{7a}$). $^{13}C\{^1H\}$ NMR ($CDCl_3$, 100 MHz): δ 161.8 (CO), 155.7 (C^{*Ar}), 142.0 (C^{Ar}), 135.8 (C^{*Ar}), 134.2 (C^{Ar}), 128.9 (C^{*Ar}), 128.3 (C^{Ar}), 125.3 (C^{Ar}), 122.2 (C^{*Ar}), 120.4 (C^{Ar}), 107.4 (C^{7}), 101.3 (C^{7a}), 66.0 (C^{β}), 60.6 (C^{α}), 51.0 ($O\underline{C}H_3$), 17.5 ($\underline{C}H_3^{7a}$), 15.5 ($\underline{C}H_3^{7}$).

(3*R*,7*R*,7a*S*)-3-(6-bromoisoquinolin-1-yl)-7-methoxy-7,7a-dimethyltetrahydro-5*H*-oxazolo[4,3-b]oxazol-5-one

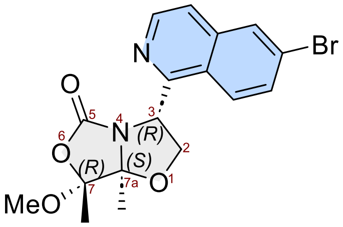

12

Following the above general procedure, the activated ester **4** was treated with 6-bromoisoquinoline affording product **12** as a colorless oil (28.3 mg, 0.072 mmol, 72% yield). $[\alpha]^{20}_{D}$ -148.6 (c 1.0, $CHCl_3$). HRMS (ESI) m/z: $[M + Na]^+$ Calcd for $C_{17}H_{17}BrN_2NaO_4$: 415.0264; Found 415.0245. ^1H NMR ($CDCl_3$, 300 MHz): δ 8.80, 8.52, 8.04, 7,79, 7.57 (5H, H^{Ar}), 6.01 (dd, *J* = 8.0, 4.8 Hz, 1H, $C\underline{H}^{\alpha}$), 5.49 (dd, *J* = 8.3, 4.8 Hz, 1H, $1C\underline{H}_2^{\beta}$), 4.33 (t, *J* = 8.2 Hz, 1H, $1C\underline{H}_2^{\beta}$), 3.43 (s, 3H, $OC\underline{H}_3$), 1.66 (s, 3H, $C\underline{H}_3^{7}$), 1.19 (s, 3H, $C\underline{H}_3^{7a}$). $^{13}C\{^1H\}$ NMR ($CDCl_3$, 100 MHz): δ 161.8 (CO), 155.6 (C^{*Ar}), 141.7 (C^{Ar}), 137.8 (C^{*Ar}), 131.5 (C^{Ar}), 129.3 (C^{Ar}), 127.5 (C^{Ar}), 126.1 (C^{*Ar}), 125.3 (C^{*Ar}), 120.5 (C^{Ar}), 107.4 (C^{7}), 101.2 (C^{7a}), 65.7 (C^{β}), 60.5 (C^{α}), 51.0 ($O\underline{C}H_3$), 17.4 ($\underline{C}H_3^{7a}$), 15.5 ($\underline{C}H_3^{7}$).

(3*R*,7*R*,7a*S*)-3-(7-bromoisoquinolin-1-yl)-7-methoxy-7,7a-dimethyltetrahydro-5*H*-oxazolo[4,3-b]oxazol-5-one

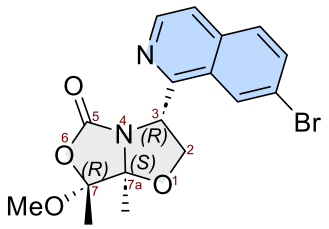

13

Following the above general procedure, the activated ester **4** was treated with 7-bromoisoquinoline affording product **13** as a colorless oil (27.5 mg, 0.070 mmol, 70% yield). $[\alpha]^{20}_{D}$ -182.3 (c 1.0, $CHCl_3$). HRMS (ESI) m/z: $[M + Na]^+$ Calcd for $C_{17}H_{17}BrN_2NaO_4$: 415.0264; Found 415.0270. ^{1}H NMR ($CDCl_3$, 300 MHz): δ 9.10, 8.49, 7.79, 7,72, 7.61 (5H, H^{Ar}), 5.95 (dd, *J* = 8.0, 4.8 Hz, 1H, $C\underline{H}^{\alpha}$), 5.43 (dd, *J* = 8.3, 4.8 Hz, 1H, $1C\underline{H}_2{}^{\beta}$), 4.31 (t, *J* = 8.2 Hz, 1H, $1C\underline{H}_2{}^{\beta}$), 3.42 (s, 3H, $OC\underline{H}_3$), 1.64 (s, 3H, $C\underline{H}_3{}^{7}$), 1.19 (s, 3H, $C\underline{H}_3{}^{7a}$). $^{13}C\{^1H\}$ NMR ($CDCl_3$, 100 MHz): δ 161.9 (CO), 154.7 (C^{*Ar}), 141.2 (C^{Ar}), 135.2 (C^{*Ar}), 134.1 (C^{Ar}), 129.0 (C^{Ar}), 128.7 (C^{*Ar}), 128.2 (C^{Ar}), 122.1 (C^{*Ar}), 121.3 (C^{Ar}), 107.4 (C^7), 101.4 (C^{7a}), 65.9 (C^{β}), 60.7 (C^{α}), 51.1 ($O\underline{C}H_3$), 17.5 ($\underline{C}H_3{}^{7a}$), 15.6 ($\underline{C}H_3{}^{7}$).

(3*R*,7*R*,7a*S*)-3-(4-hydroxyisoquinolin-1-yl)-7-methoxy-7,7a-dimethyltetrahydro-5*H*-oxazolo[4,3-b]oxazol-5-one

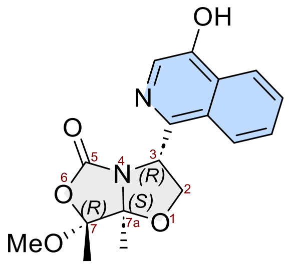

14

Following the above general procedure, the activated ester **4** was treated with isoquinolin-4-ol affording product **14** as a white solid (13.2 mg, 0.040 mmol, 40% yield, mp 94-97 °C). $[\alpha]^{20}_{D}$ -70.9 (c 1.0, $CHCl_3$). HRMS (ESI) m/z: $[M + Na]^+$ Calcd for $C_{17}H_{19}N_2NaO_5$: 354.1186; Found 354.1153. ^{1}H NMR ($CDCl_3$, 300 MHz): δ 8.66, 8.19, 8.09, 7.67 (5H, H^{Ar}), 5.95 (dd, *J* = 8.0, 4.9 Hz, 1H, $C\underline{H}^{\alpha}$), 5.24 (dd, *J* = 8.3, 4.9 Hz, 1H, $1C\underline{H}_2{}^{\beta}$), 4.32 (t, *J* = 8.1 Hz, 1H, $1C\underline{H}_2{}^{\beta}$), 3.37 (s, 3H, $OC\underline{H}_3$), 1.62 (s, 3H, $C\underline{H}_3{}^{7}$), 1.20 (s, 3H, $C\underline{H}_3{}^{7a}$). $^{13}C\{^1H\}$ NMR ($CDCl_3$, 100 MHz): δ 162.2 (CO), 148.4 (C^{*Ar}), 146.8 (C^{*Ar}), 129.5 (C^{*Ar}), 128.4 (C^{Ar}), 128.3 (C^{*Ar}), 128.1 (C^{*Ar}), 125.0 (C^{Ar}), 124.6 (C^{Ar}), 121.8 (C^{Ar}), 107.7 (C^7), 101.4 (C^{7a}), 66.5 (C^{β}), 60.6 (C^{α}), 51.1 ($O\underline{C}H_3$), 17.5 ($\underline{C}H_3{}^{7a}$), 15.7 ($\underline{C}H_3{}^{7}$).

(1-((3*R*,7*R*,7a*S*)-7-methoxy-7,7a-dimethyl-5-oxotetrahydro-5*H*-oxazolo[4,3-b]oxazol-3-yl)isoquinolin-6-yl)boronic acid

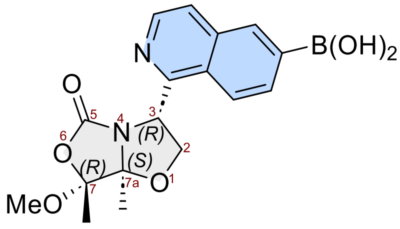

15

Following the above general procedure, the activated ester **4** was treated with isoquinolin-6-ylboronic acid and the crude mixture was purified by column chromatography (100% ethyl acetate) on silica gel affording product **15** as a colorless oil (34.4 mg, 0.096 mmol, 96% yield). $[\alpha]^{20}_{D}$ -74.3 (c 1.0, H_2O). HRMS (ESI) m/z: $[M + Na]^+$ Calcd for $C_{17}H_{19}BN_2NaO_6$: 381.1228; Found 381.1234. ^1H NMR (DMF-d7, 300 MHz): δ 8.84, 8.62, 8.56, 8.19, 7.90 (5H, H^{Ar}), 6.16 (dd, *J* = 8.1, 5.1 Hz, 1H, $C\underline{H}^{\alpha}$), 5.33 (dd, *J* = 8.2, 5.1 Hz, 1H, $1C\underline{H}_2^{\beta}$), 4.50 (t, *J* = 8.1 Hz, 1H, $1C\underline{H}_2^{\beta}$), 3.42 (s, 3H, $OC\underline{H}_3$), 1.65 (s, 3H, $C\underline{H}_3^{7}$), 1.22 (s, 3H, $C\underline{H}_3^{7a}$). $^{13}C\{^1H\}$ NMR (DMF-d7, 100 MHz): δ 162.5 (CO), 156.6 (C^{*Ar}), 141.8 (C^{Ar}), 137.0 (C^{*Ar}), 135.4 (C^{Ar}), 133.5 (C^{Ar}), 129.1 (C^{*Ar}), 124.6 (C^{Ar}), 123.0 (C^{Ar}), 108.1 (C^{7}), 102.1 (C^{7a}), 67.0 (C^{β}), 61.7 (C^{α}), 51.6 ($O\underline{C}H_3$), 17.9 ($\underline{C}H_3^{7a}$), 15.9 ($\underline{C}H_3^{7}$).

(3*R*,7*R*,7a*S*)-7-Methoxy-7,7a-dimethyl-3-(4-methylquinolin-2-yl)tetrahydro-5*H*-oxazolo[4,3-b]oxazol-5-one

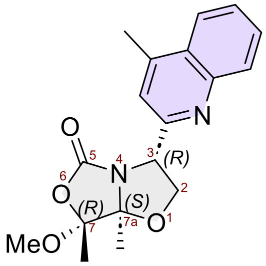

16

Following the above general procedure, the activated ester **4** was treated with lepidine affording product **16** as a colorless oil (41.4 mg, 0.126 mmol, 84% yield). $[\alpha]^{20}_{D}$ -207.1 (c 1.0, $CHCl_3$). HRMS (ESI) m/z: $[M + Na]^+$ Calcd for $C_{18}H_{20}N_2NaO_4$: 351.1315; Found 351.1330. ^1H NMR ($CDCl_3$, 400 MHz): δ 8.01, 7.71, 7.58 (6H, H^{Ar}), 5.43 (dd, *J* = 8.5, 5.7 Hz, 1H, $C\underline{H}^{\alpha}$), 4.86 (dd, *J* = 8.8, 5.7 Hz, 1H, $C\underline{H}_2^{\beta}$), 4.60 (t, *J* = 8.6 Hz, $C\underline{H}_2^{\beta}$), 3.51 (s, 3H, $OC\underline{H}_3$), 2.73 - 2.72 (m, 3H, $C\underline{H}_3^{Ar}$), 1.64 (s, 3H, $C\underline{H}_3^{7}$), 1.17 (s, 3H, $C\underline{H}_3^{7a}$). $^{13}C\{^1H\}$ NMR ($CDCl_3$, 100 MHz): δ 161.9 (4CO), 158.9 (C^{*Ar}), 147.4 (C^{*Ar}), 145.4 (C^{*Ar}), 129.8 (C^{Ar}), 129.5 (C^{Ar}), 127.6 (C^{*Ar}), 126.5 (C^{Ar}), 123.9 (C^{Ar}), 120.4 (C^{Ar}), 107.3 (C^{7}), 101.1 (C^{7a}), 68.7 (C^{β}), 63.1 (C^{α}), 51.2 ($O\underline{C}H_3$), 18.9 ($\underline{C}H_3^{Ar}$), 17.0 ($\underline{C}H_3^{7a}$), 15.6 ($\underline{C}H_3^{7}$).

(3*R*,7*R*,7a*S*)-7-methoxy-7,7a-dimethyl-3-(2-methylquinolin-4-yl)tetrahydro-5*H*-oxazolo[4,3-b]oxazol-5-one

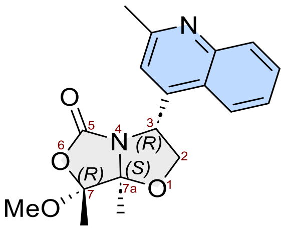

17

Following the above general procedure, the activated ester **4** was treated with 2-methylquinoline affording product **17** as a colorless oil (15.4 mg, 0.047 mmol, 47% yield). $[\alpha]^{20}_{D}$ -105.9 (c 1.0, $CHCl_3$). HRMS (ESI) m/z: $[M + Na]^+$ Calcd for $C_{18}H_{20}N_2NaO_4$: 351.1315; Found 351.1305. ^{1}H NMR ($CDCl_3$, 300 MHz): δ 8.18, 7.96, 7.69, 7.50, 7.06 (5H, H^{Ar}), 5.42 (dd, *J* = 8.5 Hz, 5.6, 1H, C$\underline{H}^{\alpha}$), 4.84 (dd, *J* = 8.7 Hz, 5.7 1H, 1C$\underline{H}_2^{\beta}$), 4.59 (t, *J* = 8.6 Hz, 1H, 1C$\underline{H}_2^{\beta}$), 4.08 (s, 3H, C$\underline{H}_3^{Ar}$), 3.51 (s, 3H, OC$\underline{H}_3$), 1.64 (s, 3H, C$\underline{H}_3^{7}$), 1.19 (s, 3H, C$\underline{H}_3^{7a}$). ^{13}C{^{1}H} NMR ($CDCl_3$, 100 MHz): δ 163.1 (C^{*Ar}), 162.0 (CO), 160.7 (C^{*Ar}), 148.6 (C^{*Ar}), 130.2 (C^{Ar}), 128.7 (C^{Ar}), 125.8 (C^{Ar}), 122.0 (C^{Ar}), 120.8 (C^{*Ar}), 107.4 (C^7), 101.1 (C^{7a}), 98.4 (C^{Ar}), 68.9 (C^{β}), 63.5 (C^{α}), 56.1 (C$\underline{H}_3^{Ar}$), 51.2 (O$\underline{C}$H$_3$), 17.1 ($\underline{C}H_3^{7a}$), 15.6 ($\underline{C}H_3^{7}$).

(3*R*,7*R*,7a*S*)-7-methoxy-3-(4-methoxyquinolin-2-yl)-7,7a-dimethyltetrahydro-5*H*-oxazolo[4,3-b]oxazol-5-one

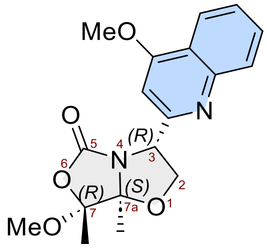

18

Following the above general procedure, the activated ester **4** was treated with 4-methoxyquinoline affording product **18** as a colorless oil (19.3 mg, 0.056 mmol, 56% yield). $[\alpha]^{20}_{D}$ -75.2 (c 1.0, $CHCl_3$). HRMS (ESI) m/z: $[M + Na]^+$ Calcd for $C_{18}H_{20}N_2NaO_5$ 367.1264; Found 367.1256. ^{1}H NMR ($CDCl_3$, 300 MHz): δ 8.32, 7.80, 7.68, 7.58, 7.52 (5H, H^{Ar}), 5.79 (dd, *J* = 8.6, 7.0 Hz, 1H, C$\underline{H}^{\alpha}$), 4.90 (t, *J* = 8.8 Hz, 1H, 1C$\underline{H}_2^{\beta}$), 3.81 (dd, *J* = 8.7, 7.0 Hz, 1H, 1C$\underline{H}_2^{\beta}$), 3.56 (s, 3H, OC$\underline{H}_3$), 2.83 (s, 3H, OC$\underline{H}_3^{Ar}$), 1.65 (s, 3H, C$\underline{H}_3^{7}$), 1.36 (s, 3H, C$\underline{H}_3^{7a}$). ^{13}C{^{1}H} NMR ($CDCl_3$, 100 MHz): δ 165.1 (C^{*Ar}), 161.6 (CO), 147.4 (C^{*Ar}), 146.5 (C^{*Ar}), 134.1 (C^{Ar}), 129.1 (C^{Ar}), 123.8 (C^{*Ar}), 123.3 (C^{Ar}), 122.6 (C^{Ar}), 118.8 (C^{Ar}), 107.2 (C^7), 101.6 (C^{7a}), 71.2 (C^{β}), 61.0 (C^{α}), 51.3 (O$\underline{C}$H$_3$), 25.1 (O$\underline{C}H_3^{Ar}$), 16.9 ($\underline{C}H_3^{7a}$), 15.5 ($\underline{C}H_3^{7}$).

(3*R*,7*R*,7a*S*)-7-methoxy-7,7a-dimethyl-3-(quinolin-4-yl)tetrahydro-5*H*-oxazolo[4,3-b]oxazol-5-one

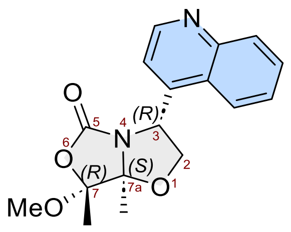

19a

Following the above general procedure, the activated ester **4** was treated with quinoline affording product **19a** as a colorless oil (14.5 mg, 0.046 mmol, 46% yield). $[\alpha]^{20}_{D}$ -108.6 (c 1.0, $CHCl_3$). HRMS (ESI) m/z: $[M + Na]^+$ Calcd for $C_{17}H_{18}N_2NaO_4$: 337.1159; Found 337.1147. ^1H NMR ($CDCl_3$, 300 MHz): δ 8.12, 8.05, 7.83, 7.73, 7.56 (6H, H^{Ar}), 5.48 (dd, *J* = 8.5, 5.6 Hz, 1H, $C\underline{H}^{\alpha}$), 4.83 (dd, *J* = 8.7, 5.6 Hz, 1H, $1C\underline{H}_2^{\beta}$), 4.63 (t, *J* = 8.6 Hz, 1H, $1C\underline{H}_2^{\beta}$), 3.51 (s, 3H, $OC\underline{H}_3$), 1.64 (s, 3H, $C\underline{H}_3^{7}$), 1.16 (s, 3H, $C\underline{H}_3^{7a}$). $^{13}C\{^1H\}$ NMR ($CDCl_3$, 100 MHz): δ 161.9 (CO), 159.5 (C^{*Ar}), 147.6 (C^{*Ar}), 137.0 (C^{*Ar}), 129.9 (C^{Ar}), 129.3 (C^{Ar}), 127.8 (C^{Ar}), 127.5 (C^{*Ar}), 126.8 (C^{Ar}), 119.7 (C^{Ar}), 107.3 (C^{7}), 101.1 (C^{7a}), 69.0 (C^{β}), 63.2 (C^{α}), 51.2 ($O\underline{C}H_3$), 17.0 ($\underline{C}H_3^{7a}$), 15.6 ($\underline{C}H_3^{7}$).

(3*R*,7*R*,7a*S*)-7-methoxy-7,7a-dimethyl-3-(quinolin-2-yl)tetrahydro-5*H*-oxazolo[4,3-b]oxazol-5-one

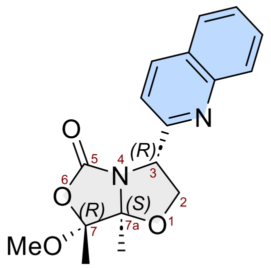

19b

Following the above general procedure, the activated ester **4** was treated with quinoline affording product **19b** as a colorless oil (13.8 mg, 0.044 mmol, 44% yield). $[\alpha]^{20}_{D}$ -124.6 (c 1.0, $CHCl_3$). HRMS (ESI) m/z: $[M + Na]^+$ Calcd for $C_{17}H_{18}N_2NaO_4$: 337.1159; Found 337.1151. ^1H NMR ($CDCl_3$, 400 MHz): δ 8.93, 8.30, 7.79, 7.66 (6H, H^{Ar}), 5.8 (t, *J* = 7.9 Hz, 1H, $C\underline{H}^{\alpha}$), 4.91 (t, *J* = 8.7, 1H, $1C\underline{H}_2^{\beta}$), 3.84 (dd, *J* = 8.6, 6.9 Hz, 1H, $1C\underline{H}_2^{\beta}$), 3.55 (s, 3H, $OC\underline{H}_3$), 1.65 (s, 3H, $C\underline{H}_3^{7}$), 1.36 (s, 3H, $C\underline{H}_3^{7a}$).$^{13}C\{^1H\}$ NMR ($CDCl_3$, 100 MHz): δ 165.0 (C^{*Ar}), 161.6 (CO), 149.9 (C^{Ar}), 147.4 (C^{*Ar}), 146.9 (C^{*Ar}), 134.1 (C^{Ar}), 130.3 (C^{Ar}), 129.9 (C^{Ar}), 125.6 (C^{*Ar}), 123.3 (C^{Ar}), 107.2 (C^{7}), 101.6 (C^{7a}), 71.1 (C^{β}), 61.0 (C^{α}), 51.3 ($O\underline{C}H_3$), 16.9 ($\underline{C}H_3^{7a}$), 15.5 ($\underline{C}H_3^{7}$).

(3*S*,7*R*,7a*S*)-3-(benzo[d]thiazol-2-yl)-7-methoxy-7,7a-dimethyltetrahydro-5*H*-oxazolo[4,3-b]oxazol-5-one

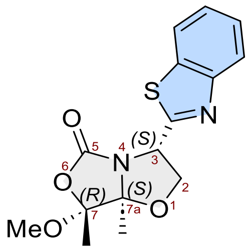

20

Following the above general procedure, the activated ester **4** was treated with benzo[d]thiazole affording product **20** as a colorless oil (13.5 mg, 0.042 mmol, 42% yield). $[\alpha]^{20}_{D}$ -168.7 (c 1.0, $CHCl_3$). HRMS (ESI) m/z: $[M + Na]^+$ Calcd for $C_{15}H_{16}N_2NaO_4S$: 343.0723; Found 343.0721. ^1H NMR ($CDCl_3$, 300 MHz): δ 7.99, 7.89, 7.50, 7.41 (4H, H^{Ar}), 5.56 (dd, *J* = 8.4, 5.7 Hz, 1H, $C\underline{H}^{\alpha}$), 4.67 (t, *J* = 8.6 Hz, 1H, $1C\underline{H}_2^{\beta}$), 4.59 (dd, *J* = 8.8, 5.7 Hz, 1H, $1C\underline{H}_2^{\beta}$), 3.51 (s, 3H, $OC\underline{H}_3$), 1.63 (s, 3H, $C\underline{H}_3^{7}$), 1.30 (s, 3H, $C\underline{H}_3^{7a}$). $^{13}C\{^1H\}$ NMR ($CDCl_3$, 100 MHz): δ 171.8 (C^{*Ar}), 161.0 (CO), 153.8 (C^{*Ar}), 135.2 (C^{*Ar}), 126.5 (C^{Ar}), 125.6 (C^{Ar}), 123.2 (C^{*Ar}), 122.0 (C^{Ar}), 107.5 (C^7), 101.6 (C^{7a}), 69.6 (C^{β}), 61.2 (C^{α}), 51.3 ($O\underline{C}H_3$), 17.9 ($\underline{C}H_3^{7a}$), 15.6 ($\underline{C}H_3^{7}$).

(3*R*,7*R*,7a*S*)-7-methoxy-7,7a-dimethyl-3-(quinoxalin-2-yl)tetrahydro-5*H*-oxazolo[4,3-b]oxazol-5-one

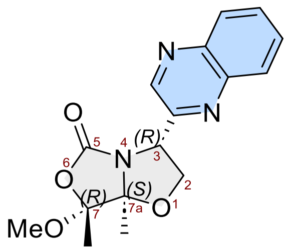

21

Following the above general procedure, the activated ester **4** was treated with quinoxaline affording product **21** as a colorless oil (12.3 mg, 0.041 mmol, 41% yield). $[\alpha]^{20}_{D}$ -129.7 (c 1.0, $CHCl_3$). HRMS (ESI) m/z: $[M + Na]^+$ Calcd for $C_{16}H_{17}N_3NaO_4$: 338.1111; Found 338.1099. ^1H NMR ($CDCl_3$, 300 MHz): δ 9.14, 8.15, 8.06, 7.80 (5H, H^{Ar}), 5.54 (dd, *J* = 8.5, 5.6 Hz, 1H, $C\underline{H}^{\alpha}$), 4.79 (dd, *J* = 8.9, 5.6 Hz, 1H, $1C\underline{H}_2^{\beta}$), 4.63 (t, *J* = 8.7 Hz, 1H, $1C\underline{H}_2^{\beta}$), 3.51 (s, 3H, $OC\underline{H}_3$), 1.65 (s, 3H, $C\underline{H}_3^{7}$), 1.18 (s, 3H, $C\underline{H}_3^{7a}$). $^{13}C\{^1H\}$ NMR ($CDCl_3$, 100 MHz): δ 161.6 (CO), 153.8 (C^{*Ar}), 144.5 (C^{Ar}), 142.2 (C^{*Ar}), 141.5 (C^{*Ar}), 130.6 (C^{Ar}), 130.2 (C^{Ar}), 129.60 (C^{Ar}), 129.3 (C^{Ar}), 107.5 (C^7), 101.4 (C^{7a}), 68.4 (C^{β}), 61.6 (C^{α}), 51.3 ($O\underline{C}H_3$), 17.0 ($\underline{C}H_3^{7a}$), 15.6 ($\underline{C}H_3^{7}$).

(3*R*,7*R*,7a*S*)-7-methoxy-7,7a-dimethyl-3-(phenanthridin-6-yl)tetrahydro-5*H*-oxazolo[4,3-b]oxazol-5-one

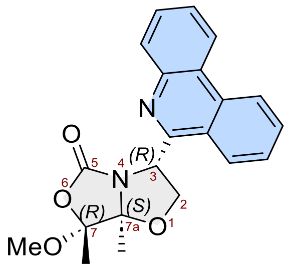

22

Following the above general procedure, the activated ester **4** was treated with phenanthridine affording product **22** as a white solid (33.5 mg, 0.092 mmol, 92% yield, mp 189-192 °C). $[\alpha]^{20}_{D}$ -167.2 (c 1.0, $CHCl_3$). HRMS (ESI) m/z: $[M + Na]^+$ Calcd for $C_{21}H_{20}N_2NaO_4$: 387.1315; Found 387.1305. ^{1}H NMR ($CDCl_3$, 300 MHz): δ 8.99, 8.65, 8.57, 8.17, 7.87, 7.74 (8H, H^{Ar}), 6.10 (dd, J = 8.0, 4.6 Hz, 1H, $C\underline{H}^{\alpha}$), 5.69 (dd, J = 8.2, 4.6 Hz, 1H, $1C\underline{H_2}^{\beta}$), 4.34 (t, J = 8.0 Hz, 1H, $1C\underline{H_2}^{\beta}$), 4.34 (s, 3H, $OC\underline{H_3}$), 1.66 (s, 3H, $C\underline{H_3}^{7}$), 1.23 (s, 3H, $C\underline{H_3}^{7a}$). $^{13}C\{^1H\}$ NMR ($CDCl_3$, 100 MHz): δ 162.0 (CO), 155.2 (C^{*Ar}), 142.6 (C^{*Ar}), 133.4 (C^{*Ar}), 130.9 (C^{Ar}), 130.5 (C^{Ar}), 128.8 (C^{Ar}), 128.0 (C^{Ar}), 127.7 (C^{Ar}), 126.7 (C^{Ar}), 125.5 (C^{*Ar}), 124.5 (C^{*Ar}), 122.4 (C^{Ar}), 122.1 (C^{Ar}), 107.4 (C^{7}), 101.4 (C^{7a}), 65.7 (C^{β}), 61.0 (C^{α}), 51.0 ($O\underline{C}H_3$), 17.7 ($\underline{C}H_3^{7a}$), 15.7 ($\underline{C}H_3^{7}$).

5. Experimental procedure to synthesize compounds 19a and 19c.

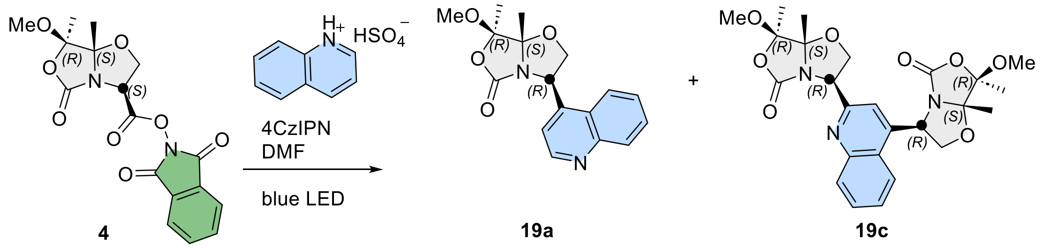

4 **19a** **19c**

A dried vial equipped with a Teflon septum and a magnetic stir bar was charged with 4-CzIPN (1 mg, 0.01 mmol, 0.01 equiv), quinoline sulfate (0.10 mmol, 1.0 equiv), the **freshly synthesized** ester **4** and vacuum was created. Then anhydrous DMF (1 mL) were added to the vial, and it was irradiated with a blue LED (30 W, λ = 450 nm) for different time and temperature. The reaction was opened to air, diluted with CH_2Cl_2, poured into a separatory funnel containing a saturated $NaHCO_3$ solution and it was checked that the pH was approximately 8. The organic layer was separated with water, dried with $MgSO_4$, filtered and concentrated under vacuo. The crude mixture was purified by column chromatography (hexane/ethyl acetate gradient from 10/0 to 8/2) on silica gel to afford desired products. Procedure based on that described in a previous work.[S2]

RAE 4 (equiv)	Temperature (ºC)	Time (h)	Yield (%)	19a/19c (ratio)
1.5	25	16	92	30:70
1.0	-10	6	41	80:20
3.5	25	16	95	0:100

(3*R*,3'*R*,7*R*,7a*S*,7'*R*,7a'*S*)-3,3'-(quinoline-2,4-diyl)bis(7-methoxy-7,7a-dimethyltetrahydro-5*H*-oxazolo[4,3-b]oxazol-5-one)

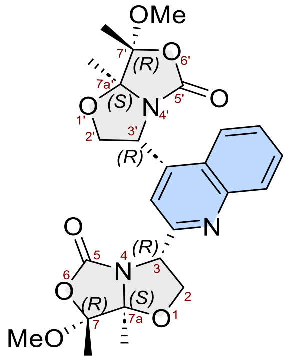

19c

mp 151-154 °C.$[\alpha]^{20}_{D}$ -248.1 (c 1.0, $CHCl_3$). HRMS (ESI) m/z: $[M + H]^+$ Calcd for $C_{25}H_{29}N_3NaO_8$: 522.1847; Found 522.1837. ^{1}H NMR ($CDCl_3$, 300 MHz): δ 8.11, 7.86, 7.76, 7.60 (5H, H^{Ar}), 5.87 (dd, *J* = 8.4, 6.6 Hz, 1H, $C\underline{H}^{\alpha}$), 5.43 (dd, *J* = 8.5, 5.8 Hz, 1H, $C\underline{H}^{\alpha'}$), 4.83 (m, 2H, $1C\underline{H}_2^{\beta}$, $1C\underline{H}_2^{\beta'}$), 4.62 (t, *J* = 8.7 Hz, 1H, $1C\underline{H}_2^{\beta'}$), 3.87 (dd, *J* = 8.7, 6.6 Hz, 1H, $1C\underline{H}_2^{\beta}$), 3.57 (s, 3H, $OC\underline{H}_3$), 3.52 (s, 3H, $OC\underline{H}_3$'), 1.66 (s, 3H, $C\underline{H}_3^{7}$), 1.64 (s, 3H, $C\underline{H}_3^{7'}$), 1.38 (s, 3H, $C\underline{H}_3^{7a}$), 1.17 (s, 3H, $C\underline{H}_3^{7a'}$). $^{13}C\{^1H\}$ NMR ($CDCl_3$, 100 MHz): δ 161.8 (CO), 161.7 (CO), 159.3 (C^{*Ar}), 147.9 (C^{*Ar}), 146.5 (C^{*Ar}), 130.5 (C^{Ar}), 130.0 (C^{Ar}), 127.4 (C^{Ar}), 124.8 (C^{*Ar}), 122.9 (C^{Ar}), 115.7 (C^{Ar}), 107.5 (C^{7}), 107.4 (C^{7}), 101.8 (C^{7a}), 101.2 (C^{7a}), 71.0 (C^{β}), 68.7 (C^{β}'), 63.3 ($C^{\alpha'}$), 61.0 (C^{α}), 51.3 ($O\underline{C}H_3$), 51.2 ($O\underline{C}H_3$'), 17.2 ($\underline{C}H_3^{7a}$), 17.0 ($\underline{C}H_3^{7a'}$), 16.0 ($\underline{C}H_3^{7}$), 15.9 ($\underline{C}H_3^{7'}$).

6. General experimental procedure to synthesize aminoalcohols 23, 24, 25, 26.

The corresponding bicyclic compound **5**, **7**, **9** or **16** (0.19 mmol) was suspended in an aqueous 6 M HCl solution (6 mL), and it was stirred at 60 ºC in an oil bath for 12 h. When the reaction finished, more water and AcOEt were added. The aqueous layer was extracted three times with water, and then the water was evaporated under vacuum.

(R)-2-amino-2-(isoquinolin-1-yl)ethan-1-ol hydrochloride

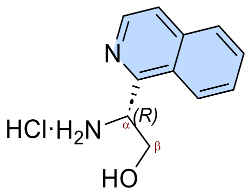

23

Product **23** was obtained from compound **5** in quantitative yield as a white solid (36 mg, 0.19 mmol). $[\alpha]^{20}_{D}$ -63.0 (c 1.0, D_2O). HRMS (ESI) m/z: $[M + H]^+$ Calcd for $C_{11}H_{13}N_2O$: 189.1022; Found 189.1023. ^1H NMR (D_2O, 400 MHz): δ 8.50, 8.21, 8.03, 7.84 (6H, H^{Ar}), 5.56 (dd, J = 6.5, 4.0 Hz, 1H, $C\underline{H}^{\alpha}$), 4.22 (dd, J = 12.5, 4.0 Hz, 1H, $C\underline{H}_2{}^{\beta}$), 4.03 (dd, J = 12.5, 6.5 Hz, 1H, $C\underline{H}_2{}^{\beta}$). $^{13}C\{^1H\}$ NMR (D_2O, 100 MHz): δ 151.8 (C^{*Ar}), 140.7 (C^{Ar}), 136.5 (C^{*Ar}), 131.3 (C^{Ar}), 128.6 (C^{Ar}), 127.8 (C^{Ar}), 125.2 (C^{*Ar}), 123.4 (C^{Ar}), 122.2 (C^{Ar}), 62.1 (C^{β}), 53.2 (C^{α}).

(1R,2R)-1-amino-1-(isoquinolin-1-yl)propan-2-ol hydrochloride

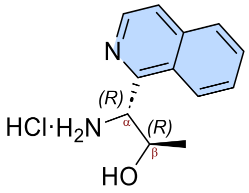

24

Product **24** was obtained from compound **7** in quantitative yield as a white solid (38 mg, 0.19 mmol). $[\alpha]^{20}_{D}$-32.5 (c 1.0, D_2O). HRMS (ESI) m/z: $[M + Na]^+$ Calcd for $C_{12}H_{14}N_2NaO$: 225.0998; Found 225.0993. ^1H NMR (D_2O, 400 MHz): δ 8.55, 8.42, 8.16, 8.03, 7.94 (6H, H^{Ar}), 5.50 (d, J = 5.4 Hz, 1H, $C\underline{H}^{\alpha}$), 4.45 (p, J = 6.4 Hz, 1H, $C\underline{H}^{\beta}$), 1.29 (d, J = 6.4 Hz, 3H, $C\underline{H}_3$). $^{13}C\{^1H\}$ NMR (D_2O, 100 MHz): δ 151.7 (C^{*Ar}), 138.0 (C^{*Ar}), 136.9 (C^{Ar}), 133.8 (C^{Ar}), 130.2 (C^{Ar}), 128.3 (C^{Ar}), 125.8 (C^{*Ar}), 124.7 (C^{Ar}), 124.4 (C^{Ar}), 67.7 (C^{β}), 55.1 (C^{α}), 19.1 ($\underline{C}H_3$).

(R)-2-amino-2-(isoquinolin-1-yl)propan-1-ol hydrochloride

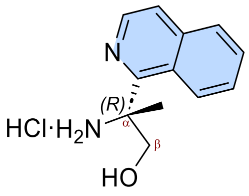

25

Product **25** was obtained from compound **9** in quantitative yield as a white solid (38 mg, 0.19 mmol). $[\alpha]^{20}_{D}$-28.9 (c 1.0, D_2O). HRMS (ESI) m/z: $[M + Na]^+$ Calcd for $C_{12}H_{14}N_2NaO$: 225.0998; Found 225.1003. ^1H NMR (D_2O, 400 MHz): δ 8.42, 8.32, 7.97, 7.82, 7.77, 7.70 (6H, H^{Ar}), 4.44 (d, J = 12.6 Hz, 1H, $C\underline{H}^{\beta}$), 4.23 (d, J = 12.6 Hz, 1H, $C\underline{H}^{\beta}$), 1.95 (s, 3H, $C\underline{H}_3$). $^{13}C\{^1H\}$ NMR (D_2O, 100 MHz): δ 154.5 (C^{*Ar}), 139.2

(C^{Ar}), 137.6 (C^{*Ar}), 130.9 (C^{Ar}), 128.6 (C^{Ar}), 128.2 (C^{Ar}), 124.7 (C^{*Ar}), 124.0 (C^{Ar}), 122.8 (C^{Ar}), 65.7 (C^{β}), 63.5 (C^{α}), 21.0 ($\underline{C}H_3$).

(*R*)-2-amino-2-(4-methylquinolin-2-yl)ethan-1-ol hydrochloride

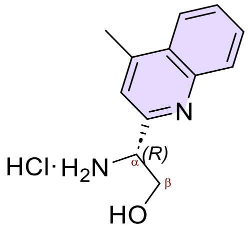

26

Product **26** was obtained from compound **16** in quantitative yield as a white solid (38 mg, 0.19 mmol). $[\alpha]^{20}_{D}$ -12.4 (c 1.0, D_2O). HRMS (ESI) m/z: $[M + Na]^+$ Calcd for $C_{12}H_{14}N_2NaO$: 225.0988; Found 225.0988. ^{1}H NMR (D_2O, 400 MHz): δ 8.38, 8.21, 8.12, 7.98, 7.94 (5H, H^{Ar}), 5.12 (t, *J* = 4.5 Hz, 1H, $C\underline{H}^{\alpha}$), 4.27 (dd, *J* = 12.4, 4.6 Hz, 1H, $C\underline{H}_2^{\beta}$), 4.19 (dd, *J* = 12.4, 5.3 Hz, 1H, $C\underline{H}_2^{\beta}$), 3.3 (s, 3H, $C\underline{H}_3^{Ar}$). $^{13}C\{^{1}H\}$ NMR (D_2O, 100 MHz): δ 160.0 (C^{*Ar}), 149.4 (C^{*Ar}), 138.5. (C^{*Ar}), 135.1 (C^{Ar}), 129.9 (C^{Ar}), 128.2 (C^{*Ar}), 125.6 (C^{Ar}), 121.6 (C^{Ar}), 120.2 (C^{Ar}), 61.2 (C^{β}), 54.1 (C^{α}), 19.7 ($\underline{C}H_3^{Ar}$).

7. Scale up of the synthetic procedure to obtain aminoalcohol 23 from Boc-L-Ser-OMe 1

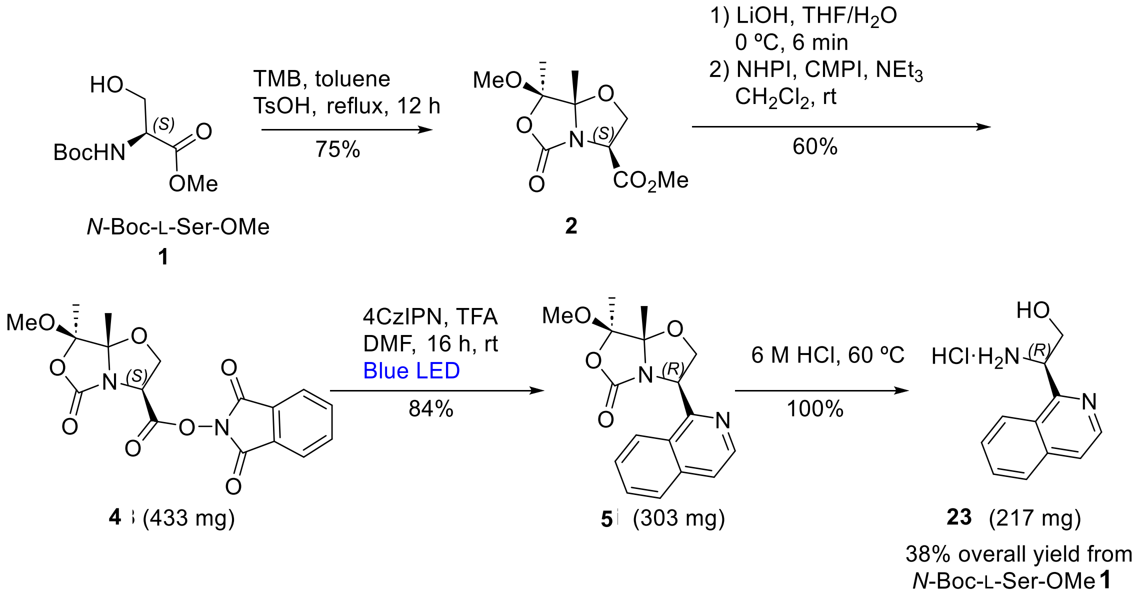

Scheme S2

Starting from 560 mg of *N*-Boc-L-Ser-OMe **1** and 2,2,3,3-tetramehoxybutane (TMB), the corresponding bicyclic *N,O*-acetal **2** was obtained in a 75% yield (470 mg). Then, it was hydrolyzed with LiOH to give acid **3**, which was coupled to *N*-hydroxyphthalimide (NHPI) with 2-chloro-1-

methylpyridinium iodide (CMPI) to obtain RAE **4** in a 60% yield (433 mg). Then, the photoinduced Minisci reaction involving the use of 4CzIPN and stoichiometric amounts of TFA gave compound **5** in an 84% yield (303 mg). Finally, it was hydrolyzed with aqueous 6 M HCl at 60 ºC to obtain chiral (*R*)-2-amino-2-(isoquinolin-1-yl)ethan-1-ol hydrochloride **23** in a quantitative yield (217 mg). In summary, chiral aminoalcohol **23** was obtained in five steps from *N*-Boc-L-Ser-OMe **1** with a 38% overall yield.

Scale up of the key photoredox Minisci reaction of activated ester 4 and isoquinoline:

A dried vial equipped with a Teflon septum and a magnetic stir bar was charged with 4-CzIPN (15.4 mg, 0.012 mmol, 0.01 equiv), isoquinoline (99 µL, 0.10 mmol, 1 equiv), TFA (77 µL, 0.12 mmol, 1.2 equiv) and vacuum was created. Then anhydrous DMF (8 mL) was added to the vial, and it was irradiated with a blue LED (30 W, λ = 450 nm) for 16 h at room temperature. The activated ester **4** (433 mg, 1.15 mmol, 1.5 equiv) was freshly synthesized and added in four portions (0.29 mmol each) every 2 h. The reaction was opened to air, diluted with CH_2Cl_2, poured into a separatory funnel containing a saturated $NaHCO_3$ solution and it was checked that the pH was approximately 8. The organic layer was separated with water, dried with $MgSO_4$, filtered and concentrated under vacuo. The crude mixture was purified by column chromatography (hexane/ethyl acetate gradient from 10:0 to 8:2) on silica gel affording product **5** as a white solid (303 mg, 0.964 mmol, 84% yield).

8. Radical trapping test with TEMPO

(3*R*,7*R*,7a*S*)-7-Methoxy-7,7a-dimethyl-3-((2,2,6,6-tetramethylpiperidin-1-yl)oxy)tetrahydro-5*H*-oxazolo[4,3-b]oxazol-5-one

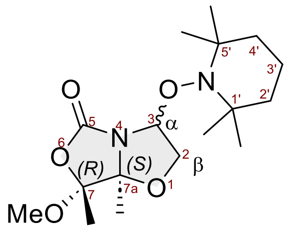

27

Following the above general procedure (section 6), the activated ester **4** was treated with 2,2,6,6-tetramethylpiperidinyloxy (TEMPO), which is a known radical trap, instead of the corresponding N-heteroarene. The crude mixture was analyzed by HRMS (ESI) and ^{1}H NMR and product **27** was detected. It could not be completely characterized by NMR since the compound decomposes in a short time in solution, because of this, we were unable to register the ^{13}C NMR spectrum.

$[\alpha]^{20}_D$ -162.4 (c 1.0, $CHCl_3$) HRMS (ESI) m/z: $[M + H]^+$ Calcd for $C_{17}H_{31}N_2O_5$: 343.2155; Found 343.2227. ^{1}H NMR ($CDCl_3$, 300 MHz): δ 5.69 (dd, *J* = 6.9, 4.5 Hz, 1H, C$\underline{H}^{\alpha}$), 4.32 (dd, *J* = 9.5, 6.9 Hz, 1H, 1C$\underline{H}_2^{\beta}$), 3.88 (dd, *J* = 9.6, 4.5 Hz, 1H, 1C$\underline{H}_2^{\beta}$) 3.43 (s, 3H, OC$\underline{H}_3$), 1.52 (s, 3H, C$\underline{H}_3^{3}$), 1.48 (s, 3H, C$\underline{H}_3^{7}$), 1.28 (s, 6H, 1C$\underline{H}_3^{1'}$, 1C$\underline{H}_3^{5'}$), 1.24 (s, 6H, 1C$\underline{H}_3^{1'}$, 1C$\underline{H}_3^{5'}$), 1.06 (m, 6H, C$\underline{H}_2^{2'}$-C$\underline{H}_2^{3'}$-C$\underline{H}_2^{4'}$).

9. X-Ray analysis

Details of the X-ray analyses are summarized in **Table S2**. Compounds **5** (**CCDC 2400619**) and **22** (**CCDC 2475248**) were dissolved in dichloromethane and n-hexane was added carefully creating an interphase in each of them. The colorless crystal block was obtained after 7 days by slow diffusion at 25 °C. The formed crystals were analyzed by X-ray diffraction. The diffraction data were collected using graphite-monochromatic Mo-K_{α} radiation with a Bruker APEX-II diffractometer at a temperature of 140 K using the APEX3 software. The absorption correction was performed using MULTI-SCAN.[S5] The structures were solved with the WINGX program suite[S6] and refined by full-matrix least squares with SHELXL.[S7] Hydrogen atoms were located by mixed methods (electron-density maps and theoretical positions).

a)

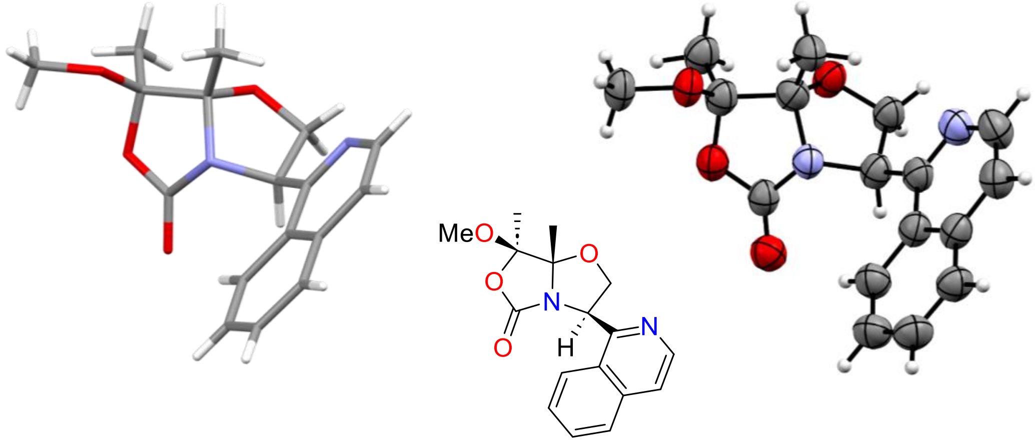

b)

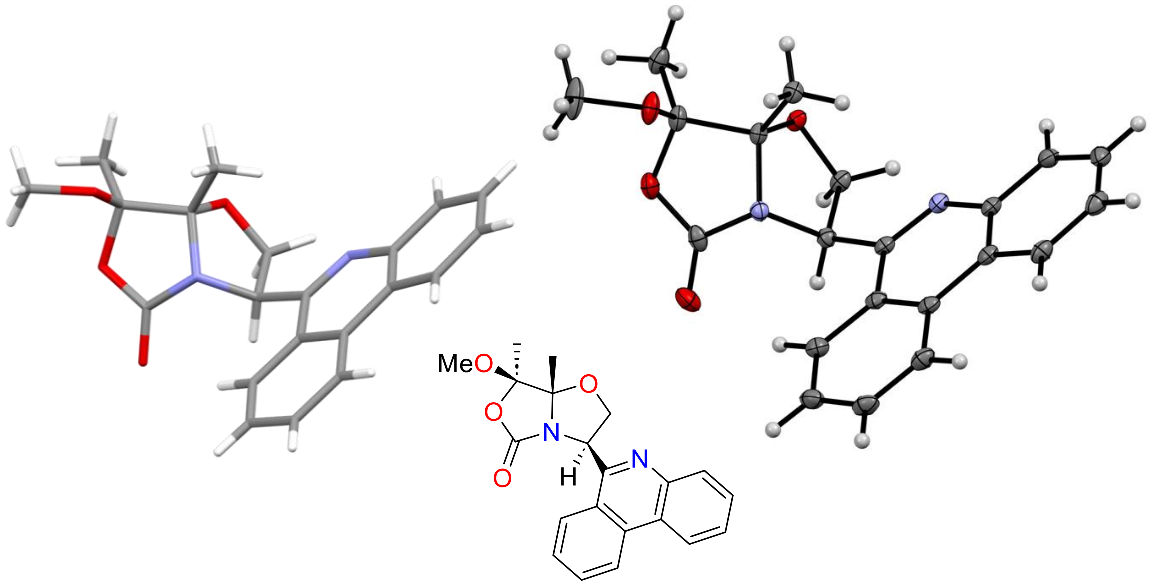

Figure S2. Sticks, 2D-ChemDraw and ORTEP3 diagram of compound **5** (a) and **22** (b) obtained by X-ray diffraction analysis showing thermal ellipsoids at the 50% probability level.

Table S2. Crystal data and structure refinement for **5** and **22**.

Empirical formula	C17 H18 N2 O4	C 21 H20 N2 O4
Formula weight	314.33	364.39
Temperature	140(2) K	100(2) K
Wavelength	0.71073 Å	0.71073 Å
Crystal system	Orthorhombic	Orthorhombic
Space group	P b c a	$P2_12_12_1$
Unit cell dimensions	a = 14.9822(12) Å a= 90°<br>b = 9.4865(7) Å b= 90°<br>c = 21.5261(16) Å g = 90°	a = 5.7181(2)Å a= 90°<br>b = 9.0851(4) b= 90°<br>c = 34.1667(16)Å g = 90°
Volume	3059.5(4) Å^3	1774.94(13) Å^3
Z	8	4
Density (calculated)	1.365 Mg/m^3	1.364 Mg/m^3
Absorption coefficient	0.098 mm^{-1}	0.095 mm^{-1}
F(000)	1328	768
Crystal size	0.230 x 0.220 x 0.080 mm^3	0.240 x 0.090 x 0.080 mm^3
Theta range for data collection	2.330 to 27.936°.	4.64 to 56.564°.
Index ranges	-19<=h<=19, -12<=k<=12, -28<=l<=28	-7<=h<=6, -12<=k<=12, -45<=l<=45
Reflections collected	84423	39167
Independent reflections	3662 [R(int) = 0.1285]	4412 [R(int) = 0.0446]
Completeness to theta = 25.242°	100.0 %	100.0 %
Refinement method	Full-matrix least-squares on F^2	Full-matrix least-squares on F^2
Data / restraints / parameters	3662 / 0 / 210	4412/ 0 / 322
Goodness-of-fit on F^2	1.553	1.042
Final R indices [I>2sigma(I)]	R1 = 0.1409, wR2 = 0.3633	R1 = 0.0363, wR2 = 0.0924
R indices (all data)	R1 = 0.2313, wR2 = 0.4547	R1 = 0.0374, wR2 = 0.0922
Largest diff. peak and hole	1.147 and -0.585 e.Å^{-3}	0.34 and -0.24 e.Å^{-3}

10. Proposed mechanism for the Minisci-type reaction.

Building upon the proposed mechanism for this Minisci-type reaction, (Scheme S5),[S8] the photoredox Ir-catalyst has a suitable redox potential to interact with the *N*-(acyloxy)phthalimide derivative **4**, to produce an α-aminocarboxylate radical (**B**) and also generate an α-aminoalkyl radical (**C**) after decarboxylation. The generated α-aminoalkyl radical (**C**) can attack isoquinolin-2-ium (**F**) formed by the activation of isoquinoline with an acid cocatalyst (PA) to produce a radical cation (**D**). This radical cation can be further oxidized by the excited photoreductive catalyst [Ir(III)]* and then deprotonated by a counteranion to obtain the desired α-aminoalkylation product **5**, with regeneration of the photoreductive catalyst. The reaction works equally well with 4CzIPN and TFA instead of Ir-catalyst and PA. The key steps to control stereochemistry involve the formation of radical specie **C** and its transformation into radical cation **D** (see computational section).

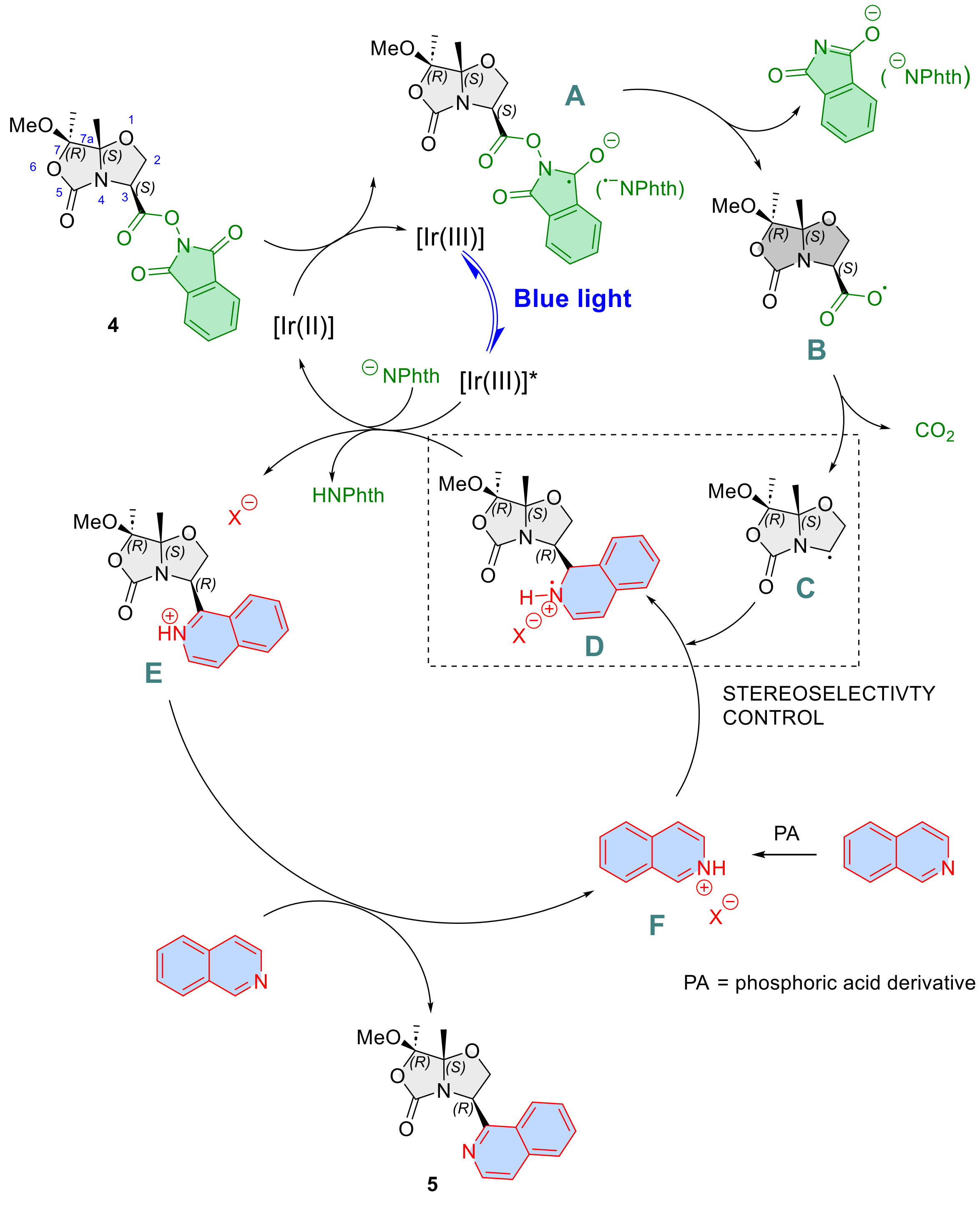

Scheme S3. Proposed mechanism for the Minisci-type reaction of active ester **4** and isoquinoline.

11. NMR spectra

^1H NMR in $CDCl_3$ (400 MHz) compound 4

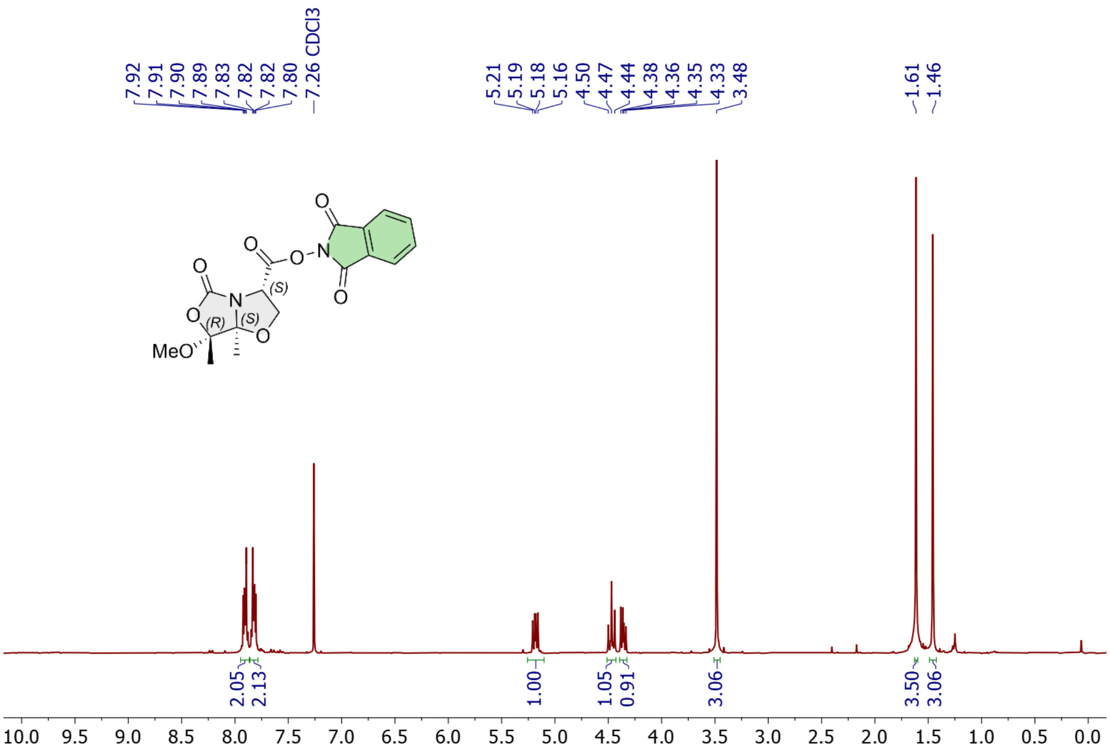

^{13}C {^1H} NMR in $CDCl_3$ (100 MHz) compound 4

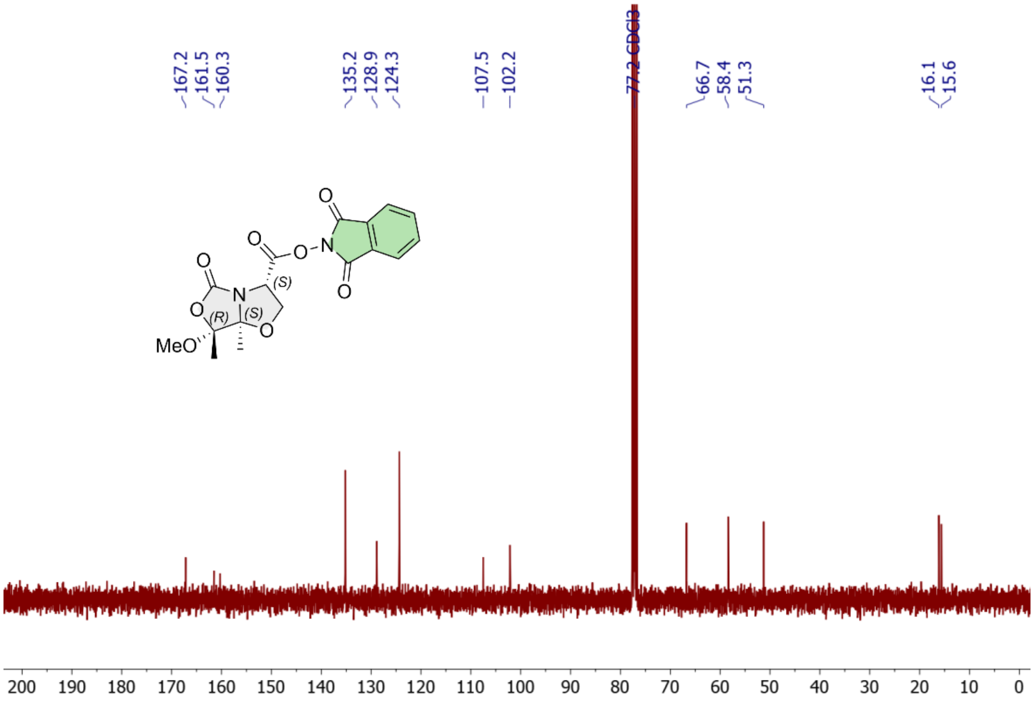

COSY in $CDCl_3$ (400 MHz) compound 4

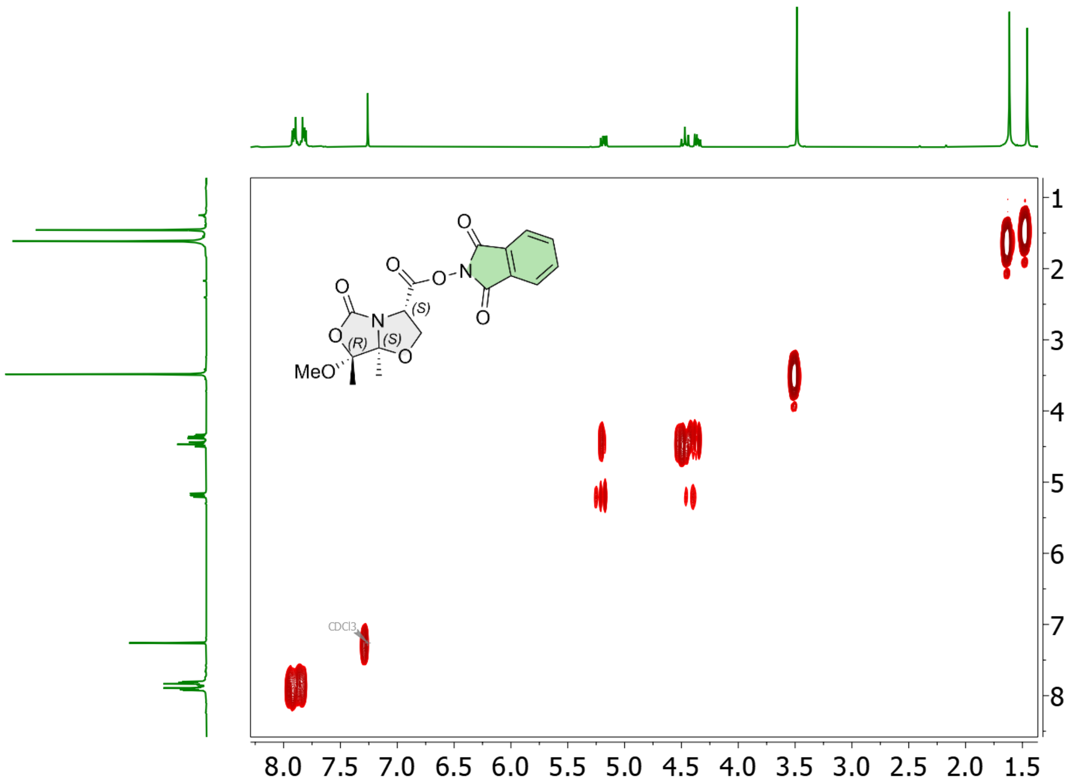

HSQC in $CDCl_3$ (400 MHz) compound 4

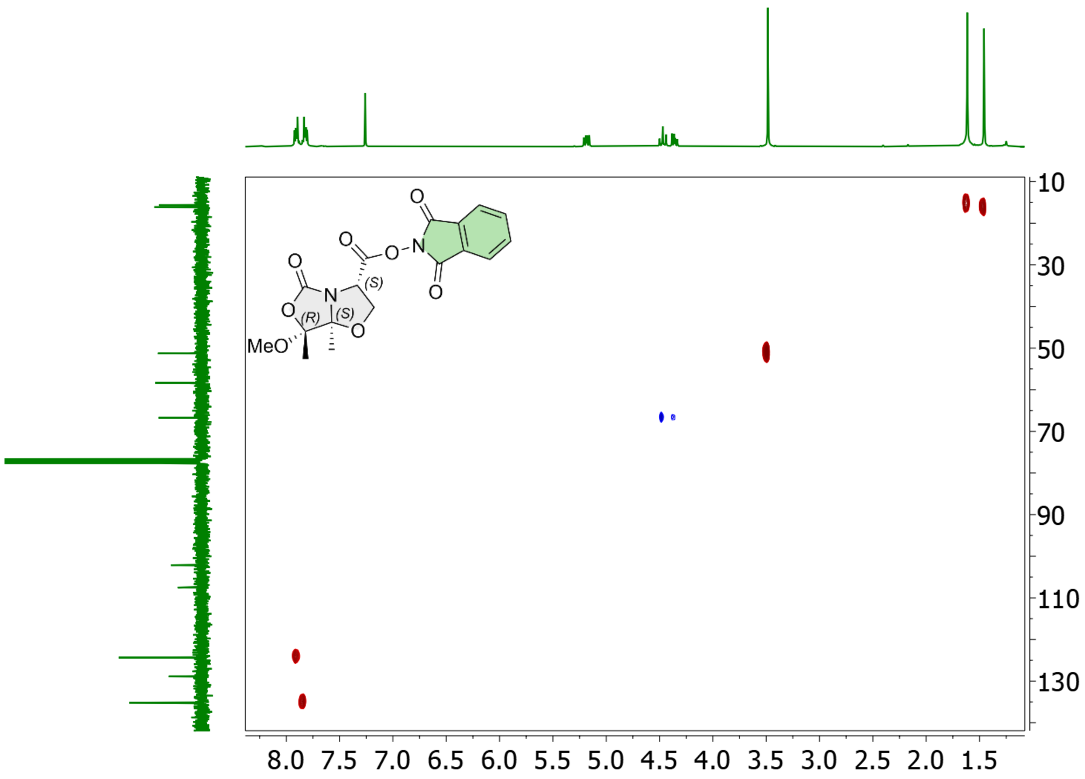

^{1}H NMR in $CDCl_3$ (400 MHz) compound 6

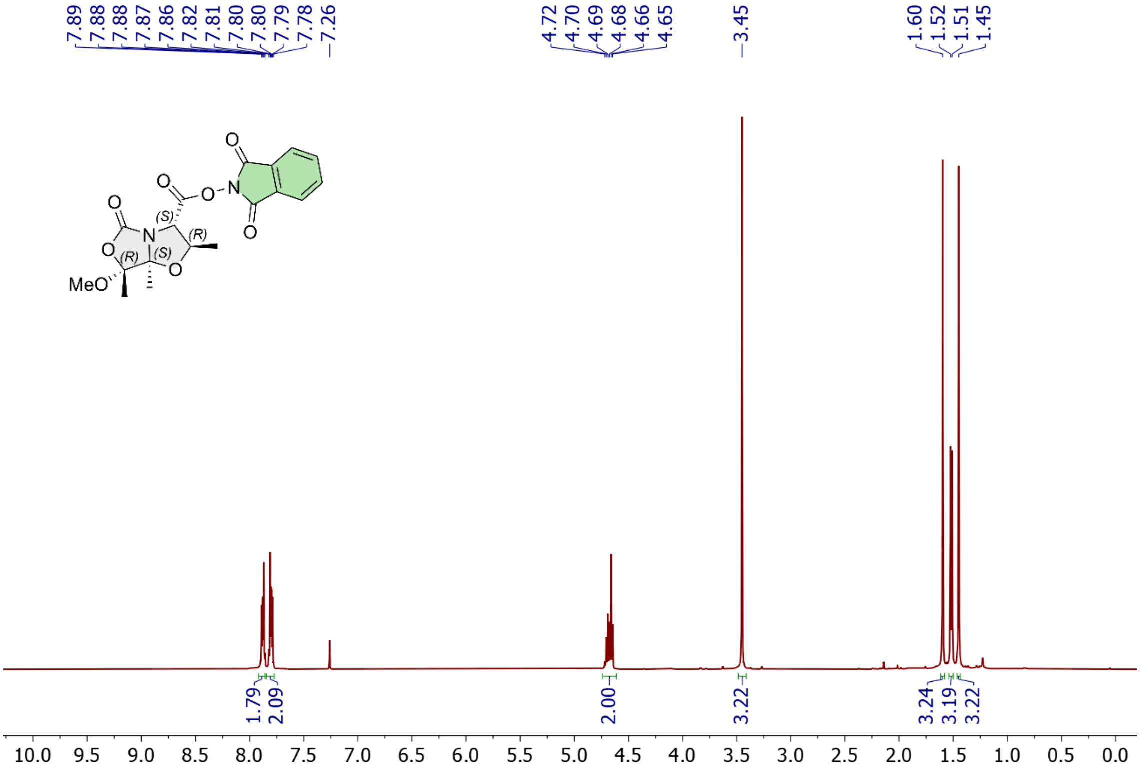

^{13}C {^{1}H} NMR in $CDCl_3$ (100 MHz) compound 6

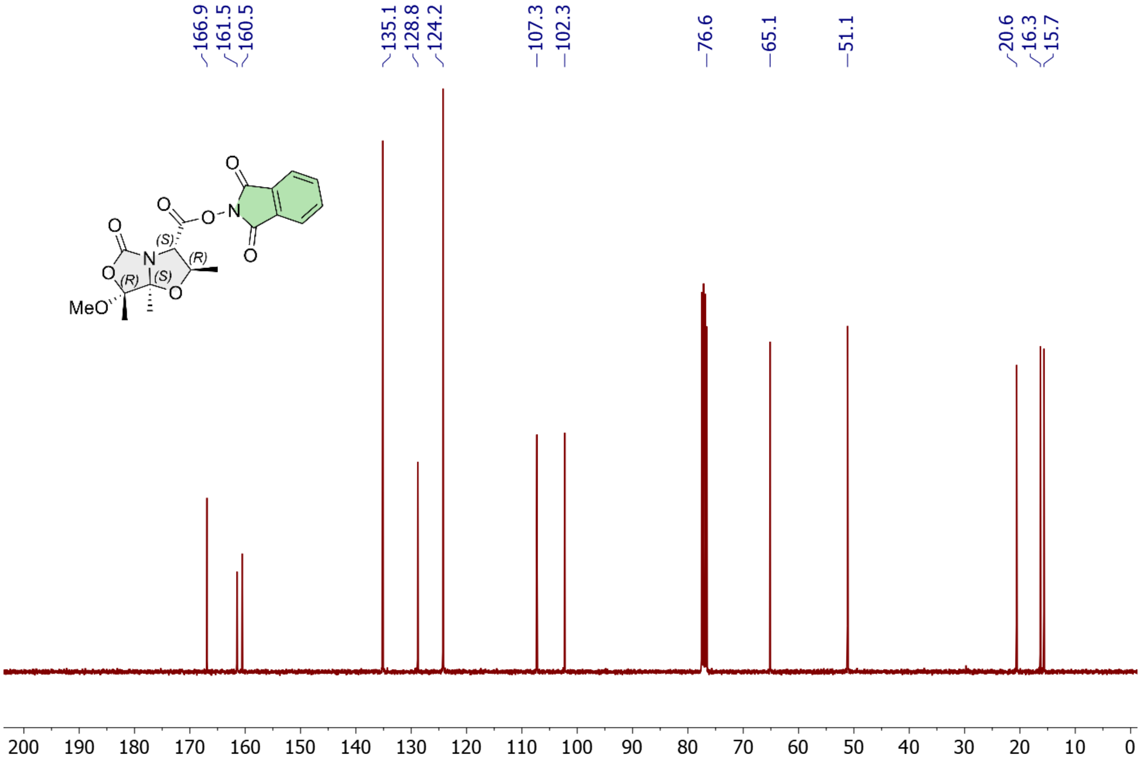

COSY in $CDCl_3$ (400 MHz) compound 6

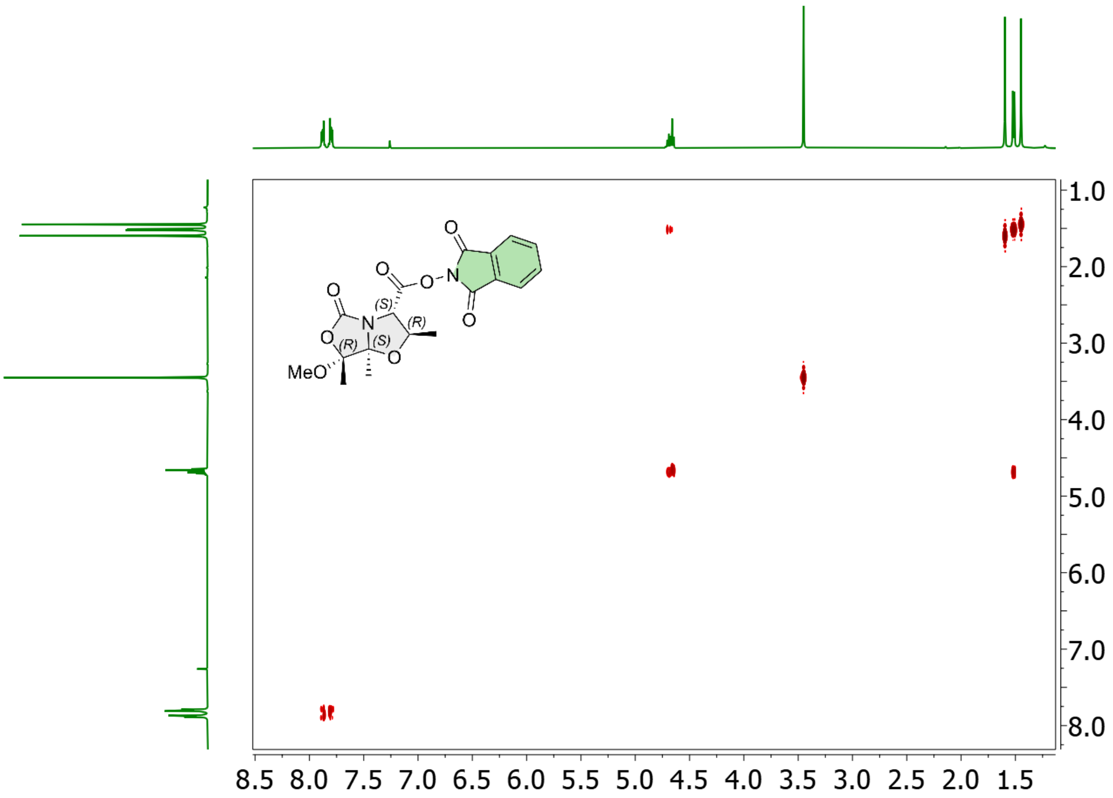

HSQC in $CDCl_3$ (400 MHz) compound 6

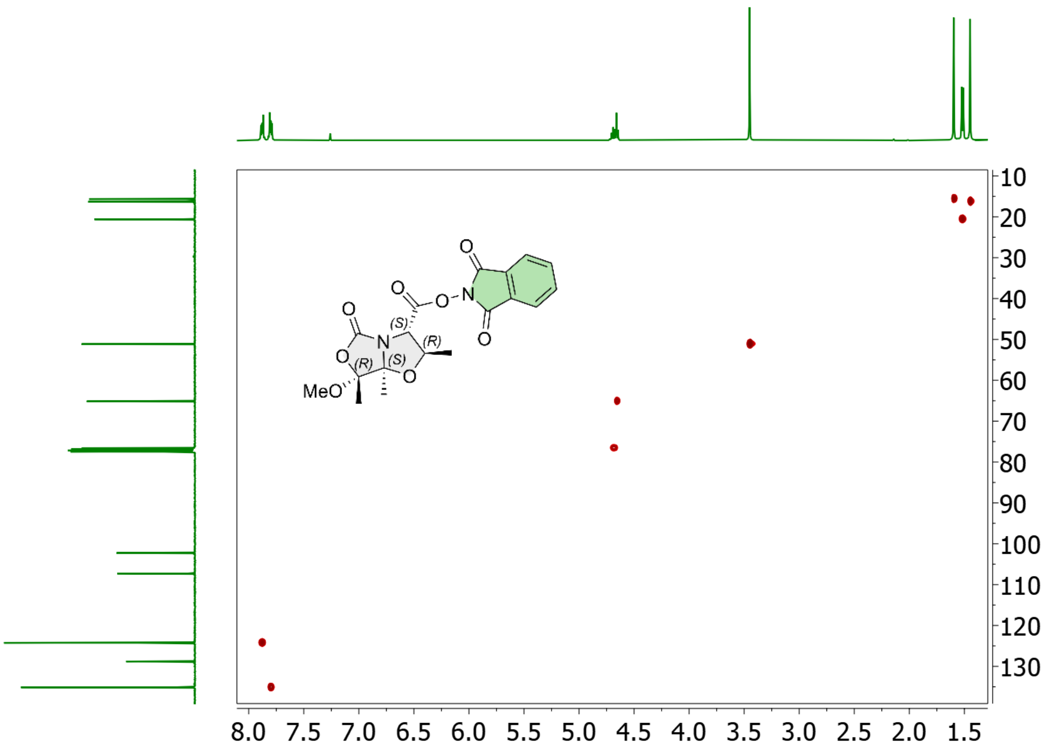

^{1}H NMR in $CDCl_3$ (400 MHz) compound 8

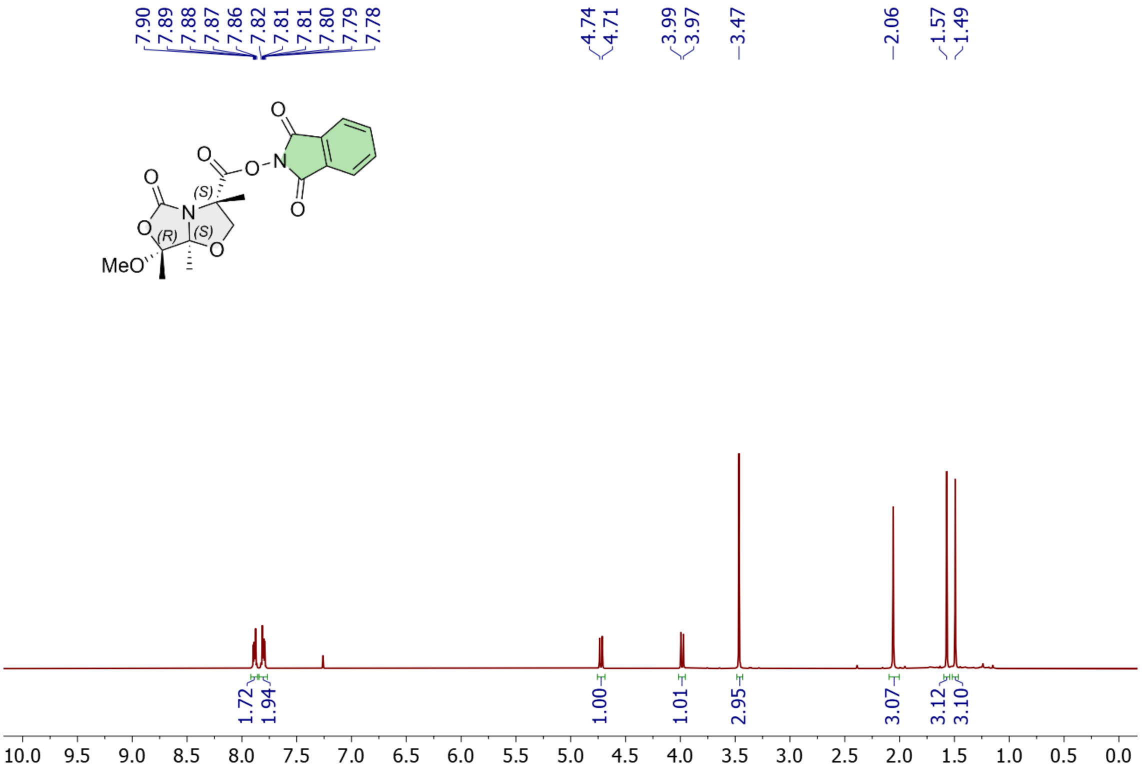

^{13}C {^{1}H} NMR in $CDCl_3$ (100 MHz) compound 8

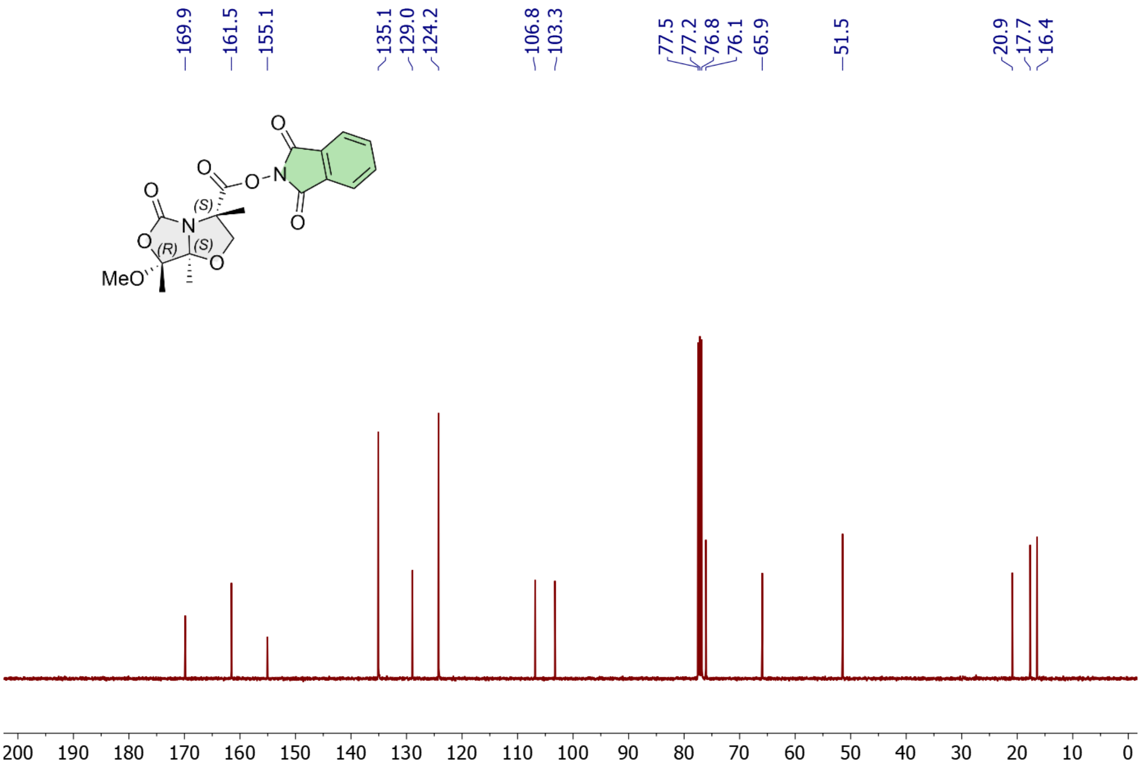

COSY in $CDCl_3$ (400 MHz) compound 8

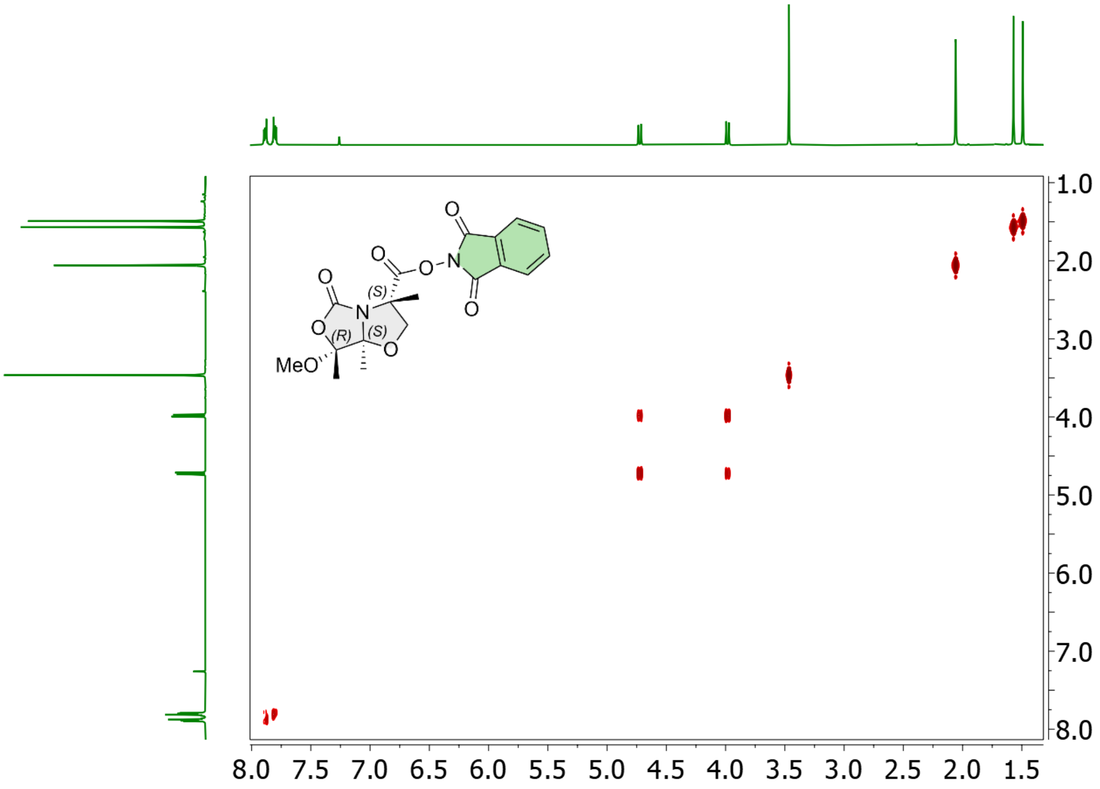

HSQC in $CDCl_3$ (400 MHz) compound 8

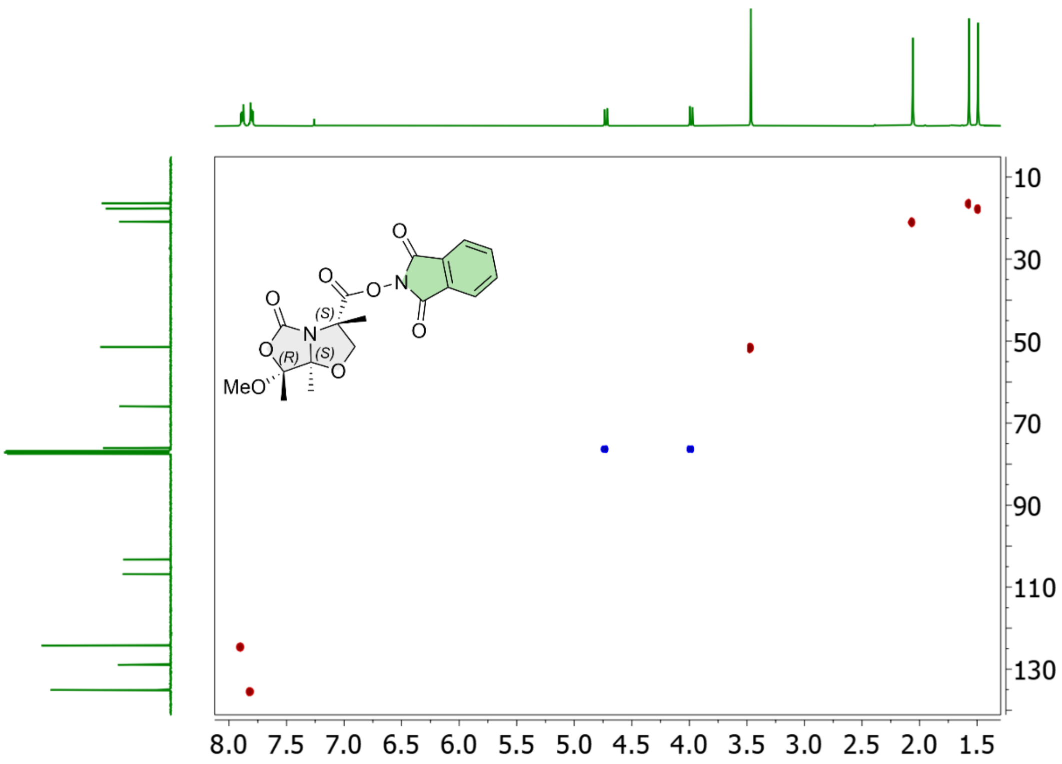

^{1}H NMR in $CDCl_3$ (400 MHz) compound 5

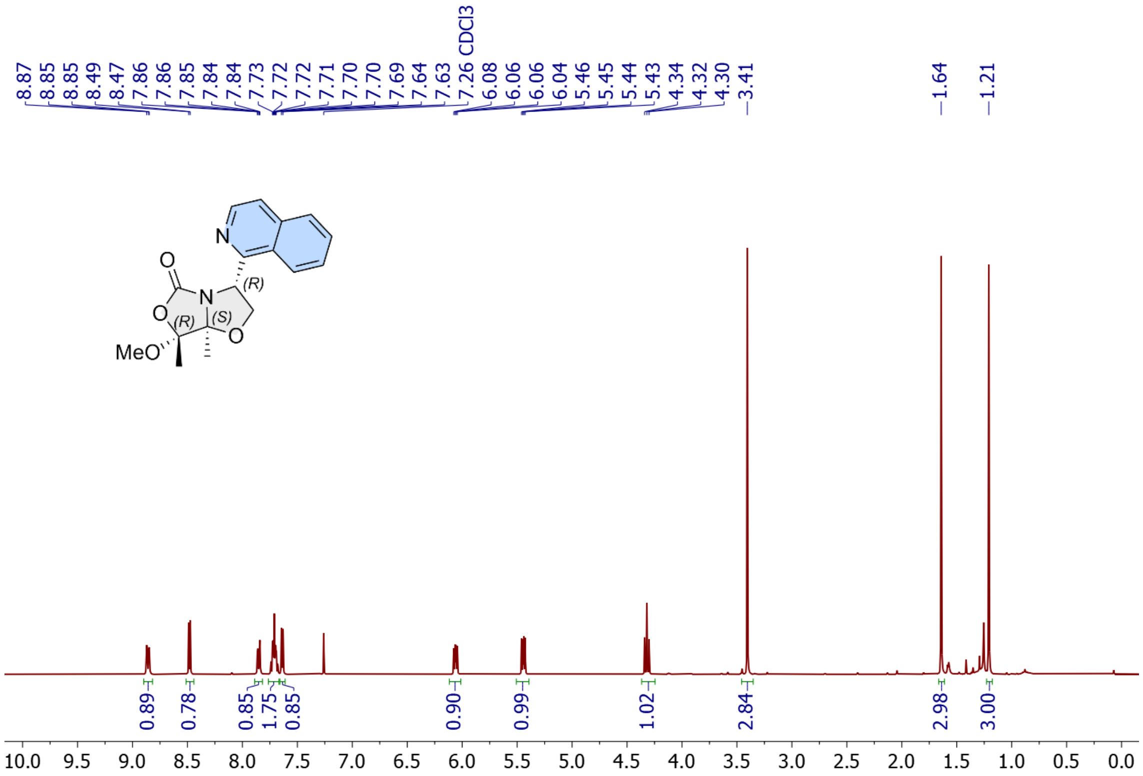

^{13}C {^{1}H} NMR in $CDCl_3$ (100 MHz) compound 5

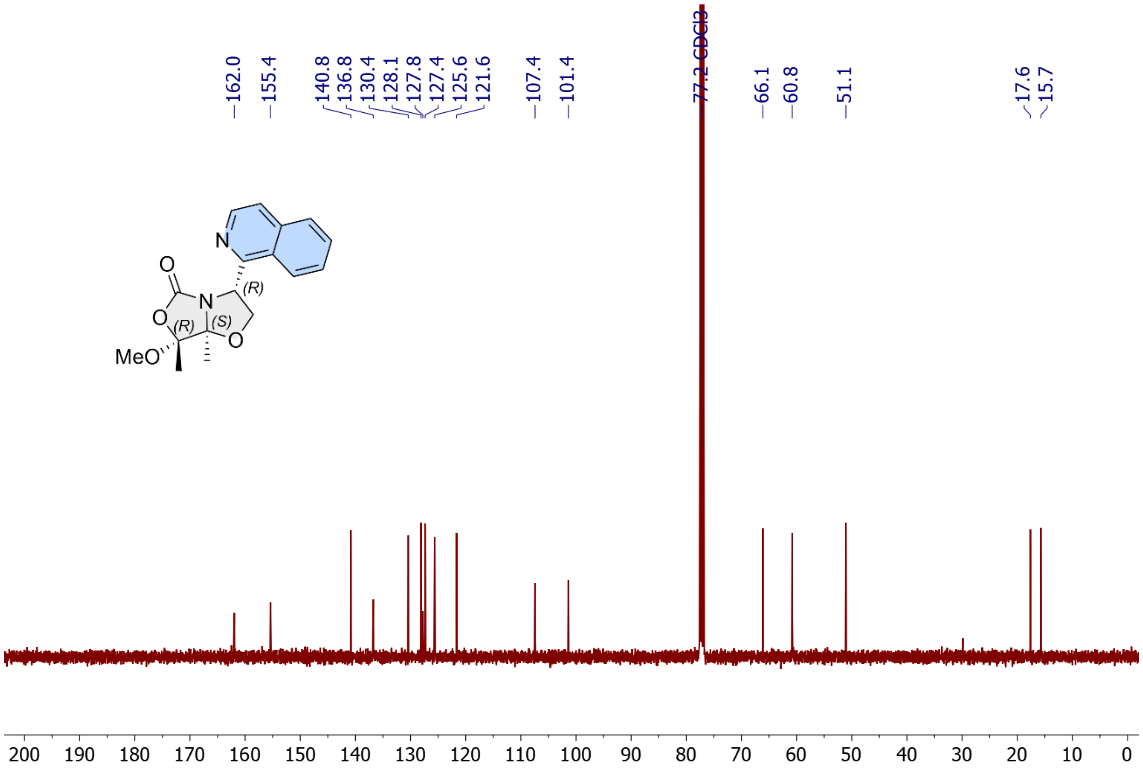

COSY in $CDCl_3$ (400 MHz) compound 5

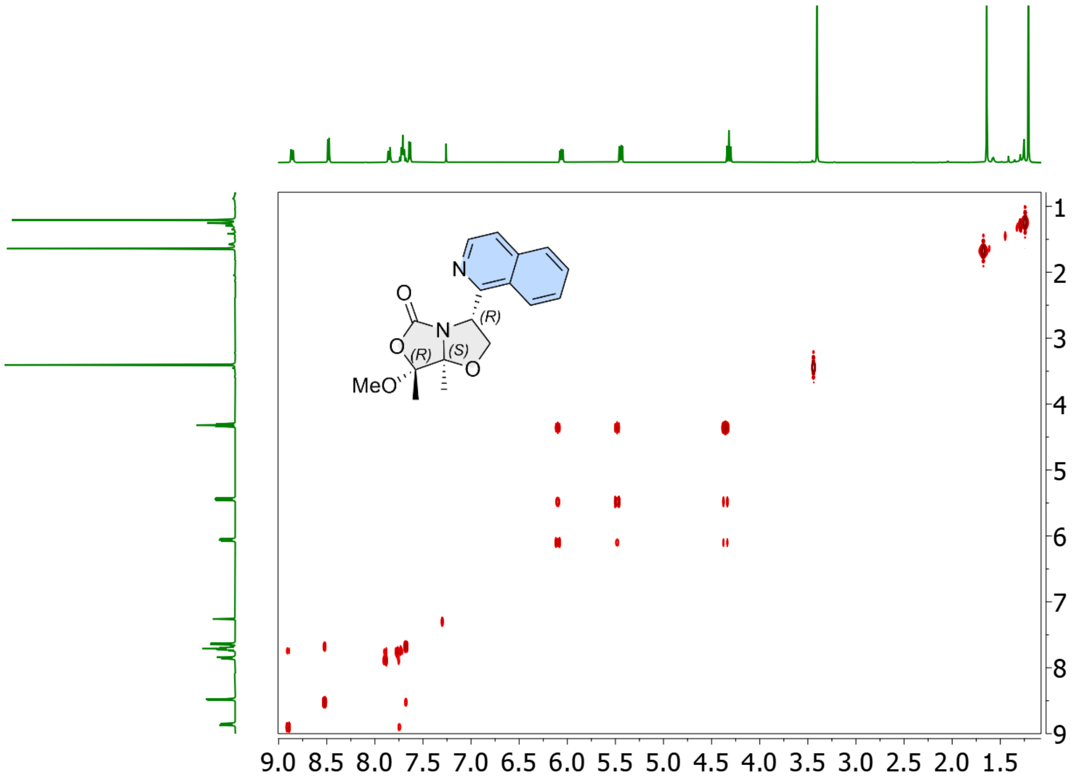

HSQC in $CDCl_3$ (400 MHz) compound 5

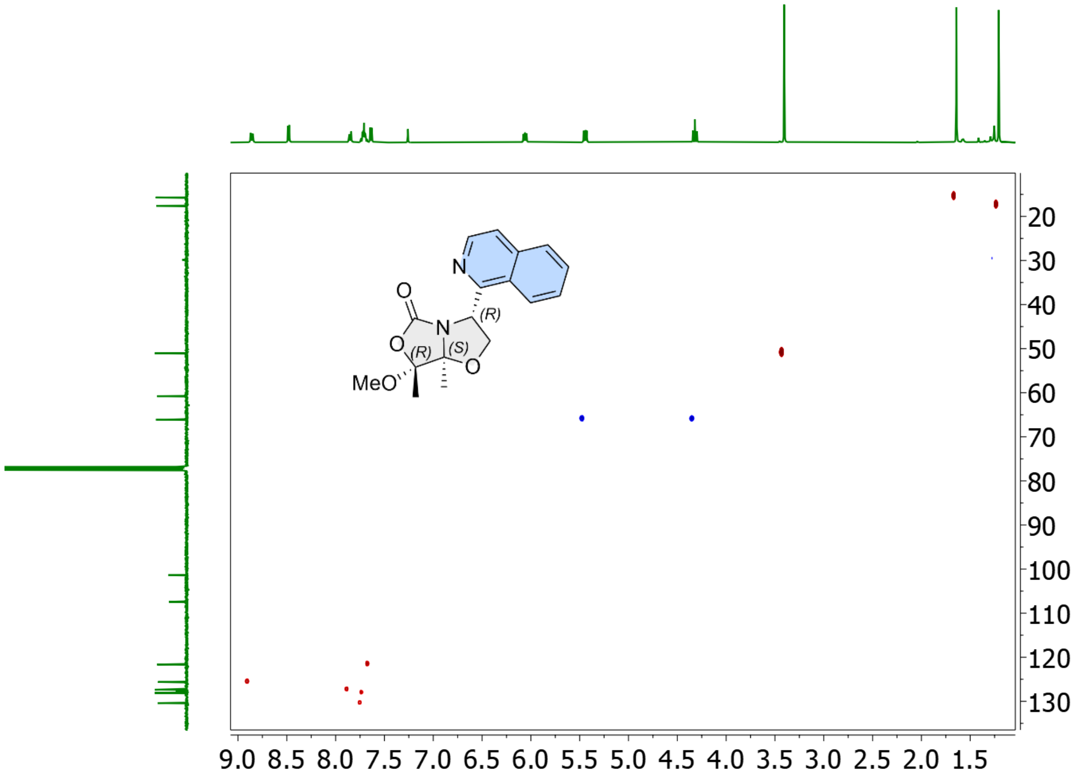

NOESY in $CDCl_3$ (400 MHz) compound 5

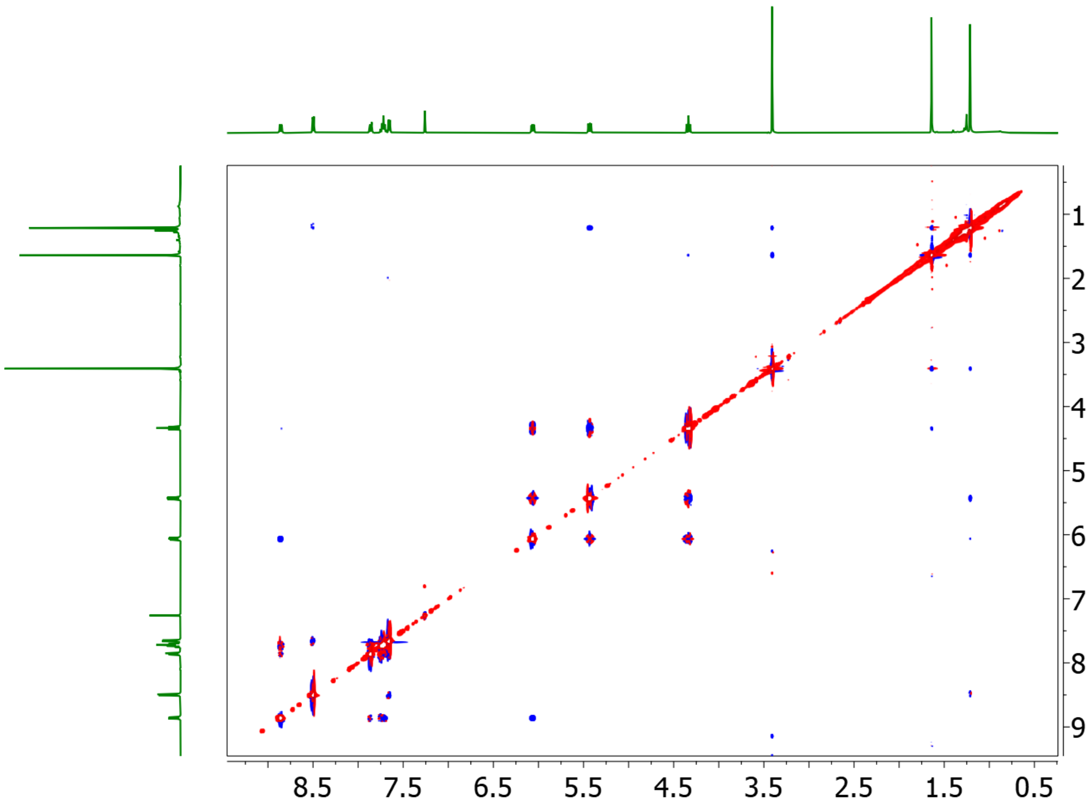

^{1}H NMR in $CDCl_3$ (400 MHz) compound 7

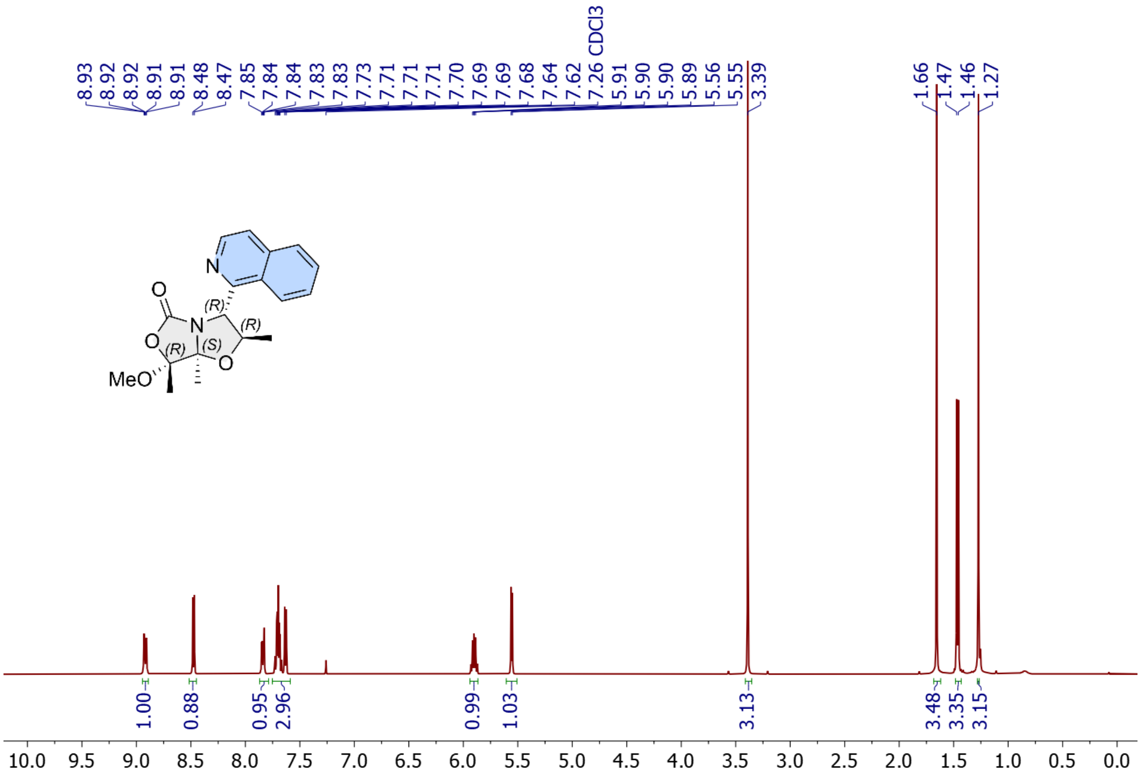

^{13}C {^{1}H} NMR in $CDCl_3$ (100 MHz) compound 7

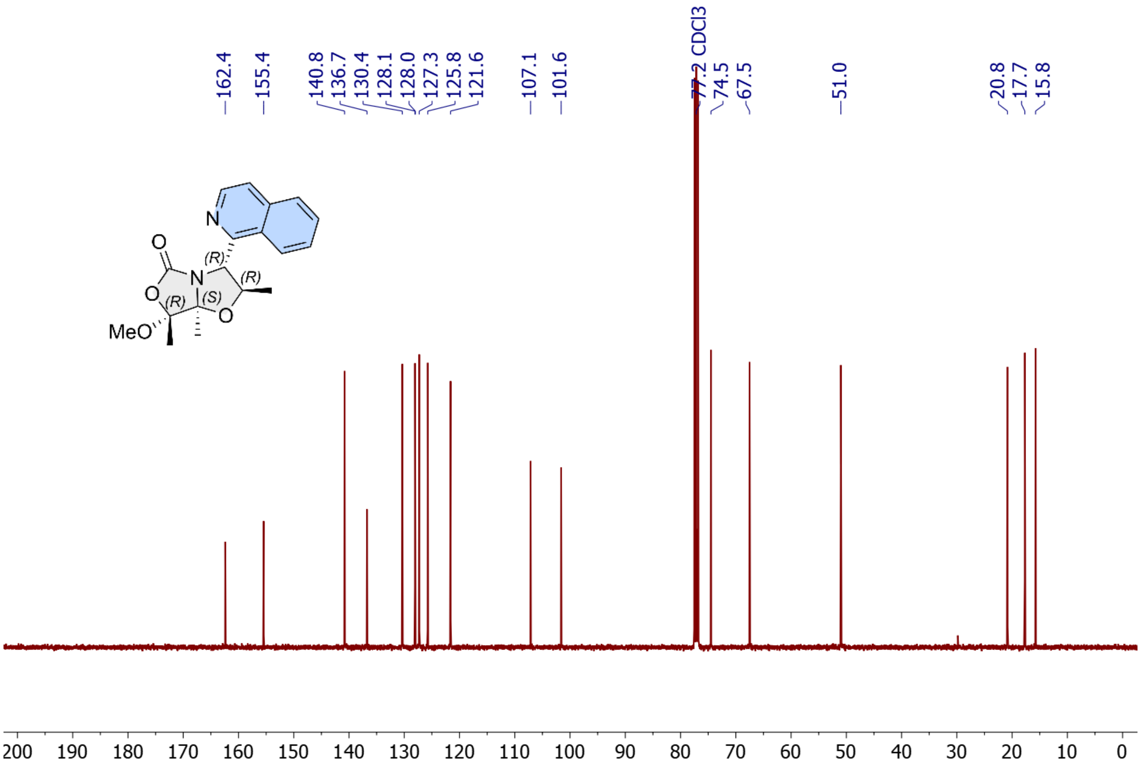

COSY in $CDCl_3$ (400 MHz) compound 7

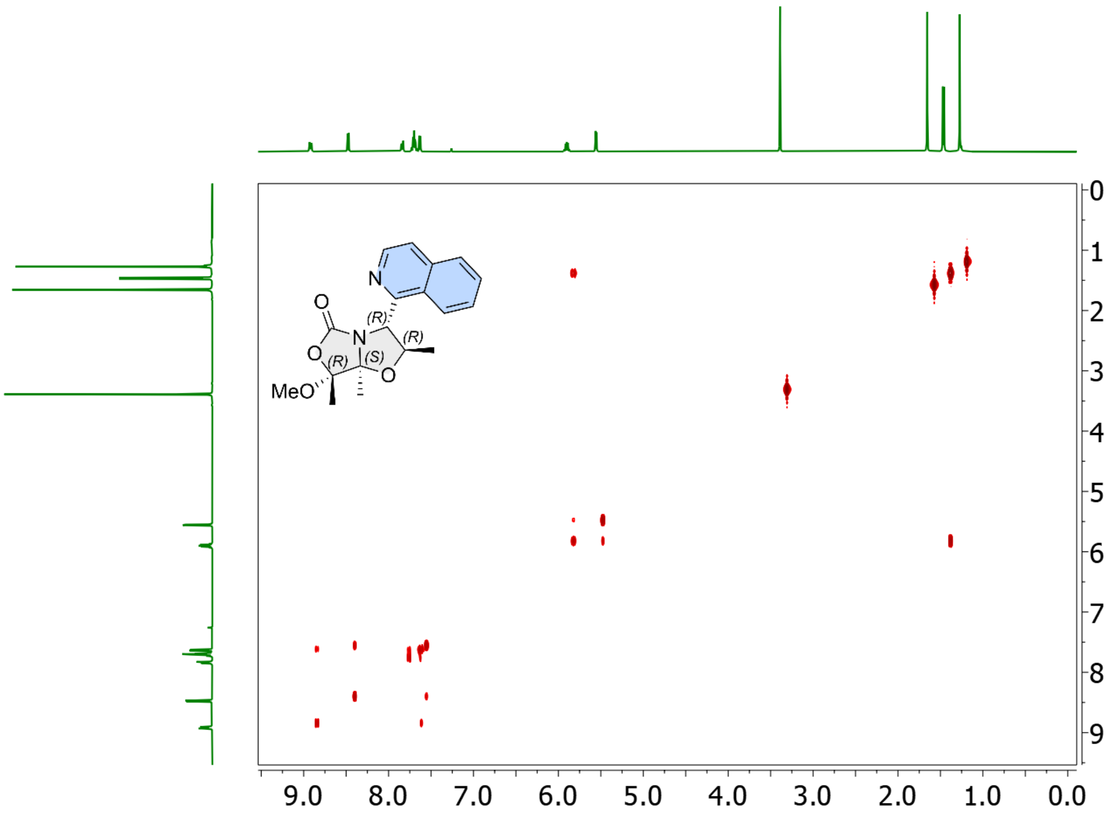

HSQC in $CDCl_3$ (400 MHz) compound 7

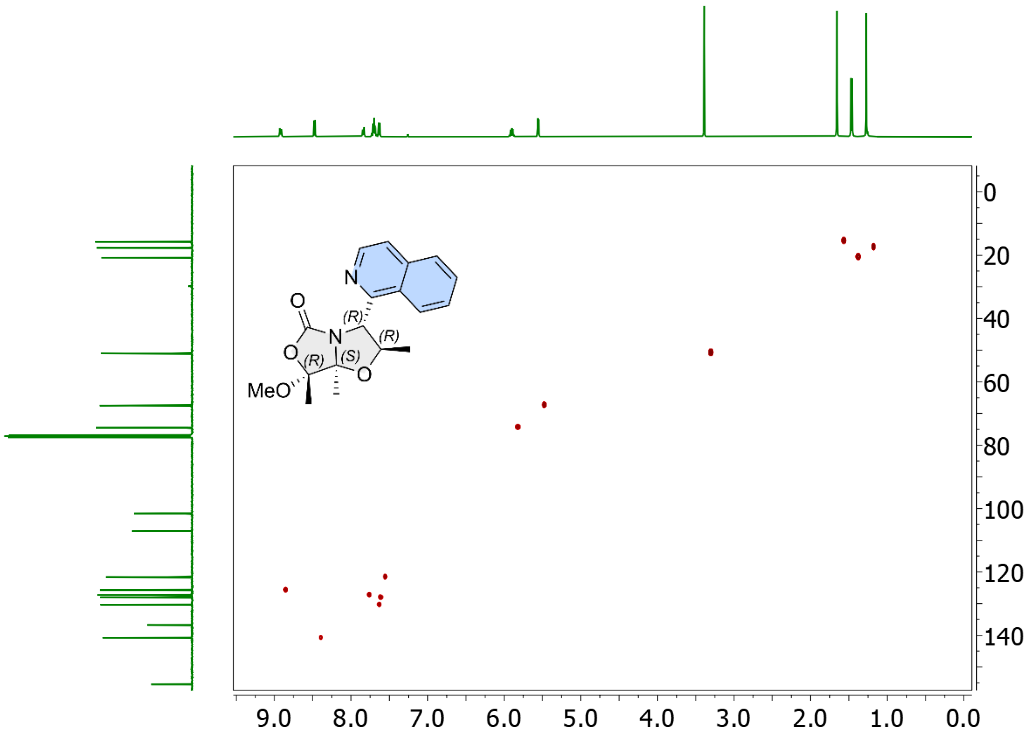

NOESY in $CDCl_3$ (400 MHz) compound 7

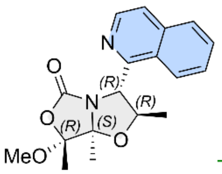

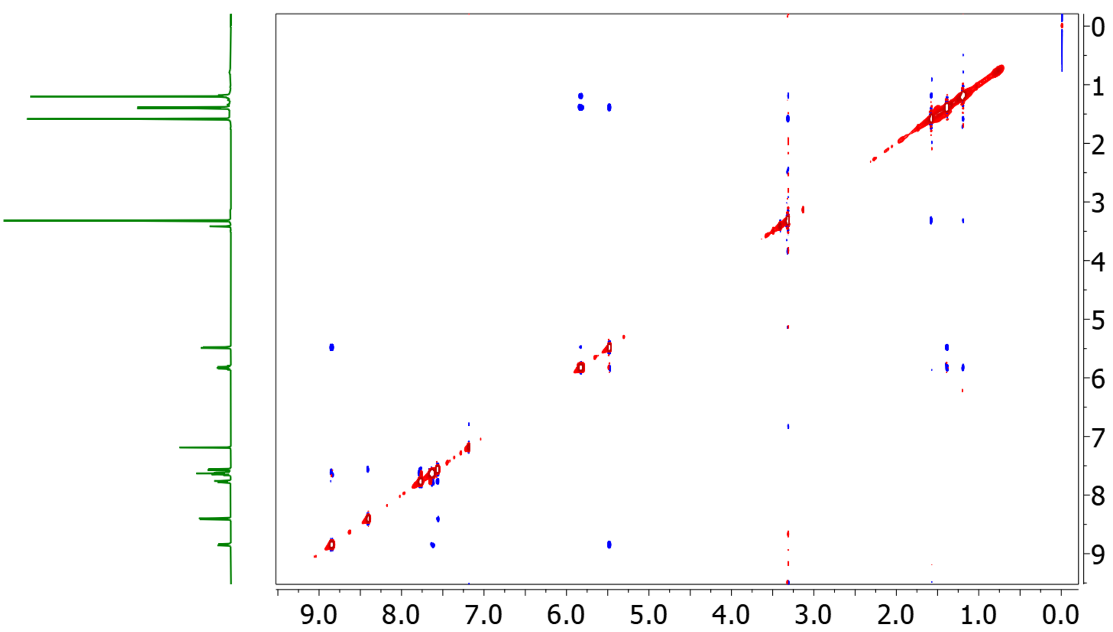

^{1}H NMR in $CDCl_3$ (400 MHz) compound 9

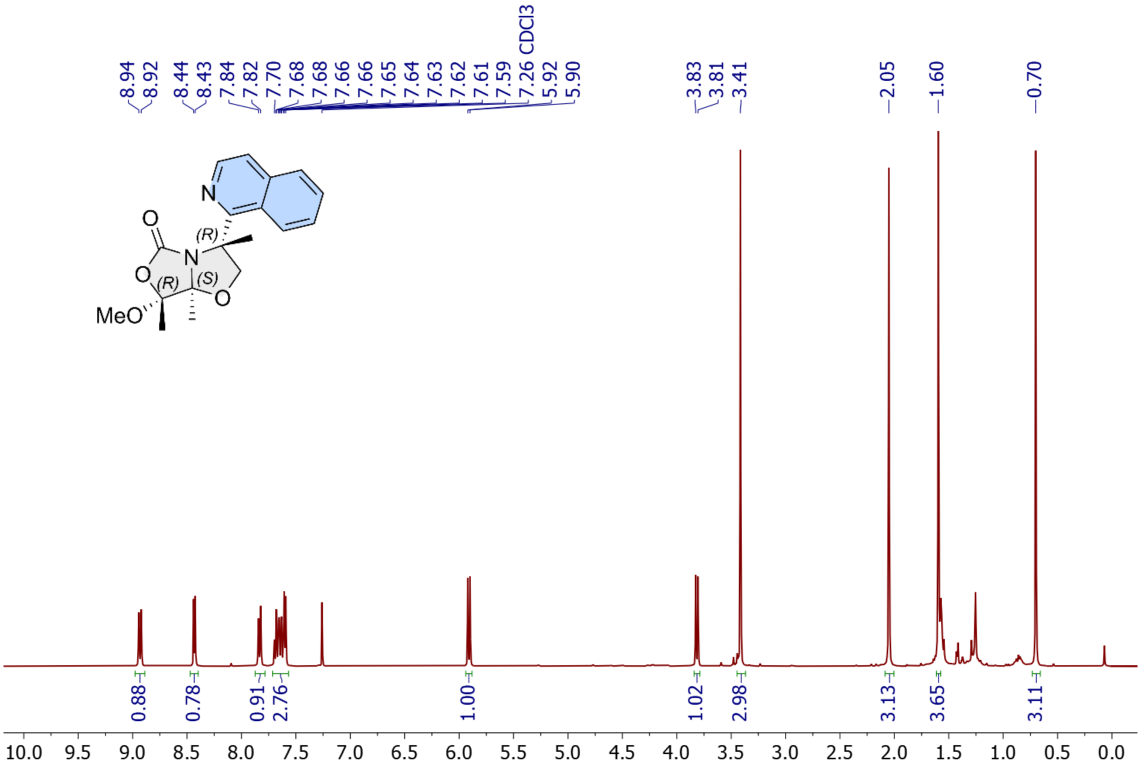

^{13}C {^{1}H} NMR in $CDCl_3$ (100 MHz) compound 9

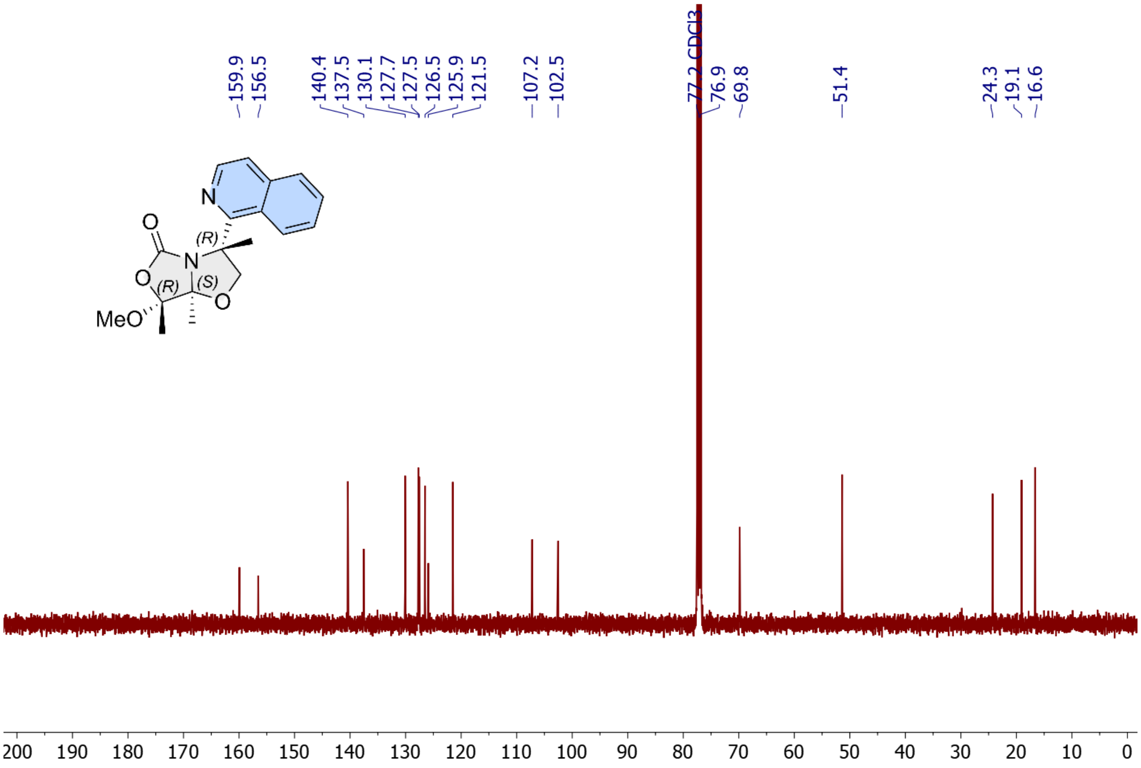

COSY in $CDCl_3$ (400 MHz) compound 9

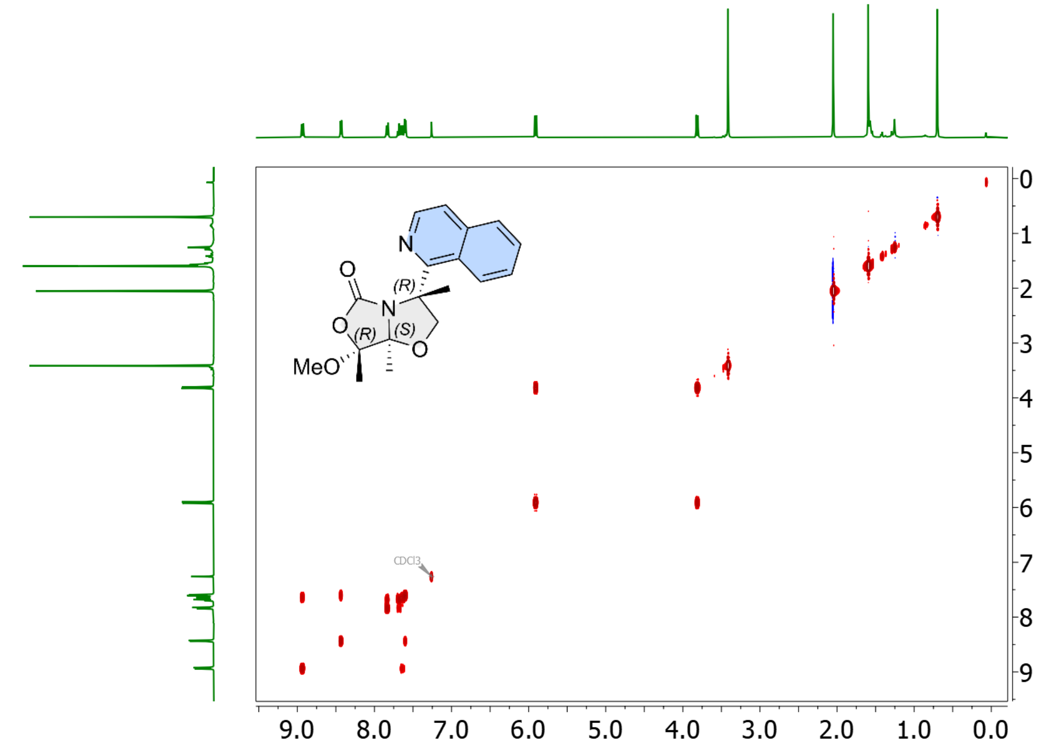

HSQC in $CDCl_3$ (400 MHz) compound 9

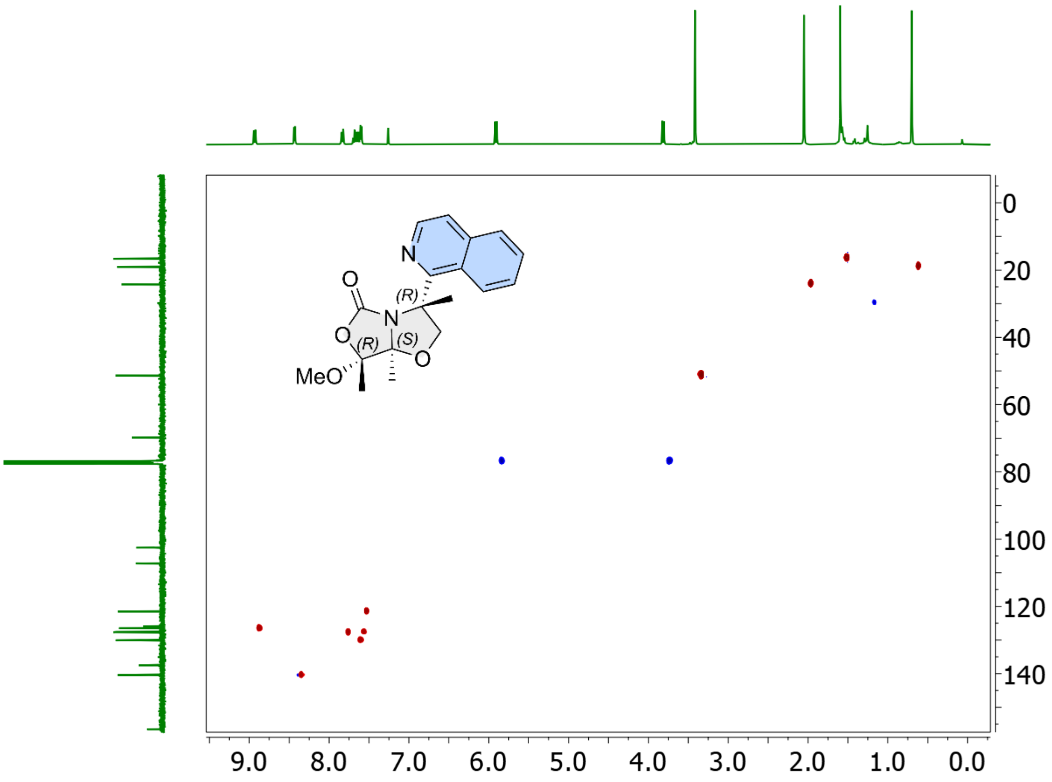

NOESY in $CDCl_3$ (400 MHz) compound 9

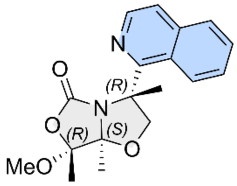

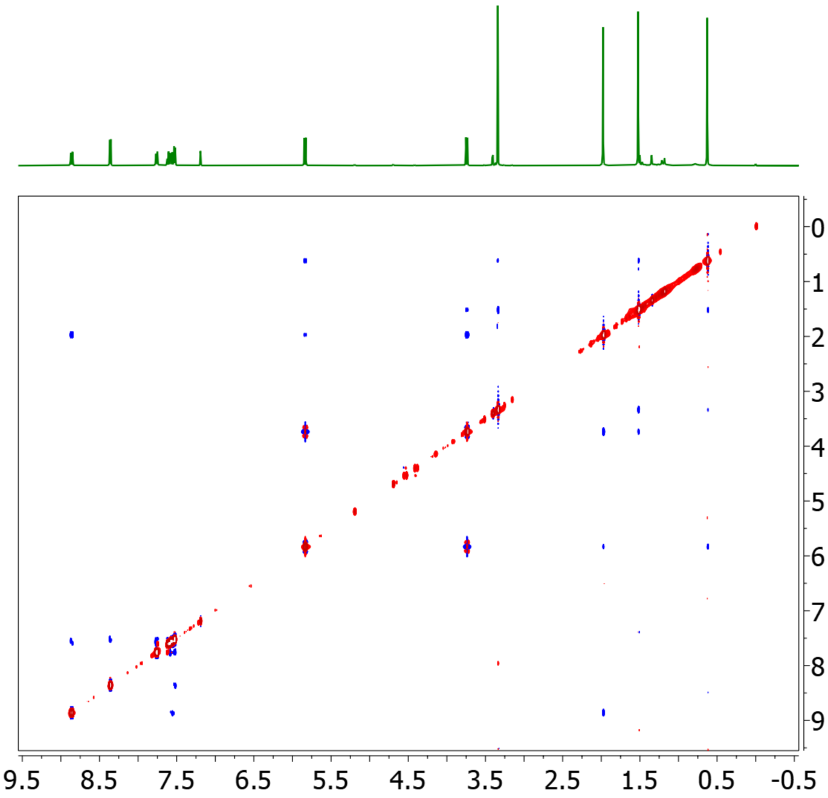

^{1}H NMR in $CDCl_3$ (400 MHz) compound 10

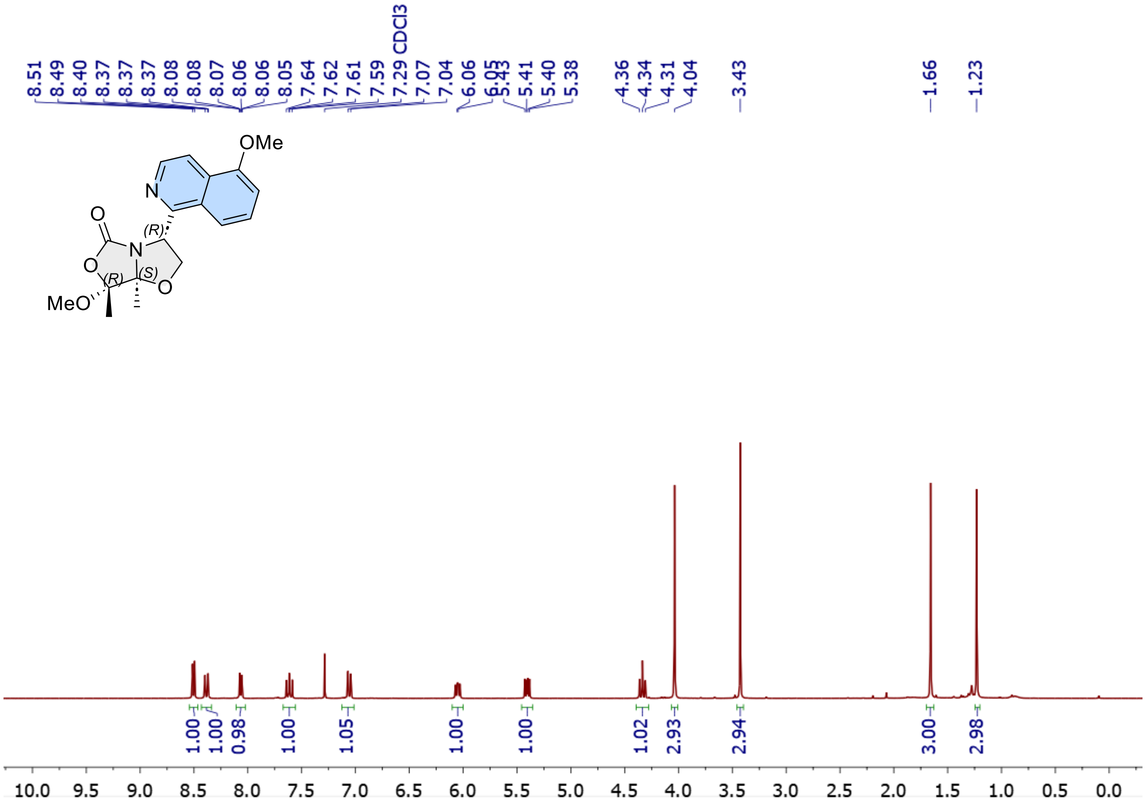

^{13}C {^{1}H} NMR in $CDCl_3$ (100 MHz) compound 10

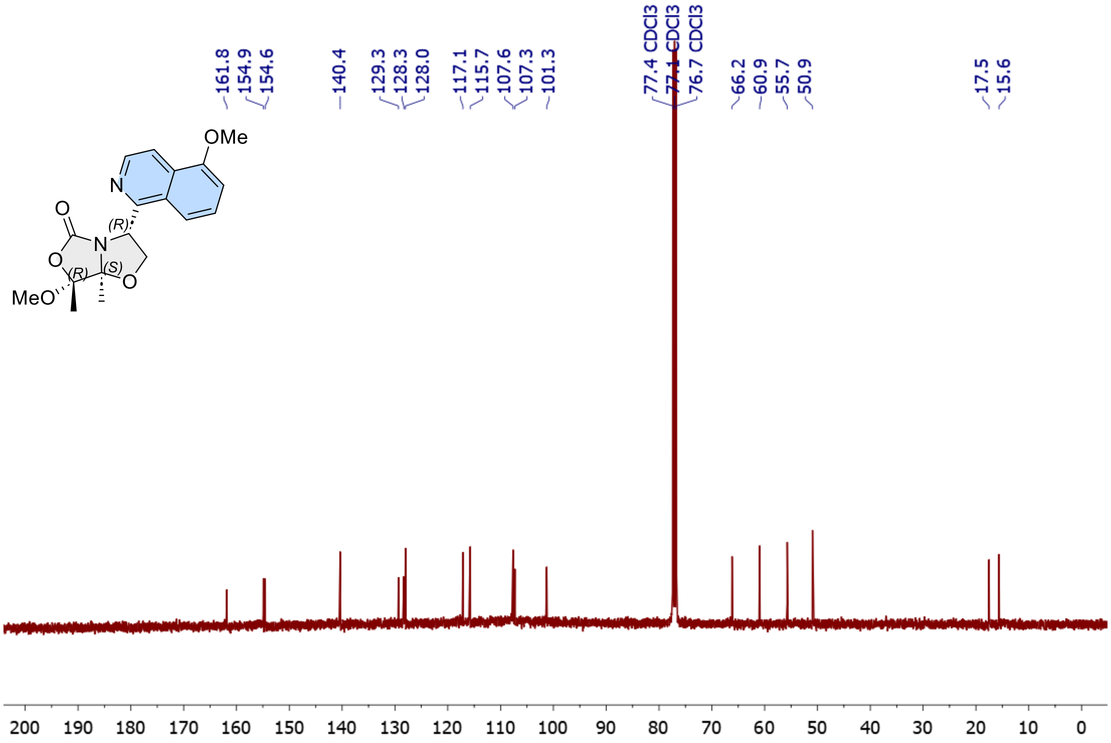

COSY in $CDCl_3$ (400 MHz) compound 10

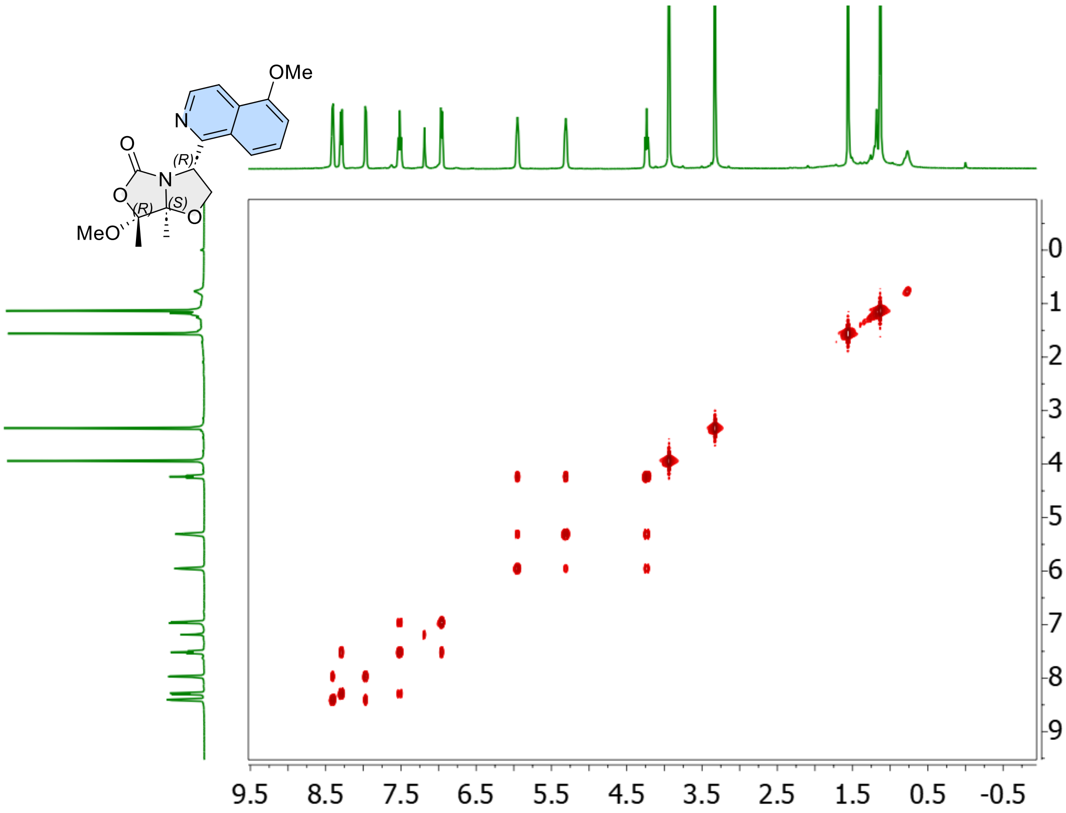

HSQC in $CDCl_3$ (400 MHz) compound 10

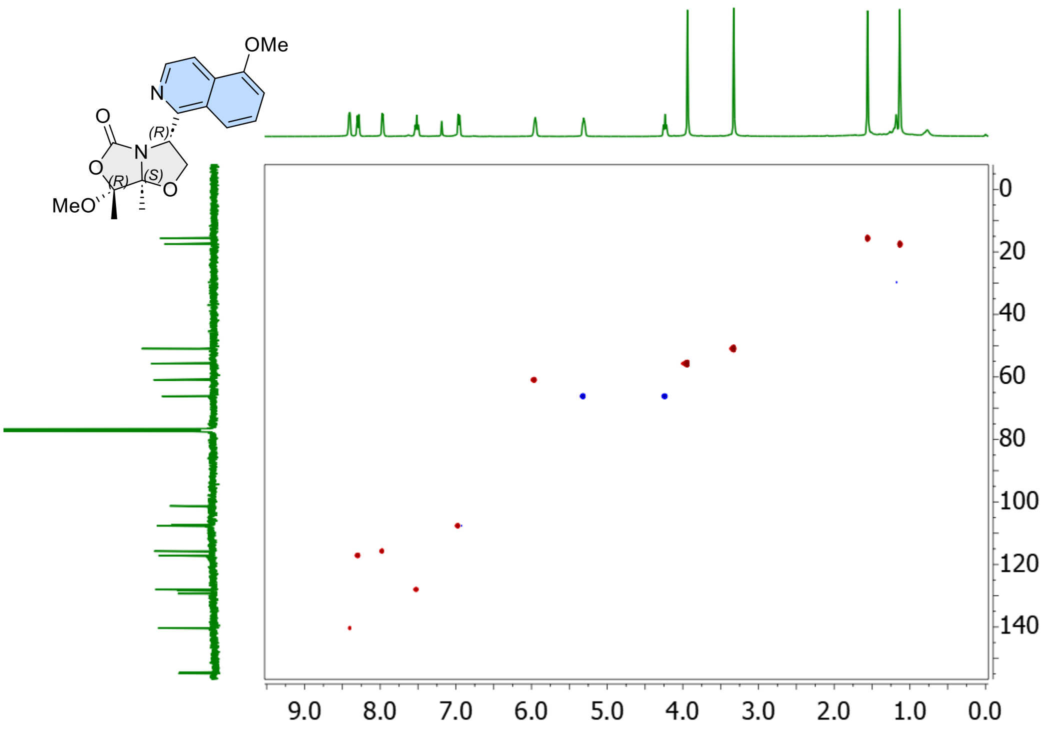

NOESY in $CDCl_3$ (400 MHz) compound 10

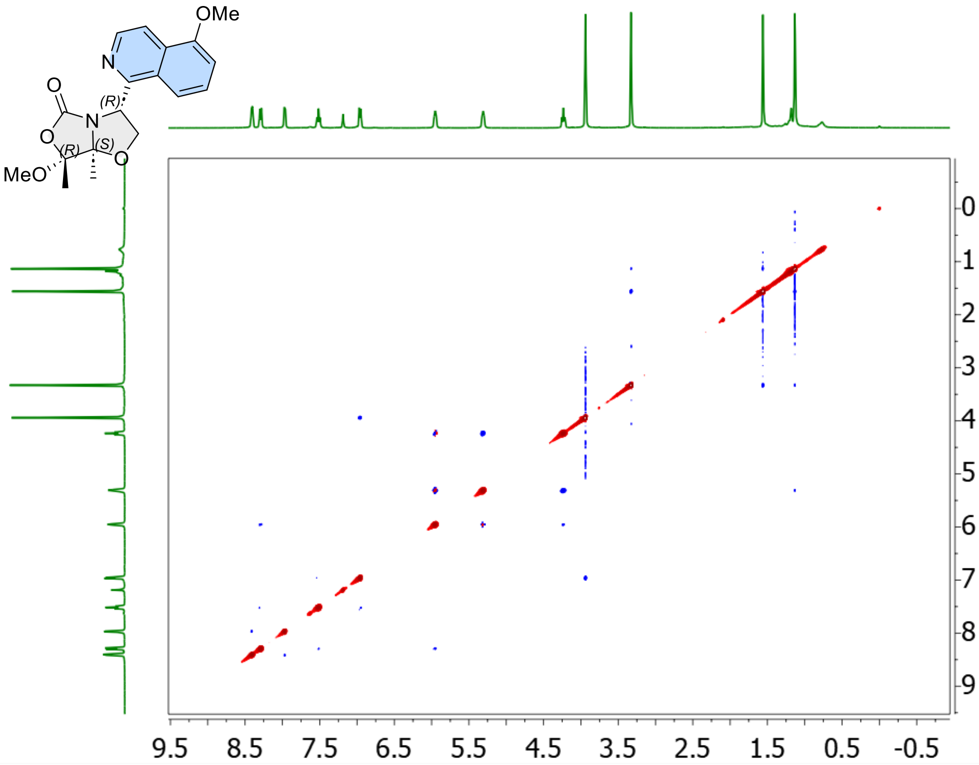

^{1}H NMR in $CDCl_3$ (400 MHz) compound 11

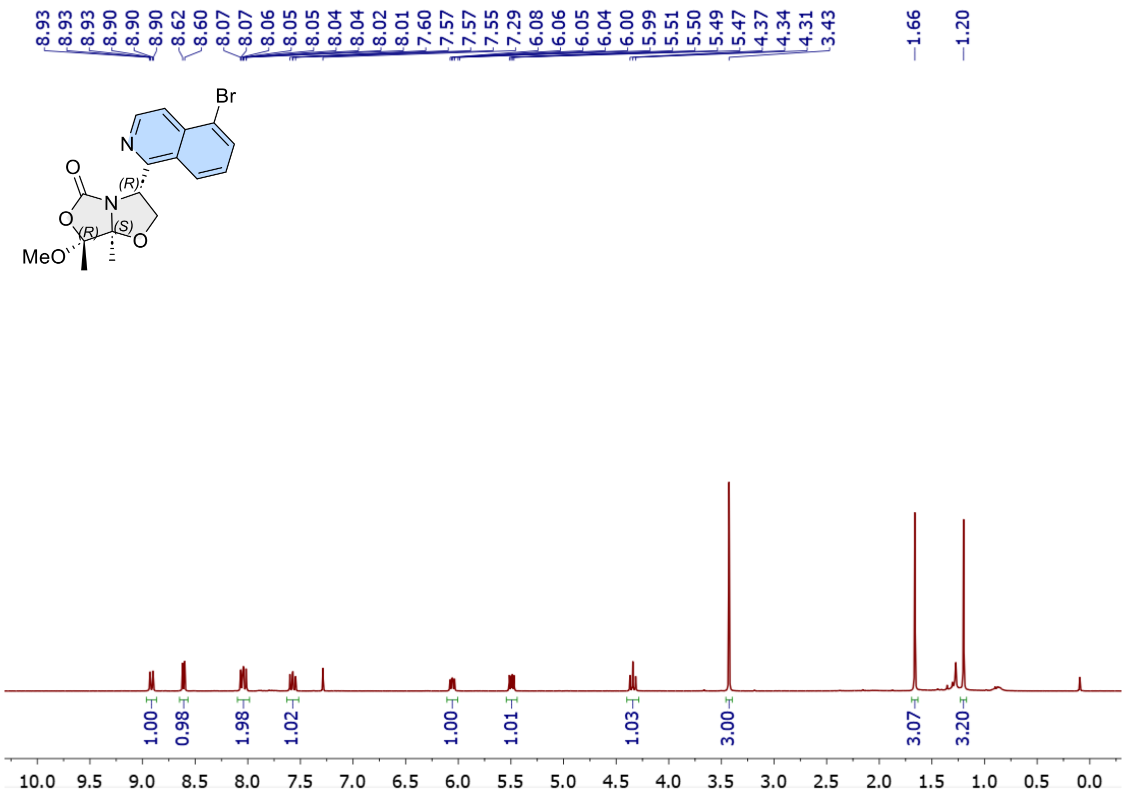

^{13}C {^{1}H} NMR in $CDCl_3$ (100 MHz) compound 11

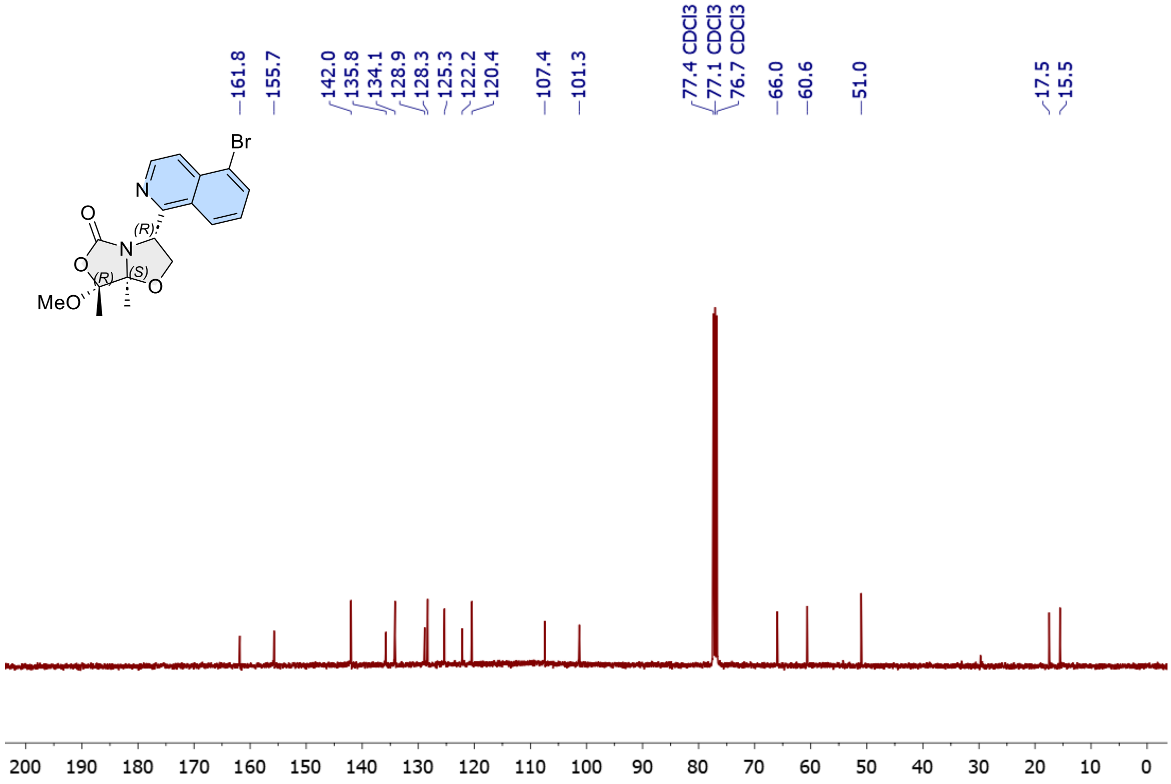

COSY in $CDCl_3$ (400 MHz) compound 11

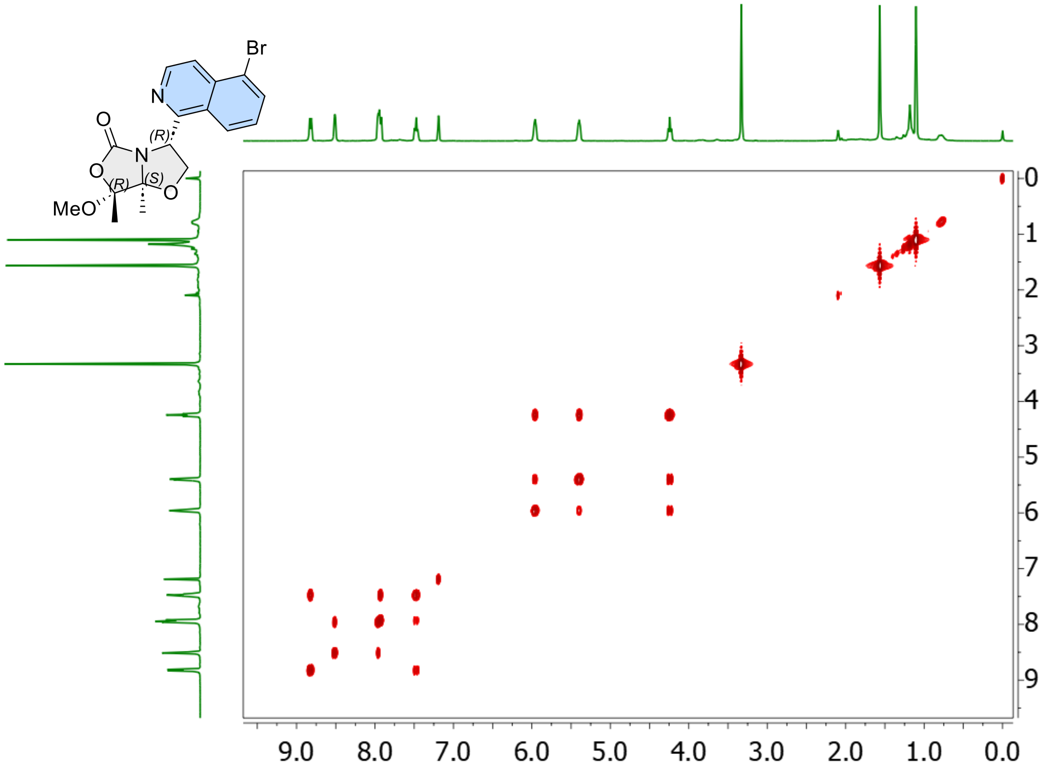

HSQC in $CDCl_3$ (400 MHz) compound 11

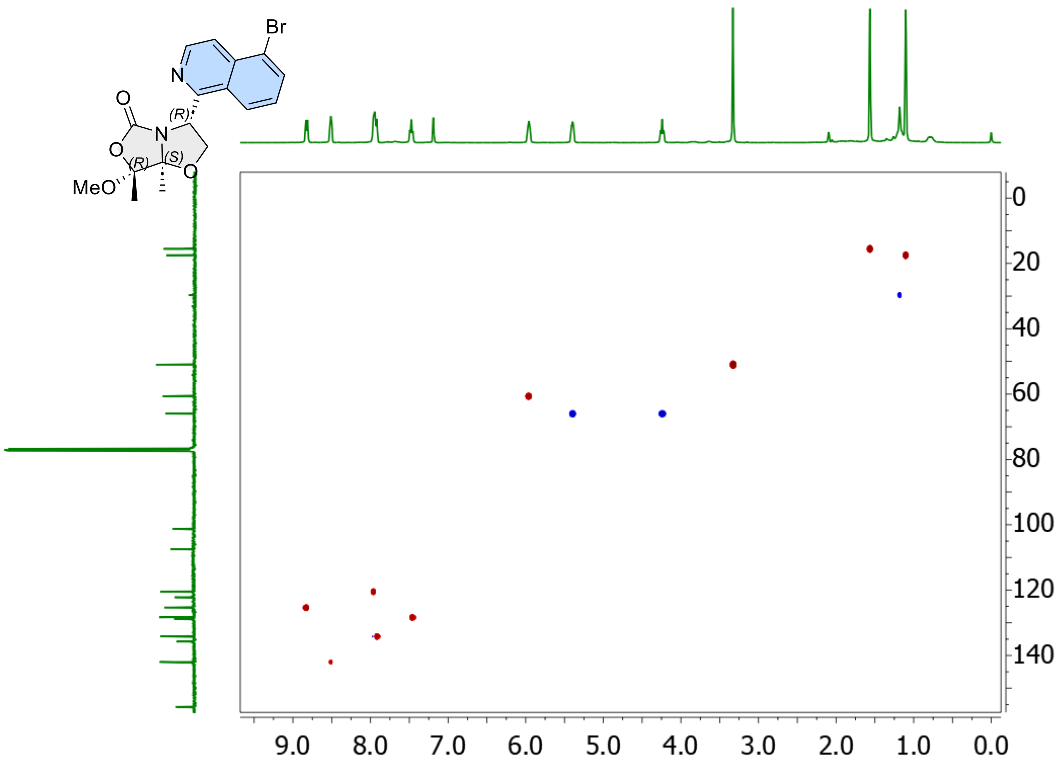

NOESY in $CDCl_3$ (400 MHz) compound 11

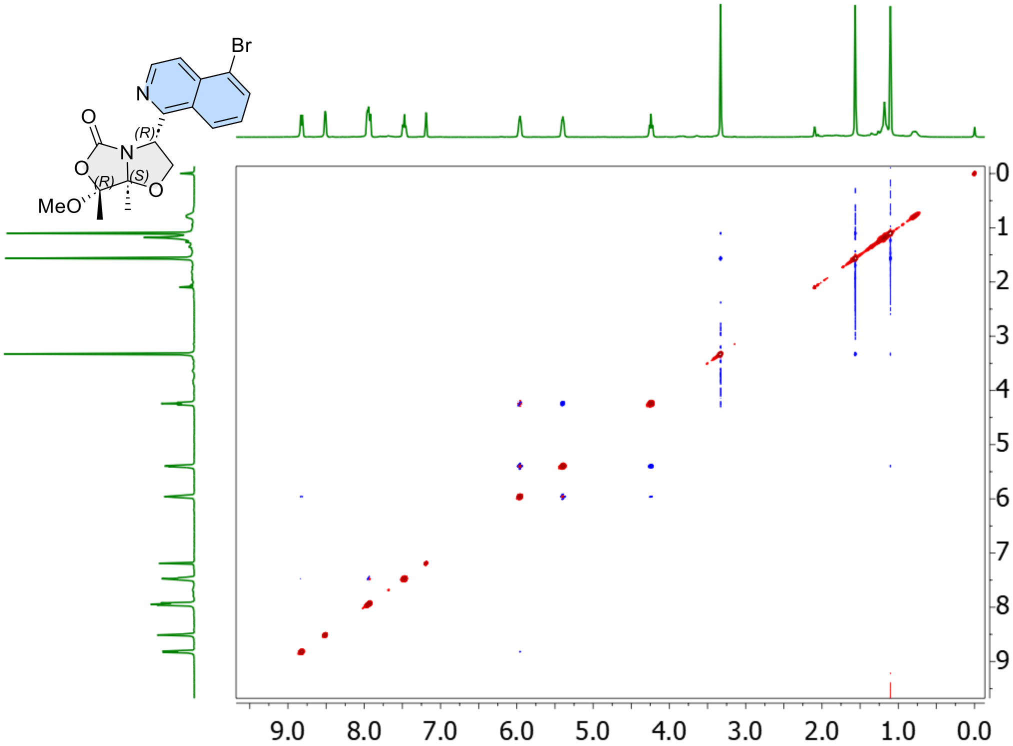

^1H NMR in $CDCl_3$ (400 MHz) compound 12

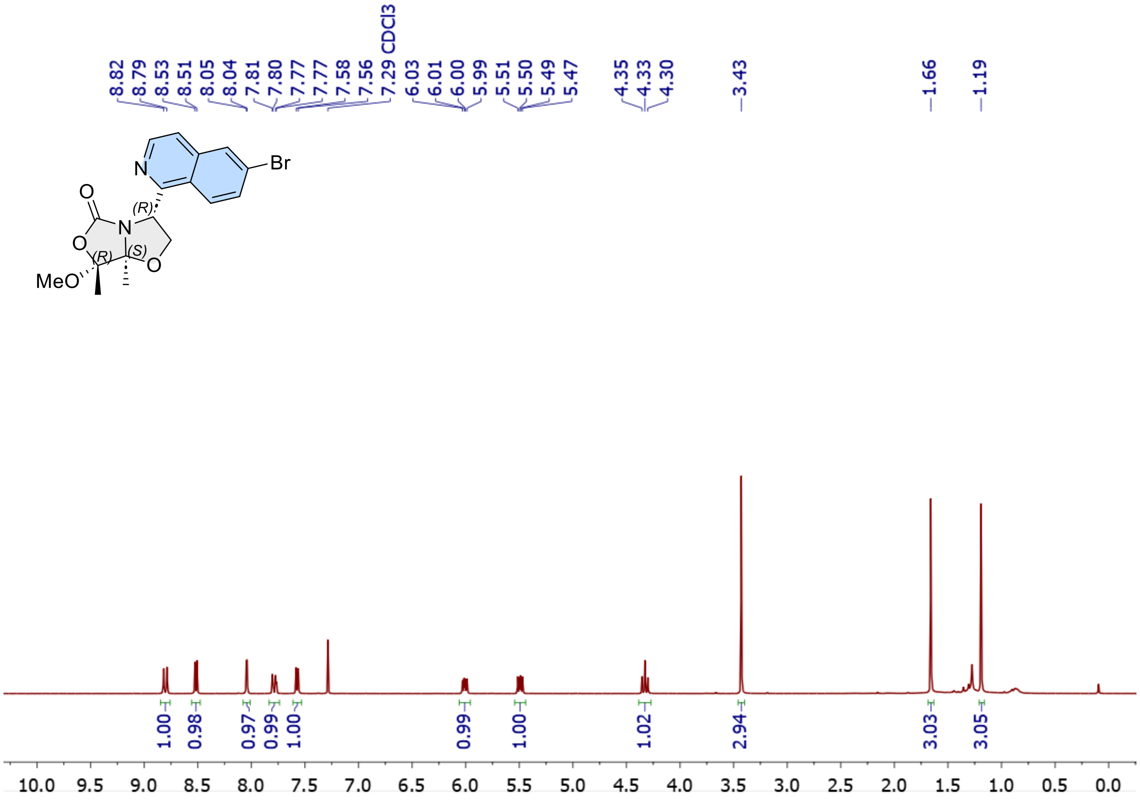

^{13}C {^1H} NMR in $CDCl_3$ (100 MHz) compound 12

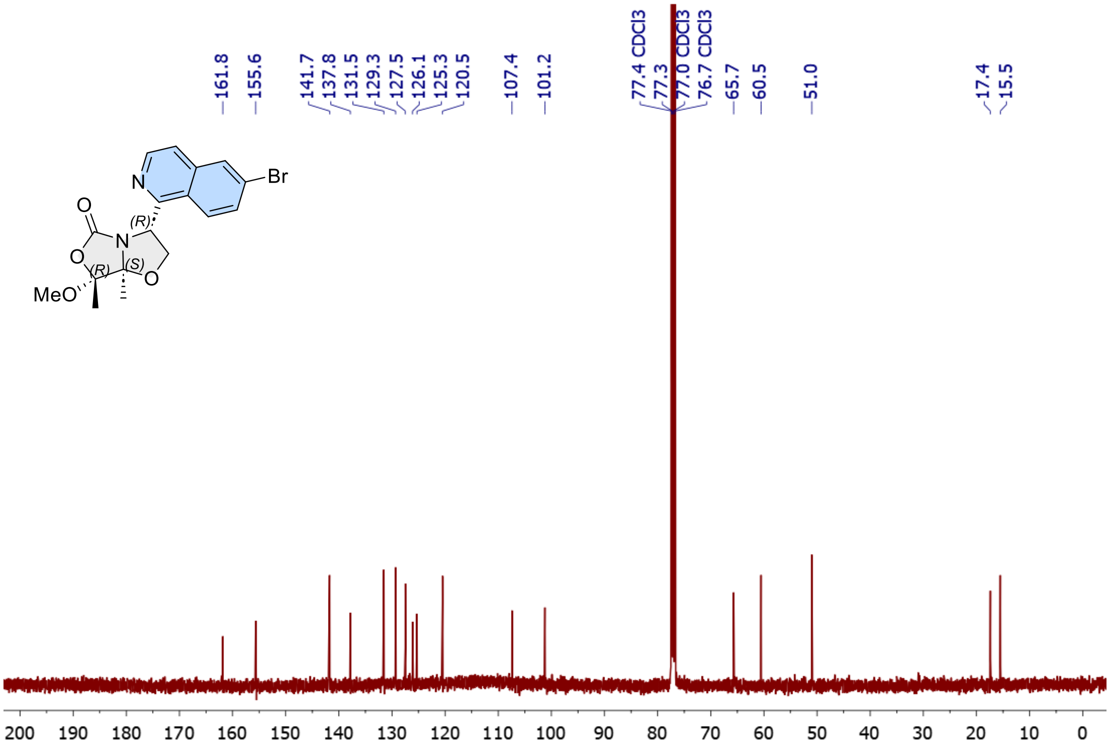

COSY in $CDCl_3$ (400 MHz) compound 12

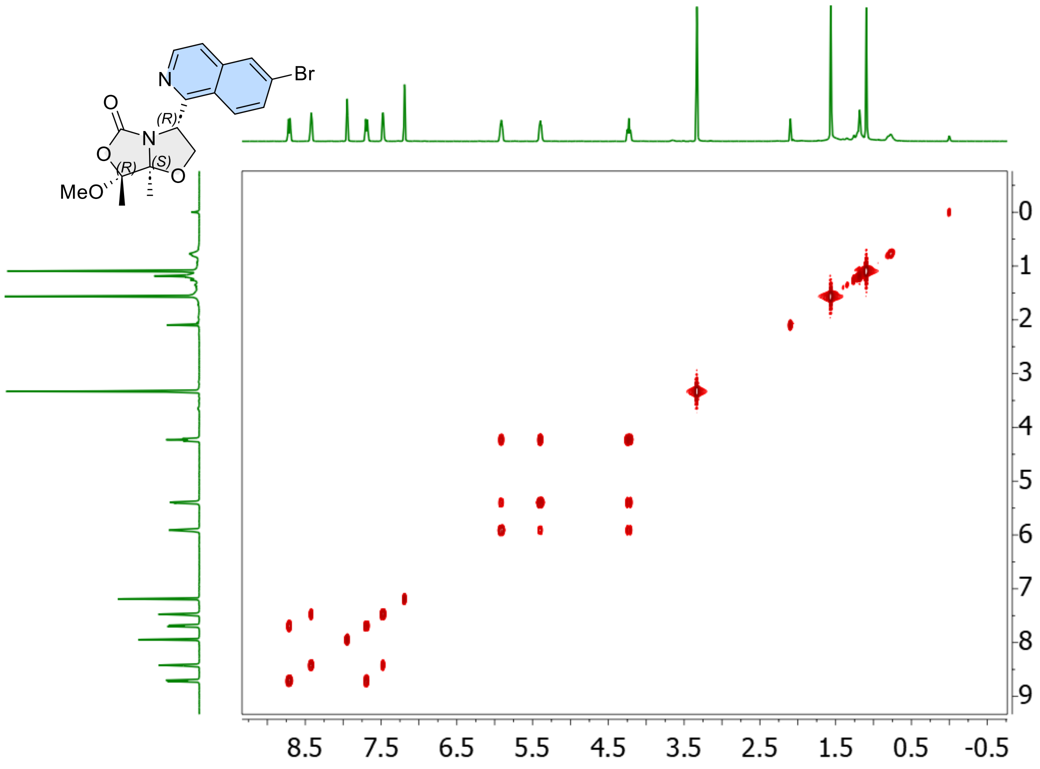

HSQC in $CDCl_3$ (400 MHz) compound 12

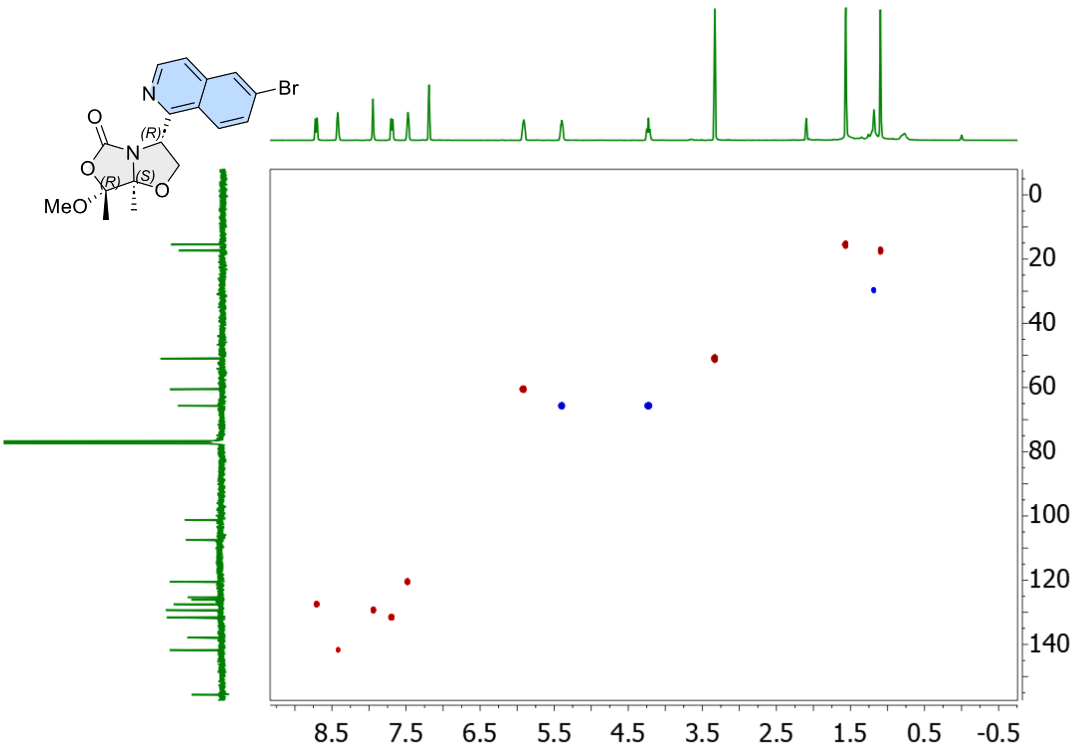

NOESY in $CDCl_3$ (400 MHz) compound 12

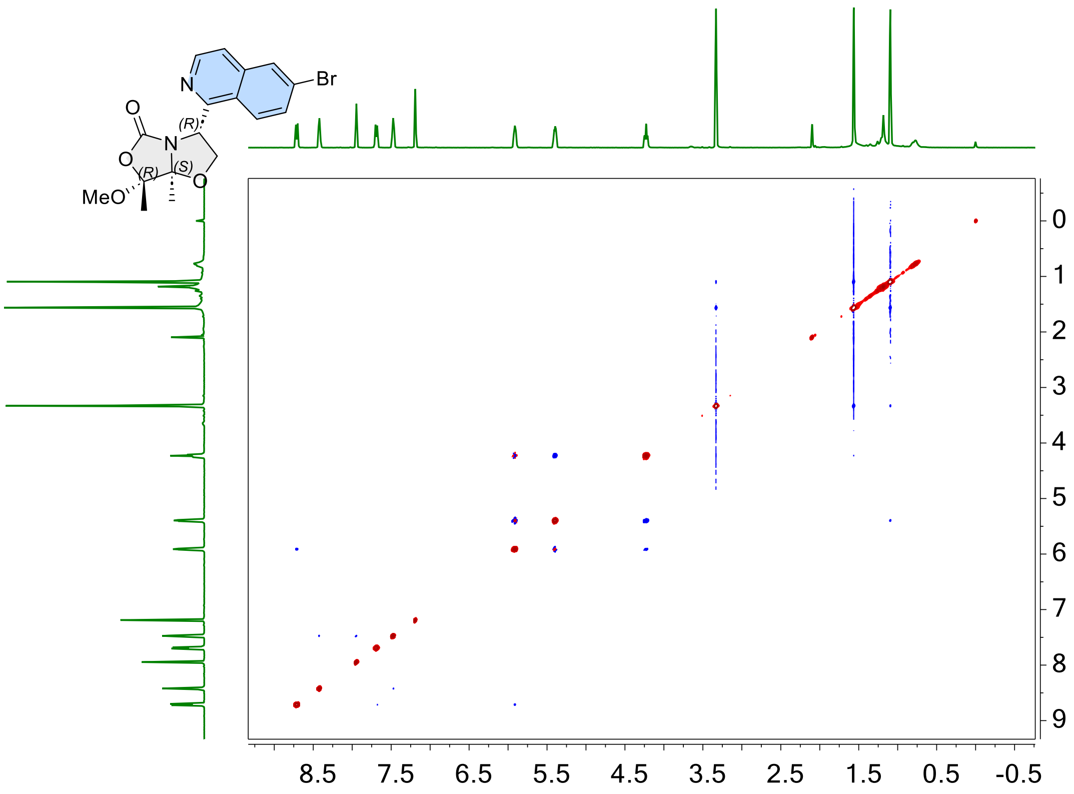

^{1}H NMR in $CDCl_3$ (400 MHz) compound 13

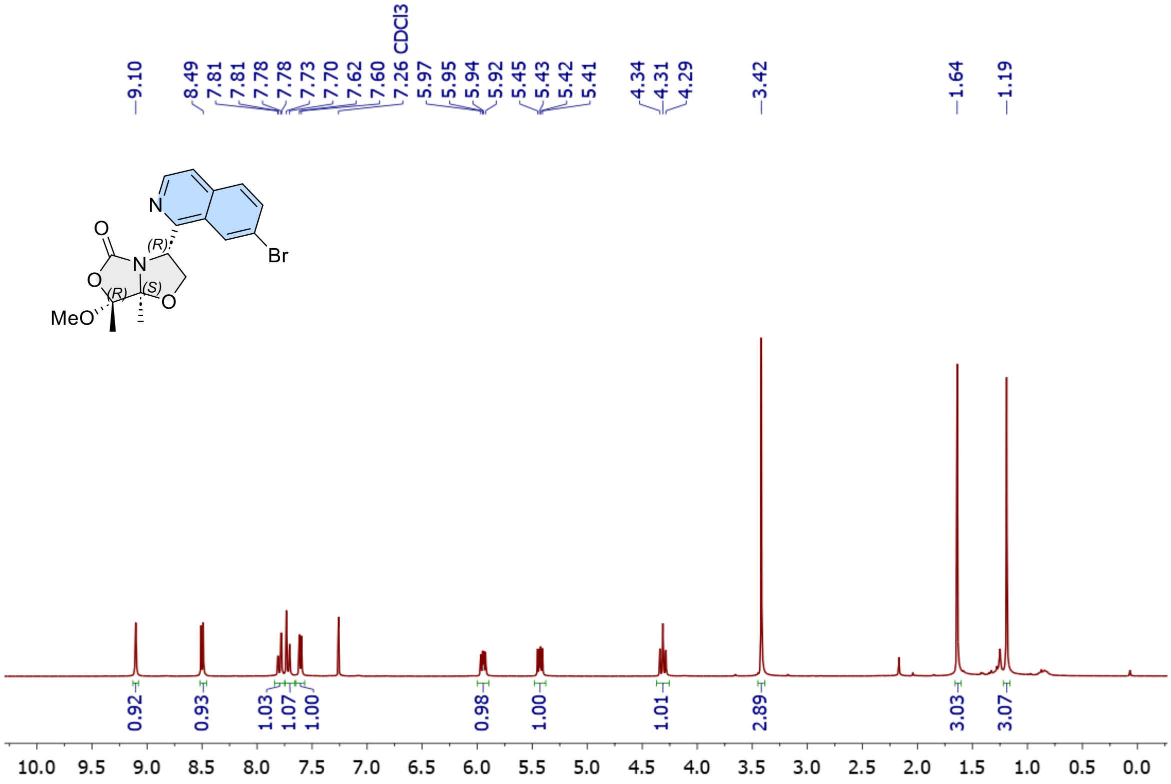

^{13}C {^{1}H} NMR in $CDCl_3$ (100 MHz) compound 13

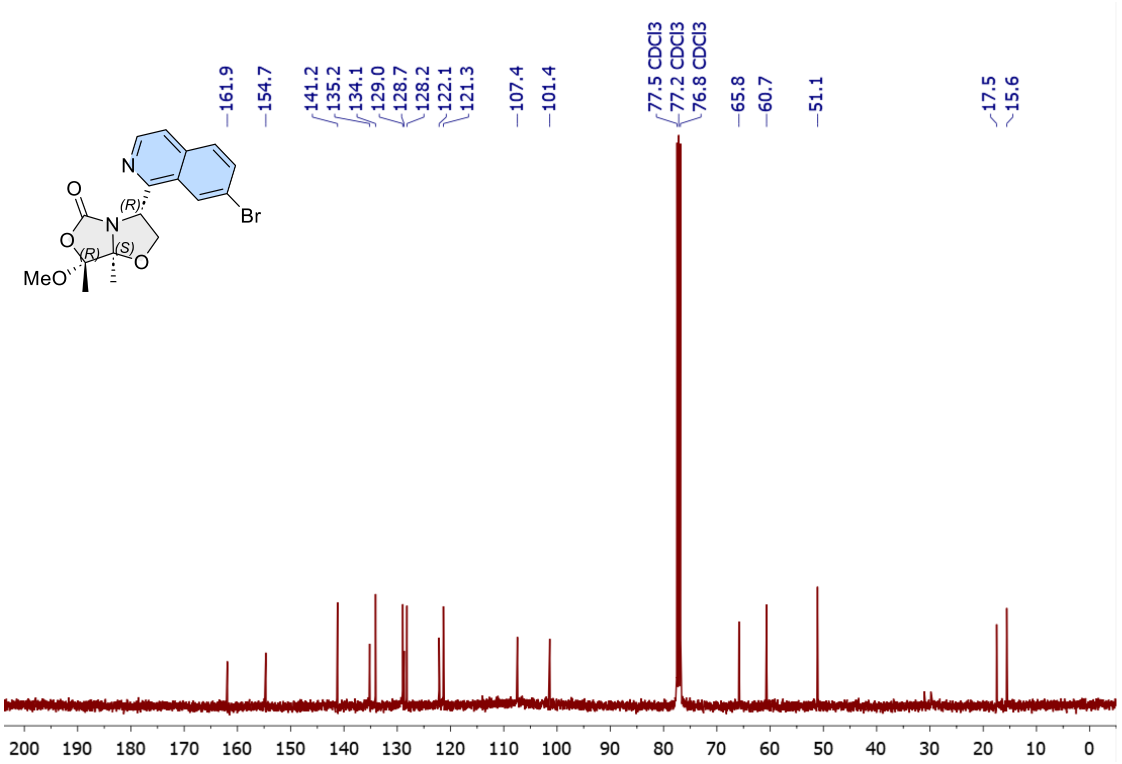

COSY in $CDCl_3$ (400 MHz) compound 13

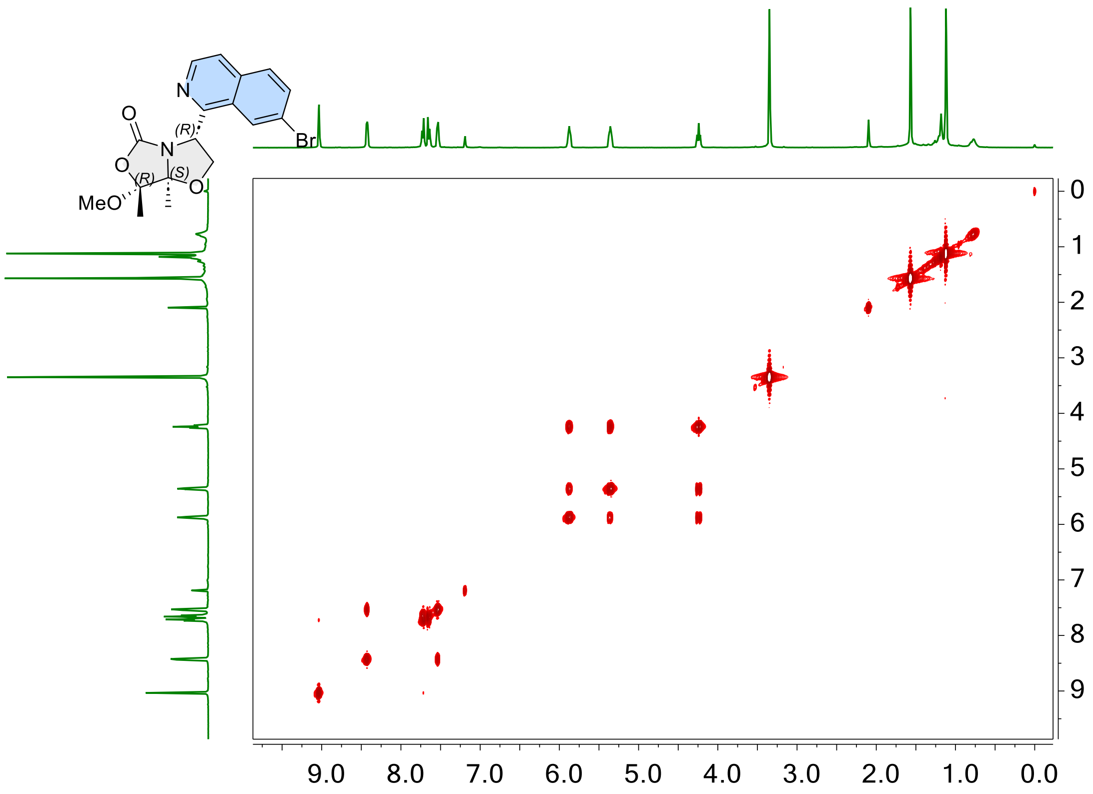

HSQC in $CDCl_3$ (400 MHz) compound 13

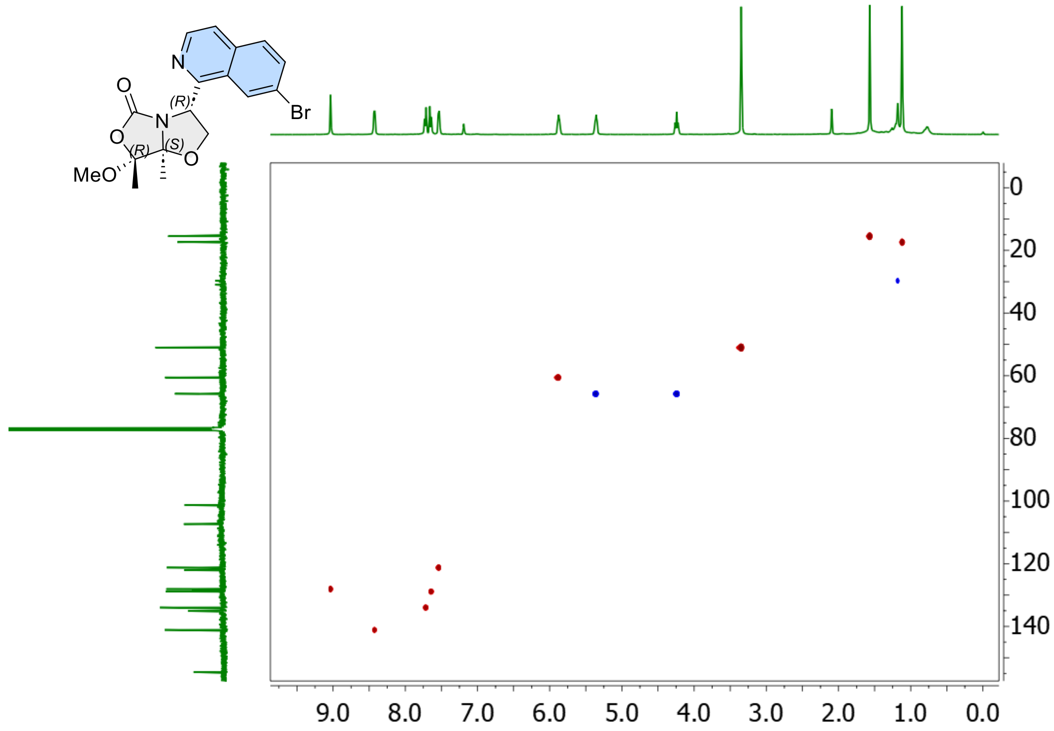

NOESY in $CDCl_3$ (400 MHz) compound 13

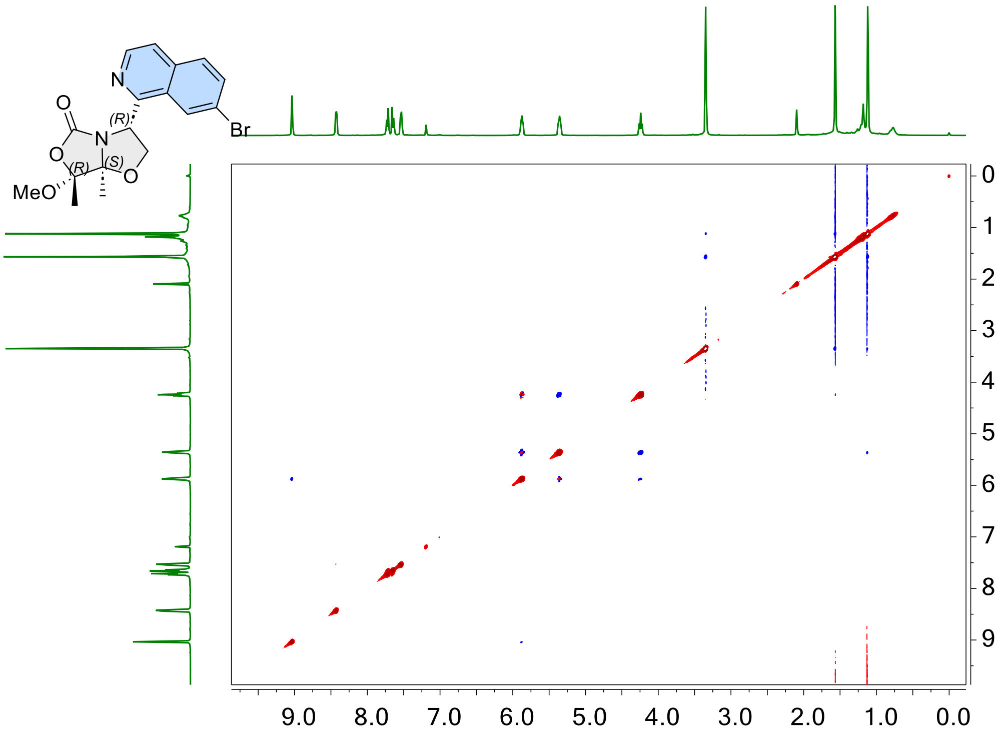

^{1}H NMR in $CDCl_3$ (400 MHz) compound 14

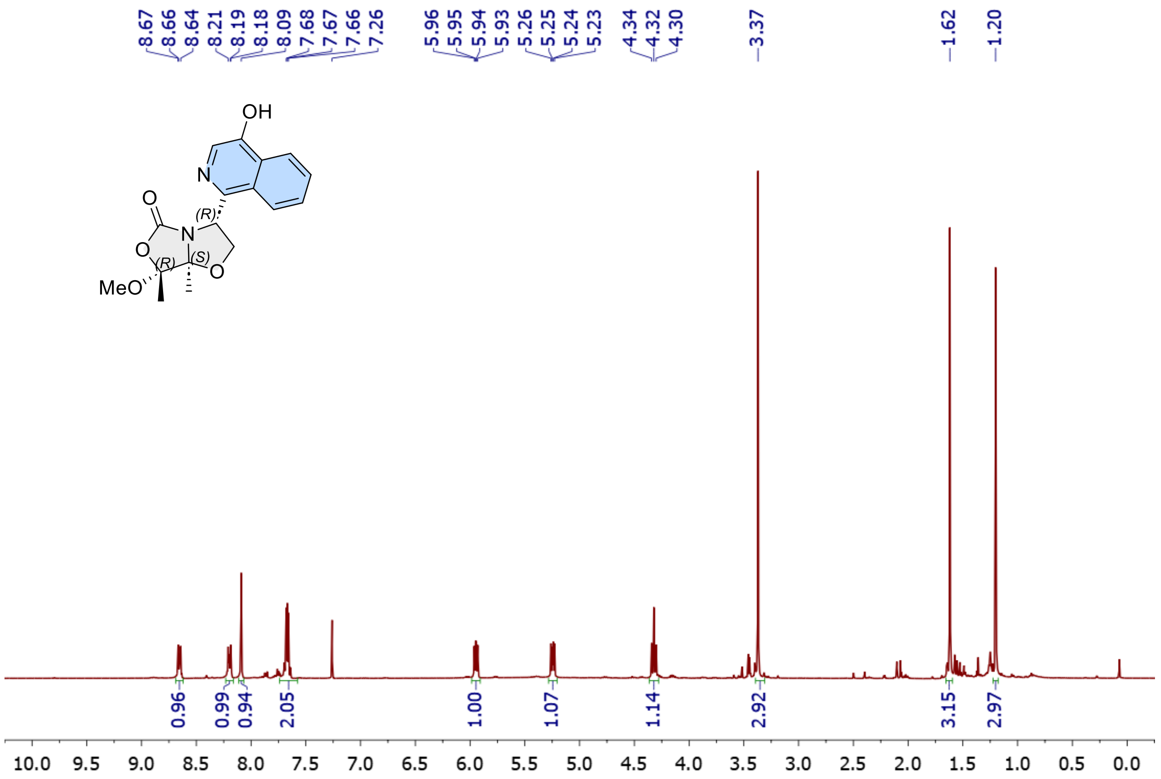

^{13}C {^{1}H} NMR in $CDCl_3$ (100 MHz) compound 14

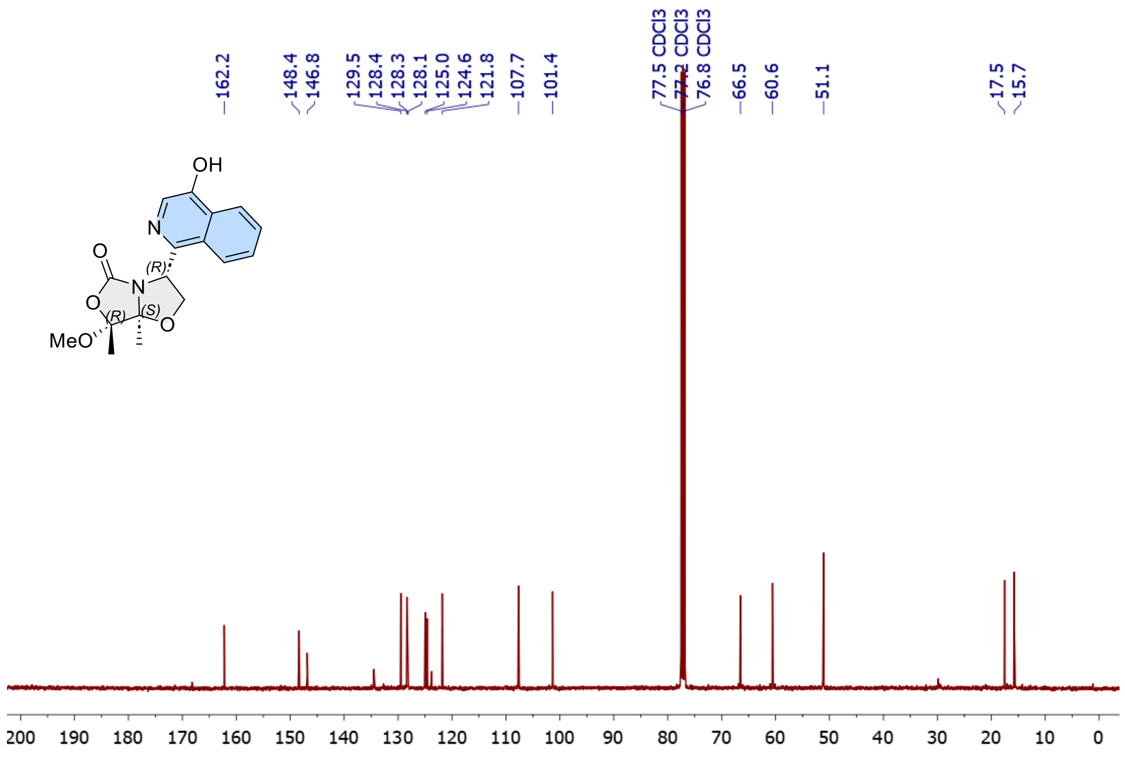

COSY in $CDCl_3$ (400 MHz) compound 14

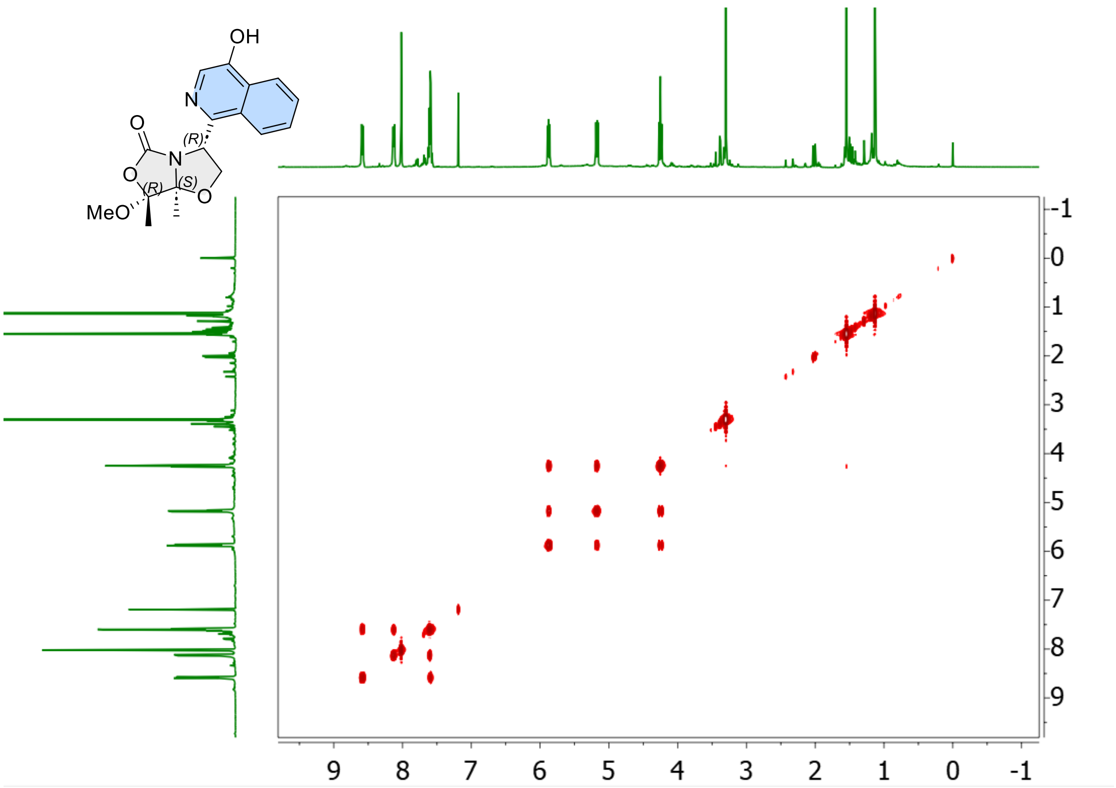

HSQC in $CDCl_3$ (400 MHz) compound 14

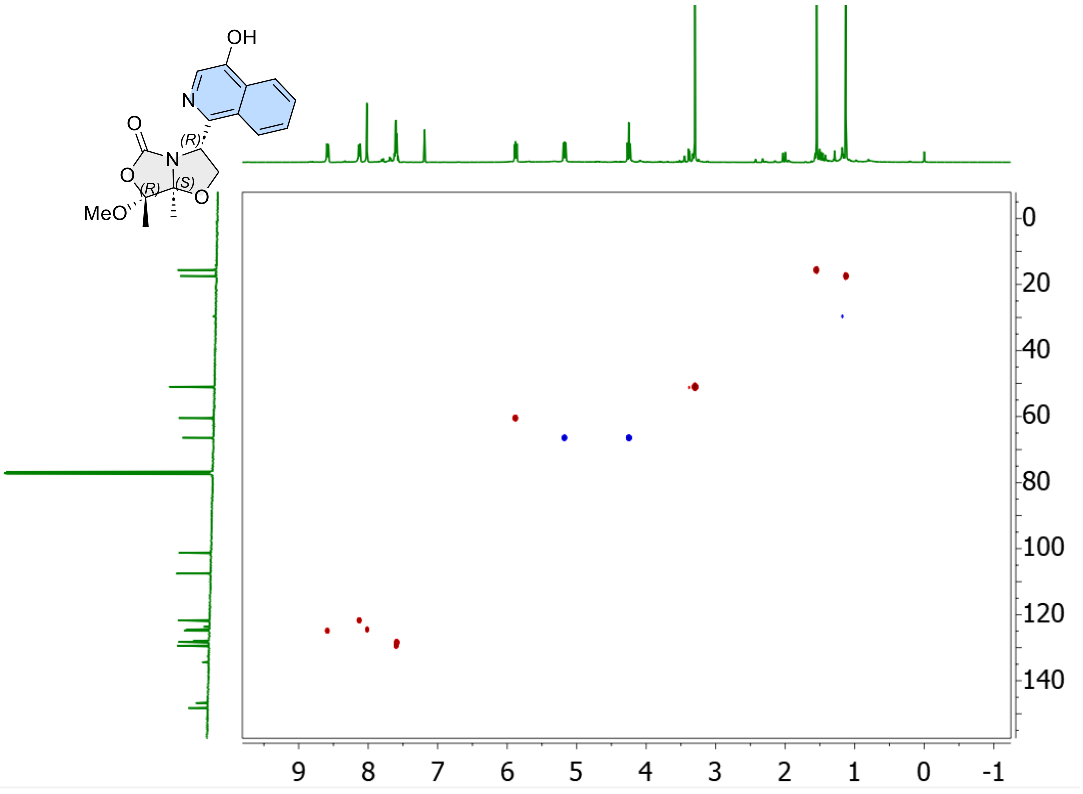

NOESY in CDCl$_3$ (400 MHz) compound 14

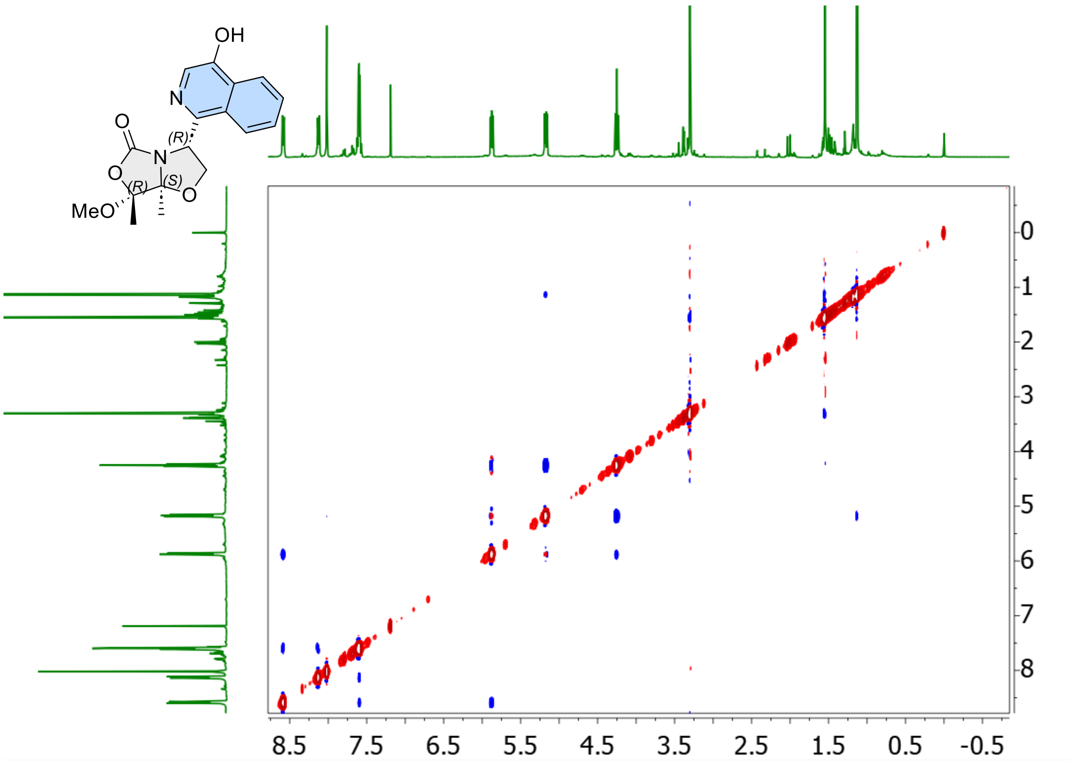

^{1}H NMR in DMF-d7 (400 MHz) compound 15

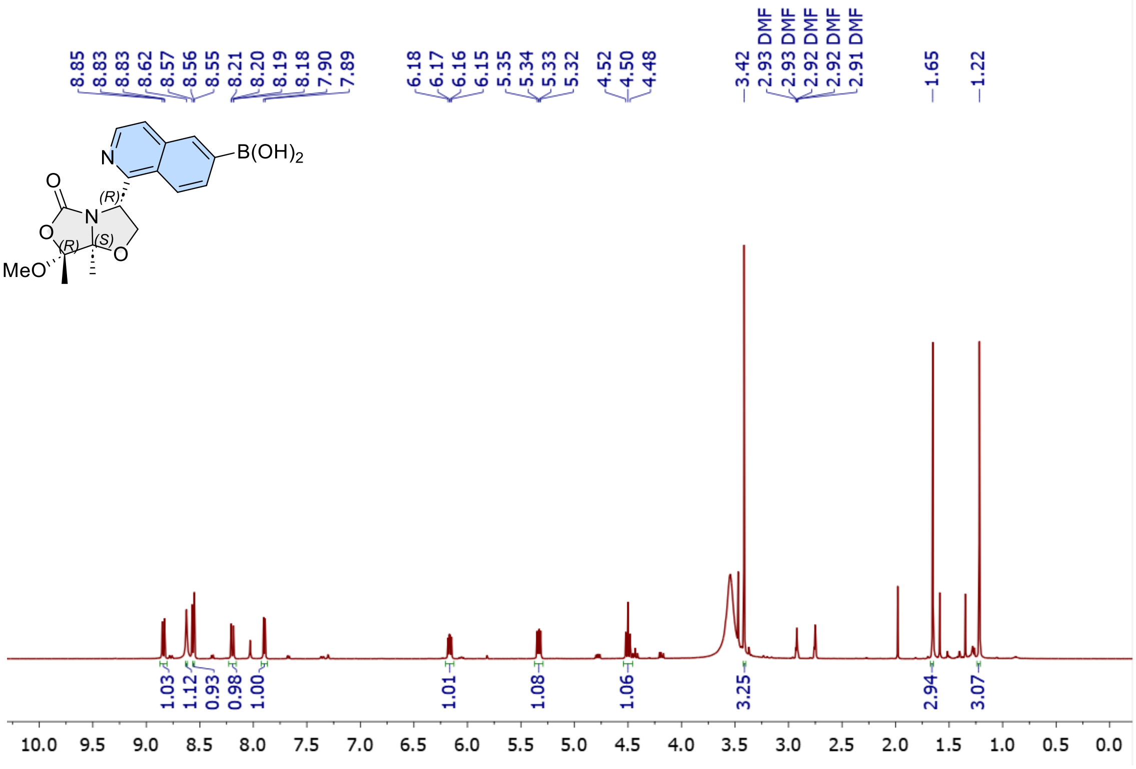

^{13}C {^{1}H} NMR in DMF-d7 (100 MHz) compound 15

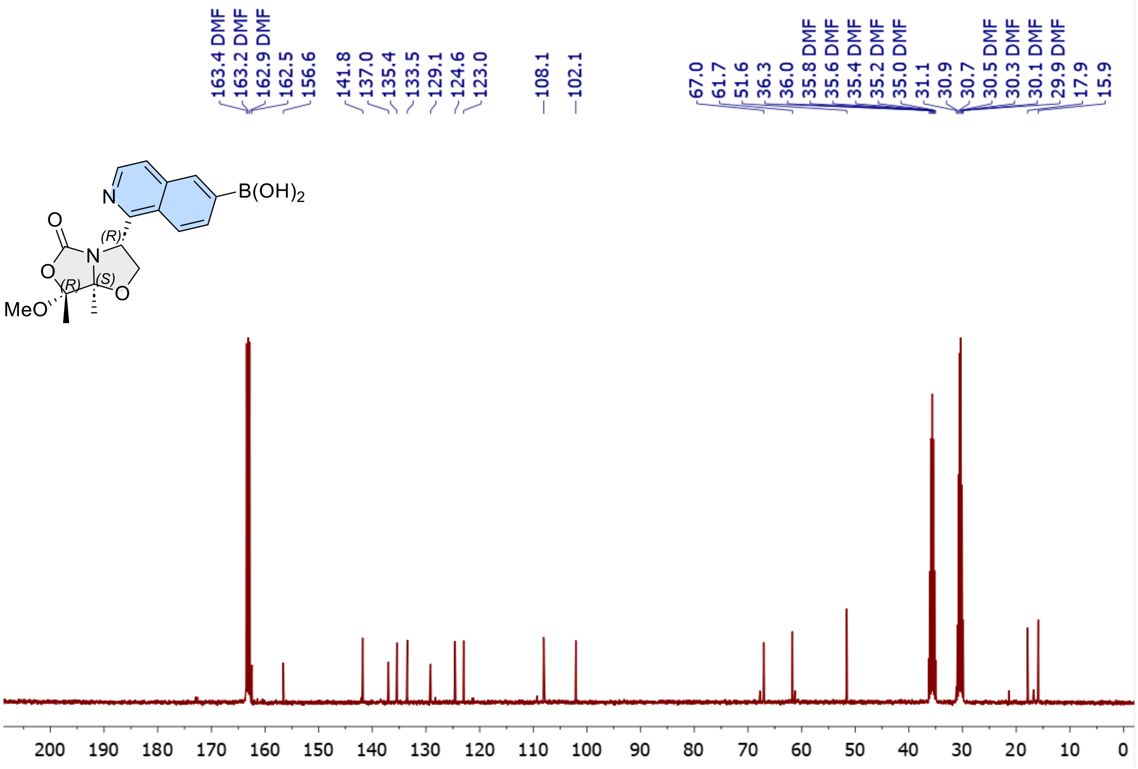

COSY in DMF-d7 (400 MHz) compound 15

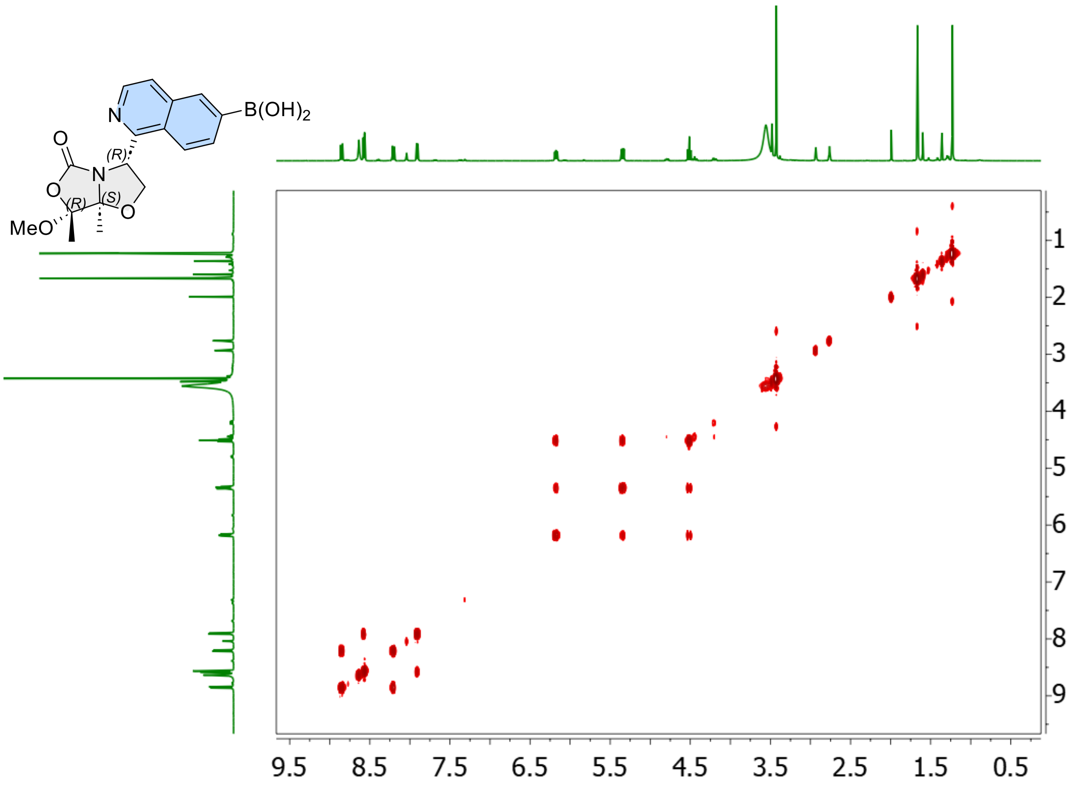

HSQC in DMF-d7 (400 MHz) compound 15

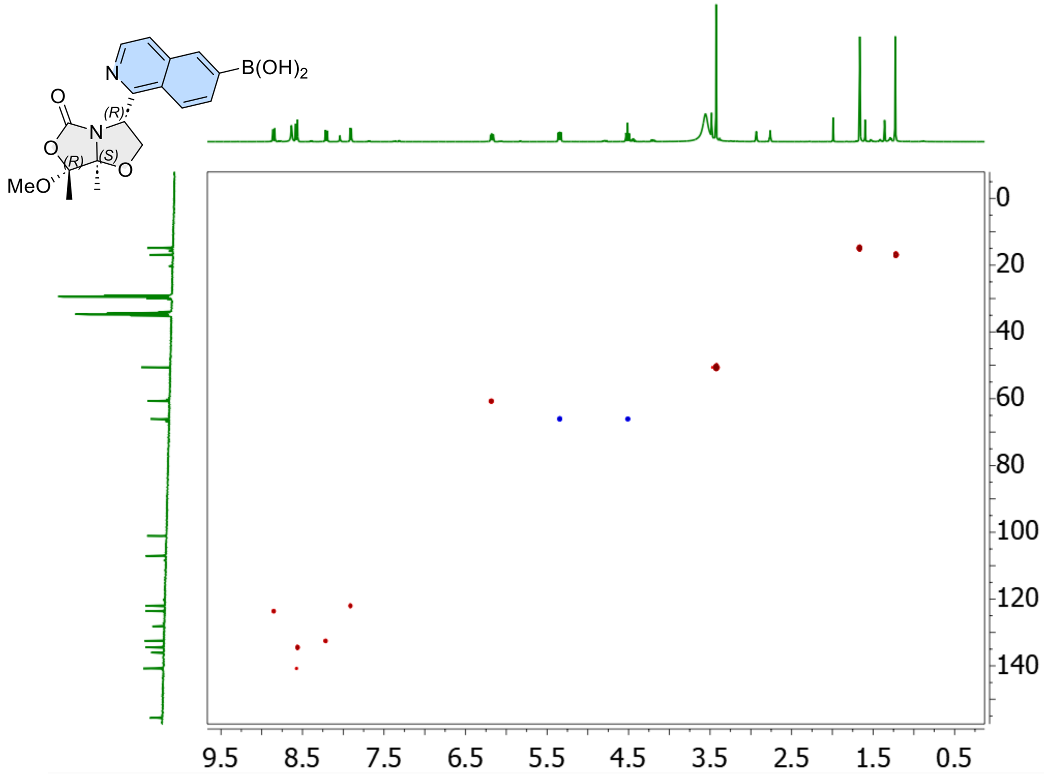

NOESY in DMF-d7 (400 MHz) compound 15

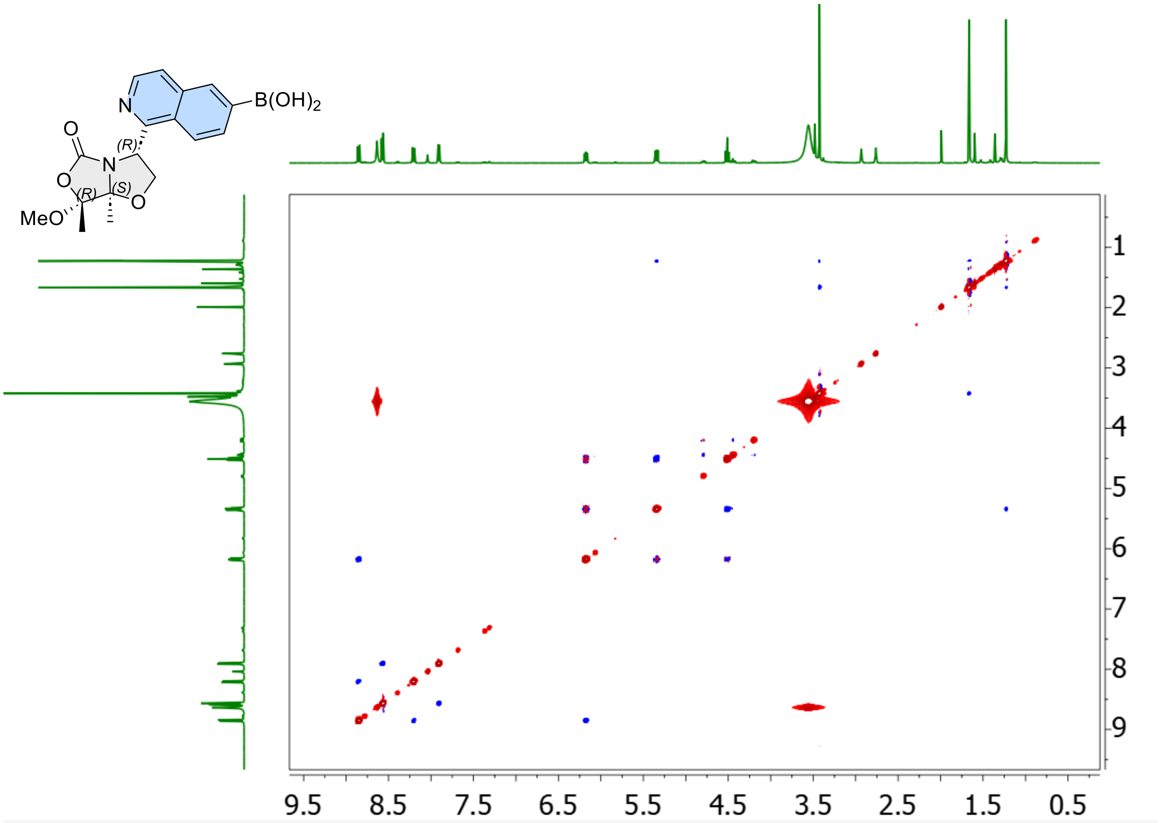

^{1}H NMR in $CDCl_3$ (400 MHz) compound 16

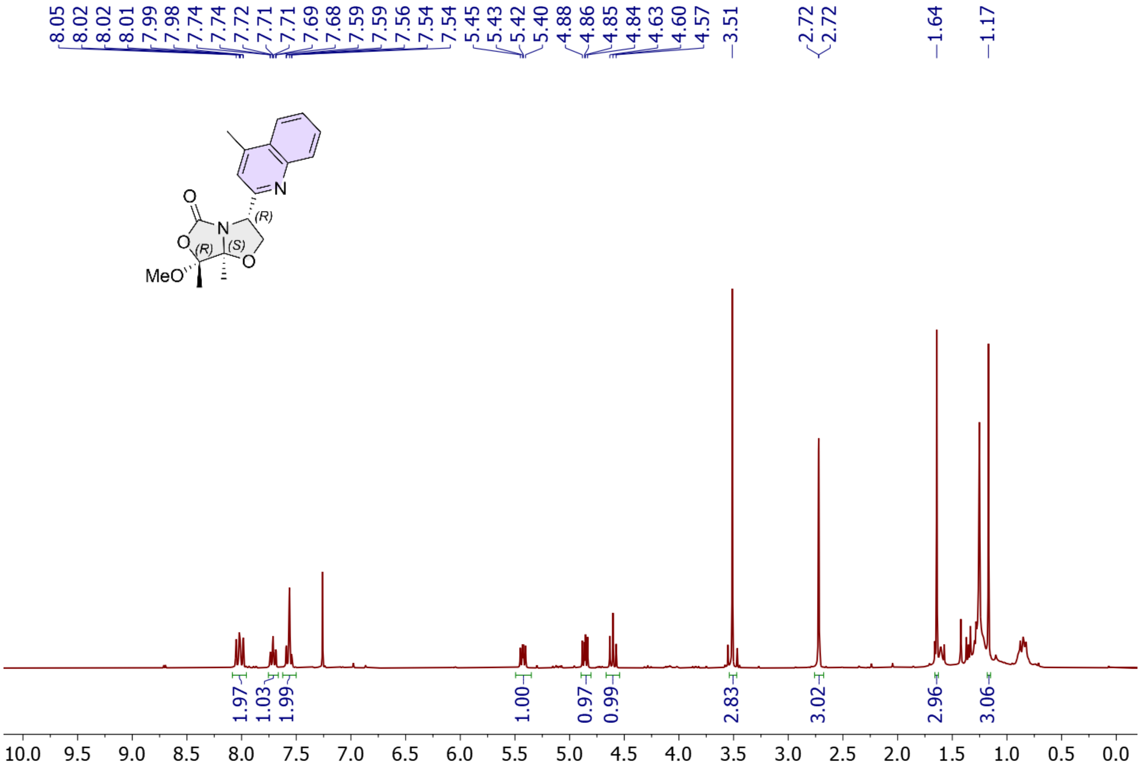

^{13}C {^{1}H} NMR in $CDCl_3$ (100 MHz) compound 16

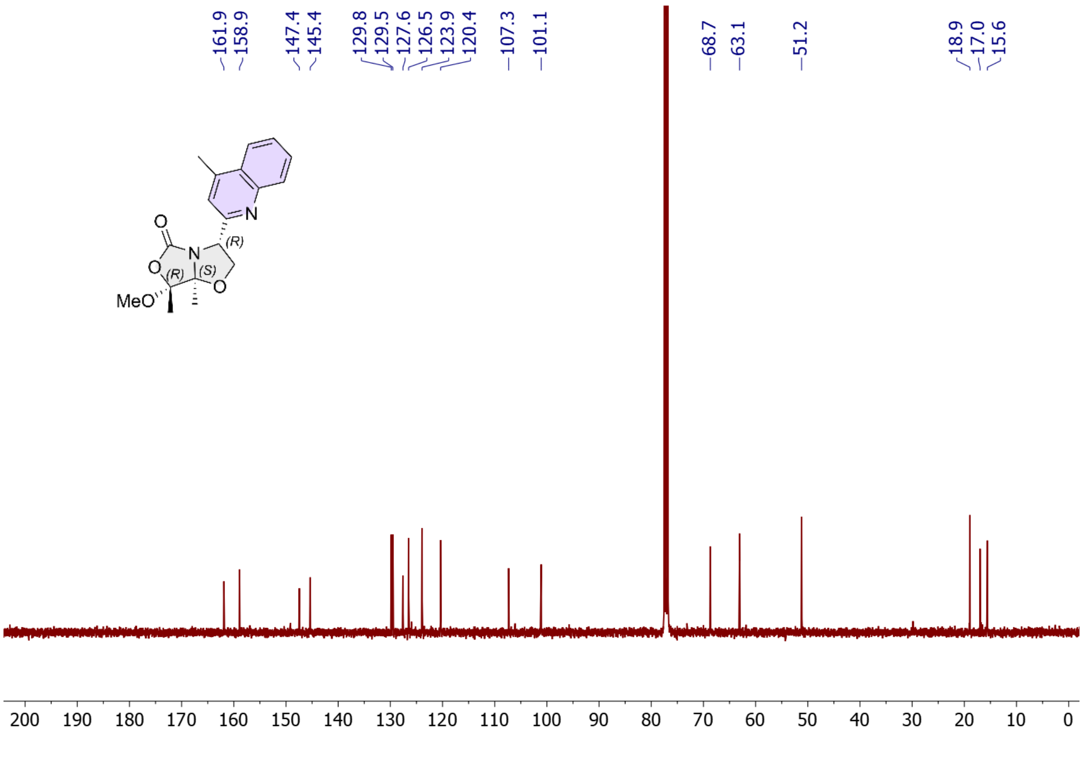

COSY in $CDCl_3$ (400 MHz) compound 16

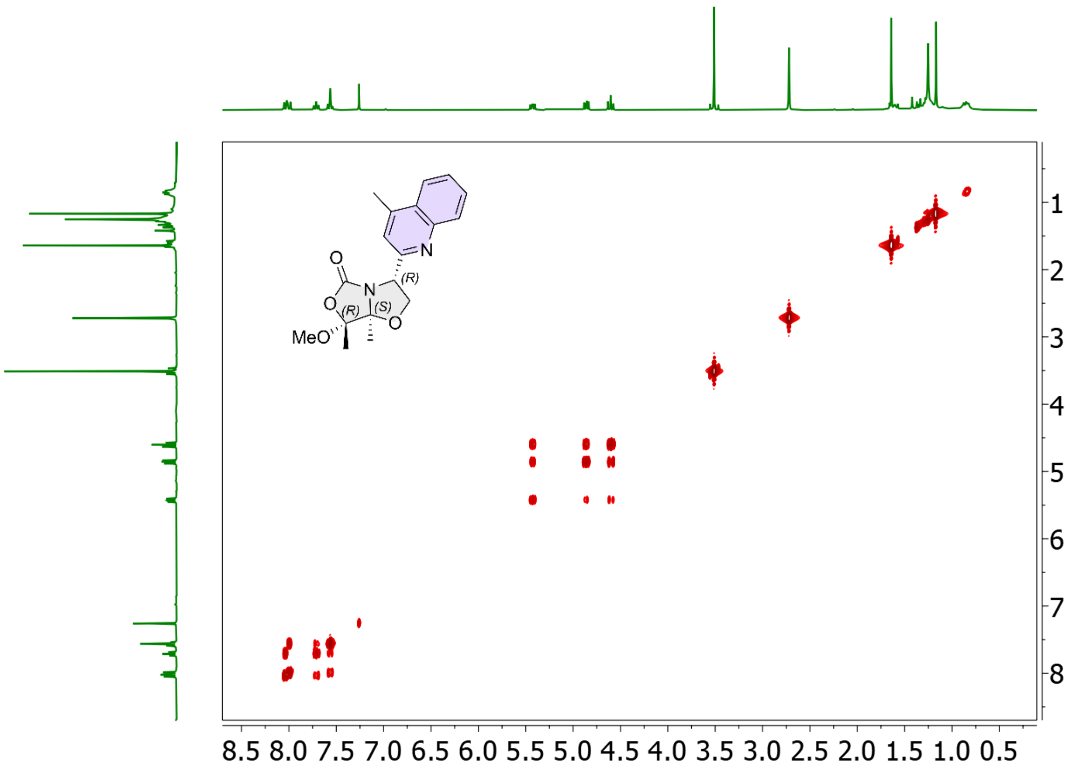

HSQC in $CDCl_3$ (400 MHz) compound 16

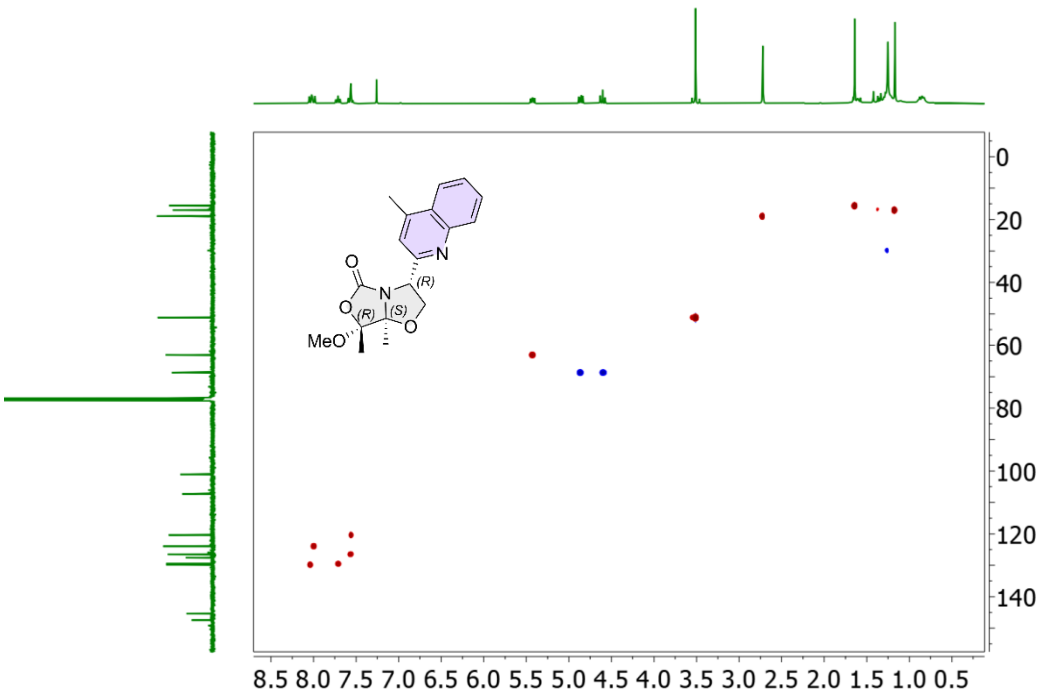

^{1}H NMR in $CDCl_3$ (400 MHz) compound 17

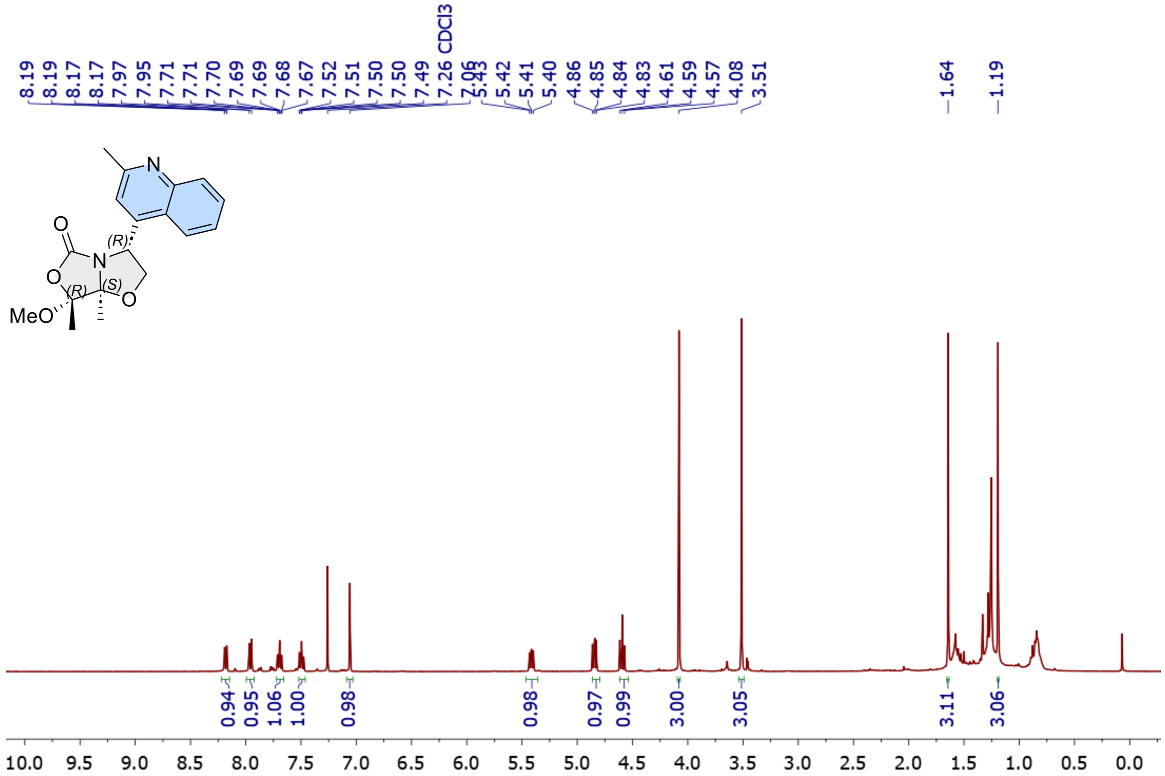

^{13}C {^{1}H} NMR in $CDCl_3$ (100 MHz) compound 17

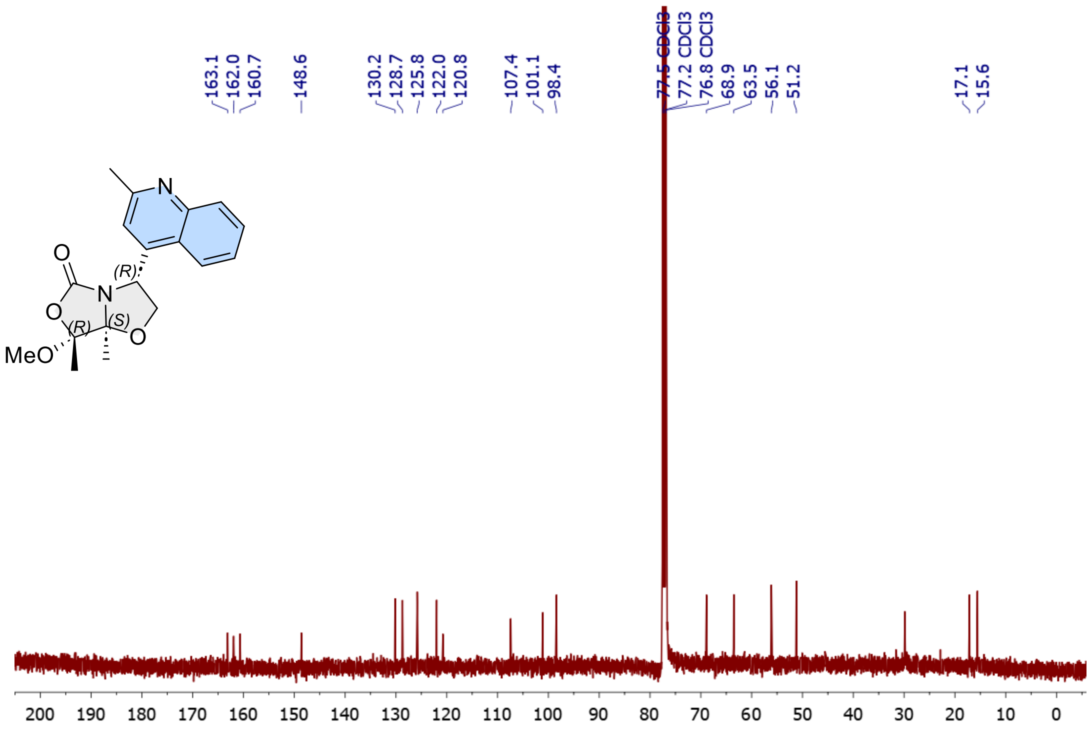

COSY in $CDCl_3$ (400 MHz) compound 17

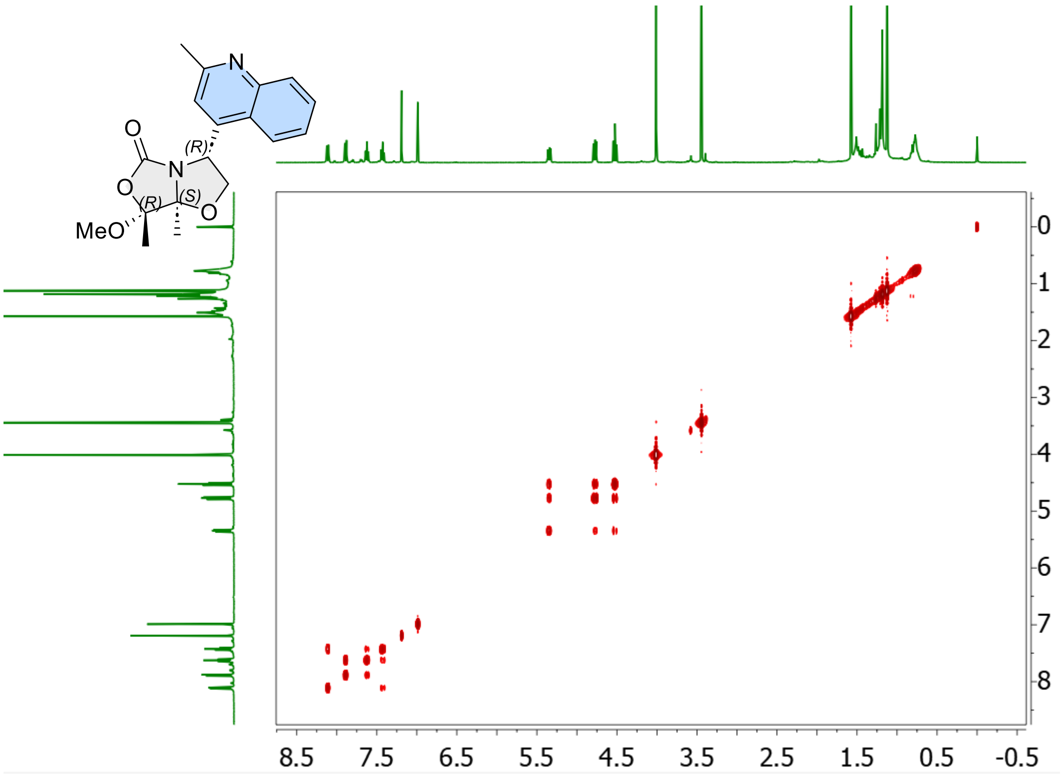

HSQC in $CDCl_3$ (400 MHz) compound 17

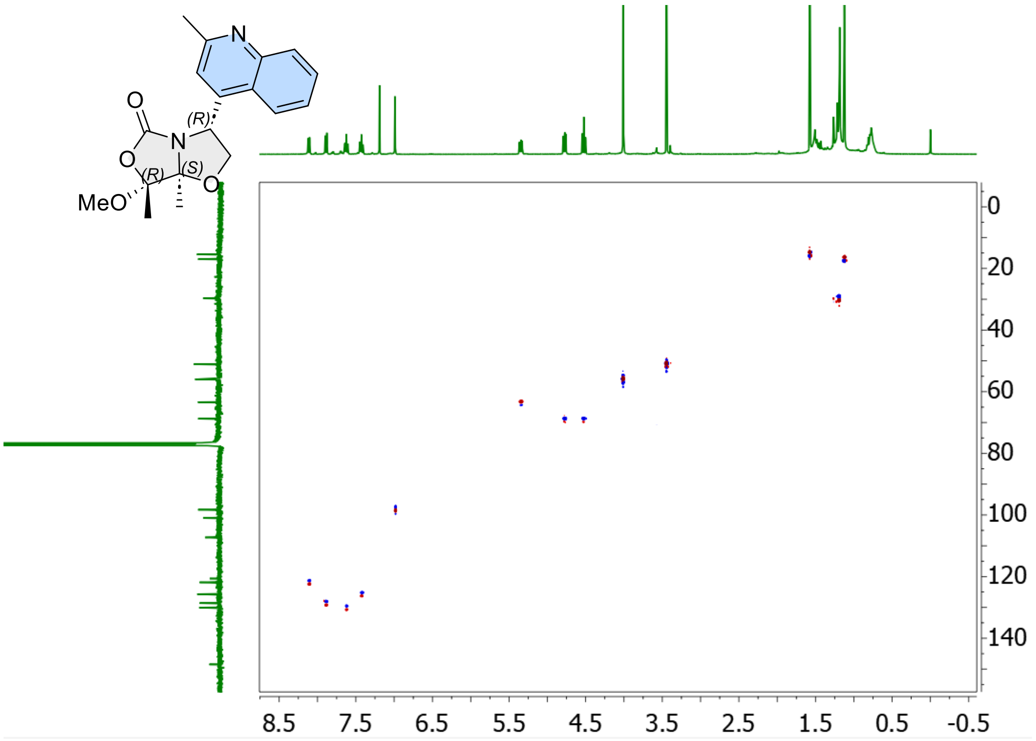

NOESY in $CDCl_3$ (400 MHz) compound 17

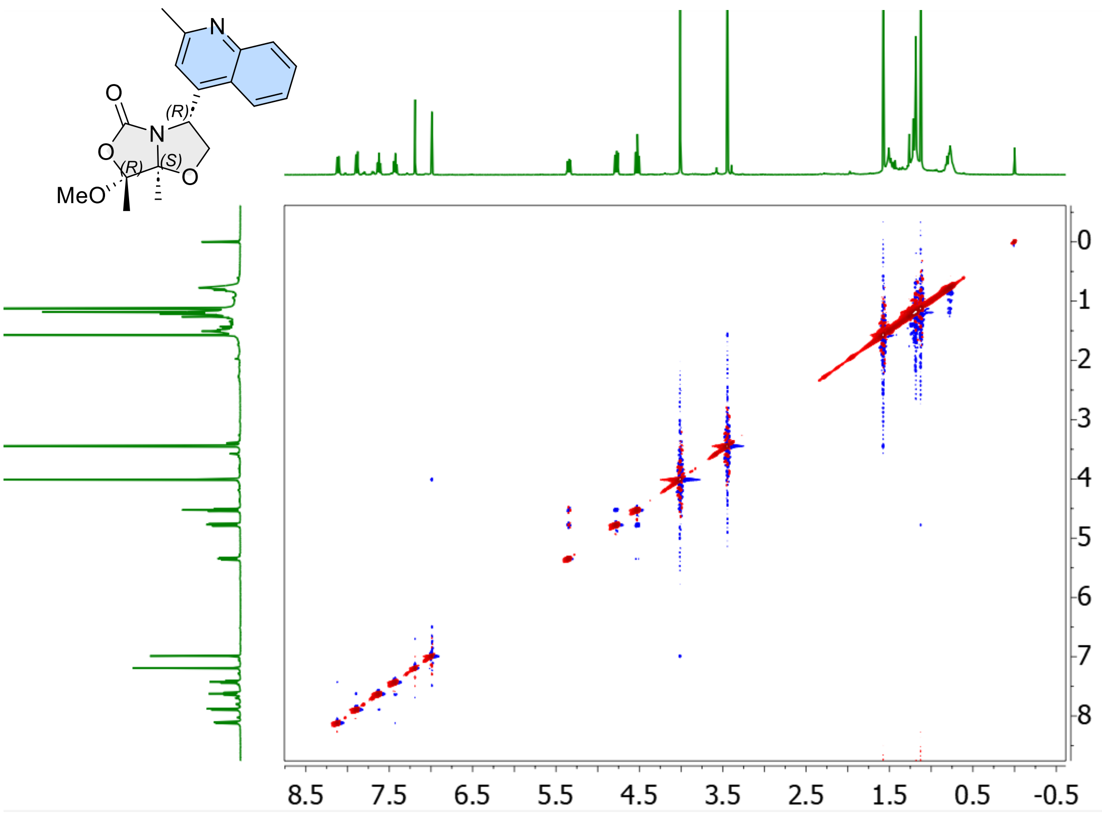

^{1}H NMR in $CDCl_3$ (400 MHz) compound 18

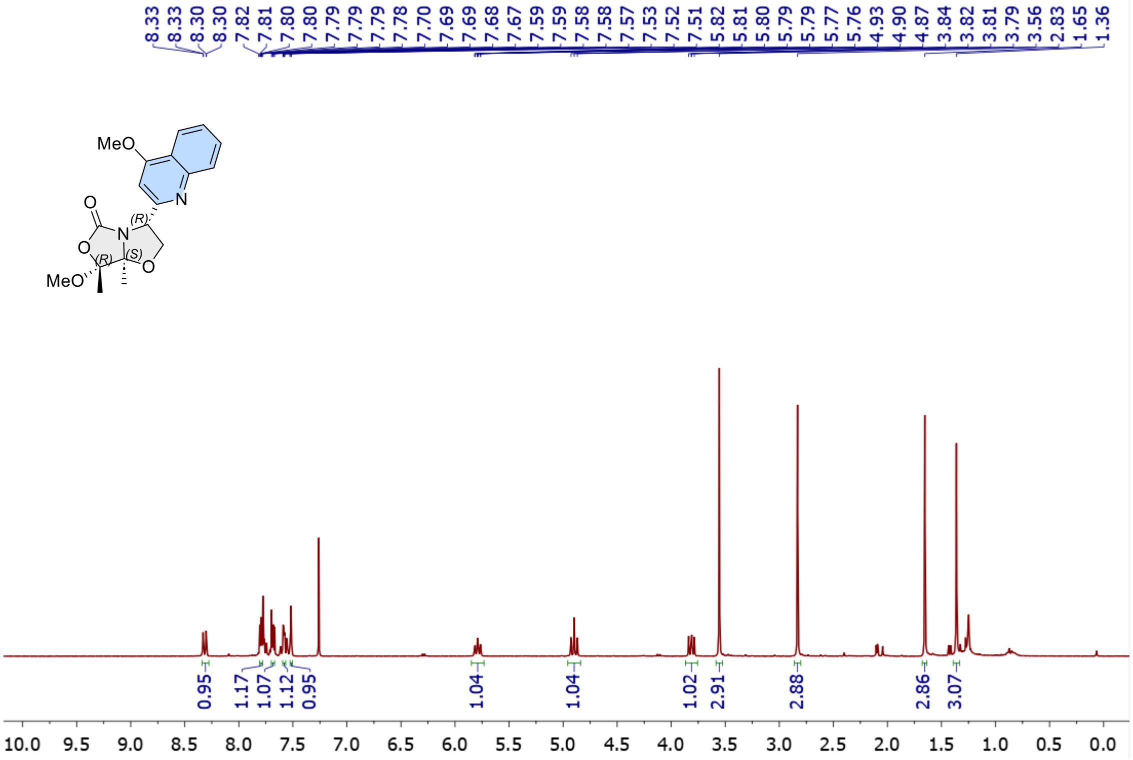

^{13}C {^{1}H} NMR in $CDCl_3$ (100 MHz) compound 18

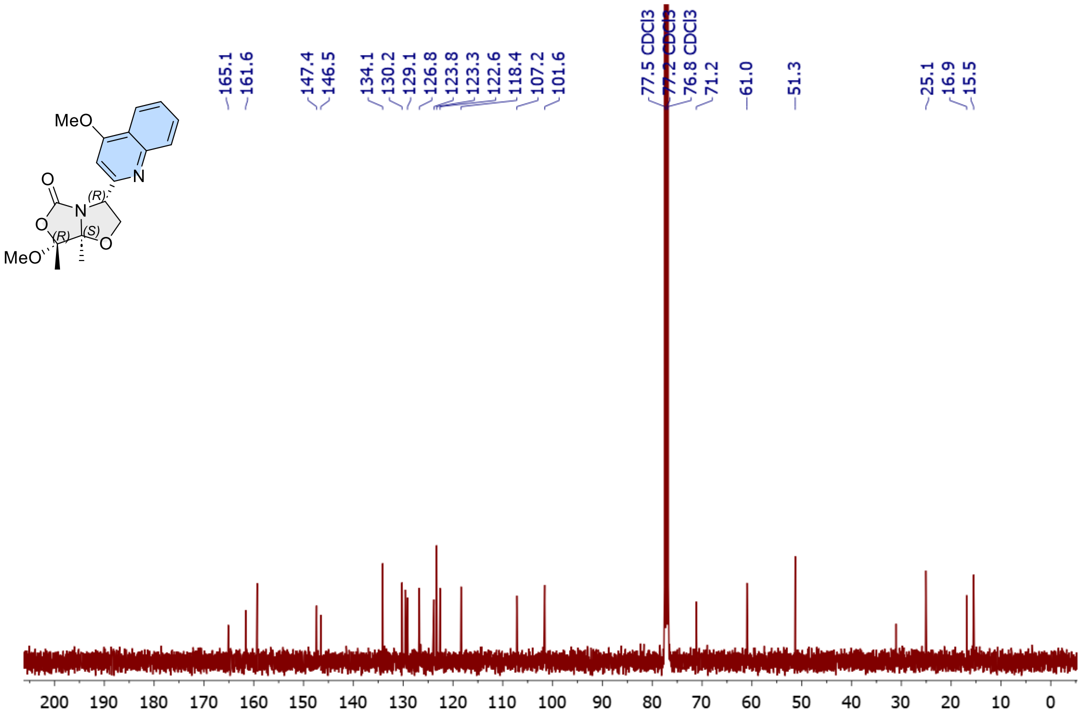

COSY in $CDCl_3$ (400 MHz) compound 18

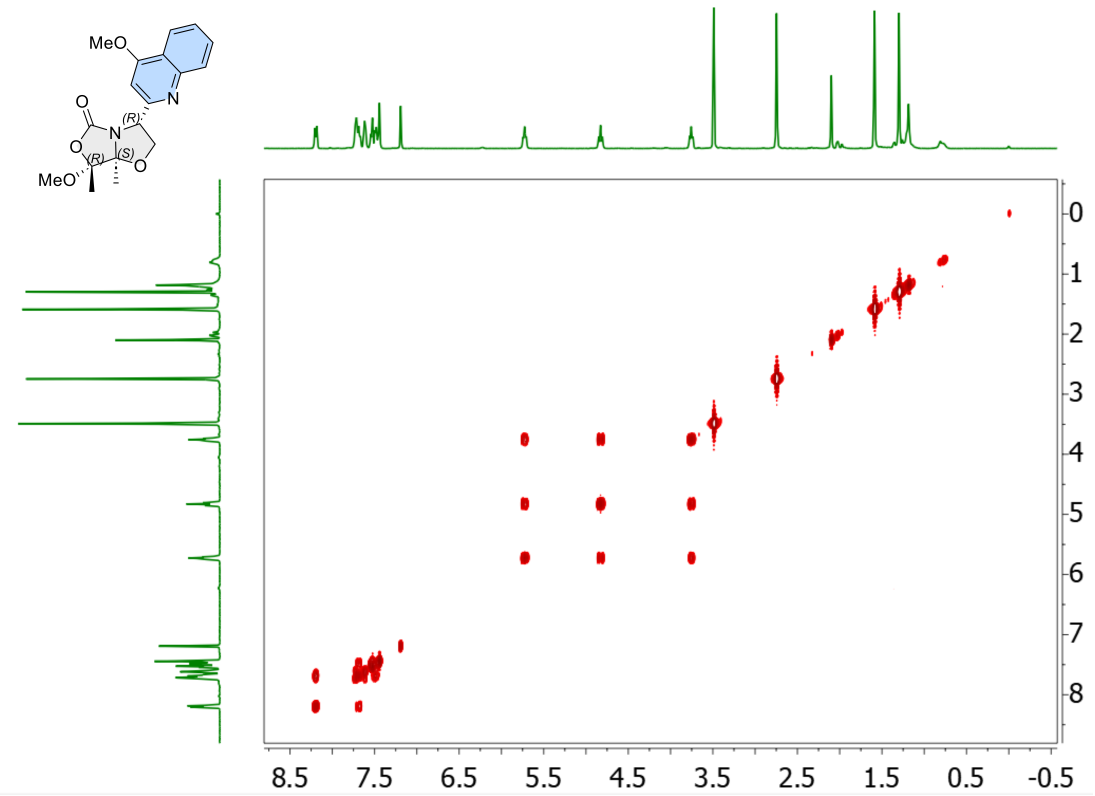

HSQC in $CDCl_3$ (400 MHz) compound 18

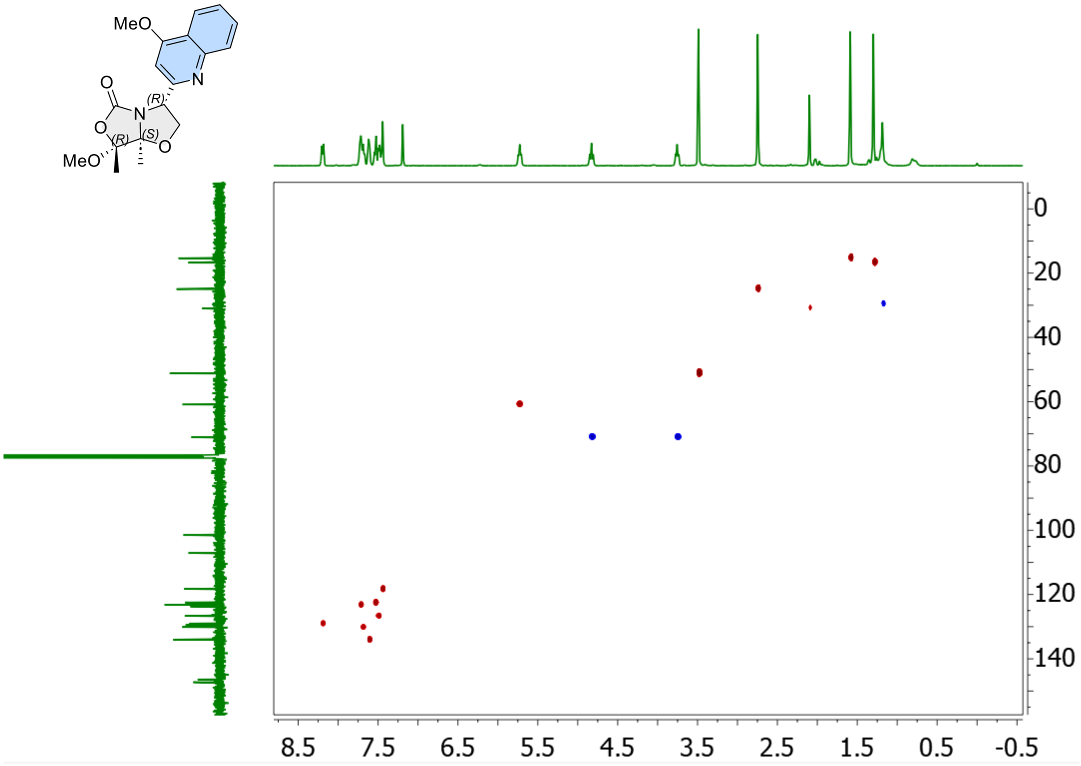

NOESY in $CDCl_3$ (400 MHz) compound 18

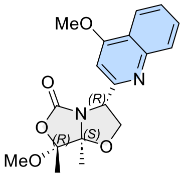

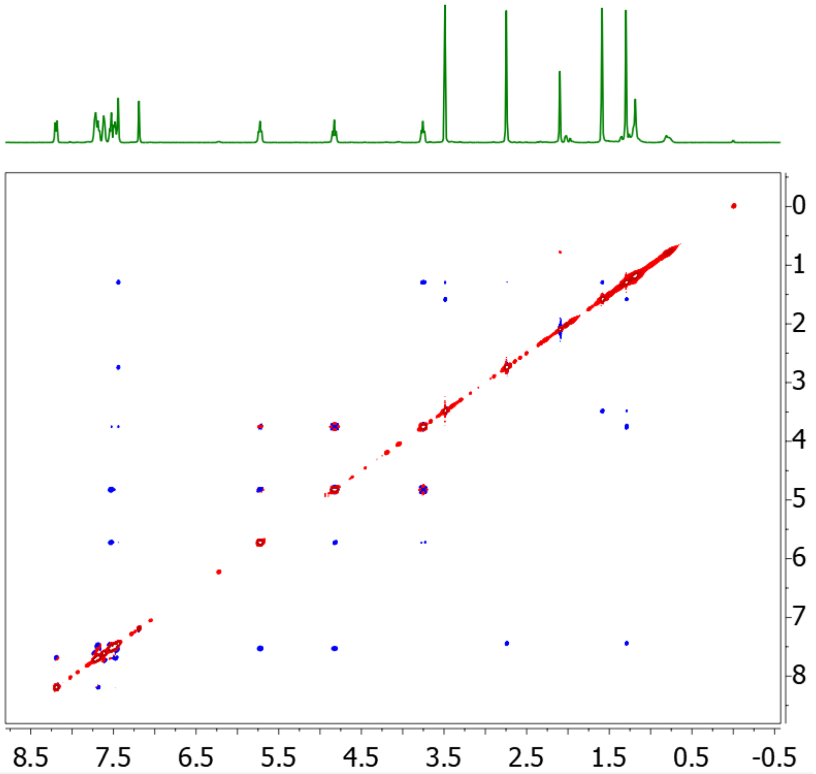

^{1}H NMR in $CDCl_3$ (400 MHz) compound 19a

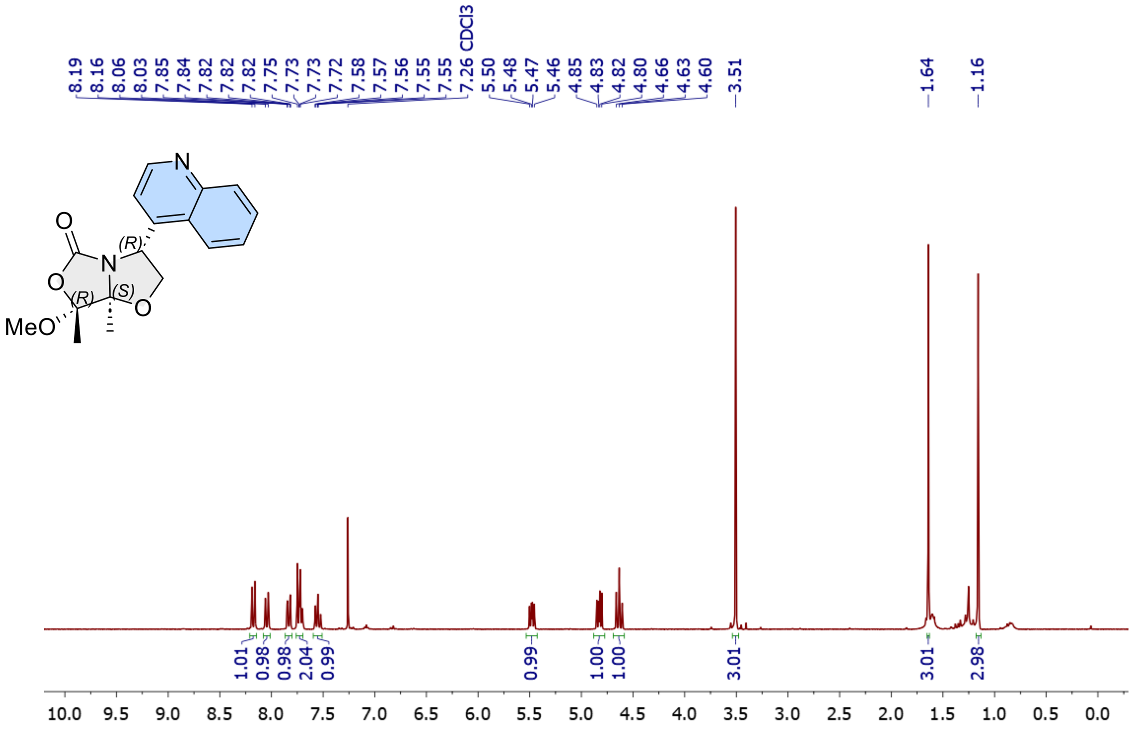

$^{13}C\ \{^{1}H\}$ NMR in $CDCl_3$ (100 MHz) compound 19a

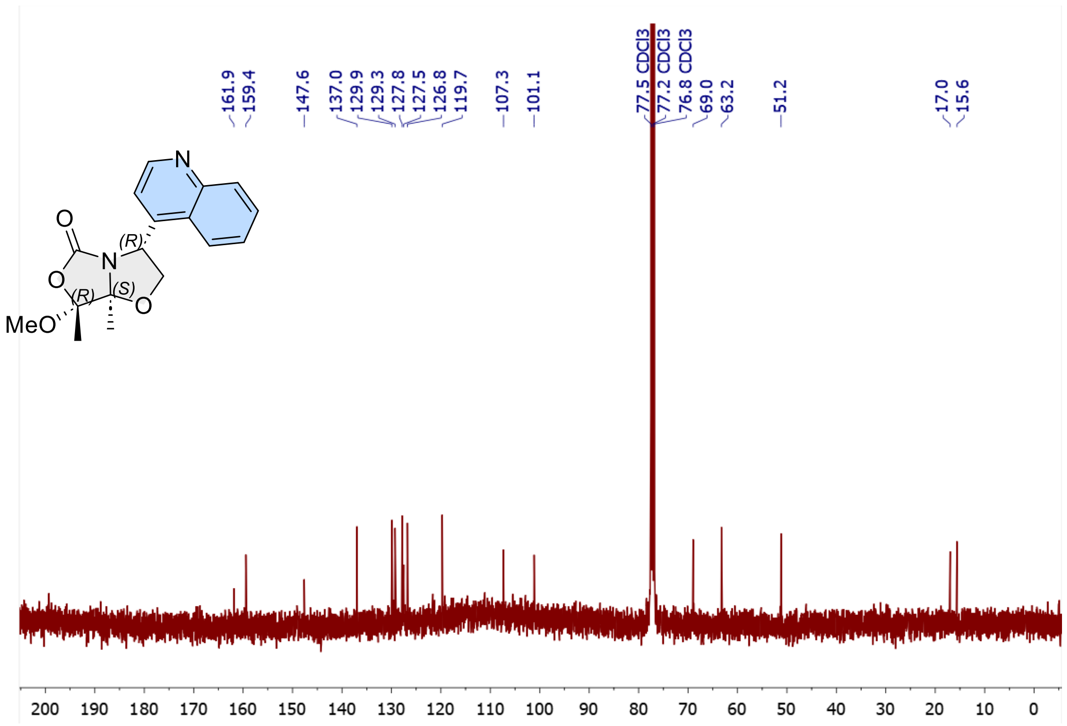

COSY in $CDCl_3$ (400 MHz) compound 19a

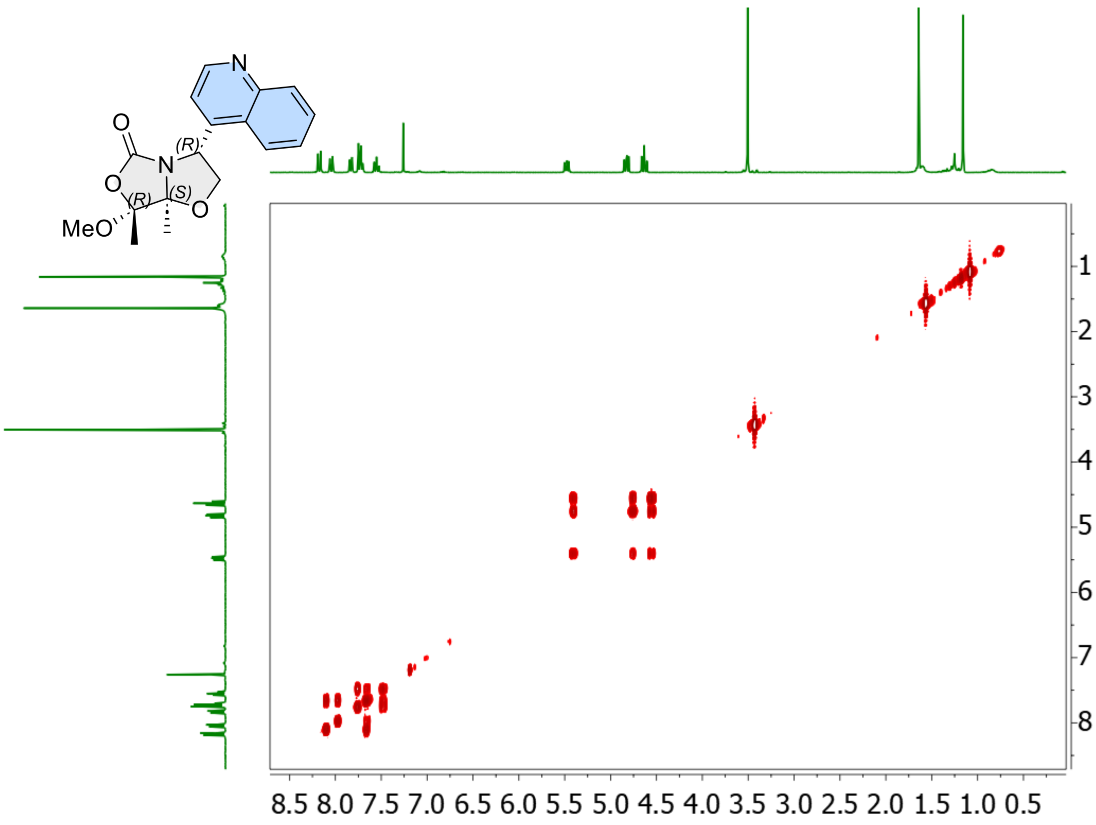

HSQC in $CDCl_3$ (400 MHz) compound 19a

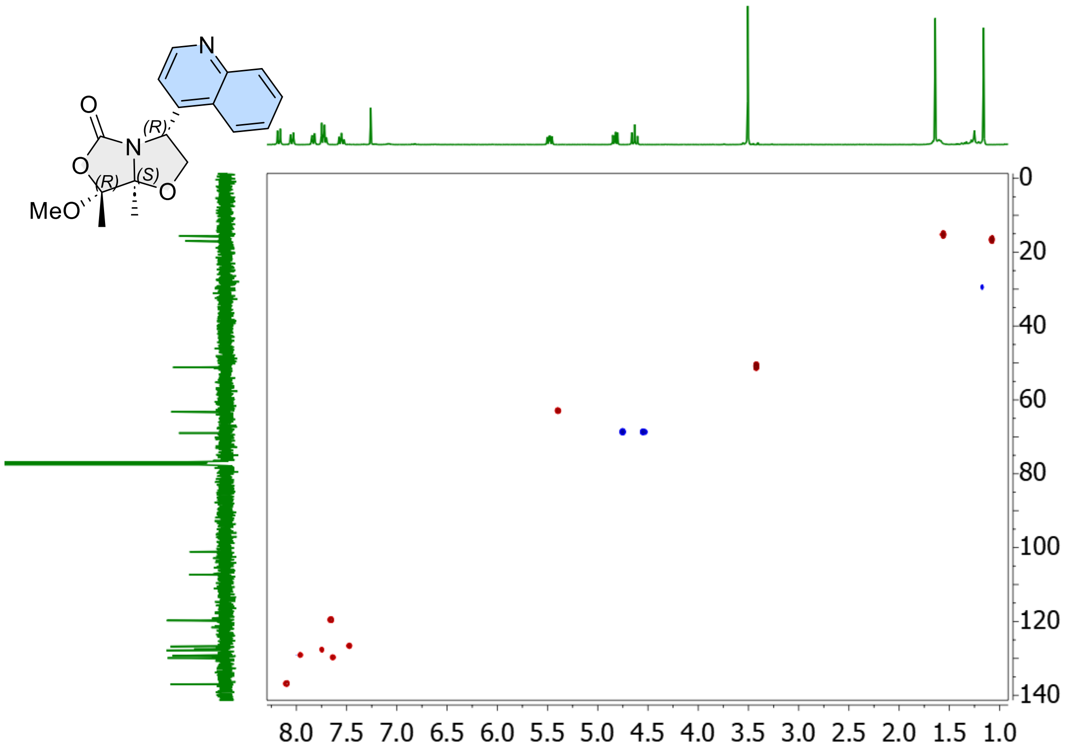

NOESY in $CDCl_3$ (400 MHz) compound 19a

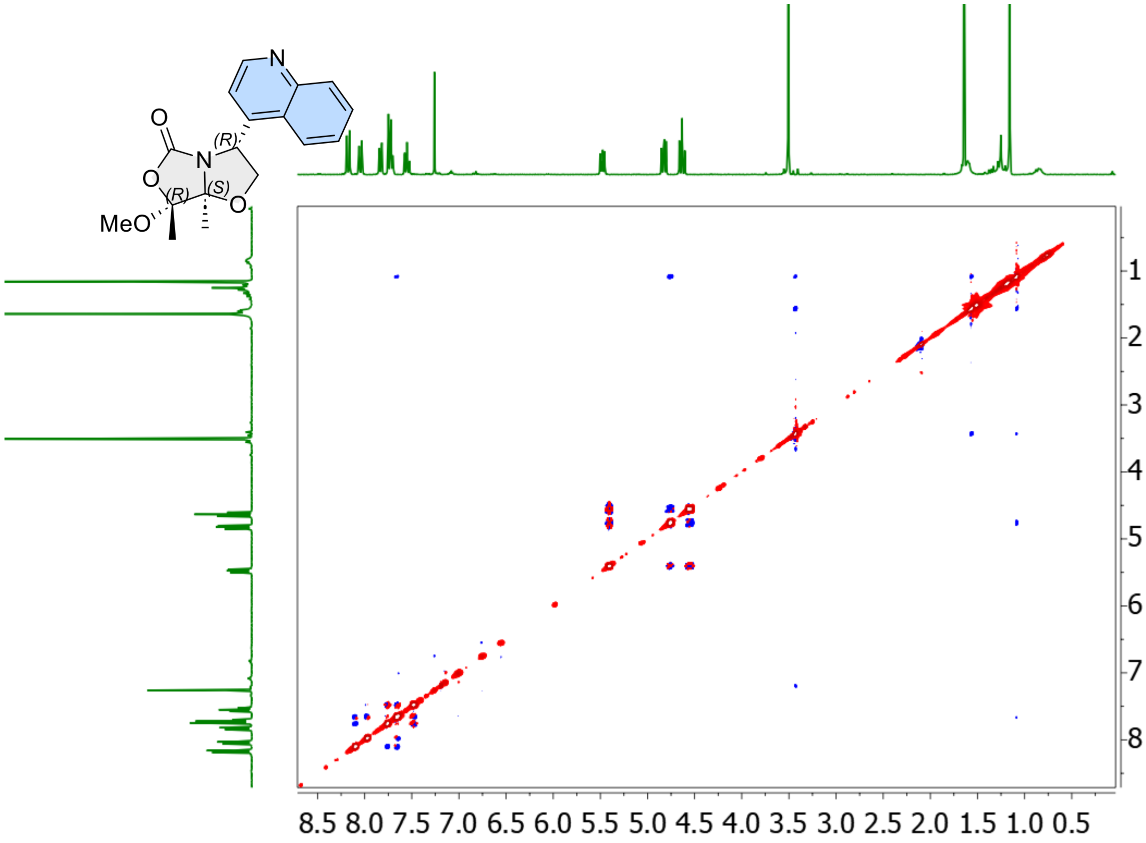

^{1}H NMR in $CDCl_3$ (400 MHz) compound 19b

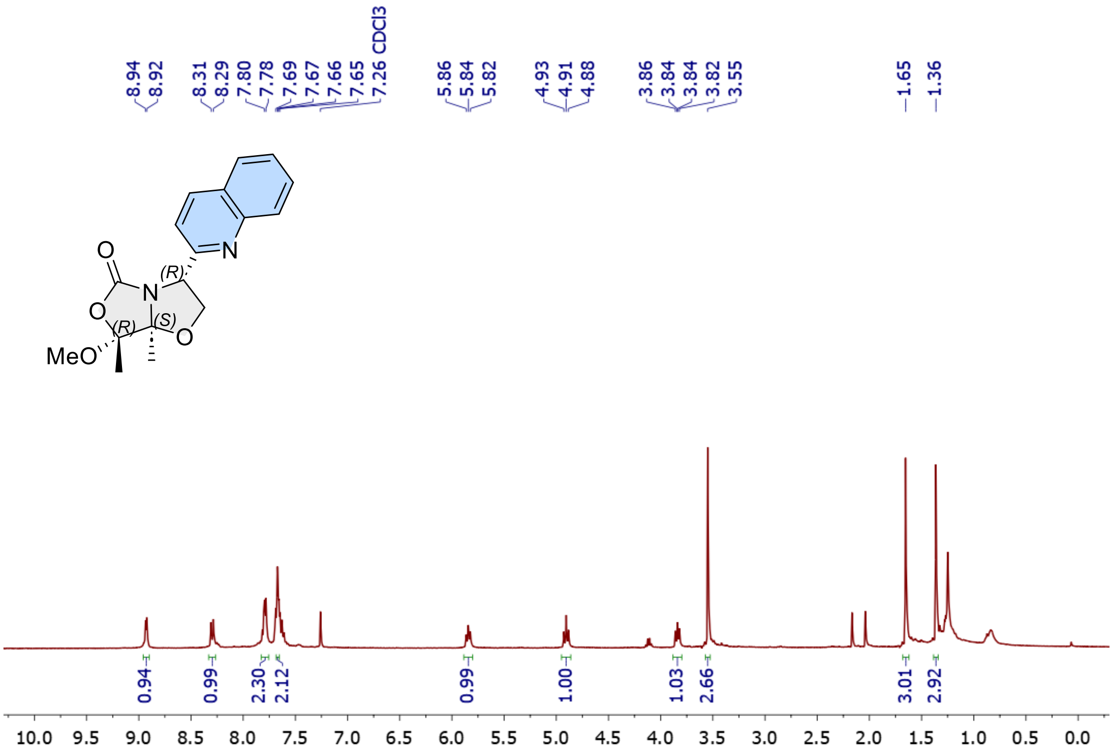

^{13}C {^{1}H} NMR in $CDCl_3$ (100 MHz) compound 19b

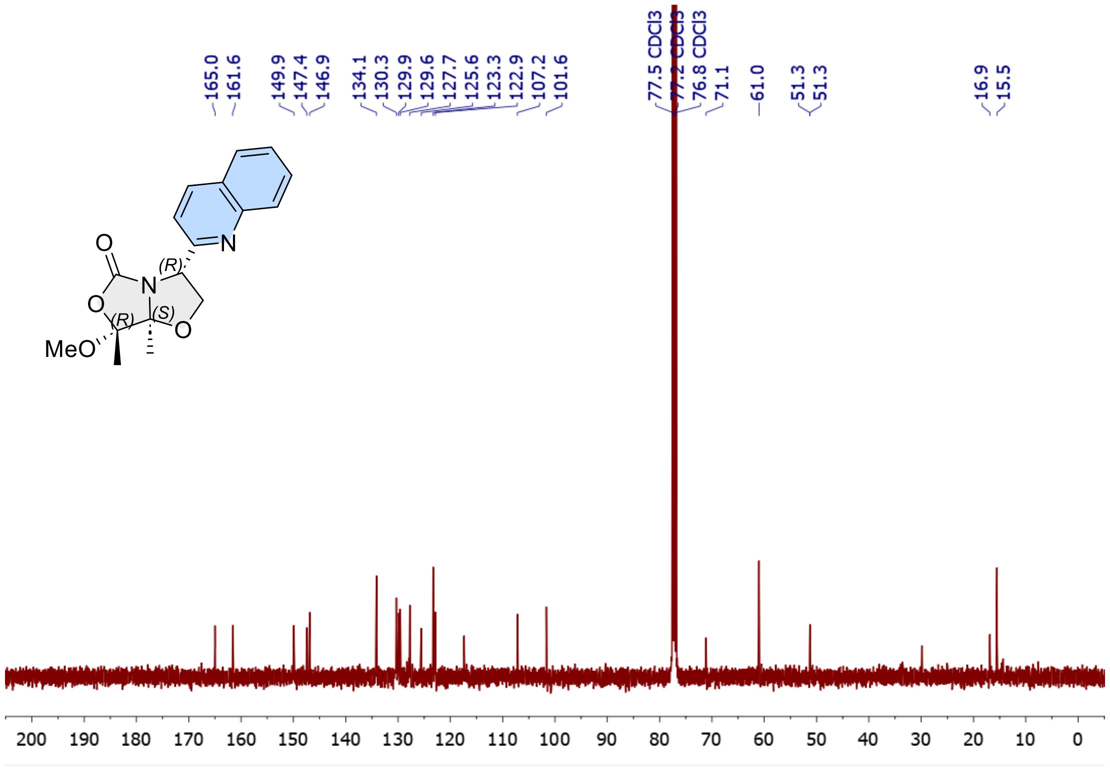

COSY in $CDCl_3$ (400 MHz) compound 19b

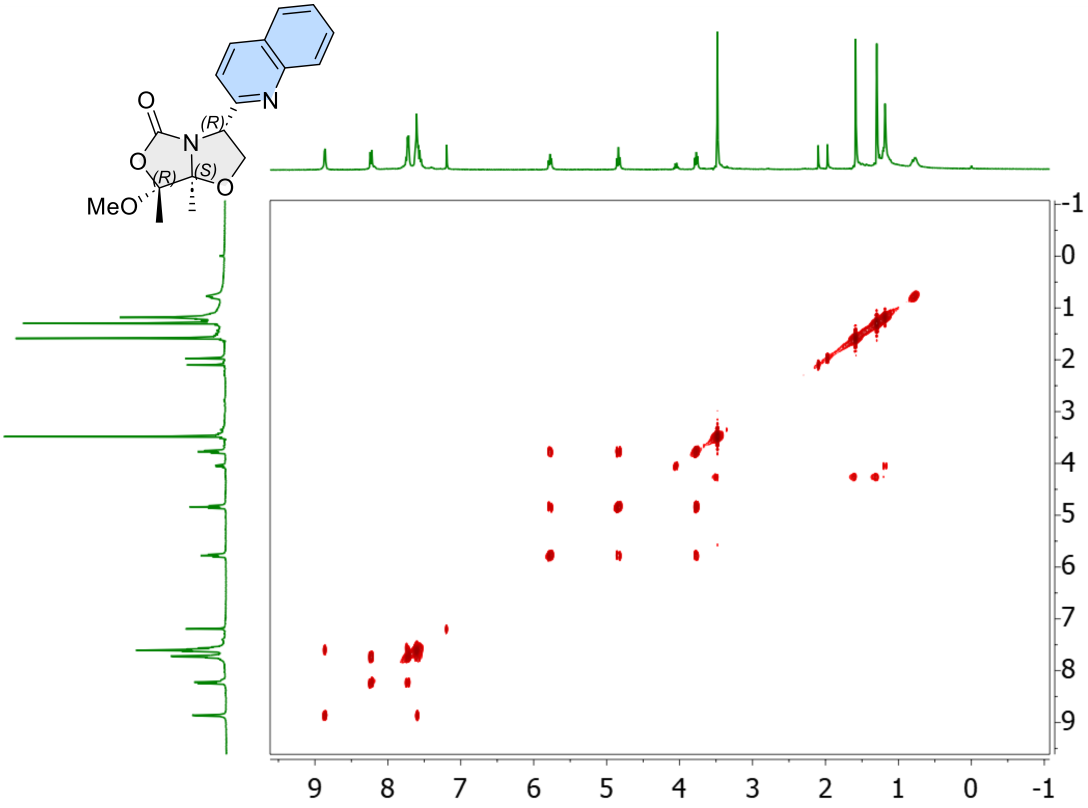

HSQC in $CDCl_3$ (400 MHz) compound 19b

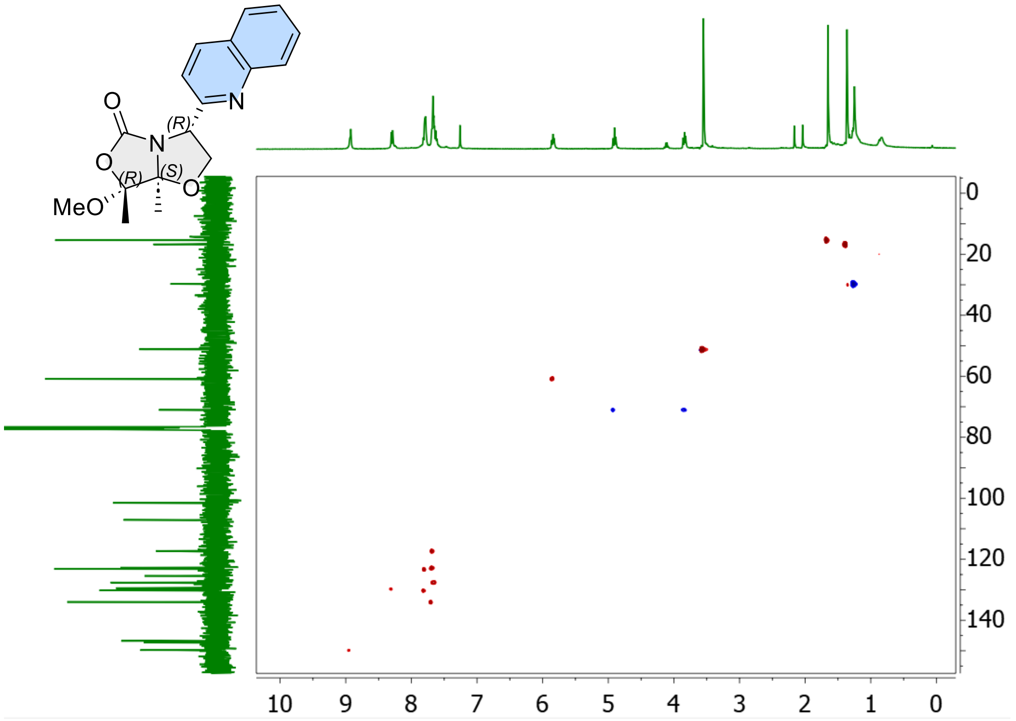

NOESY in $CDCl_3$ (400 MHz) compound 19b

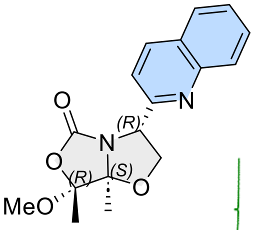

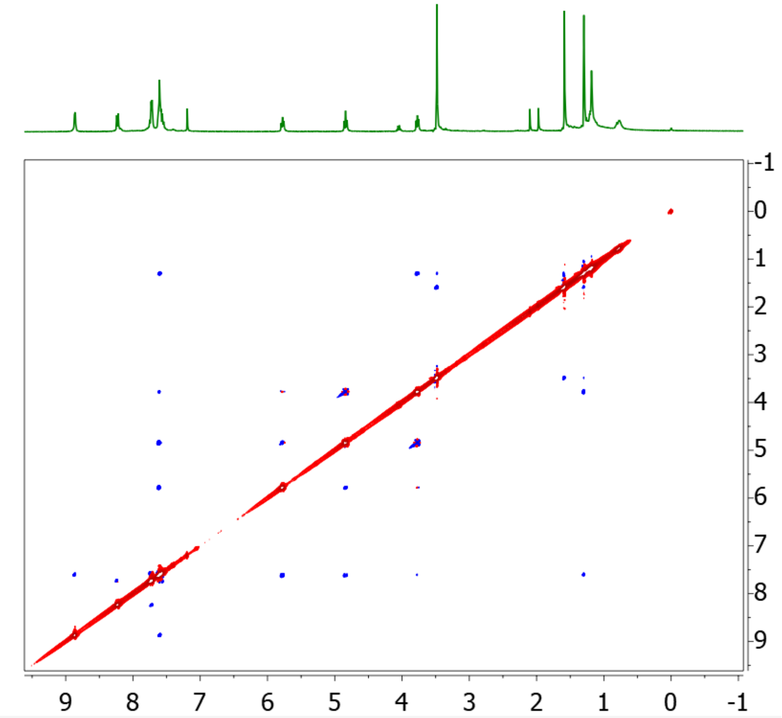

^{1}H NMR in $CDCl_3$ (400 MHz) compound 19c

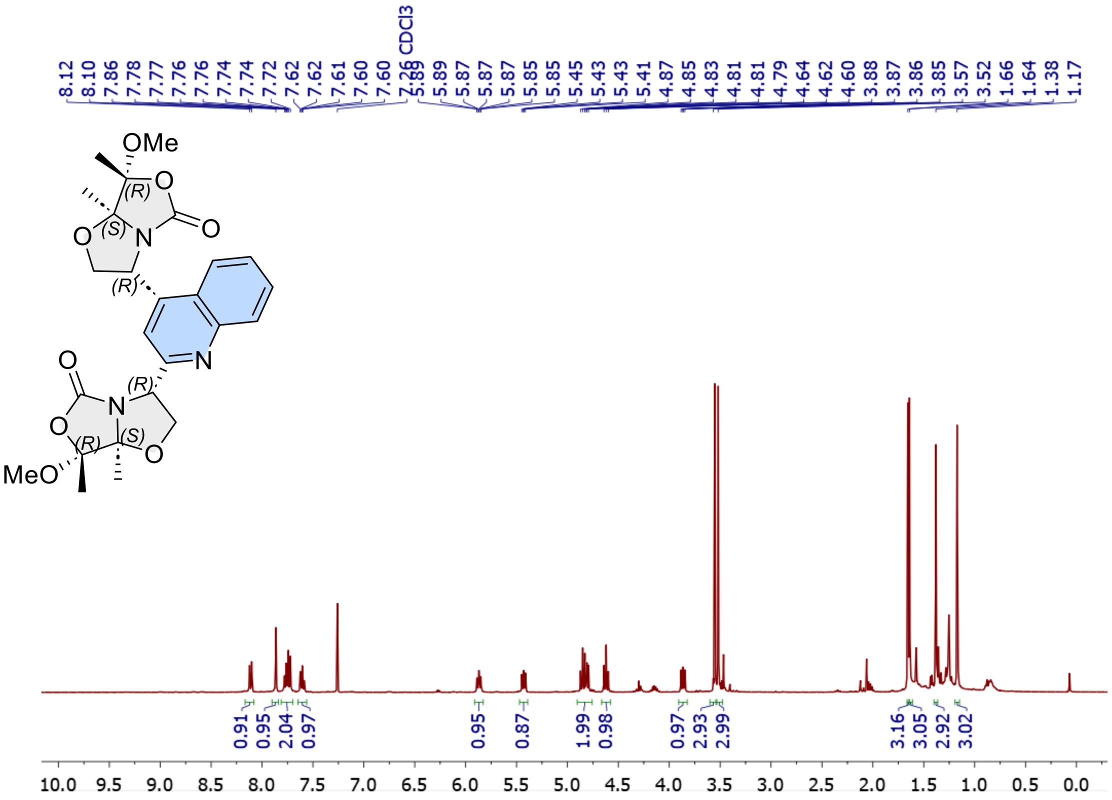

^{13}C {^{1}H} NMR in $CDCl_3$ (100 MHz) compound 19c

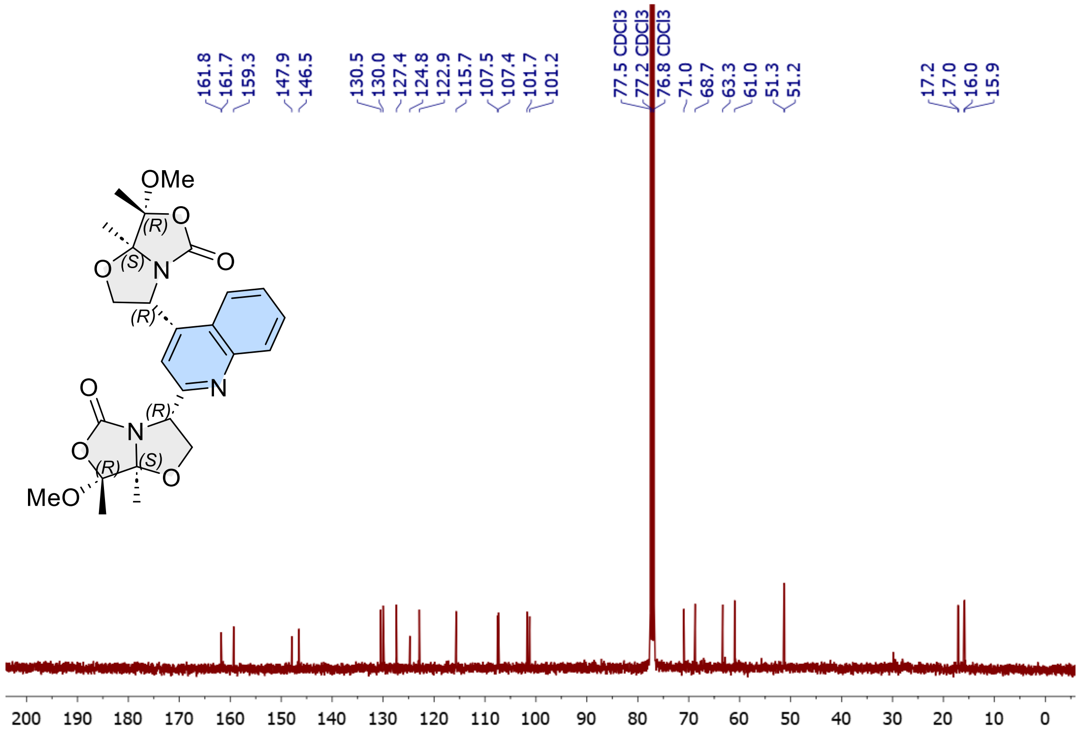

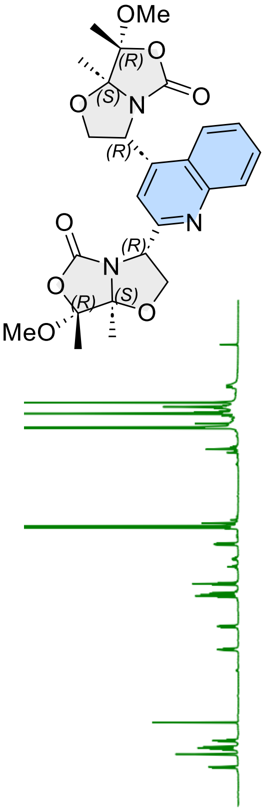

COSY in $CDCl_3$ (400 MHz) compound 19c

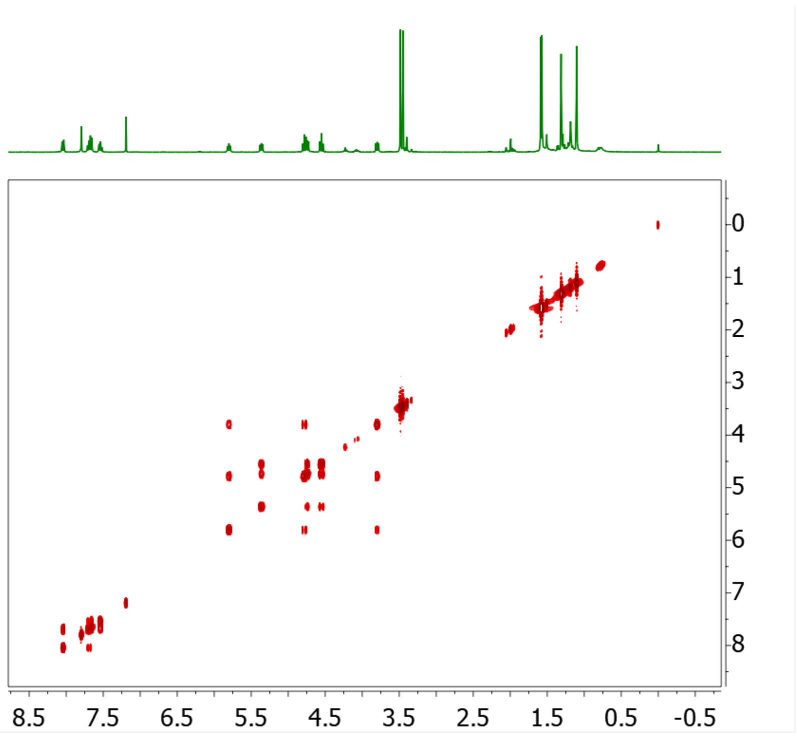

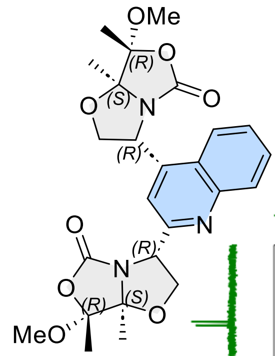

HSQC in $CDCl_3$ (400 MHz) compound 19c

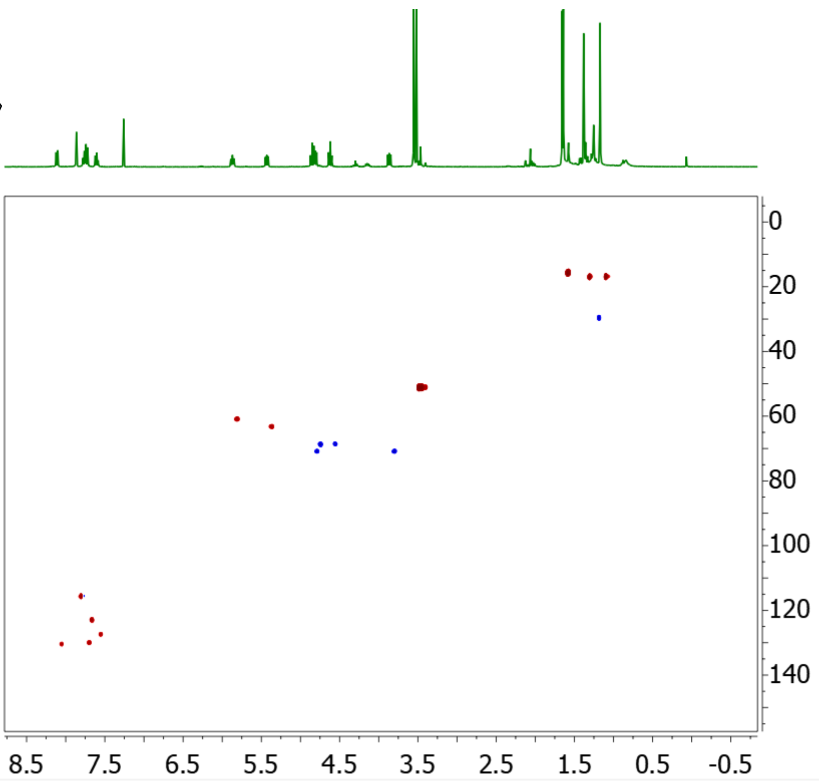

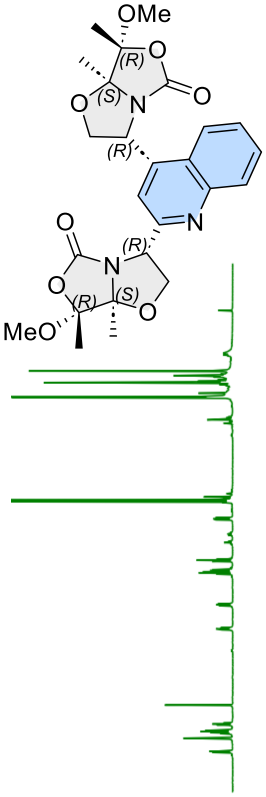

NOESY in $CDCl_3$ (400 MHz) compound 19c

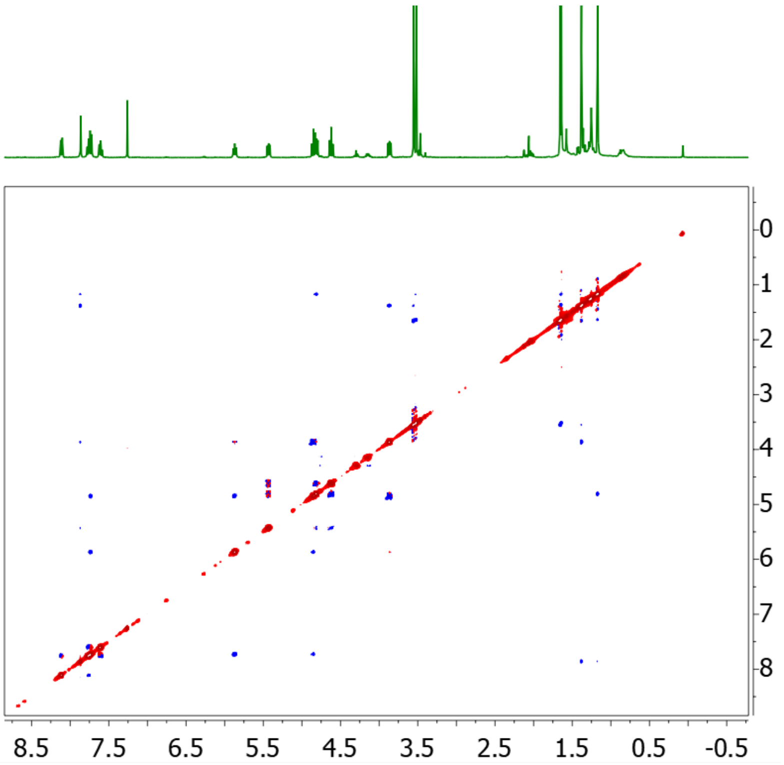

^1H NMR in $CDCl_3$ (400 MHz) compound 20

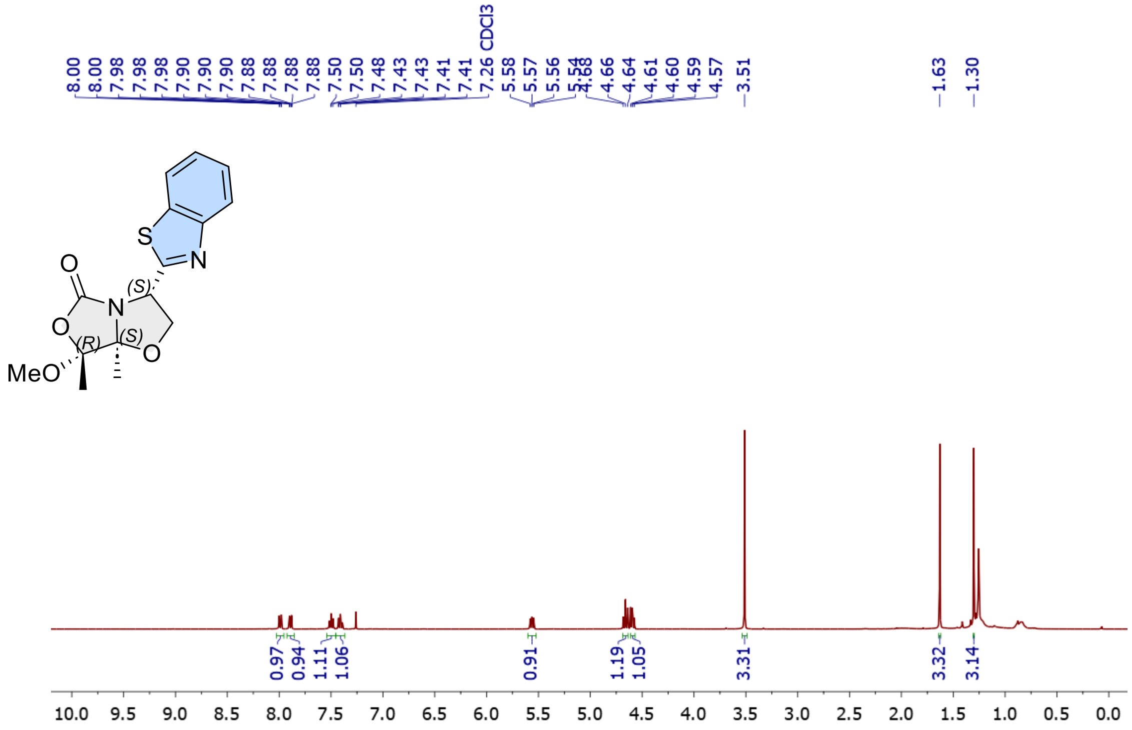

$^{13}C\{^1H\}$ NMR in $CDCl_3$ (100 MHz) compound 20

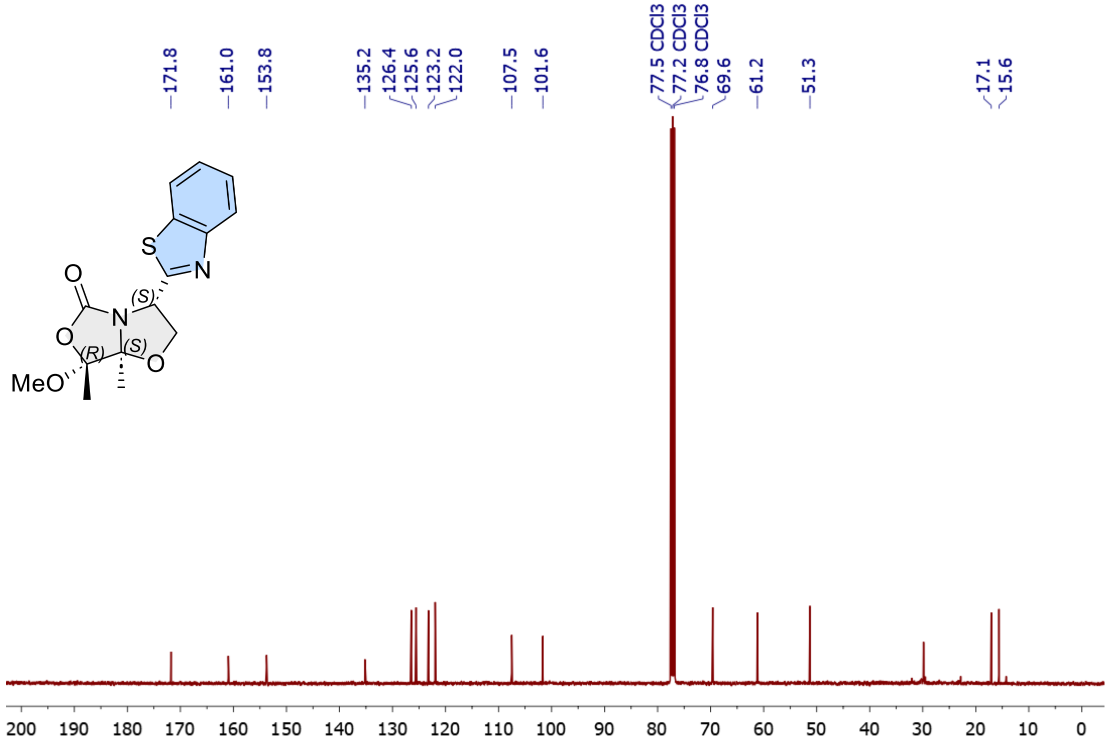

COSY in $CDCl_3$ (400 MHz) compound 20

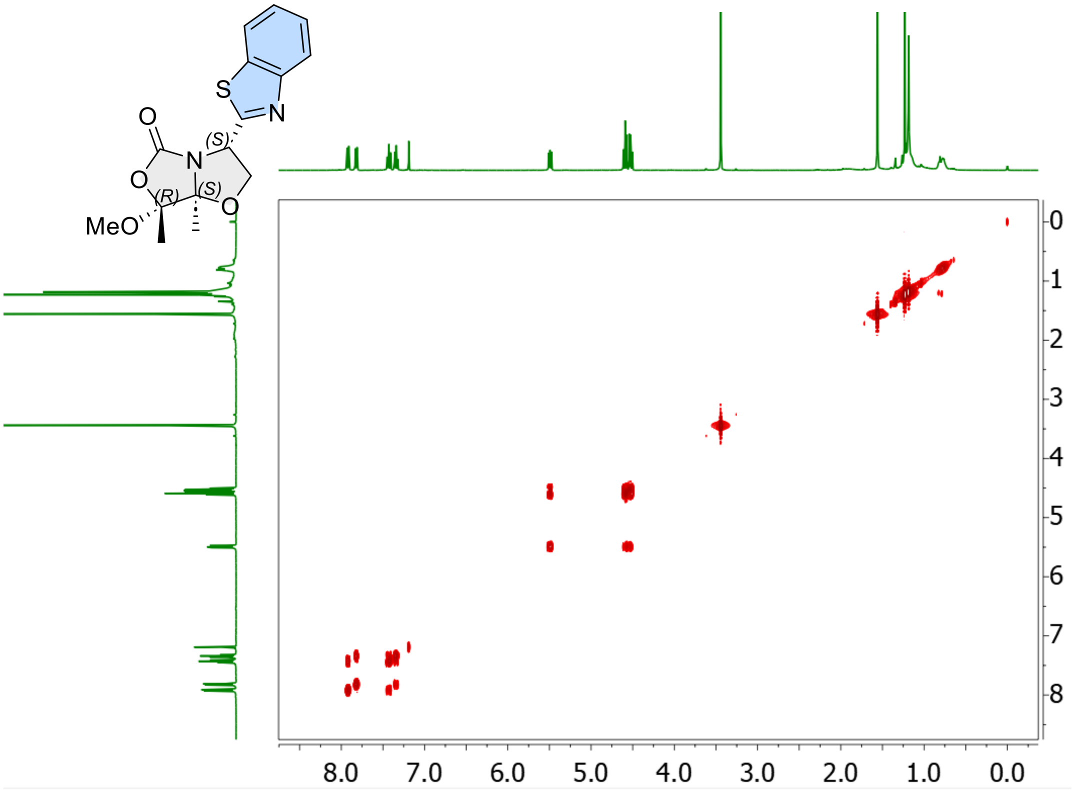

HSQC in $CDCl_3$ (400 MHz) compound 20

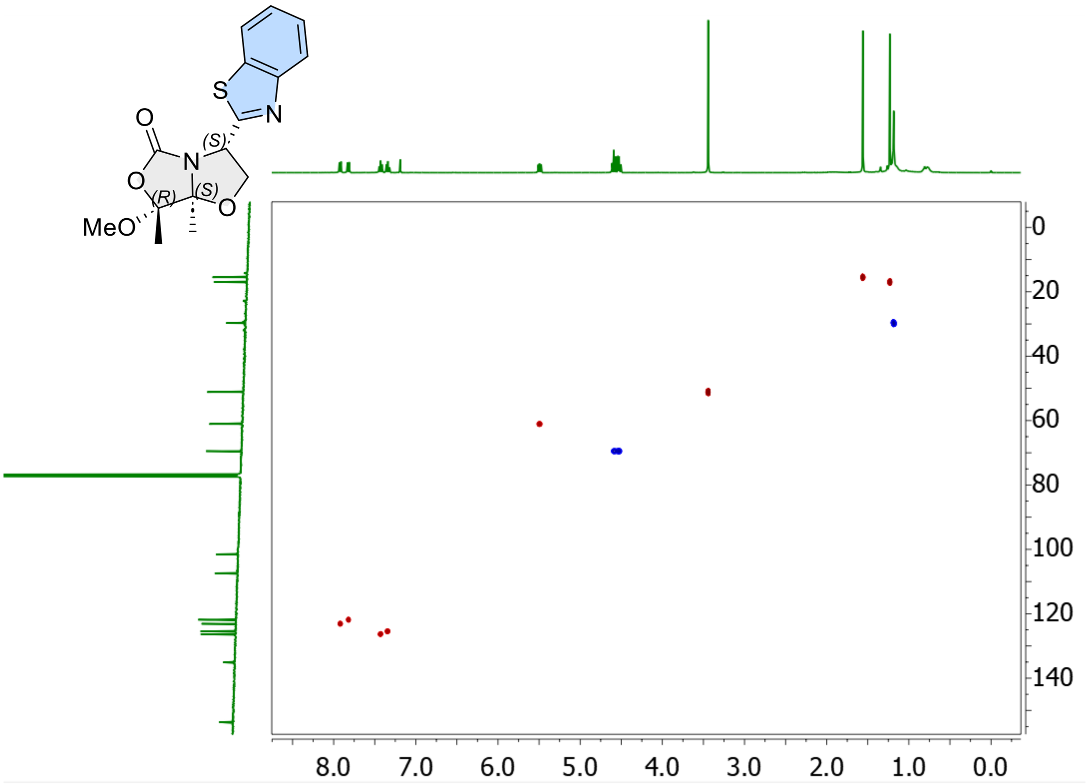

NOESY in $CDCl_3$ (400 MHz) compound 20

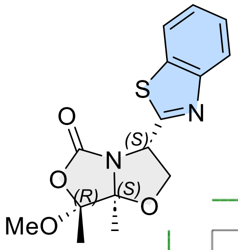

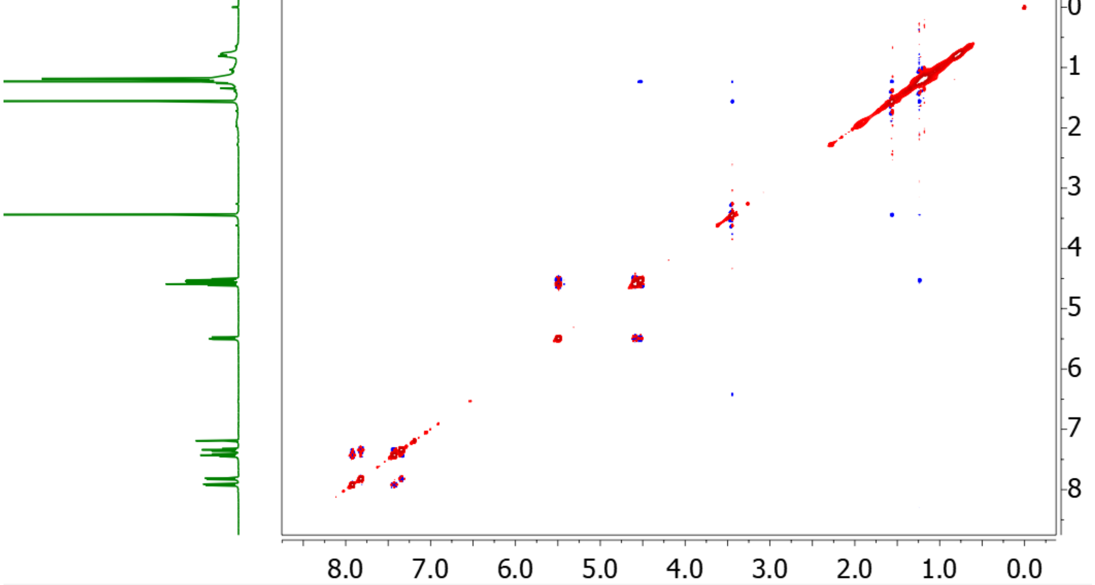

^1H NMR in $CDCl_3$ (400 MHz) compound 21

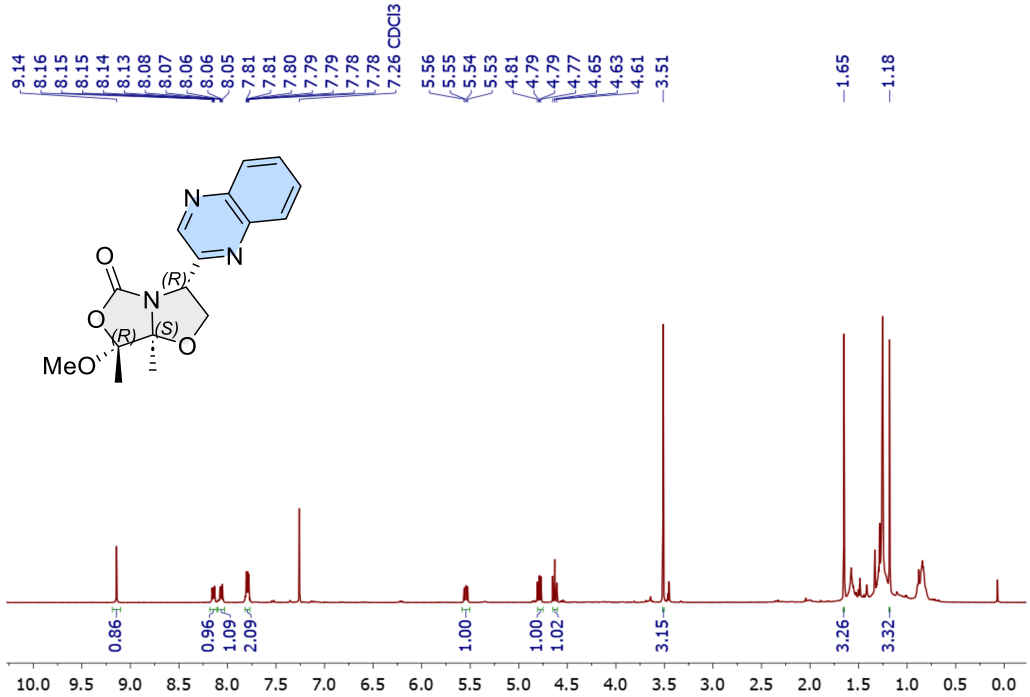

^{13}C {^1H} NMR in $CDCl_3$ (100 MHz) compound 21

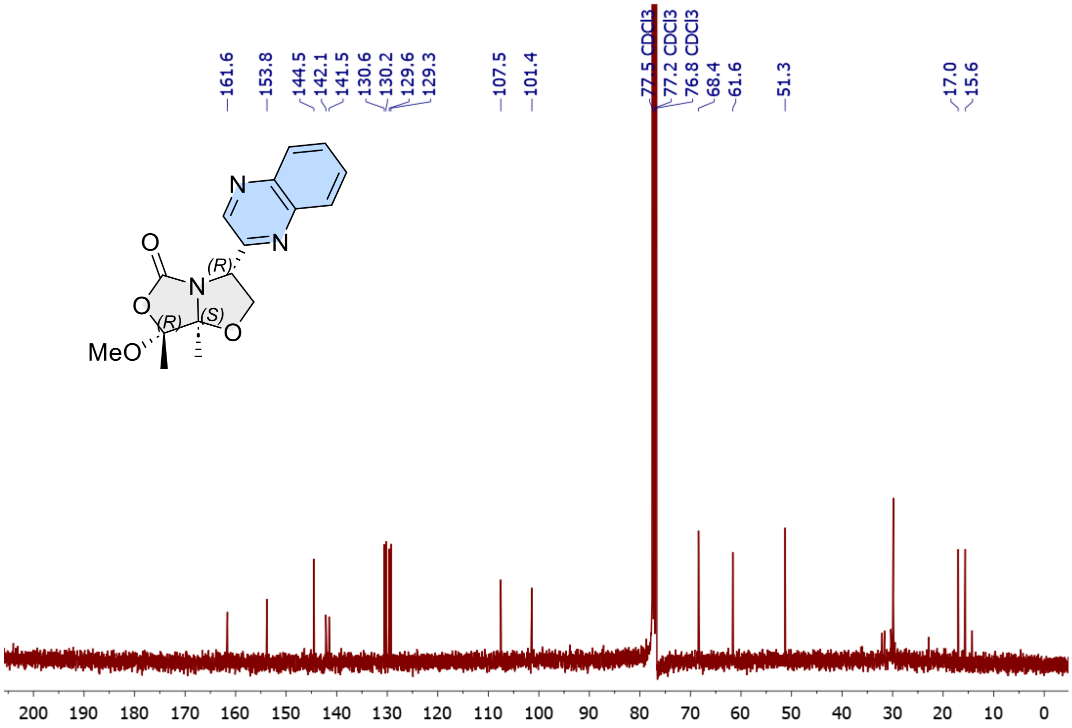

COSY in $CDCl_3$ (400 MHz) compound 21

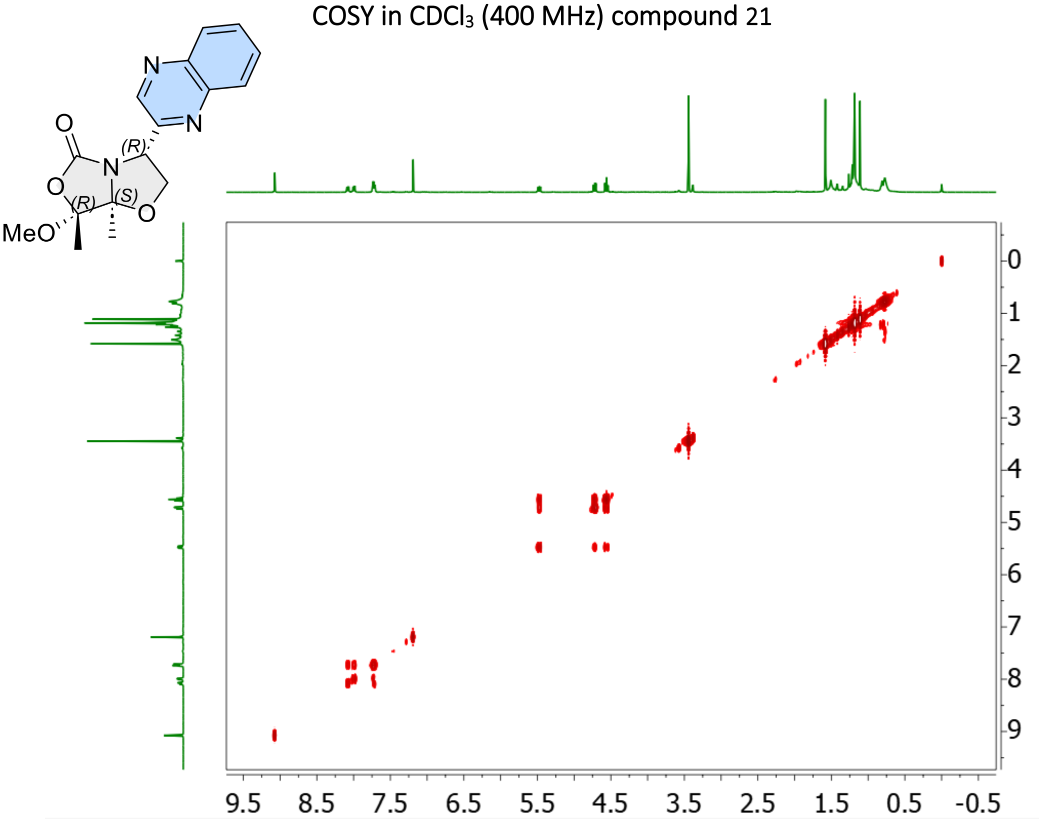

HSQC in $CDCl_3$ (400 MHz) compound 21

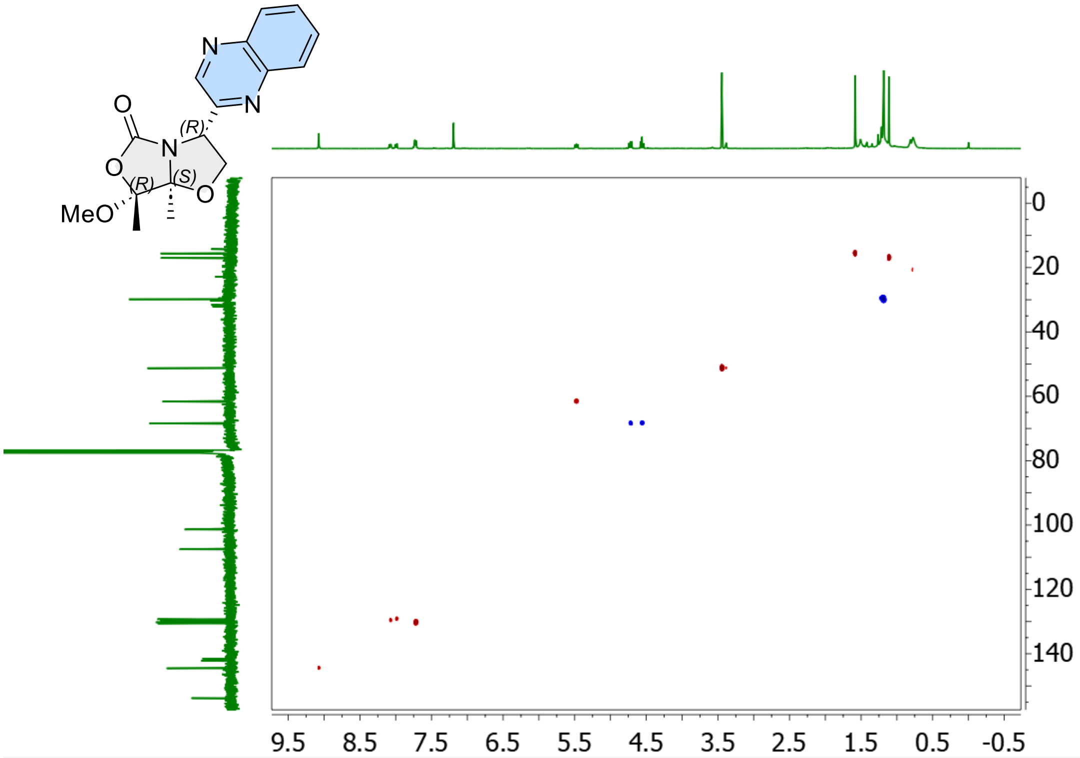

NOESY in $CDCl_3$ (400 MHz) compound 21

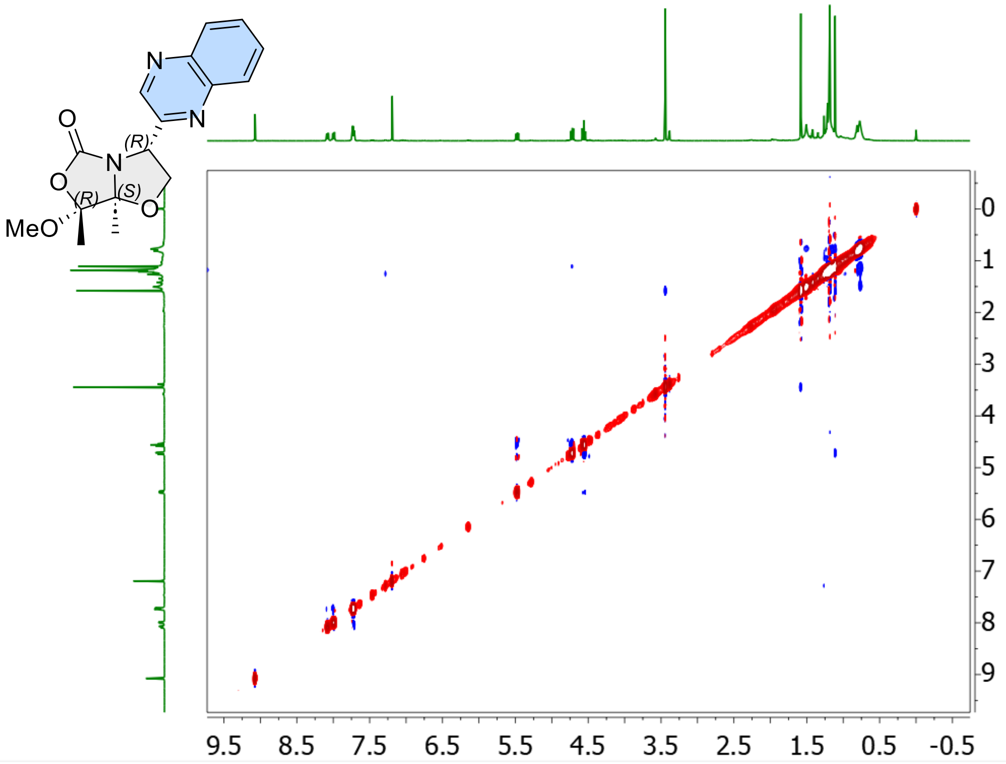

^1H NMR in $CDCl_3$ (400 MHz) compound 22

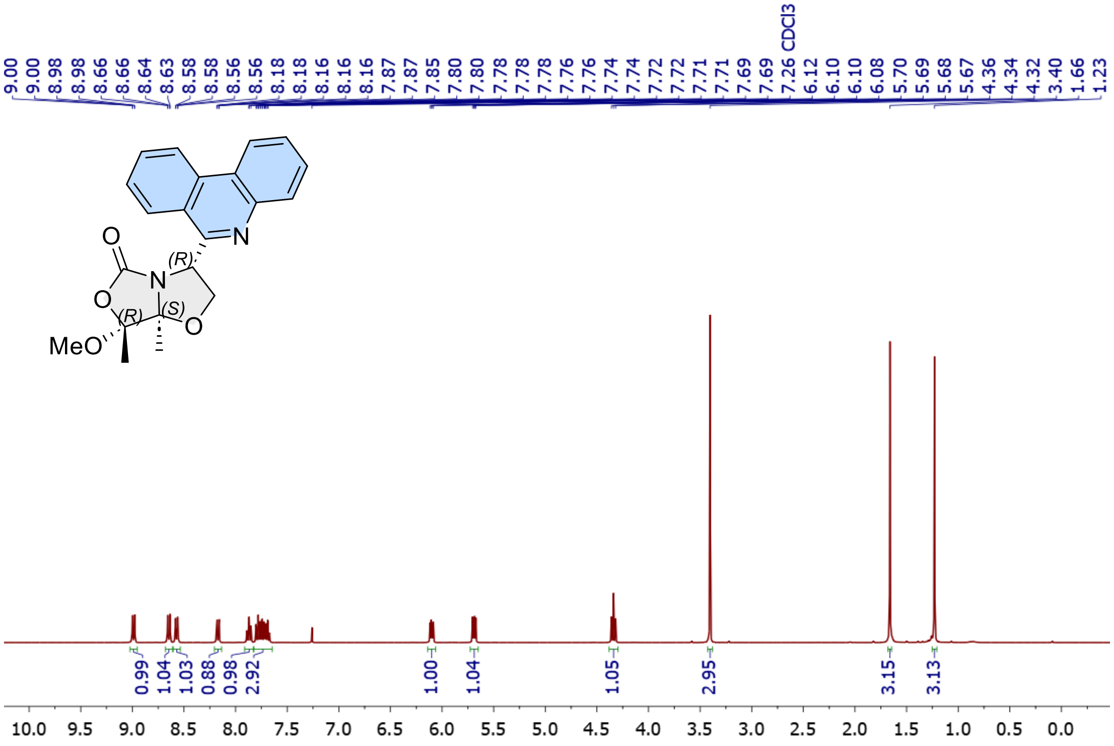

^{13}C {^1H} NMR in $CDCl_3$ (100 MHz) compound 22

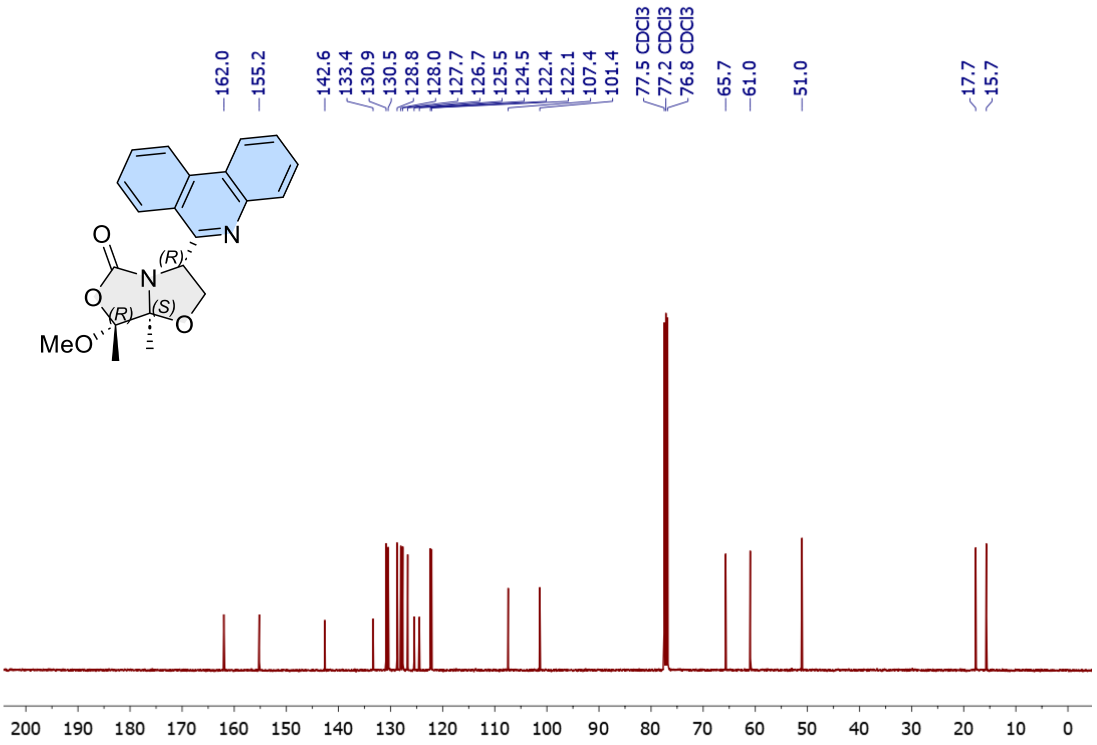

COSY in $CDCl_3$ (400 MHz) compound 22

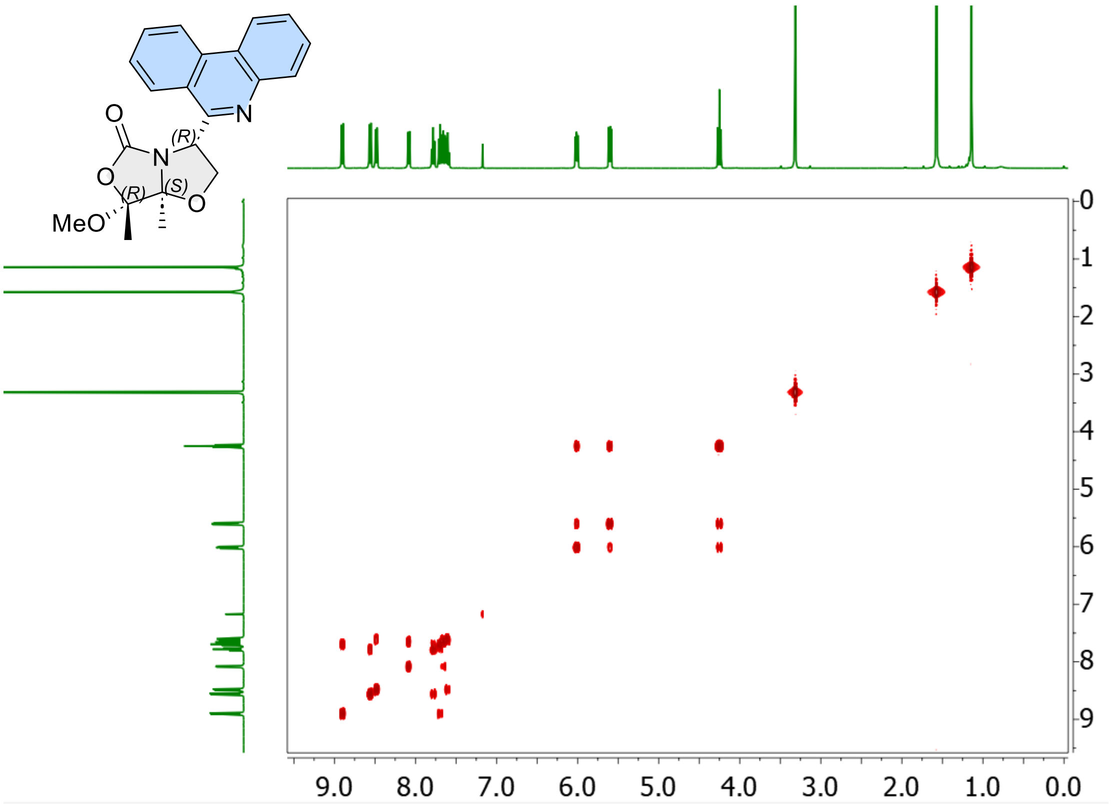

HSQC in $CDCl_3$ (400 MHz) compound 22

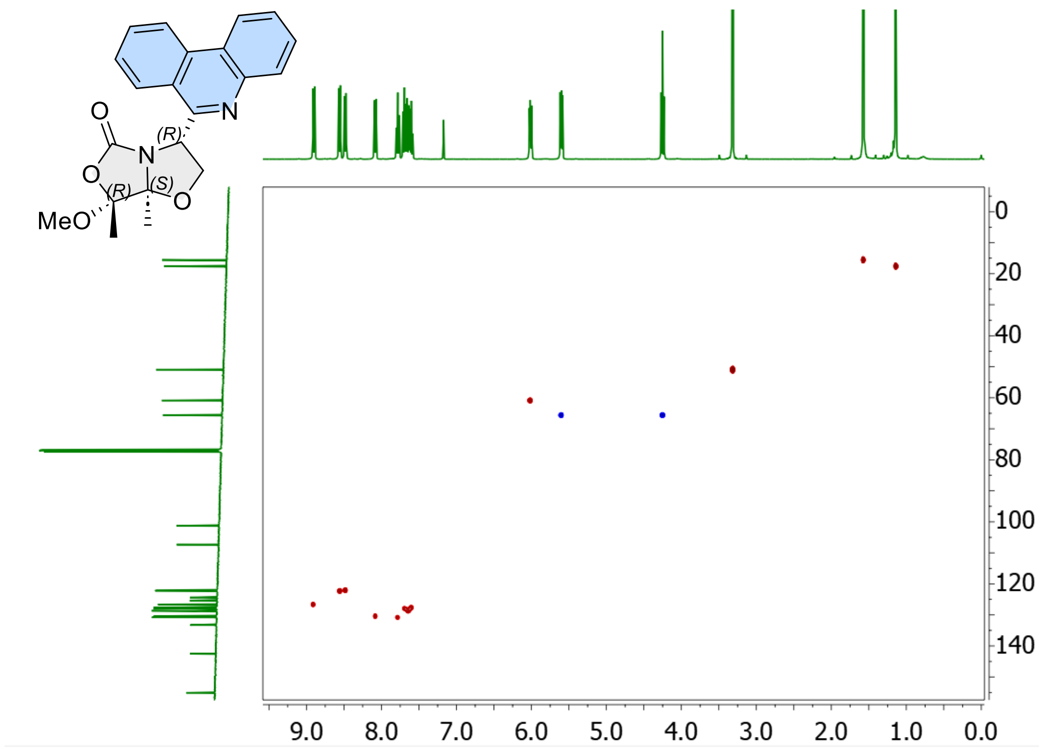

NOESY in $CDCl_3$ (400 MHz) compound 22

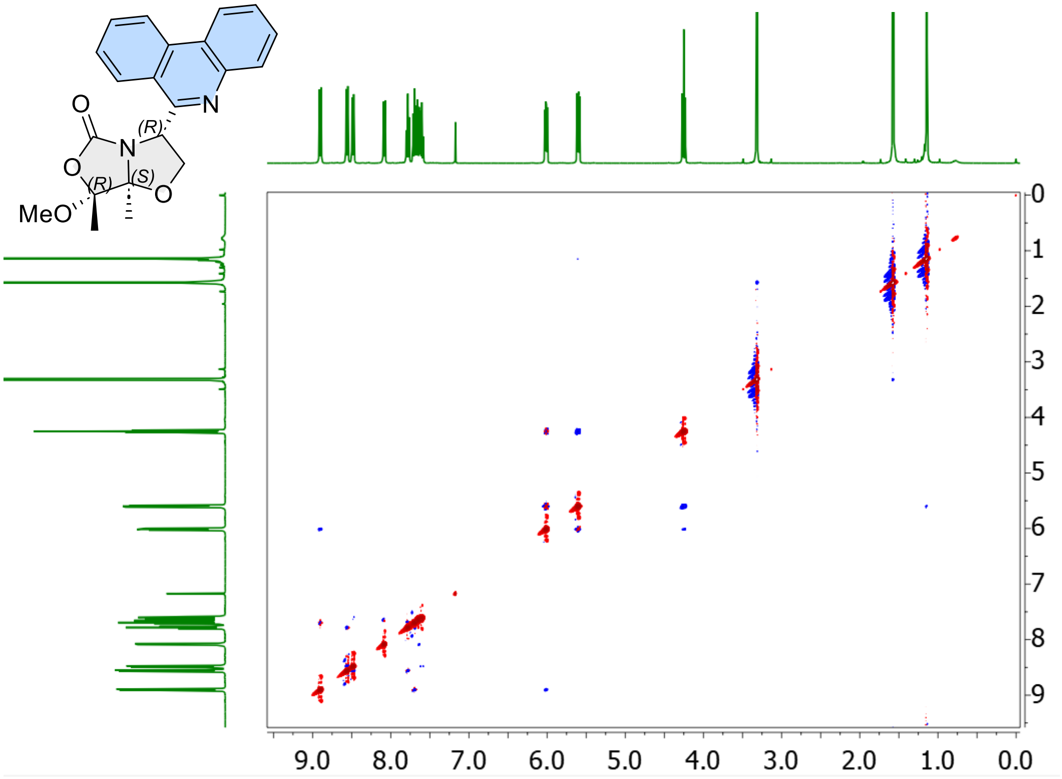

^1H NMR in D_2O (400 MHz) compounds 23

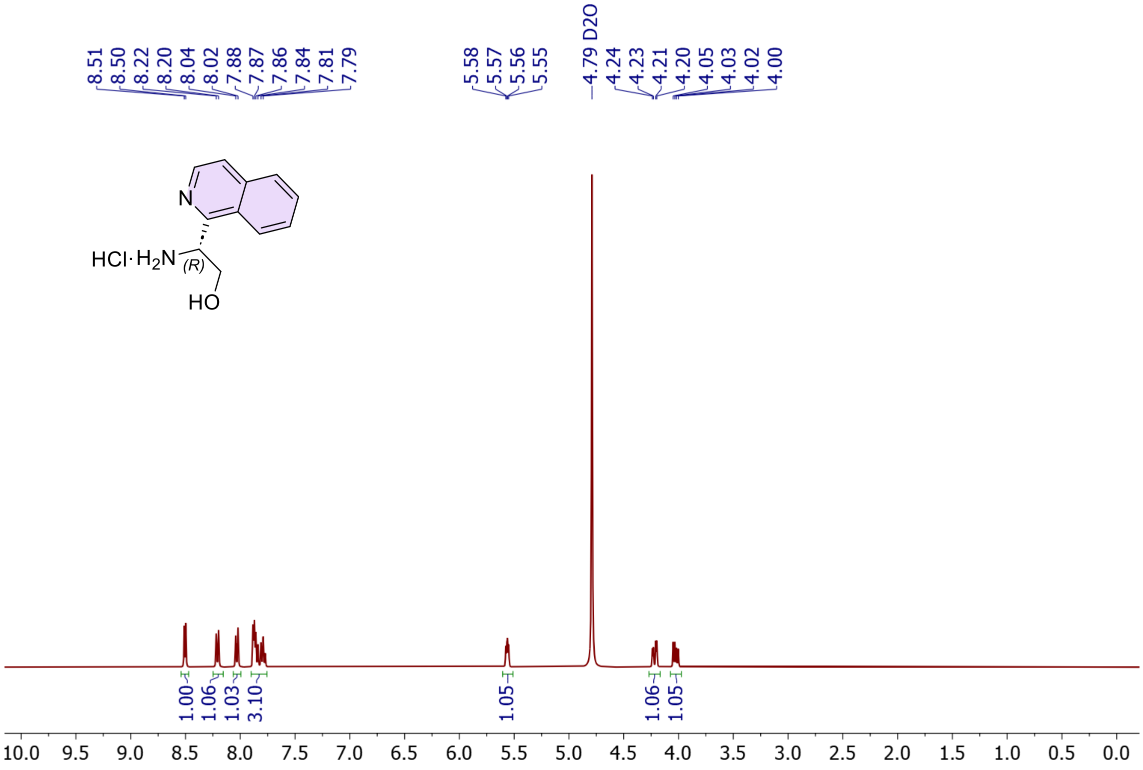

^{13}C {^1H} NMR in D_2O (100 MHz) compounds 23

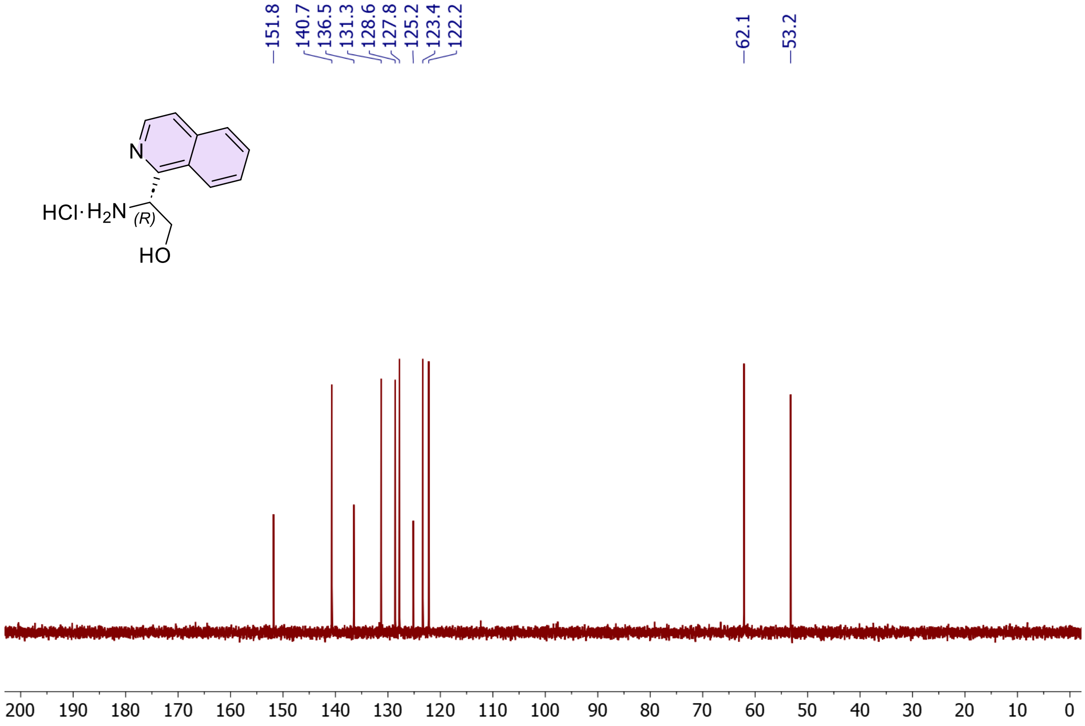

COSY in D_2O (400 MHz) compounds 23

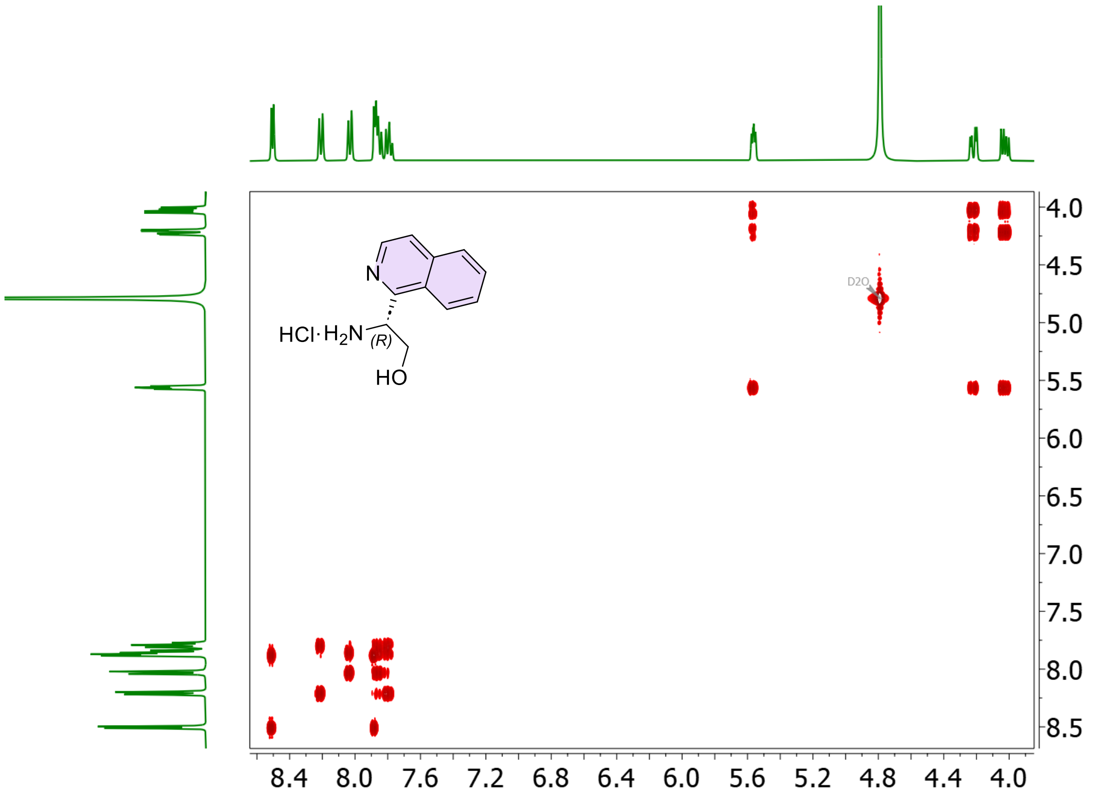

HSQC in D_2O (400 MHz) compounds 23

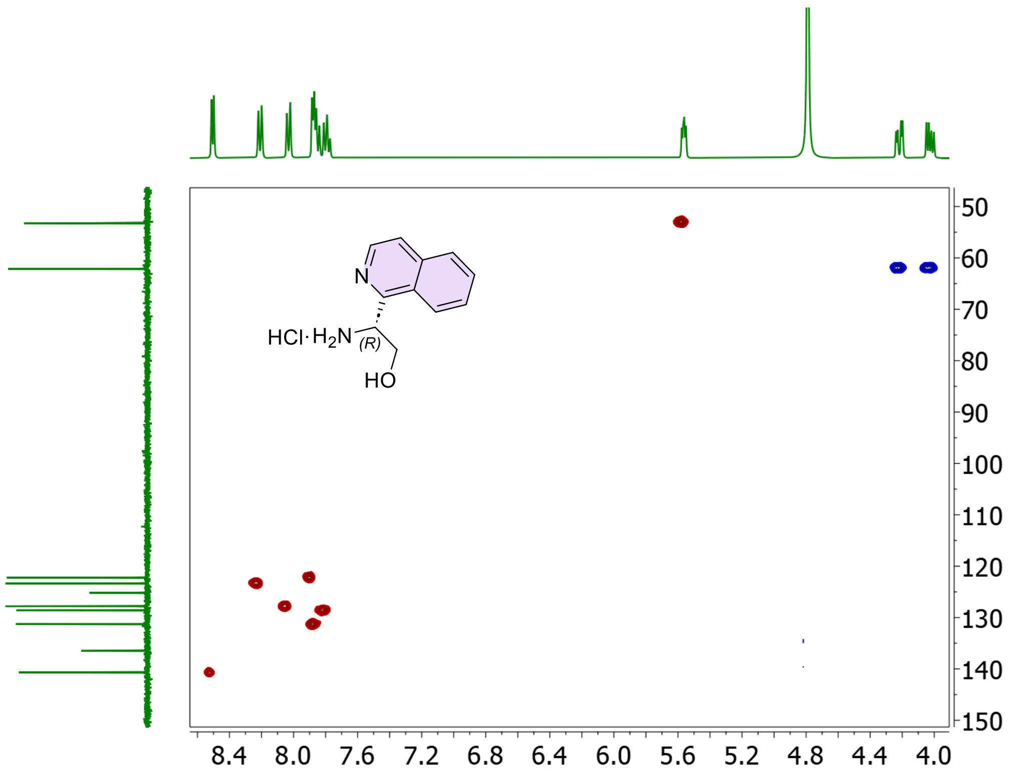

^{1}H NMR in D_2O (400 MHz) compound 24

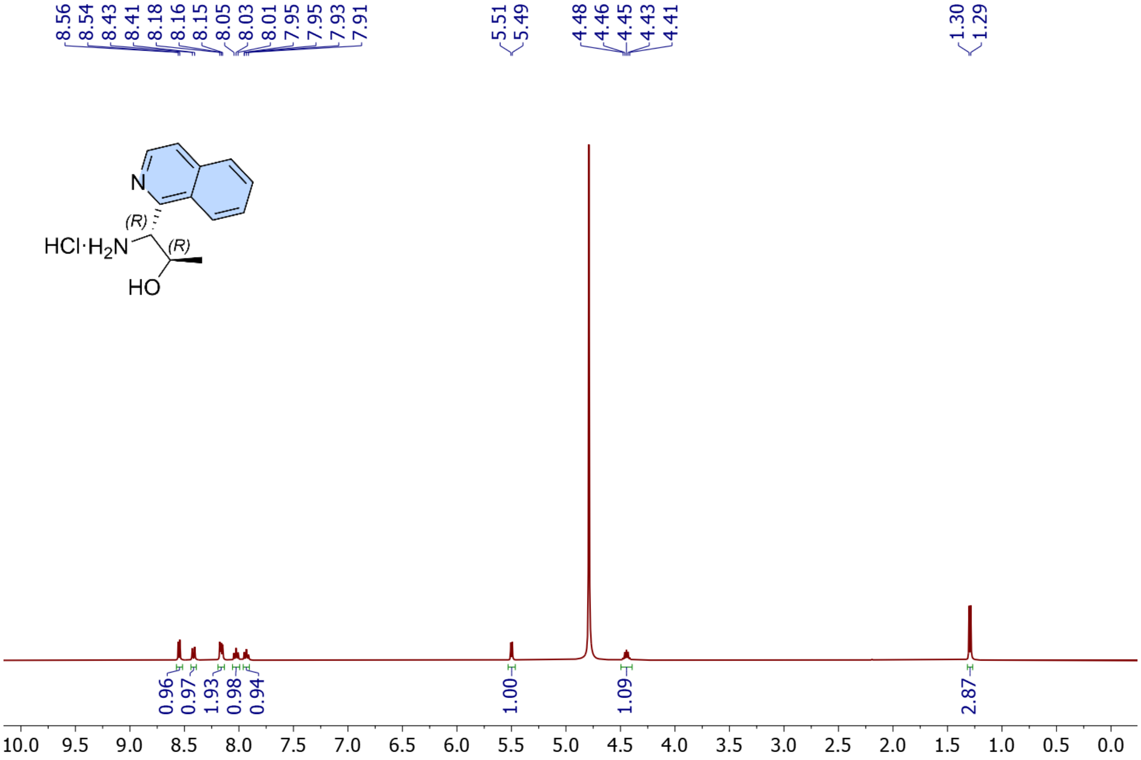

$^{13}C\{^{1}H\}$ NMR in D_2O (100 MHz) compound 24

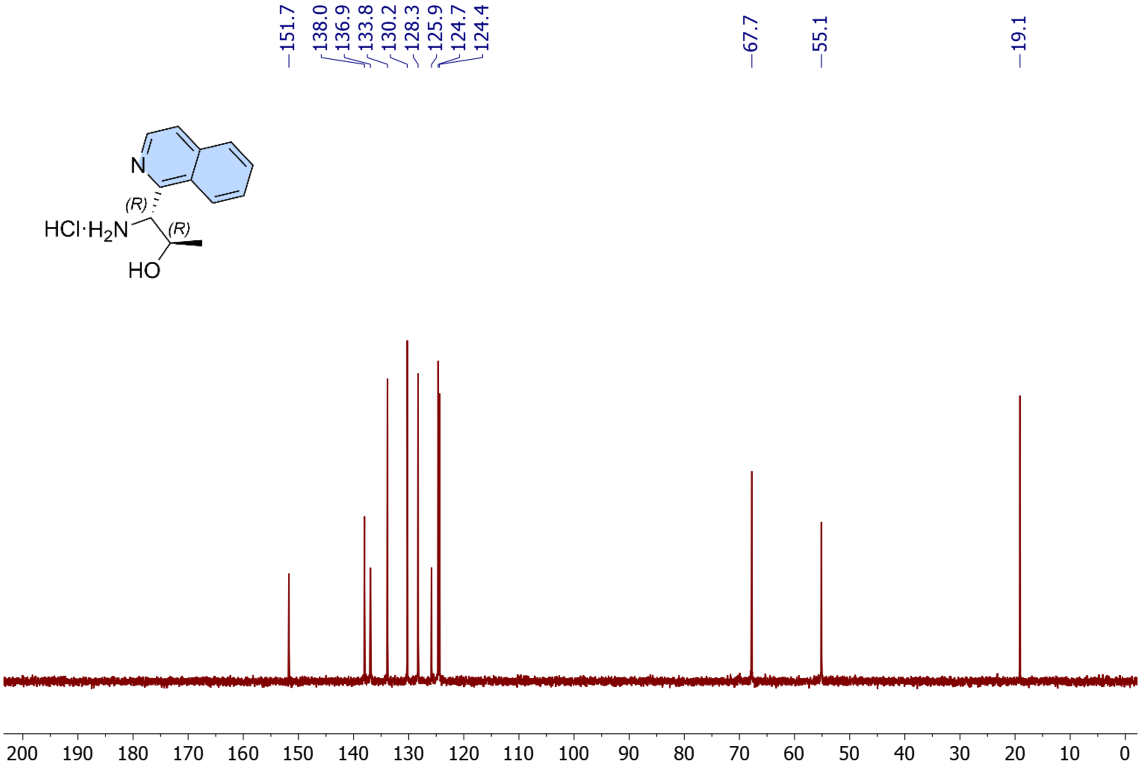

COSY in D_2O (400 MHz) compound 24

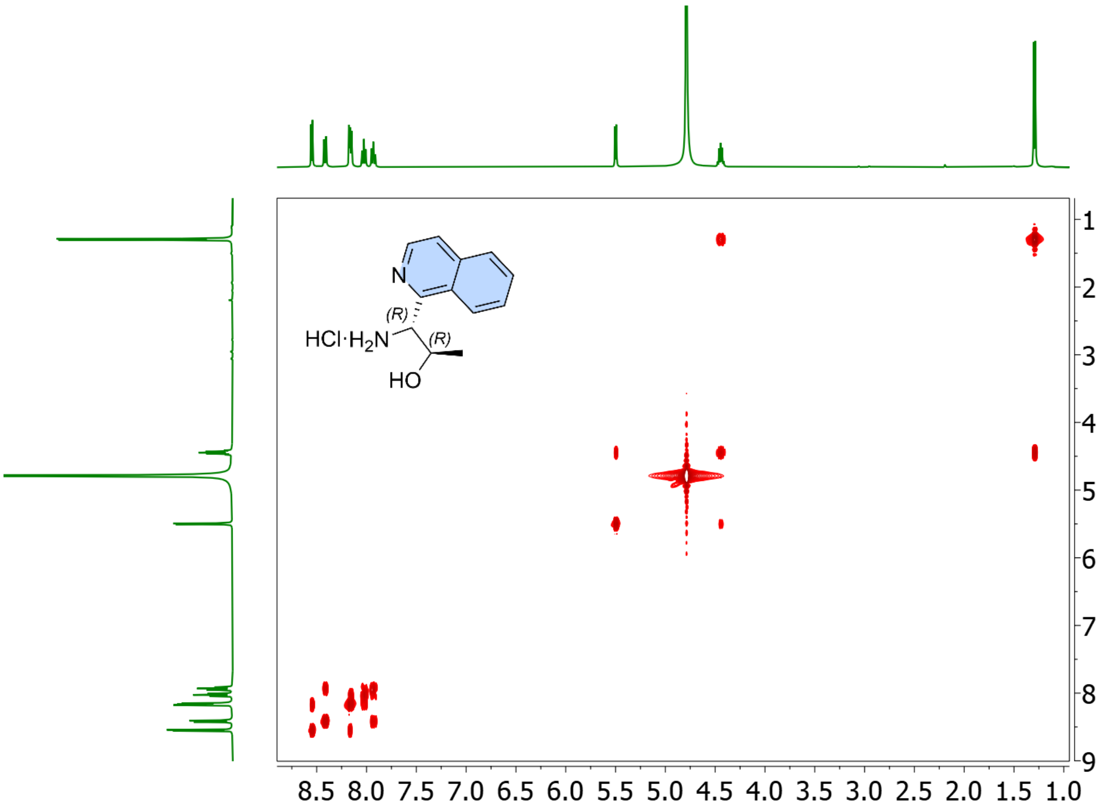

HSQC in D_2O (400 MHz) compound 24

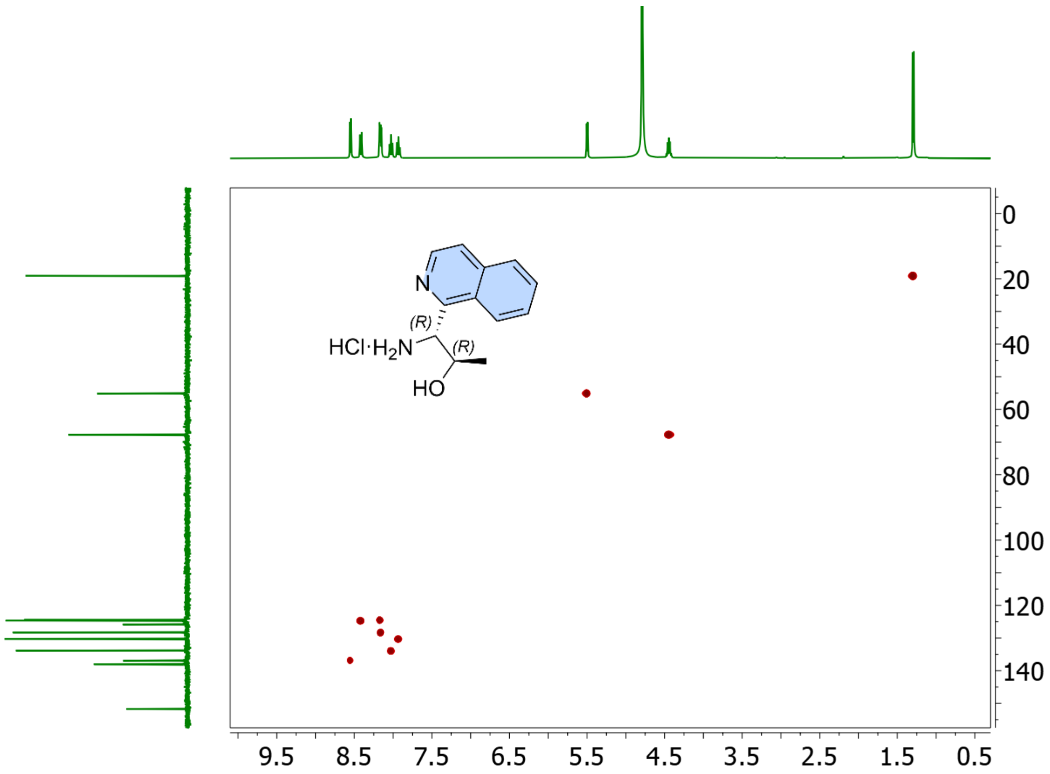

^{1}H NMR in D_2O (400 MHz) compound 25

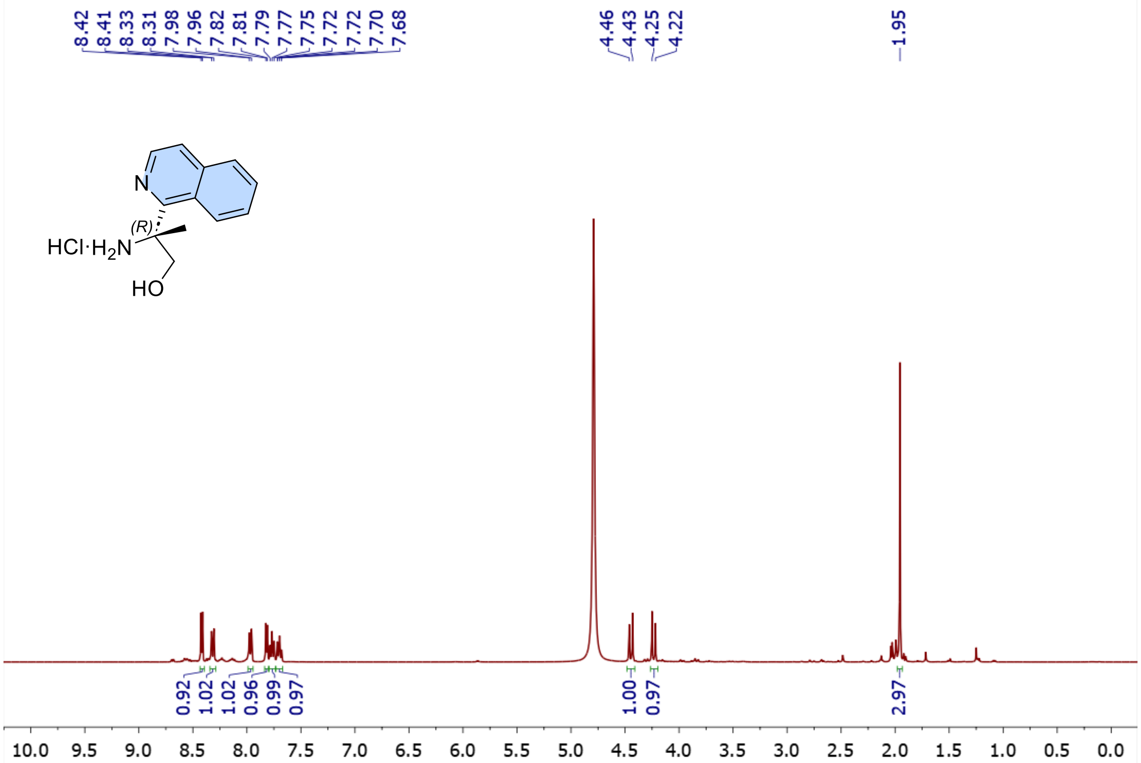

^{13}C {^{1}H} NMR in D_2O (100 MHz) compound 25

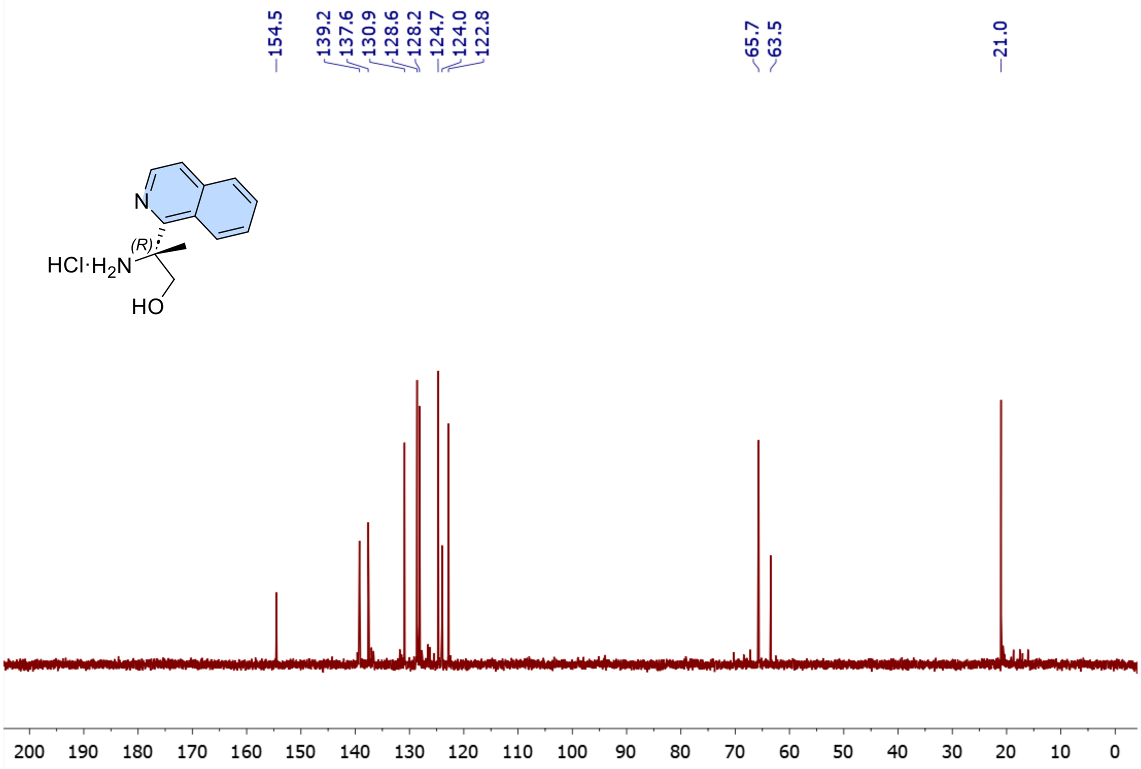

COSY in D_2O (400 MHz) compound 25

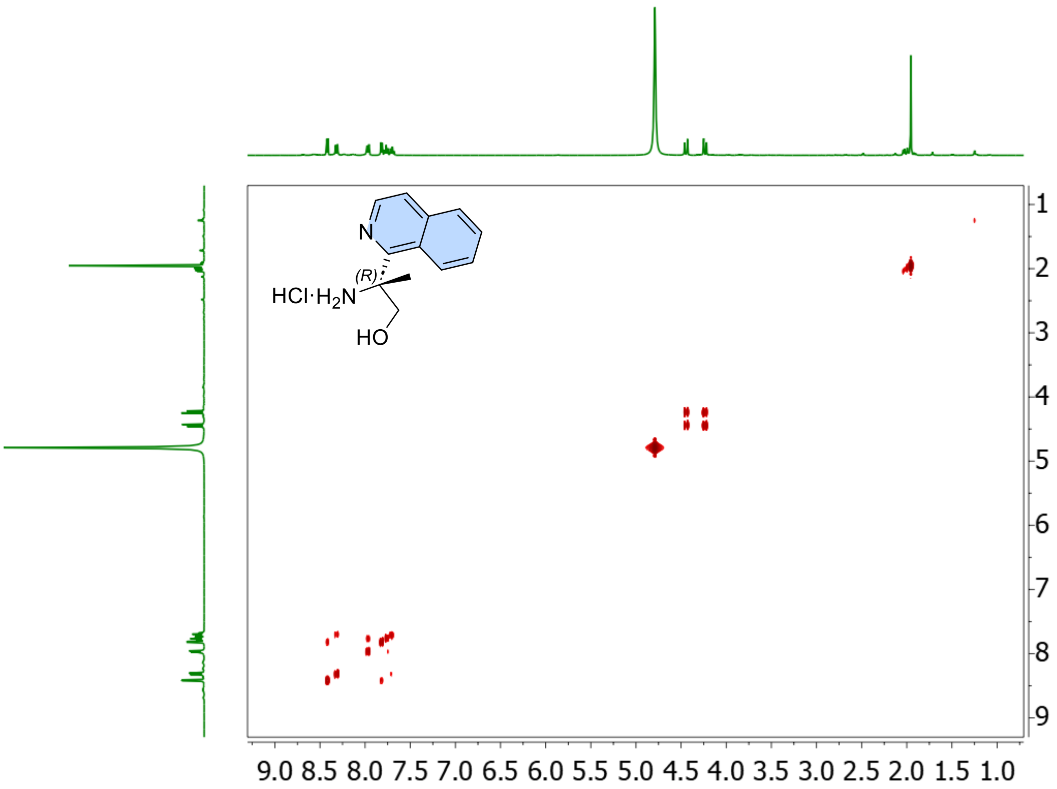

HSQC in D_2O (400 MHz) compound 25

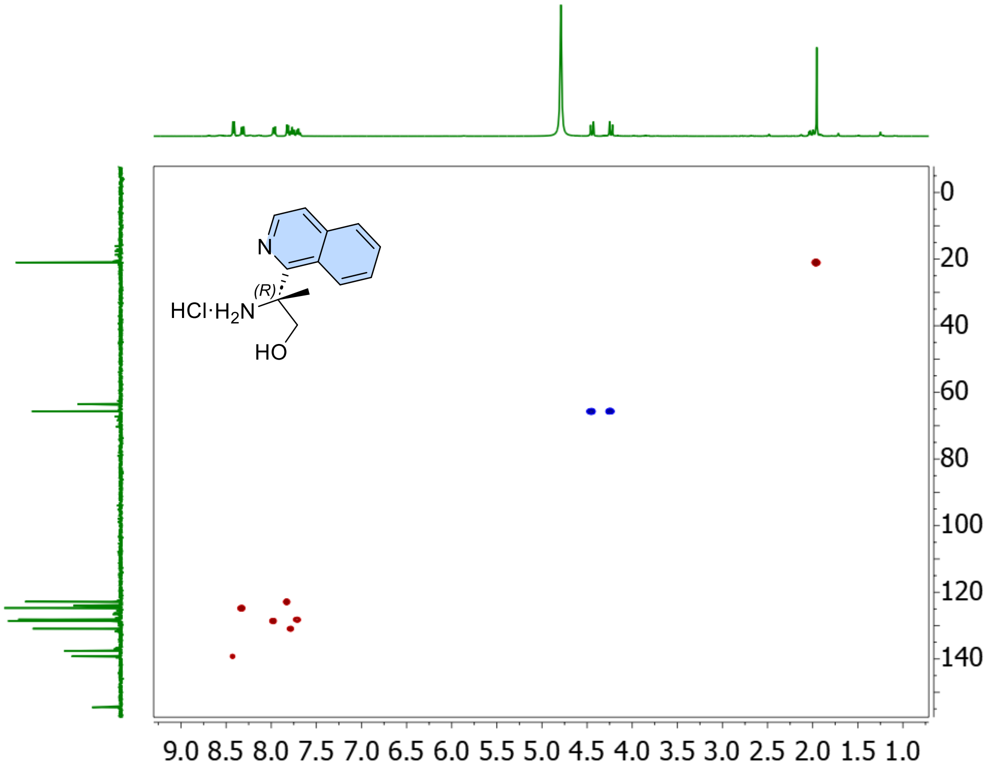

^1H NMR in D_2O (400 MHz) compounds 26

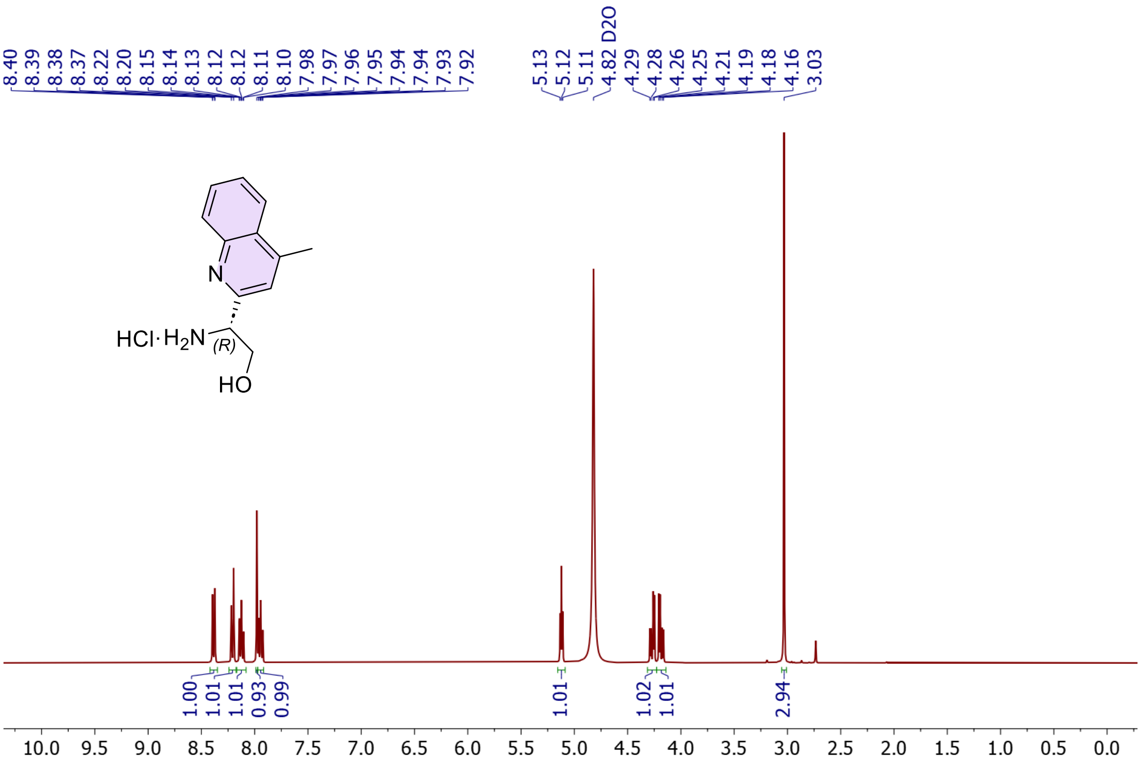

^{13}C {^1H} NMR in D_2O (100 MHz) compounds 26

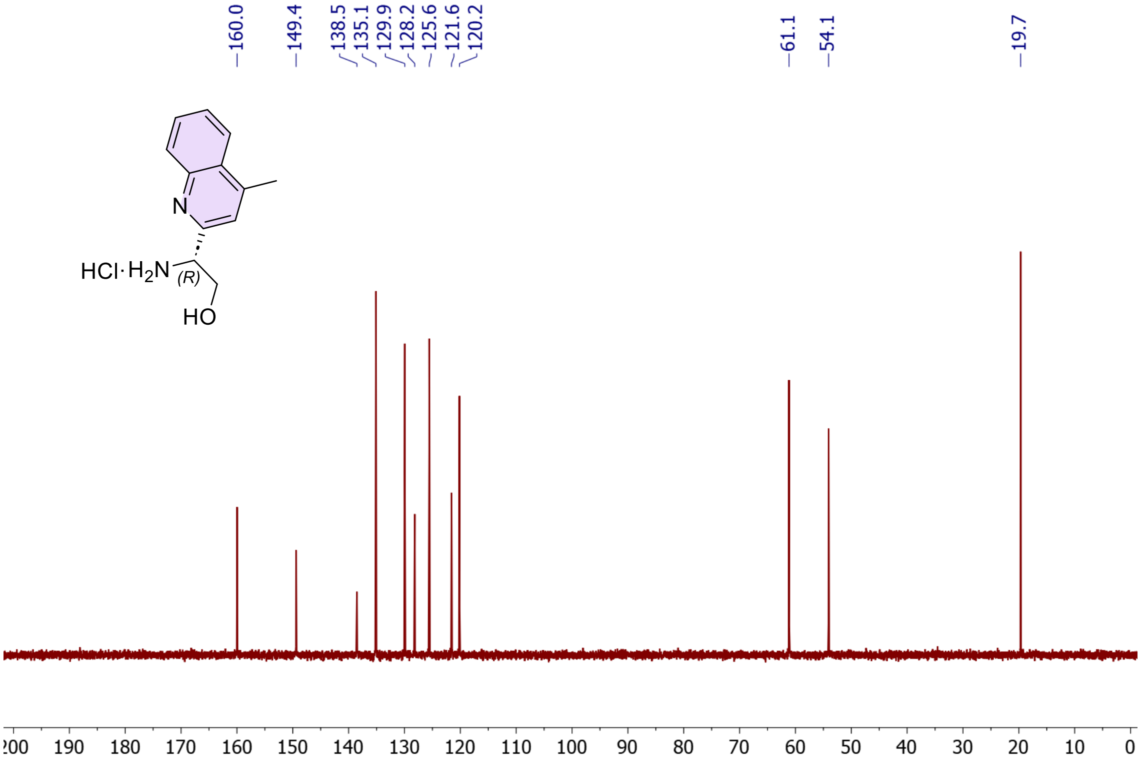

COSY in D_2O (400 MHz) compounds 26

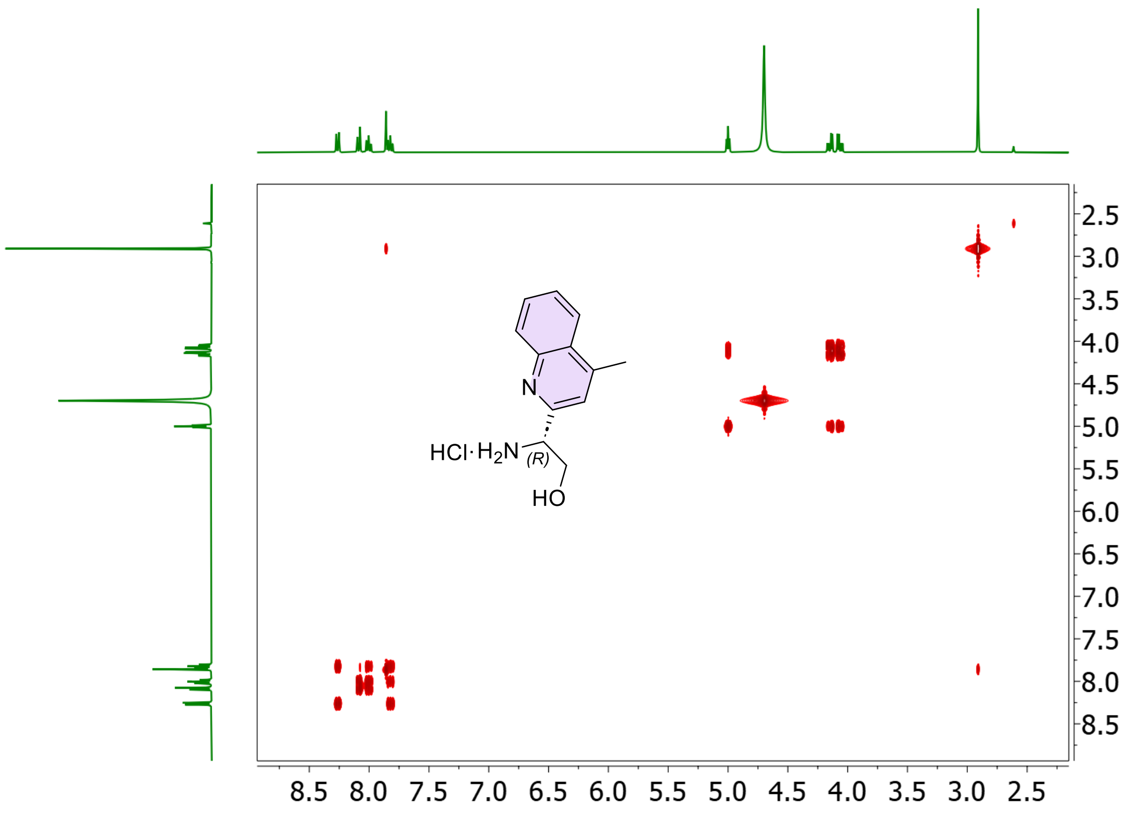

HSQC in D_2O (400 MHz) compounds 26

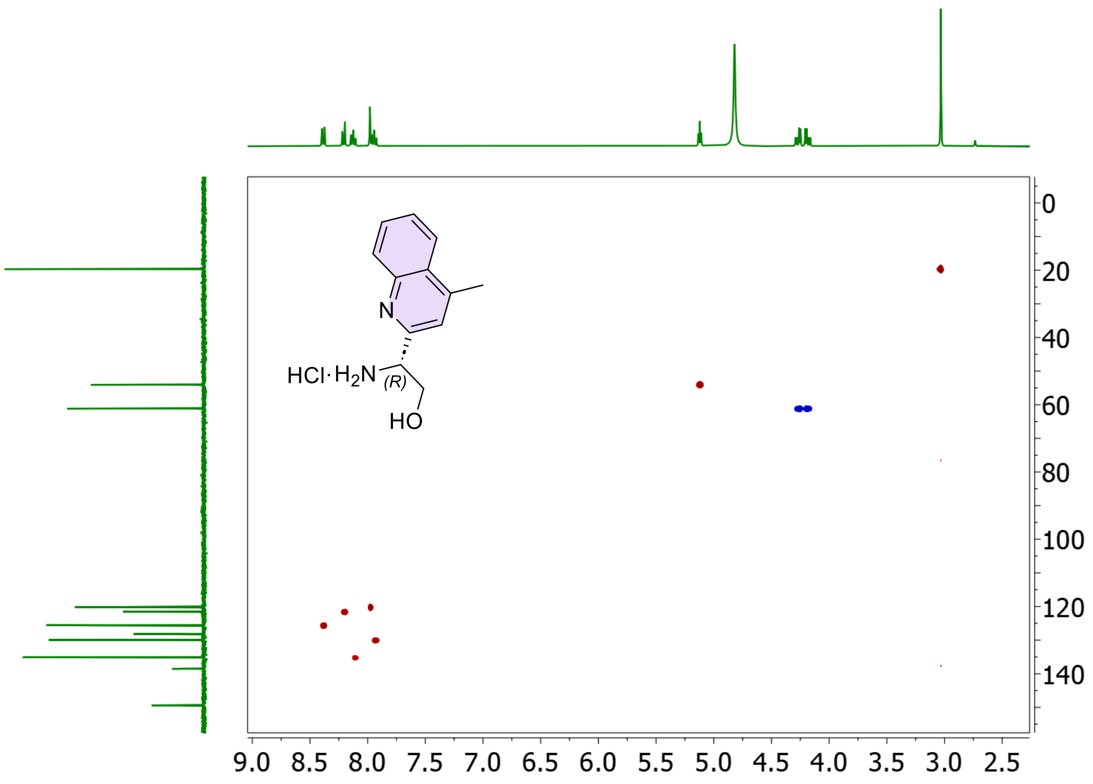

^{1}H NMR in $CDCl_3$ (400 MHz) of the crude mixture of compound 27

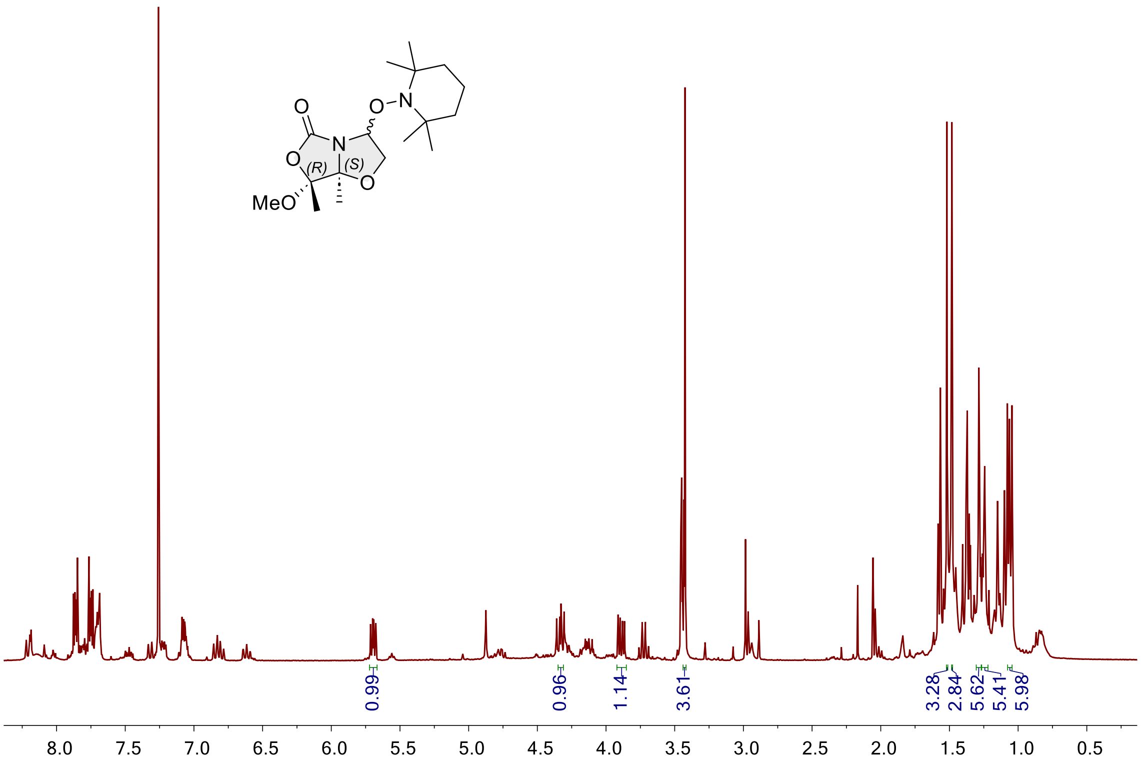

^{1}H NMR in $CDCl_3$ (400 MHz) compound 27 (after purification by column chromatography)

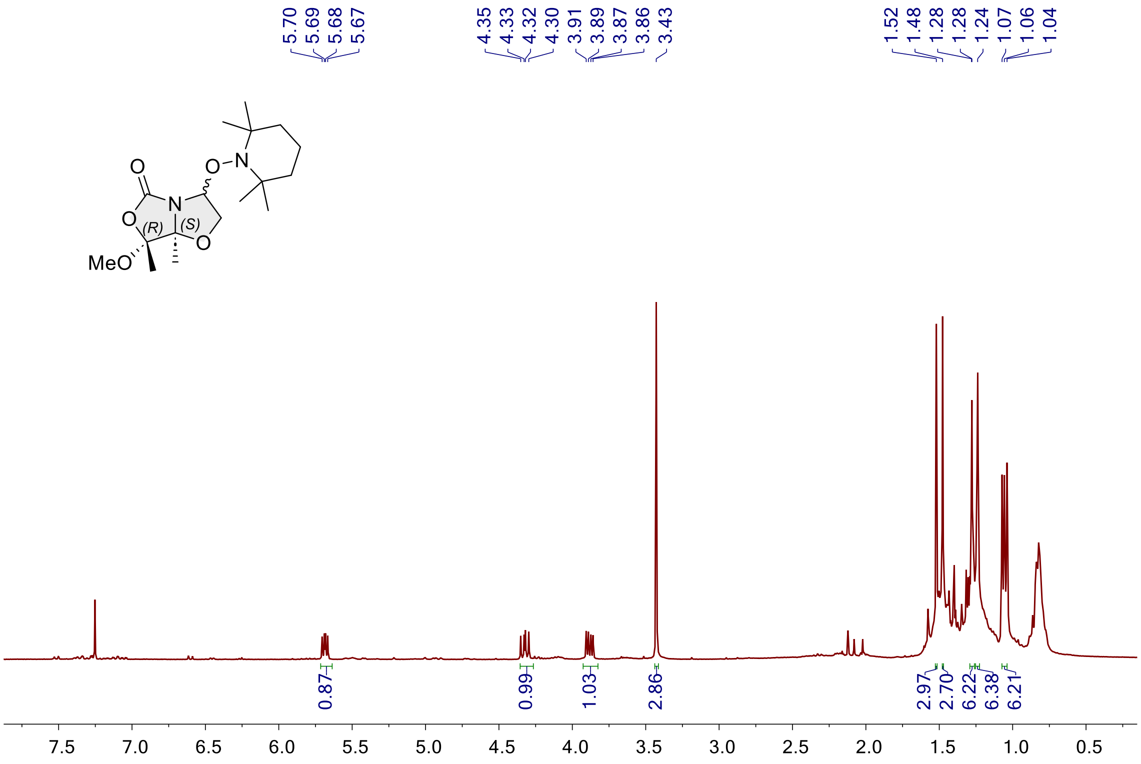

^{1}H NMR in $CDCl_3$ (400 MHz) compound 27 after 16 h

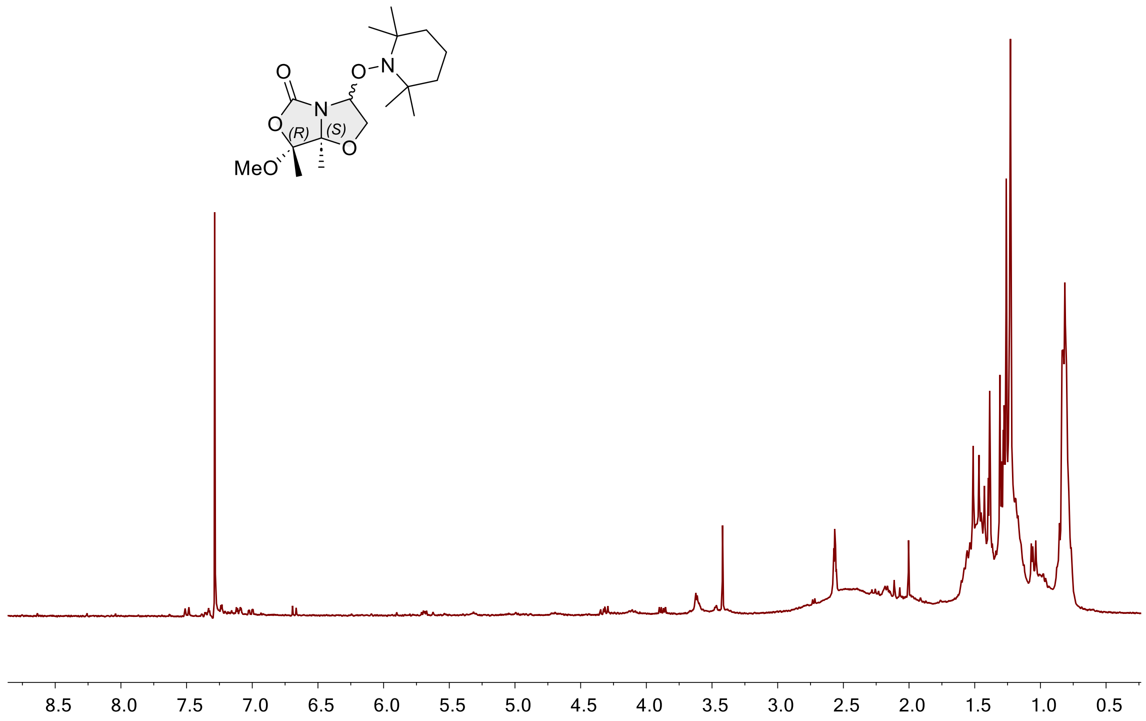

12. NOESY experiments

The absolute configurations of the new stereocenters created in the photoredox Minisci reaction of active esters **4**, **6** and **8** were assessed by 2D-NOESY experiments. Initially, we could distinguish both hydrogens of the methylene group –C3-**CH₂**-CH– (marked as Hβ1 and Hβ2 in the picture) of the rigid bicyclic system attending to the NOE contact between the hydrogens of methyl group –**CH₃**-C7a– (Me7a) and Hβ2 of the rigid bicyclic system, which indicates that they are on the same face of the molecule. This feature is corroborated by the NOE contact between the hydrogens of methyl group –**CH₃**-C7– (Me7) and Hβ1. For the case of the stereocenter (C3) corresponding, for instance, to Cα of adduct **5** formed in the Minisci reaction of **4** with isoquinoine, the NOE contacts of Hα with both Hβ1 and Hβ2 make difficult the analysis. However, the NOE contacts between H3' of isoquinoline ring and Hβ2 along with the NOE contacts of H8' of isoquinoline with Hα and Hβ1 allow to conclude that C3 displays an (*R*)-configuration in compound **5**. Similar structural characteristics were found in adducts **7** and **9**. Alternatively, these structural features were also determined by X-ray analysis of a single crystal of compound **5** (see below). NOTE: H^1 and H^2 stand $H^{\beta1}$ and $H^{\beta2}$ or $H^{\beta'}$and H^{β}, respectively, in all figures.

Compound 5

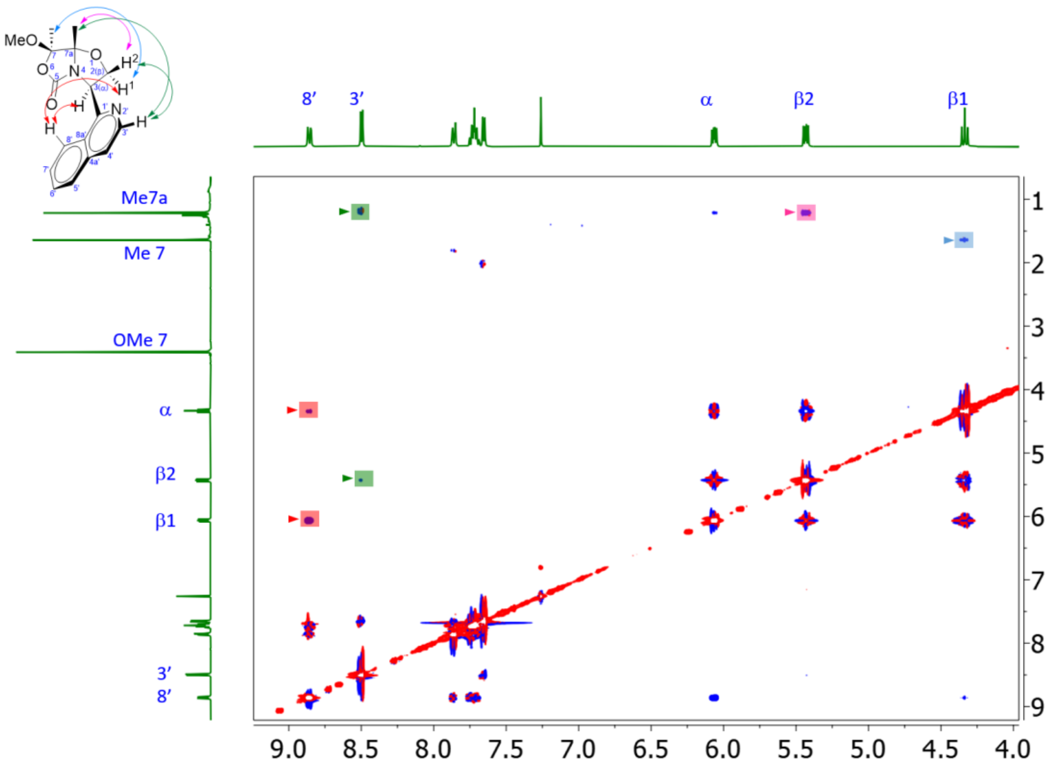

Compound 7

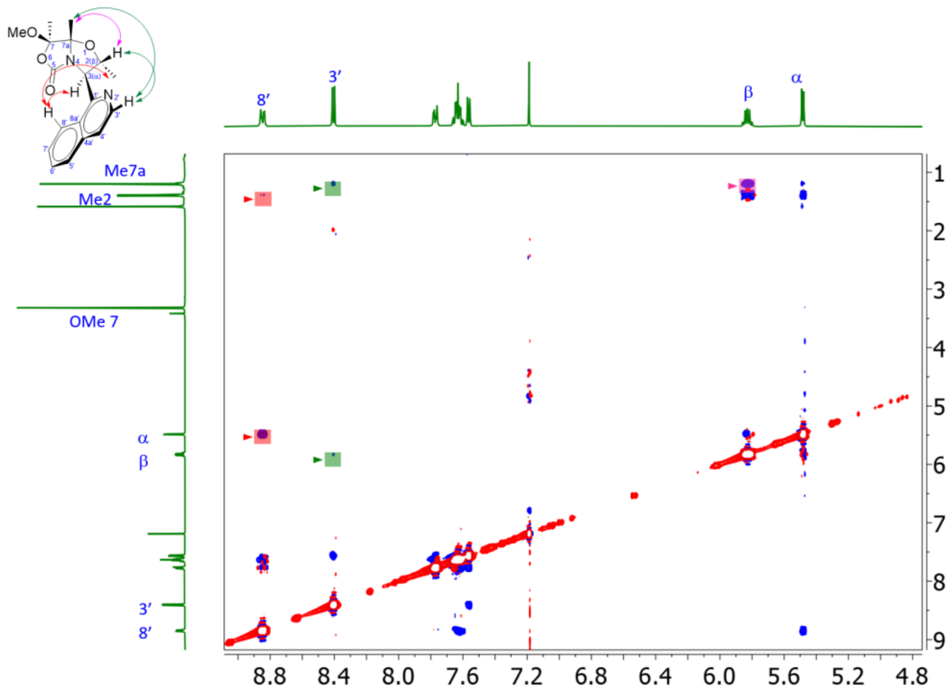

Compound 9

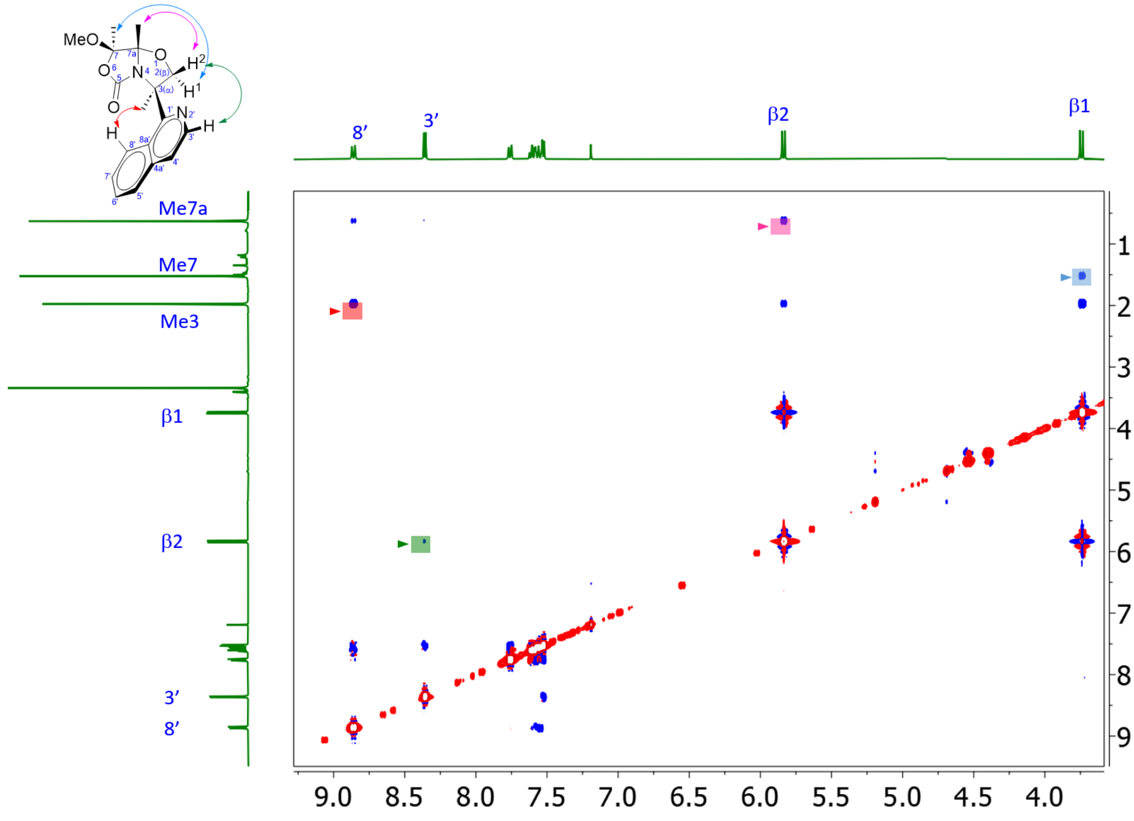

13. Quantum mechanical calculations.

Full geometry optimizations and transition structure (TS) searches were carried out with Gaussian 16[S9] using the M06-2X hybrid functional[S10] and 6-311G(d,p) basis set with ultrafine integration grids. Bulk solvent effects in *N,N*-dimethylformamide (DMF) were considered implicitly through the IEF-PCM polarizable continuum model.[S11] The possibility of different conformations was taken into account for all structures. All stationary points were characterized by a frequency analysis performed at the same level used in the geometry optimizations from which thermal corrections were obtained at 298.15 K. The quasiharmonic approximation reported by Truhlar *et al.* was used to replace the harmonic oscillator approximation for the calculation of the vibrational contribution to enthalpy and entropy.[S12] Scaled frequencies were not considered. Mass-weighted intrinsic reaction coordinate (IRC) calculations were carried out using the Hessian-based predictor-corrector integrator scheme by Hratchian and Schlegel[S13] in order to ensure that the TSs indeed connected the appropriate reactants and products. Free energies calculated using the gas phase standard state concentration (1 atm = 1/24.5 M) were converted to reproduce the standard state concentration in solution (1 M) by subtracting or adding 1.89 kcal mol^{-1} for bimolecular additions and decompositions, respectively. Gibbs free energies (ΔG) were used for the discussion on the relative stabilities of the considered structures. The lowest energy conformer for each calculated stationary point (Supplementary Figure S3) was considered in the discussion; all the computed structures can be obtained from authors upon request. Non-covalent-interactions (NCI) were calculated with NCIPLOT 4.[S14] Cartesian coordinates, electronic energies, entropies, enthalpies, Gibbs free energies, and lowest frequencies of the calculated structures are summarized in Supplementary Table S5.

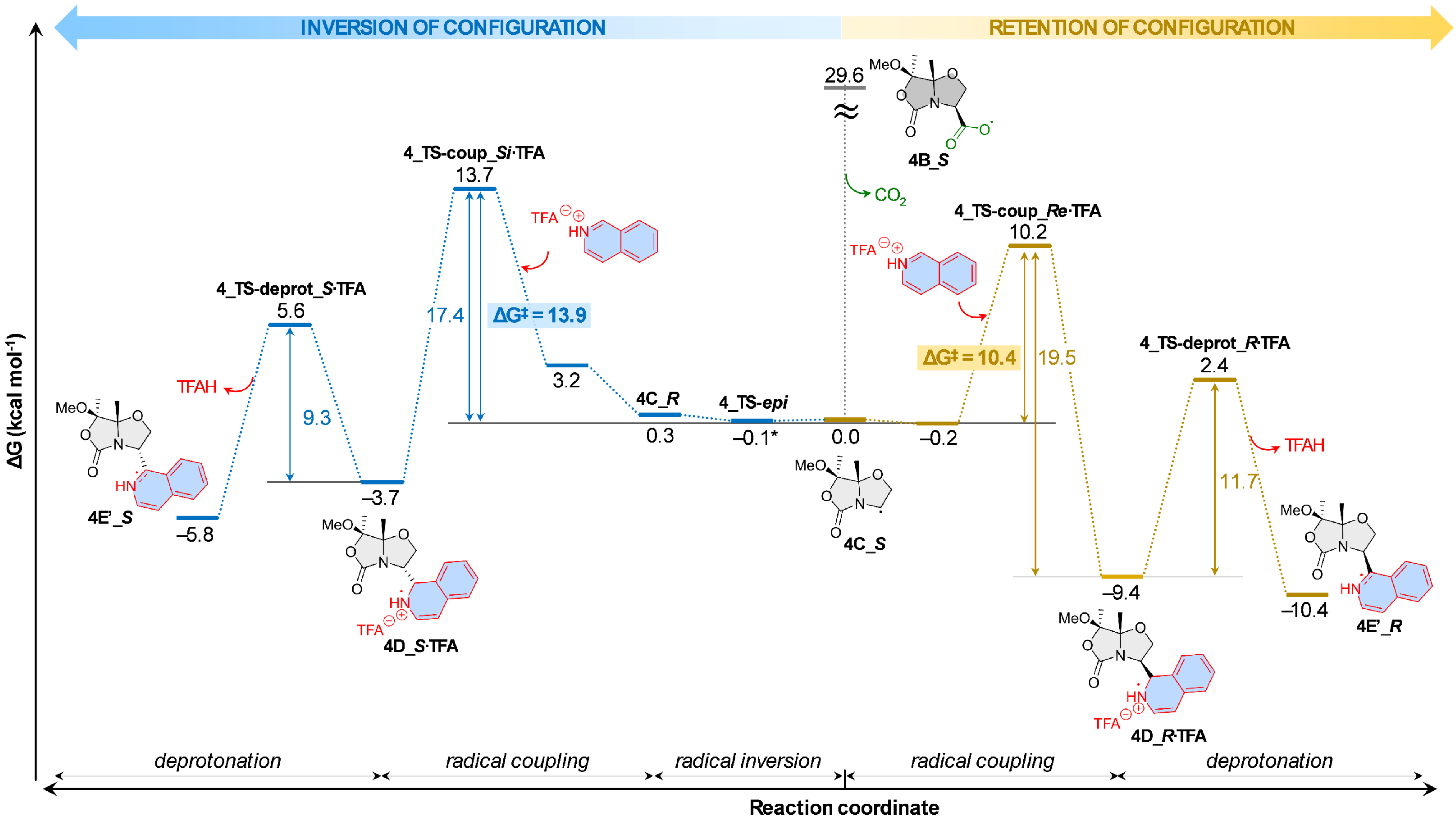

Figure S3. Minimum energy pathways (MEP) calculated with PCM(DMF)/M06-2X/6-311G(d,p) for reactions originating from Ser-derived bicyclic carboxyl radical **4B_*S***. This species decarboxylates to form pyramidalized C-centered radicals in (*S*) and (*R*) configurations in a rapid equilibrium; each of those radicals then undergo diastereoselective radical coupling with the isoquinolinium-trifluoroacetate ion pair (**4_TS-coup**). The resulting intermediate adducts, **4D·TFA**, subsequently undergo internal deprotonation by TFA to yield quasi-aromatic radicals **4E'**. The bicyclic C-centered radical pyramidalized in (*S*) configuration (**4C_*S***) has been arbitrarily assigned a free energy value (ΔG) of zero. *The epimerization of bicyclic C-centered radicals is virtually barrierless, which in some cases leads to negative $\Delta G^{\ddagger}$ values due to inaccuracies in the calculation of vibrational entropies.

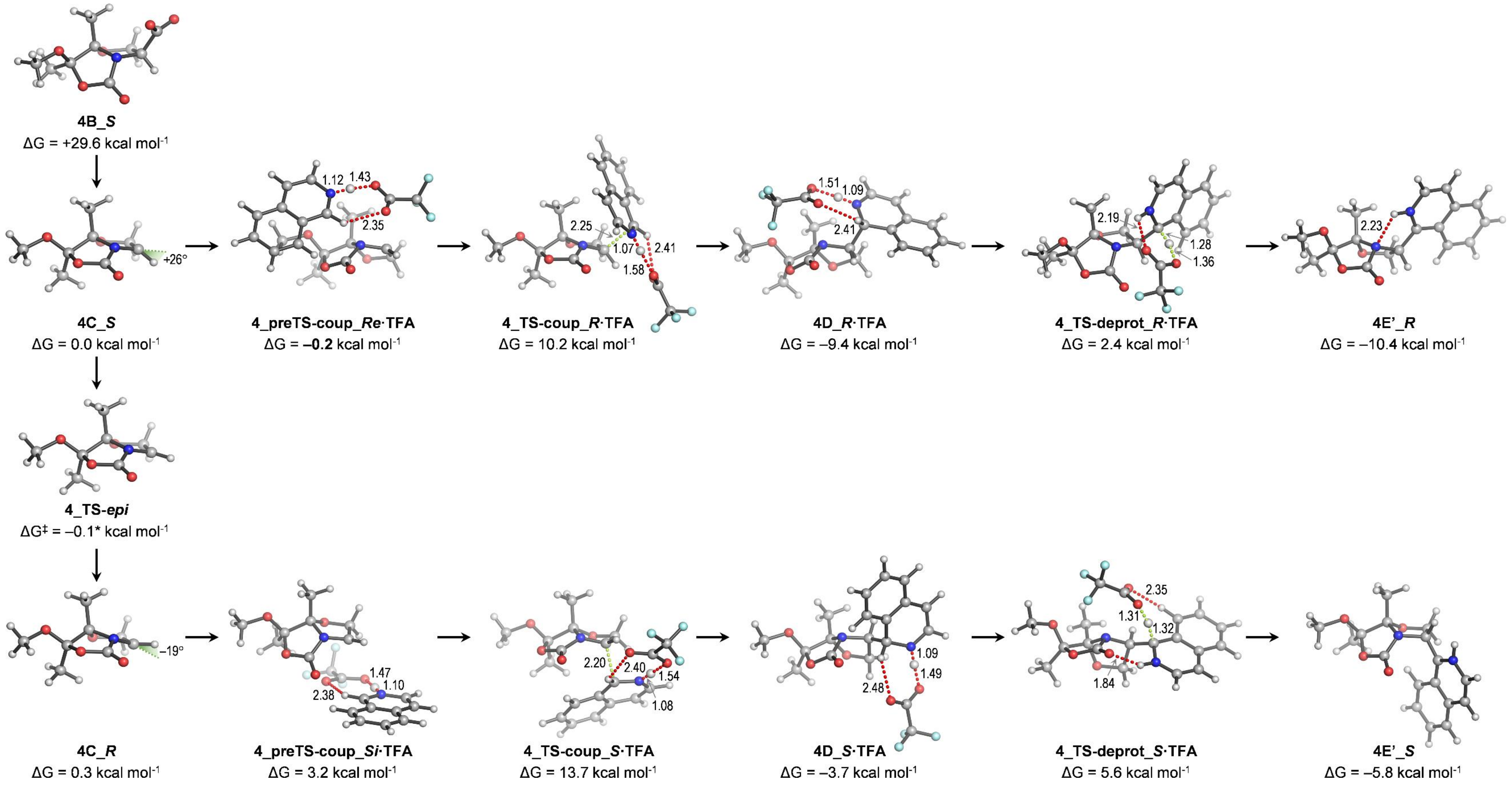

Figure S4. Guide to compound numbering of calculated structures for the minimum energy pathways (MEP; only the lowest energy conformers are shown) calculated for the reactions originating from Ser-derived bicyclic carboxyl radical **4B_*S*** and isoquinolinium-trifluoroacetate ion pair, as depicted in Figure S3.

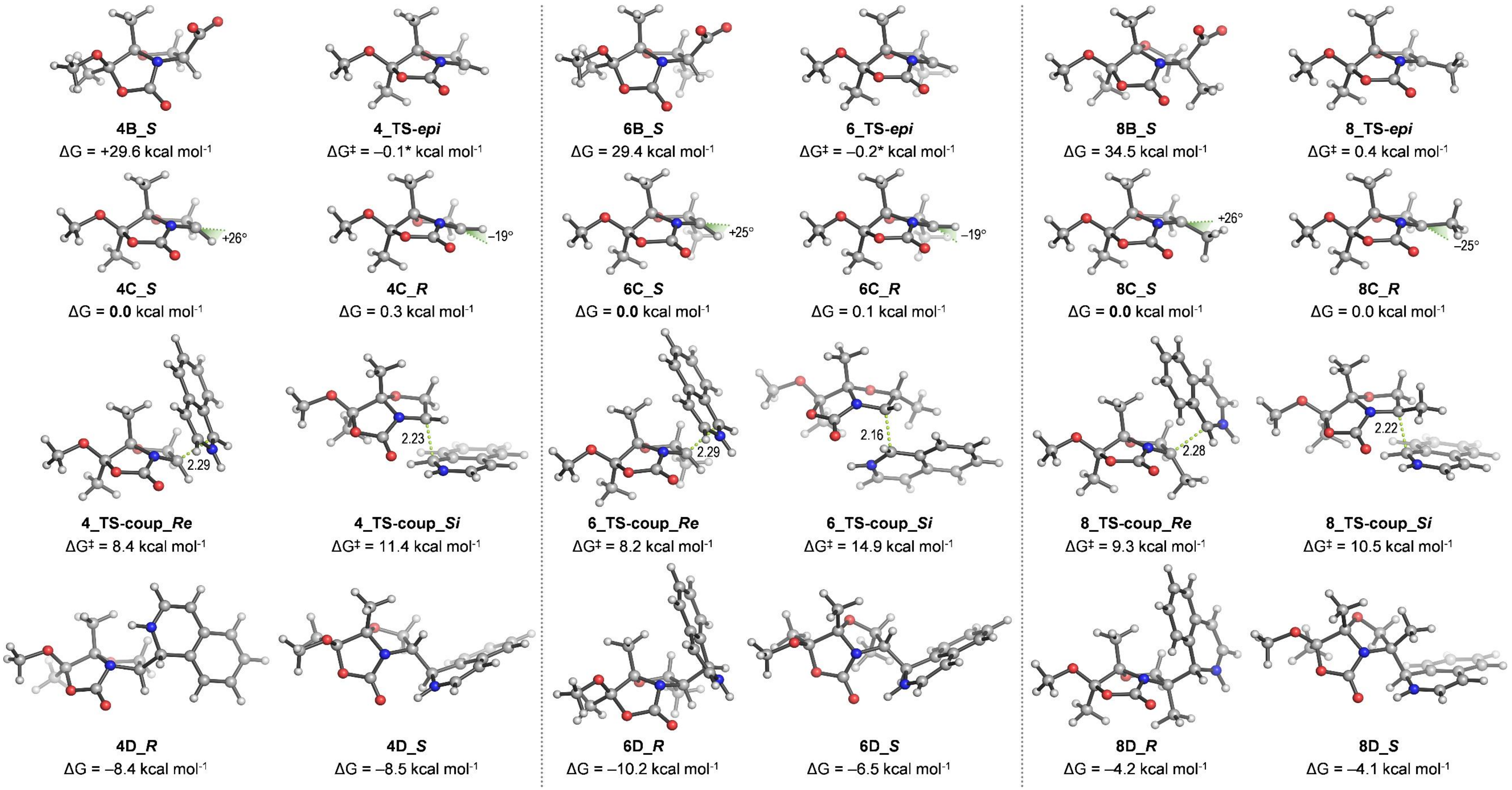

Figure S5. Guide to compound numbering of calculated structures for bicyclic alkyl radical generation and radical C–C coupling with cationic isoquinolinium as an electrophile (only the lowest energy conformers are shown). The bicyclic C-centered radicals pyramidalized in (*S*) configuration (**4C_*S***, **6C_*S*** and **8C_*S***) have been arbitrarily assigned a free energy value (ΔG) of zero. *The epimerization of bicyclic C-centered radicals is virtually barrierless, which in some cases leads to negative $\Delta G^{\ddagger}$ values due to inaccuracies in the calculation of vibrational entropies. Distances are given in angstrom and pyramidalization angles in degrees.

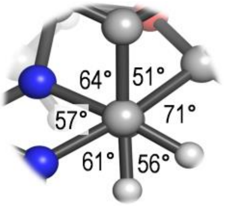

4_TS-coup_*Re*
ΔG‡ = 8.4 kcal mol-1

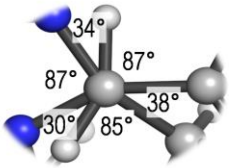

4_TS-coup_*Si*
ΔG‡ = 11.4 kcal mol-1

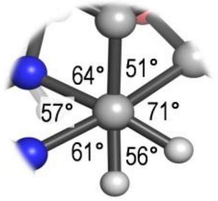

6_TS-coup_*Re*
ΔG‡ = 8.2 kcal mol-1

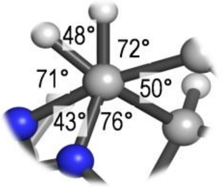

6_TS-coup_*Si*
ΔG‡ = 14.9 kcal mol-1

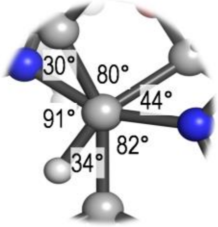

8_TS-coup_*Re*
ΔG‡ = 9.3 kcal mol-1

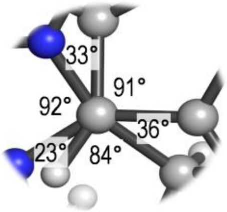

8_TS-coup_*Si*
ΔG‡ = 10.5 kcal mol-1

Figure S6. Newman projections along the forming C–C bond in low energy transition states (TS) calculated for the nucleophilic attack of bicyclic C-centered radicals to cationic isoquinolinium. Dihedral angles are given in degrees. Note the greater staggering in the MeSer-derived transition structures due to the presence of the α-methyl group at position C3 of the bicycle.

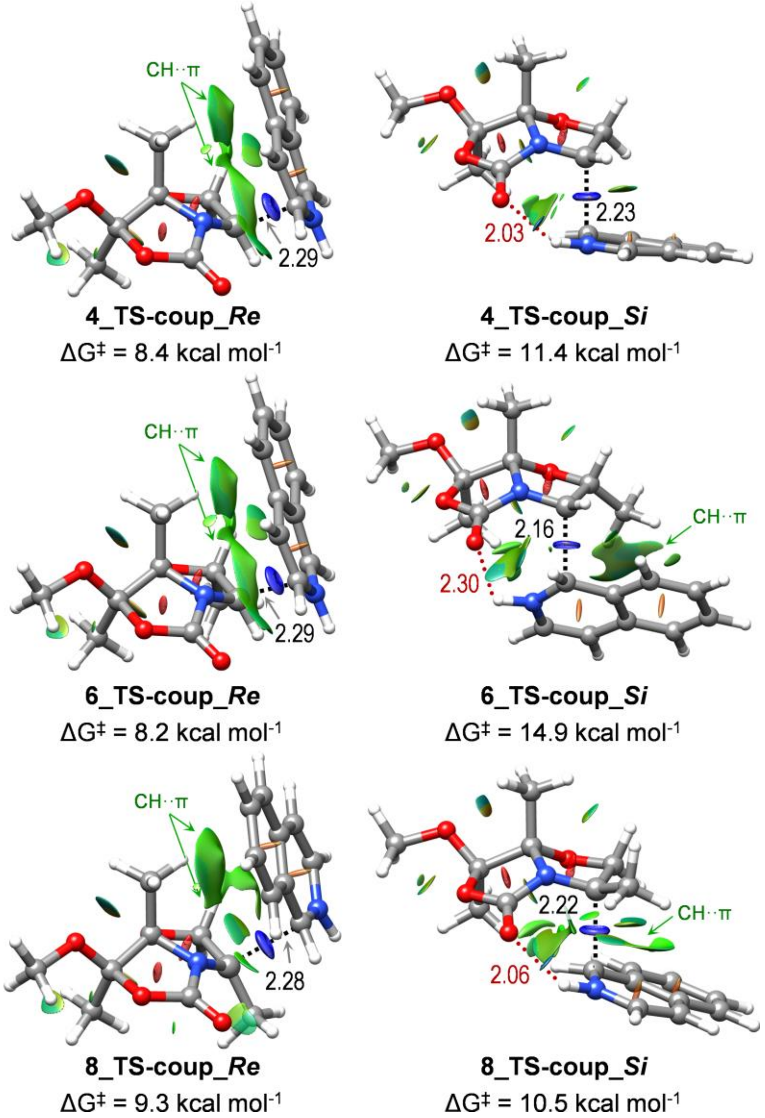

Figure S7. Non-covalent interactions (NCI) occurring in low energy transition states (TS) calculated for the nucleophilic attack of bicyclic C-centered radicals to cationic isoquinolinium. Reduced density gradient (RDG) isosurfaces are plotted at a 0.35 level and color-coded based on $sign(\lambda_2)\rho$: –3.0 (blue; strong attractive interactions) < 0.0 (green; weak van der Waals interactions) < 3.0 (red; strong repulsive interactions). Forming C–C bonds and hydrogen bonds are shown as black and red dashed lines, respectively. Distances are given in angstrom.

Table S5. Energies, entropies, and lowest frequencies of the lowest energy calculated structures.[a]

Structure	E_{elec} (Hartree)	E_{elec} + ZPE (Hartree)	H (Hartree)	S (cal mol^{-1} K^{-1})	G (Hartree)	Lowest freq. (cm^{-1})	# of imag. freq.
**CO_2**	–188.572876	–188.560973	–188.557419	51.0	–188.581653	678.6	0
**isoquinoline**	–402.300489	–402.149591	–402.141853	82.1	–402.180866	167.1	0
**4B_*S***	–856.139377	–855.920685	–855.904838	122.4	–855.961860	54.5	0
**4C_*S***	–667.595267	–667.392398	–667.379055	109.5	–667.430388	48.4	0
**4_TS-*epi***	–667.594522	–667.392753	–667.379685	108.3	–667.430575	–311.1	1
**4C_*R***	–667.594714	–667.392059	–667.378714	108.9	–667.429969	59.8	0
**4_TS-coup_*Re***	–1069.904902	–1069.549203	–1069.528702	143.7	–1069.594809	–291.9	1
**4_TS-coup_*Si***	–1069.900098	–1069.544522	–1069.524042	144.0	–1069.590065	–343.2	1
**4D_*R***	–1069.935503	–1069.576343	–1069.555996	144.0	–1069.621635	35.4	0
**4D_*S***	–1069.934936	–1069.576160	–1069.555631	144.5	–1069.621792	38.7	0
**4_preTS-coup_*Re*·TFA**	–1596.282845	–1595.901669	–1595.872729	187.0	–1595.955990	31.6	0
**4_preTS-coup_*Si* ·TFA**	–1596.277950	–1595.896152	–1595.867062	191.9	–1595.950546	13.0	0
**4_TS-coup_*Re*·TFA**	–1596.268092	–1595.885789	–1595.857592	187.5	–1595.939414	–321.8	1
**4_TS-coup_*Si*·TFA**	–1596.262874	–1595.880552	–1595.852523	186.8	–1595.933868	–352.9	1
**4D_*R*·TFA**	–1596.303556	–1595.917733	–1595.890206	178.8	–1595.970557	28.0	0
**4D_*S*·TFA**	–1596.292835	–1595.908009	–1595.879772	188.5	–1595.961554	13.9	0
**4_TS-deprot_*R*·TFA**	–1596.281372	–1595.899376	–1595.871996	178.9	–1595.951898	–1001.6	1
**4_TS-deprot_*S*·TFA**	–1596.275703	–1595.893899	–1595.866444	177.0	–1595.946683	–1269.4	1
**4E'_*R*·TFA**	–1069.501530	–1069.156980	–1069.136436	144.1	–1069.202543	31.4	0
**4E'_*S*·TFA**	–1069.494852	–1069.150086	–1069.129578	144.0	–1069.195160	27.2	0
**6B_*S***	–895.449902	–895.203424	–895.186097	129.3	–895.245906	39.6	0
**6C_*S***	–706.905663	–706.674884	–706.660095	116.2	–706.714190	42.9	0
**6_TS-*epi***	–706.905111	–706.675290	–706.660827	114.5	–706.714474	–294.6	1
**6C_*R***	–706.905376	–706.674683	–706.659921	115.3	–706.714036	57.6	0
**6_TS-coup_*Re***	–1109.215476	–1108.831979	–1108.809963	150.5	–1108.878966	–278.0	1
**6_TS-coup_*Si***	–1109.205917	–1108.821741	–1108.800112	147.5	–1108.868304	–354.0	1
**6D_*R***	–1109.248548	–1108.861708	–1108.839914	148.6	–1108.908346	37.1	0
**6D_*S***	–1109.242243	–1108.855777	–1108.833886	150.5	–1108.902339	28.0	0
**8B_*S***	–895.443160	–895.196687	–895.179511	127.3	–895.238765	51.7	0
**8C_*S***	–706.906174	–706.675340	–706.660247	119.1	–706.715095	36.0	0
**8_TS-*epi***	–706.905045	–706.674906	–706.660333	116.2	–706.714447	–161.5	1
**8C_*R***	–706.906544	–706.675512	–706.660621	116.5	–706.715156	44.0	0
**8_TS-coup_*Re***	–1109.214900	–1108.831327	–1108.809294	149.6	–1108.878144	–226.3	1
**8_TS-coup_*Si***	–1109.213313	–1108.829657	–1108.807813	148.8	–1108.876268	–329.6	1
**8D_*R***	–1109.239865	–1108.853364	–1108.831654	147.8	–1108.899618	32.3	0
**8D_*S***	–1109.239855	–1108.853249	–1108.831681	146.9	–1108.899487	35.4	0

[a]Energy values calculated at the PCM(DMF)/M06-2X/6-311G(d,p) level. 1 Hartree = 627.51 kcal mol^{-1}. Thermal corrections at 298.15 K.

Table S6. Absolute (ΔG‡) and relative (ΔΔG‡) activation energies, Boltzmann factors and population of all conformers (*conf n*) calculated with PCM(DMF)/M06-2X/6-311G(d,p) for the *Re* and *Si* C–C coupling transition states between bicyclic radicals **4-6C** and cationic isoquinolinium (**TS-coup**). The aggregated population (last column) represents the percentage of each diastereomer obtained under strict kinetic control and Curtin-Hammett conditions at the radical epimerization step.

Structure	ΔG‡ (kcal mol^{-1})	ΔΔG‡ (kcal mol^{-1})	Boltzmann factor	Population (%)	
**4_TS-coup_*Re*** *(conf 1)*	8.4	0.00	1.00	64.7	99.5
**4_TS-coup_*Re*** *(conf 2)*	11.0	2.53	0.01	0.9	
**4_TS-coup_*Re*** *(conf 3)*	9.6	1.16	0.14	9.2	
**4_TS-coup_*Re*** *(conf 4)*	10.2	1.82	0.05	3.0	
**4_TS-coup_*Re*** *(conf 5)*	9.2	0.74	0.29	18.7	
**4_TS-coup_*Re*** *(conf 6)*	10.2	1.82	0.05	3.0	
**4_TS-coup_*Si*** *(conf 1)*	12.9	4.45	0.00	0.0	0.5
**4_TS-coup_*Si*** *(conf 2)*	15.0	6.58	0.00	0.0	
**4_TS-coup_*Si*** *(conf 3)*	11.4	2.98	0.01	0.4	
**4_TS-coup_*Si*** *(conf 4)*	12.6	4.16	0.00	0.1	
**4_TS-coup_*Si*** *(conf 5)*	14.6	6.16	0.00	0.0	
**4_TS-coup_*Si*** *(conf 6)*	13.8	5.38	0.00	0.0	
**6_TS-coup_*Re*** *(conf 1)*	8.2	0.00	1.00	54.8	100.0
**6_TS-coup_*Re*** *(conf 2)*	9.7	1.53	0.08	4.1	
**6_TS-coup_*Re*** *(conf 3)*	9.5	1.26	0.12	6.5	
**6_TS-coup_*Re*** *(conf 4)*	10.0	1.76	0.05	2.8	
**6_TS-coup_*Re*** *(conf 5)*	8.6	0.39	0.52	28.5	
**6_TS-coup_*Re*** *(conf 6)*	9.9	1.65	0.06	3.4	
**6_TS-coup_*Si*** *(conf 1)*	16.9	8.68	0.00	0.0	0.0
**6_TS-coup_*Si*** *(conf 2)*	16.9	8.71	0.00	0.0	
**6_TS-coup_*Si*** *(conf 3)*	19.2	10.96	0.00	0.0	
**6_TS-coup_*Si*** *(conf 4)*	14.9	6.69	0.00	0.0	
**6_TS-coup_*Si*** *(conf 5)*	22.1	13.88	0.00	0.0	
**6_TS-coup_*Si*** *(conf 6)*	28.3	20.06	0.00	0.0	
**8_TS-coup_*Re*** *(conf 1)*	9.3	0.04	0.93	25.3	96.0
**8_TS-coup_*Re*** *(conf 2)*	12.4	3.13	0.01	0.1	
**8_TS-coup_*Re*** *(conf 3)*	11.6	2.28	0.02	0.6	
**8_TS-coup_*Re*** *(conf 4)*	9.6	0.32	0.58	15.9	
**8_TS-coup_*Re*** *(conf 5)*	11.1	1.77	0.05	1.4	
**8_TS-coup_*Re*** *(conf 6)*	9.3	0.00	1.00	27.2	
**8_TS-coup_*Re*** *(conf 7)*	9.3	0.04	0.94	25.5	
**8_TS-coup_*Si*** *(conf 1)*	12.6	3.28	0.00	0.1	4.0
**8_TS-coup_*Si*** *(conf 2)*	14.9	5.59	0.00	0.0	
**8_TS-coup_*Si*** *(conf 3)*	10.5	1.18	0.14	3.7	
**8_TS-coup_*Si*** *(conf 4)*	13.0	3.70	0.00	0.1	
**8_TS-coup_*Si*** *(conf 5)*	12.9	3.58	0.00	0.1	
**8_TS-coup_*Si*** *(conf 6)*	13.1	3.85	0.00	0.0	

Table S7. Absolute (ΔG‡) and relative (ΔΔG‡) activation energies, Boltzmann factors and population of all conformers (*conf n*) calculated with PCM(DMF)/M06-2X/6-311G(d,p) for the *Re* and *Si* C–C coupling transition states between Ser-derived bicyclic radicals **4C** and isoquinolinium-trifluoroacetate ion pair (**4_TS-coup·TFA**). The aggregated population (last column) represents the percentage of each diastereomer obtained under strict kinetic control and Curtin-Hammett conditions at the radical epimerization step.

Structure	ΔG‡ (kcal mol^{-1})	ΔΔG‡ (kcal mol^{-1})	Boltzmann factor	Population (%)	
**4_TS-coup_*Re*·TFA** *(conf 1)*	10.8	0.2	0.71	17.4	99.8
**4_TS-coup_*Re*·TFA** *(conf 2)*	11.3	0.7	0.32	8.0	
**4_TS-coup_*Re*·TFA** *(conf 3)*	10.6	0.0	1.00	24.7	
**4_TS-coup_*Re*·TFA** *(conf 4)*	11.0	0.4	0.49	12.0	
**4_TS-coup_*Re*·TFA** *(conf 5)*	10.8	0.2	0.71	17.7	
**4_TS-coup_*Re*·TFA** *(conf 6)*	10.7	0.1	0.81	20.0	
**4_TS-coup_*Si*·TFA** *(conf 1)*	20.2	9.6	0.00	0.0	0.2
**4_TS-coup_*Si*·TFA** *(conf 2)*	14.1	3.5	0.00	0.1	
**4_TS-coup_*Si*·TFA** *(conf 3)*	14.1	3.5	0.00	0.1	
**4_TS-coup_*Si*·TFA** *(conf 4)*	15.3	4.7	0.00	0.0	
**4_TS-coup_*Si*·TFA** *(conf 5)*	15.3	4.7	0.00	0.0	
**4_TS-coup_*Si*·TFA** *(conf 6)*	22.8	12.2	0.00	0.0	
**4_TS-coup_*Si*·TFA** *(conf 7)*	16.8	6.2	0.00	0.0	

Table S8. Absolute ($\Delta G^{\ddagger}$) and relative ($\Delta\Delta G^{\ddagger}$) activation energies, Boltzmann factors and population of all conformers (*conf n*) calculated with PCM(DMF)/M06-2X/6-311G(d,p) for Ser-derived bicyclic *(R)* and *(S)* deprotonation transition states (**4_TS-deprot·TFA**). The aggregated population (last column) represents the percentage of each diastereomer hypothetically (and incorrectly) assuming reversible radical C-C coupling and Curtin-Hammett conditions for the whole reaction pathway. Note that even in this scenario the reaction is completely diastereoselective towards the experimentally observed isomer.

Structure	$\Delta G^{\ddagger}$ (kcal mol^{-1})	$\Delta\Delta G^{\ddagger}$ (kcal mol^{-1})	Boltzmann factor	Population (%)	
**4_TS-deprot_*R*·TFA** *(conf 1)*	19.7	7.97	0.00	0.0	
**4_TS-deprot_*R*·TFA** *(conf 2)*	16.8	5.12	0.00	0.0	
**4_TS-deprot_*R*·TFA** *(conf 3)*	18.6	6.94	0.00	0.0	
**4_TS-deprot_*R*·TFA** *(conf 4)*	14.8	3.12	0.01	0.4	
**4_TS-deprot_*R*·TFA** *(conf 5)*	12.8	1.06	0.17	13.4	
**4_TS-deprot_*R*·TFA** *(conf 6)*	11.7	0.00	1.00	79.5	
**4_TS-deprot_*R*·TFA** *(conf 7)*	20.1	8.39	0.00	0.0	
**4_TS-deprot_*R*·TFA** *(conf 8)*	17.9	6.21	0.00	0.0	
**4_TS-deprot_*R*·TFA** *(conf 9)*	16.2	4.47	0.00	0.0	
**4_TS-deprot_*R*·TFA** *(conf 10)*	21.1	9.36	0.00	0.0	99.6
**4_TS-deprot_*R*·TFA** *(conf 11)*	16.5	4.82	0.00	0.0	
**4_TS-deprot_*R*·TFA** *(conf 12)*	15.5	3.80	0.00	0.1	
**4_TS-deprot_*R*·TFA** *(conf 13)*	14.5	2.75	0.01	0.8	
**4_TS-deprot_*R*·TFA** *(conf 14)*	18.2	6.53	0.00	0.0	
**4_TS-deprot_*R*·TFA** *(conf 15)*	16.9	5.21	0.00	0.0	
**4_TS-deprot_*R*·TFA** *(conf 16)*	14.1	2.38	0.02	1.4	
**4_TS-deprot_*R*·TFA** *(conf 17)*	13.6	1.86	0.04	3.4	
**4_TS-deprot_*R*·TFA** *(conf 18)*	15.2	3.53	0.00	0.2	
**4_TS-deprot_*R*·TFA** *(conf 19)*	15.5	3.80	0.00	0.1	
**4_TS-deprot_*S*·TFA** *(conf 1)*	24.1	12.36	0.00	0.0	
**4_TS-deprot_*S*·TFA** *(conf 2)*	22.4	10.72	0.00	0.0	
**4_TS-deprot_*S*·TFA** *(conf 3)*	18.7	6.97	0.00	0.0	
**4_TS-deprot_*S*·TFA** *(conf 4)*	15.6	3.94	0.00	0.1	
**4_TS-deprot_*S*·TFA** *(conf 5)*	16.7	5.02	0.00	0.0	
**4_TS-deprot_*S*·TFA** *(conf 6)*	19.5	7.82	0.00	0.0	
**4_TS-deprot_*S*·TFA** *(conf 7)*	17.9	6.15	0.00	0.0	
**4_TS-deprot_*S*·TFA** *(conf 8)*	19.1	7.41	0.00	0.0	
**4_TS-deprot_*S*·TFA** *(conf 9)*	25.7	13.98	0.00	0.0	
**4_TS-deprot_*S*·TFA** *(conf 10)*	20.7	9.01	0.00	0.0	0.4
**4_TS-deprot_*S*·TFA** *(conf 11)*	26.2	14.54	0.00	0.0	
**4_TS-deprot_*S*·TFA** *(conf 12)*	18.0	6.29	0.00	0.0	
**4_TS-deprot_*S*·TFA** *(conf 13)*	15.0	3.27	0.00	0.3	
**4_TS-deprot_*S*·TFA** *(conf 14)*	18.0	6.26	0.00	0.0	
**4_TS-deprot_*S*·TFA** *(conf 15)*	22.7	10.96	0.00	0.0	
**4_TS-deprot_*S*·TFA** *(conf 16)*	20.3	8.64	0.00	0.0	
**4_TS-deprot_*S*·TFA** *(conf 17)*	21.3	9.59	0.00	0.0	
**4_TS-deprot_*S*·TFA** *(conf 18)*	19.9	8.15	0.00	0.0	
**4_TS-deprot_*S*·TFA** *(conf 19)*	18.9	7.19	0.00	0.0	

Cartesian coordinates of the lowest energy structures calculated with PCM(DMF)/M06-2X/6-311G(d,p)

Structure **CO_2**

C	0.000000	0.000000	0.000000
O	0.000000	0.000000	1.154360
O	0.000000	0.000000	−1.154360

Structure **isoquinoline**

C	1.200982	−1.355609	−0.000001
C	−0.032930	−0.692029	0.000009
C	2.373381	0.704647	0.000026
H	3.207590	−1.170958	0.000200
C	−0.033067	0.731215	−0.000019
C	1.212919	1.410002	−0.000002
H	3.357515	1.146792	−0.000020
H	1.244812	2.491211	−0.000052
H	1.283678	−2.434195	−0.000293
N	2.327459	−0.663157	−0.000028
C	−1.271516	1.411748	0.000000
C	−2.440755	0.696052	−0.000004
H	−3.389004	1.219215	0.000022
C	−2.436755	−0.721381	−0.000012
H	−3.378144	−1.255220	−0.000120
C	−1.256179	−1.410606	0.000046
H	−1.279158	2.494586	0.000044
H	−1.235982	−2.493562	0.000151

Structure **4B_*S***

O	−0.203832	−0.946817	−1.502307
O	1.587113	1.200612	−0.403821
O	−0.035879	2.746760	−0.330483
N	−0.469622	0.531269	0.186018
C	1.706816	−0.225024	−0.175054
C	−2.827171	0.004761	0.566062
C	0.327823	1.615801	−0.194171
O	−3.900884	−0.470540	0.309825
C	−1.579611	−0.583926	−1.581737
H	−1.756721	−0.146649	−2.560870
H	−2.228916	−1.451401	−1.444400
C	0.233648	−0.696795	−0.174744
C	−1.783753	0.451857	−0.434705
O	2.177747	−0.429341	1.110066
C	3.524264	−0.025940	1.349574
H	4.229456	−0.724638	0.895113
H	3.653026	−0.034769	2.428943
H	3.700269	0.983090	0.971308
C	2.568912	−0.806316	−1.271302
H	2.734169	−1.867445	−1.077730
H	3.530051	−0.294182	−1.302859
H	2.069815	−0.686133	−2.229741
C	−0.068469	−1.868547	0.735002
H	0.144137	−1.611949	1.769706
H	0.543766	−2.721153	0.438683
H	−1.117719	−2.157712	0.640124
O	−2.467227	0.194576	1.823364
H	−2.085042	1.436835	−0.797110

Structure **4C_*S***

O	1.040481	−1.506866	0.629131
O	−0.837305	1.319214	−0.092189
O	0.877627	2.781268	−0.208395
N	1.192510	0.501742	−0.394391
C	−0.962763	−0.103940	0.239383
C	0.464753	1.655586	−0.226414
C	2.440196	−1.181832	0.570852
H	2.844824	−1.240515	1.582489
H	2.962830	−1.912428	−0.057370
C	0.367736	−0.698673	−0.308853
C	2.491986	0.214346	0.003106
O	−2.012448	−0.627681	−0.474736
C	−3.312003	−0.182160	−0.092622
H	−3.617936	−0.628750	0.855531
H	−3.985657	−0.511523	−0.879685
H	−3.341682	0.906837	−0.020149
C	−1.092929	−0.211085	1.744547
H	−1.267968	−1.249948	2.024902
H	−1.915920	0.411978	2.092965
H	−0.171417	0.126059	2.219013
C	0.239868	−1.399551	−1.647434
H	−0.247946	−0.749810	−2.373539
H	−0.350577	−2.307172	−1.530382
H	1.238523	−1.656076	−2.006315
H	3.137459	1.015528	0.327395

Structure **4_TS-*epi***

O	1.037401	−1.517093	0.615737
O	−0.850673	1.311980	−0.123776
O	0.850089	2.794792	−0.195627
N	1.197278	0.516977	−0.365302
C	−0.961571	−0.106787	0.233675
C	0.452085	1.663799	−0.220353
C	2.437941	−1.202002	0.557920
H	2.856510	−1.341879	1.556109
H	2.947579	−1.889316	−0.130572
C	0.372939	−0.688648	−0.310453
C	2.472619	0.224550	0.096458
O	−2.008558	−0.653440	−0.466163
C	−3.310914	−0.217458	−0.082362
H	−3.604256	−0.650636	0.875945
H	−3.986079	−0.569319	−0.858298
H	−3.353637	0.872208	−0.029115
C	−1.080728	−0.191582	1.741317
H	−1.248990	−1.226826	2.038512
H	−1.903584	0.433285	2.086794
H	−0.156898	0.156328	2.204066
C	0.258890	−1.357282	−1.666467
H	−0.228986	−0.693086	−2.379528
H	−0.326527	−2.271159	−1.575040
H	1.261036	−1.598265	−2.026066
H	3.289245	0.922377	0.054532

Structure **4C_*R***

O	1.033787	−1.525974	0.605091
O	−0.851931	1.309073	−0.136567
O	0.843341	2.796533	−0.197145
N	1.202896	0.518968	−0.354830
O	−2.008396	−0.660576	−0.460786
C	−0.960015	−0.107931	0.231483
C	0.260418	−1.338760	−1.677378
H	−0.224005	−0.664197	−2.383134
H	−0.328832	−2.251339	−1.597872
H	1.261732	−1.578947	−2.039296
C	2.463540	0.241355	0.168008

C	0.449893	1.664651	−0.222695
C	2.432682	−1.211149	0.546927
H	2.863604	−1.408726	1.529174
H	2.934917	−1.854185	−0.188803
C	0.374207	−0.686610	−0.313203
C	−3.310238	−0.226443	−0.072837
H	−3.988319	−0.585546	−0.842870
H	−3.356260	0.863400	−0.026366
H	−3.596915	−0.654389	0.889818
C	−1.072208	−0.182847	1.740199
H	−1.240839	−1.215920	2.044573
H	−1.892025	0.445752	2.086105
H	−0.145184	0.166065	2.196168
H	3.327764	0.839211	−0.071446

Structure **4_TS-coup_*Re***

O	−1.178619	−1.059636	1.825746
O	−2.493870	0.911466	−0.710676
O	−1.209916	2.731464	−0.367137
N	−0.632330	0.653331	0.457396
C	−2.650380	−0.402535	−0.053524
C	−1.433286	1.564599	−0.229038
C	−0.077580	−0.368936	2.431957
H	−0.326716	−0.160096	3.470454
H	0.822642	−0.992863	2.394352
C	−1.208871	−0.698903	0.465995
C	0.069508	0.896903	1.615277
O	−2.972171	−1.323144	−1.011930
C	−4.279320	−1.217044	−1.577106
H	−5.039887	−1.547590	−0.867732
H	−4.282679	−1.873215	−2.443360
H	−4.480328	−0.192152	−1.894554
C	−3.667246	−0.228535	1.053689
H	−3.871351	−1.194515	1.515946
H	−4.587312	0.192814	0.650640
H	−3.276468	0.446040	1.815204
C	−0.429038	−1.683140	−0.379870
H	−0.358212	−1.338797	−1.411867
H	−0.934155	−2.647518	−0.360516
H	0.573929	−1.794700	0.036504
H	−0.004138	1.887962	2.047196
C	2.196871	1.367069	0.915794
C	2.592869	0.134962	0.316948
C	2.434612	−0.019325	−1.083690
C	1.684222	2.267219	−1.235281
H	1.707063	3.306197	0.527986
C	1.932033	1.080521	−1.844607
H	1.339917	3.151289	−1.749948
H	1.781018	0.982571	−2.910929
H	2.469661	1.612438	1.933890
C	2.802809	−1.242438	−1.681118
C	3.304278	−2.263766	−0.908874
H	3.584393	−3.201592	−1.372163
C	3.466567	−2.104072	0.483225
H	3.874353	−2.915666	1.071950
C	3.118185	−0.921360	1.091675
H	3.259623	−0.783741	2.157549
H	2.682939	−1.362715	−2.751123
N	1.884461	2.400574	0.108857

Structure **4_TS-coup_*Si***

O	1.104750	−1.584055	0.823279
O	2.779653	1.080893	−0.656170
O	1.091214	2.564603	−0.572234
N	1.123459	0.663802	0.741781
C	2.931082	−0.358299	−0.332321
C	1.617310	1.549653	−0.195768
C	−0.118577	−1.134386	1.410012
H	−0.939516	−1.721158	0.998889
H	−0.107039	−1.266038	2.498486
C	2.001109	−0.504171	0.903628
C	−0.189150	0.334562	1.055769
O	4.223288	−0.576115	0.051332
C	5.210306	−0.497093	−0.978538
H	5.157702	−1.365188	−1.637698
H	6.170126	−0.486410	−0.469334
H	5.092280	0.422124	−1.555252
C	2.470950	−1.143988	−1.539864
H	2.656116	−2.205255	−1.371873
H	3.002175	−0.809869	−2.429895
H	1.402483	−1.002750	−1.695097
C	2.733767	−0.469092	2.230639
H	3.380658	0.405833	2.284242
H	3.344028	−1.365956	2.326123
H	2.010609	−0.429348	3.046082
H	−0.798003	1.028909	1.625631
C	−1.425388	0.772173	−0.751605
C	−2.489840	−0.170025	−0.603250
C	−3.664032	0.246014	0.074763
C	−2.796210	2.499000	0.149519
H	−0.930556	2.736355	−0.698982
C	−3.773142	1.610375	0.479727
H	−2.844312	3.554441	0.372689
H	−4.652643	1.953176	1.007418
H	−0.596991	0.572547	−1.420873
C	−4.697498	−0.690523	0.288219
C	−4.566050	−1.977828	−0.175195
H	−5.363240	−2.692606	−0.013765
C	−3.406207	−2.380146	−0.869913
H	−3.328041	−3.394474	−1.239592
C	−2.377146	−1.492449	−1.078096
H	−1.484084	−1.796916	−1.611256
H	−5.592734	−0.376476	0.811002
N	−1.686025	2.078166	−0.513632

Structure **4D_*R***

O	1.069623	0.015677	1.689160
O	3.065029	−1.298748	−0.311749
O	1.518370	−2.349262	−1.543753
N	1.055984	−0.313480	−0.554074
C	3.179920	−0.069868	0.440424
C	1.857039	−1.412417	−0.880917
C	−0.303932	−0.125432	1.352363
H	−0.758515	−0.820296	2.053714
H	−0.823745	0.837278	1.416681
C	1.717856	0.455458	0.508943
C	−0.291408	−0.672934	−0.094876
O	3.889723	0.840753	−0.323265
C	5.266667	0.535998	−0.533957
H	5.851904	0.722093	0.368664
H	5.603496	1.201242	−1.324992
H	5.390874	−0.500411	−0.854082
C	3.798695	−0.393717	1.781077
H	3.962943	0.530449	2.337580
H	4.751578	−0.902477	1.639579
H	3.130277	−1.039598	2.345241

C	1.617817	1.963731	0.368501
H	1.945788	2.282454	−0.618584
H	2.260304	2.430470	1.115997
H	0.596273	2.298991	0.546559
H	−0.391144	−1.758950	−0.101497
C	−1.398189	−0.122173	−1.026128
C	−2.760104	−0.331004	−0.420548
C	−3.548838	0.773891	−0.014989
C	−1.892137	2.288351	−0.973110
H	−0.263813	1.458549	−1.826222
C	−3.092186	2.088788	−0.287247
H	−1.545960	3.278635	−1.240774
H	−3.677629	2.950381	0.001524
H	−1.318830	−0.671163	−1.970323
C	−4.793351	0.549904	0.612237
C	−5.241315	−0.736827	0.819856
H	−6.195180	−0.910517	1.300414
C	−4.464125	−1.821196	0.397375
H	−4.823459	−2.831322	0.548181
C	−3.232266	−1.619011	−0.218801
H	−2.649151	−2.471448	−0.547554
H	−5.386956	1.402924	0.918353
N	−1.130536	1.275780	−1.327759

Structure **4D_*S***

O	1.324988	−1.883775	0.040120
O	2.833827	0.468660	−1.143822
O	1.045362	1.669353	−1.734949
N	1.032929	0.365335	0.182480
C	3.240861	−0.370826	−0.031602
C	1.581016	0.900364	−0.974969
C	−0.070052	−1.670101	−0.174693
H	−0.276420	−1.853319	−1.231426
H	−0.659551	−2.353377	0.437437
C	1.890214	−0.723664	0.638974
C	−0.327676	−0.190163	0.207013
O	3.932493	0.413266	0.873334
C	5.210881	0.879981	0.446087
H	5.947602	0.074621	0.464149
H	5.501284	1.650257	1.155968
H	5.152138	1.312390	−0.554781
C	4.015028	−1.543141	−0.585876
H	4.390715	−2.150145	0.239490
H	4.855974	−1.188440	−1.180941
H	3.364092	−2.147557	−1.212407
C	1.949886	−0.877158	2.143835
H	2.280299	0.047212	2.610772
H	2.649318	−1.676629	2.392042
H	0.966212	−1.155054	2.525368
H	−0.684573	−0.114237	1.236495
C	−1.364063	0.525690	−0.691002
C	−2.728702	−0.063066	−0.430545
C	−3.590477	0.571052	0.499235
C	−2.147597	2.537957	0.458188
H	−0.543959	2.454239	−0.794685
C	−3.248544	1.856406	0.993843
H	−1.953999	3.577516	0.689037
H	−3.881509	2.363900	1.708229
H	−1.086136	0.413908	−1.740098
C	−4.816055	−0.038482	0.840661
C	−5.181757	−1.233225	0.255231
H	−6.122901	−1.700337	0.514170
C	−4.342672	−1.830034	−0.689573
H	−4.640821	−2.754779	−1.167106
C	−3.122796	−1.246858	−1.030535
H	−2.491723	−1.721504	−1.772141
H	−5.467556	0.454788	1.551882
N	−1.329618	1.951541	−0.381701

Structure **4_preTS-coup_*Re*·TFA**

O	1.435134	-3.001118	-0.335666
O	-1.195389	-1.880511	0.934199
O	-0.220127	-0.749576	2.606464
N	0.834417	-1.014633	0.561501
C	-0.899451	-2.236350	-0.440326
C	-0.194398	-1.175276	1.485859
C	2.614935	-2.500761	0.310189
H	2.946703	-3.247447	1.033118
H	3.412373	-2.338009	-0.423531
C	0.605351	-1.879300	-0.593302
C	2.170519	-1.214642	0.964934
O	-1.602619	-1.374994	-1.269886
C	-3.020438	-1.517738	-1.252913
H	-3.325250	-2.500519	-1.616190
H	-3.405326	-0.757355	-1.927659
H	-3.414983	-1.347182	-0.246623
C	-1.223174	-3.702456	-0.620616
H	-1.117160	-3.970507	-1.673132
H	-2.241440	-3.908796	-0.294379
H	-0.533498	-4.297825	-0.027608
C	0.989529	-1.229057	-1.906220
H	0.381192	-0.349120	-2.096441
H	0.851486	-1.948028	-2.715088
H	2.037352	-0.926838	-1.860680
H	2.472083	-0.859871	1.937494
C	-0.997589	1.557932	0.360765
C	-2.384567	1.408595	0.553330
C	-1.306412	2.111377	-1.898230
H	0.605141	1.902464	-0.926077
C	-3.244518	1.667731	-0.546554
C	-2.660354	2.029403	-1.788260
H	-0.796080	2.363438	-2.817222
H	-3.286948	2.224263	-2.649112
H	-0.270216	1.419710	1.154941
N	-0.508337	1.885223	-0.816204
C	-4.641712	1.526529	-0.368982
C	-5.137892	1.128803	0.845159
H	-6.206957	1.014589	0.977107
C	-4.274963	0.856438	1.936348
H	-4.697020	0.535637	2.880230
C	-2.920483	0.997209	1.800533
H	-2.243174	0.780750	2.618247
O	2.034520	1.854980	1.211605
C	2.534207	1.659405	0.112193
O	2.019490	1.748794	-1.035816
C	4.031435	1.266381	0.056320
9	4.252412	0.243924	-0.785723
9	4.770120	2.298623	-0.383506
9	4.515662	0.911386	1.248234
H	-5.302810	1.725176	-1.204078

Structure **4_preTS-coup_*Si*·TFA**

O	-2.417439	0.148518	1.439187
O	-1.713331	-2.028532	-0.980520
O	0.519169	-1.923423	-0.821219
N	-0.844277	-1.414644	0.975914

C	-2.880907	-1.654411	-0.194496
C	-0.567047	-1.788955	-0.325775
C	-1.245757	0.544598	2.159838
H	-1.048083	1.593153	1.935617
H	-1.405845	0.444271	3.241722
C	-2.280267	-1.236191	1.183312
C	-0.173247	-0.381385	1.652874
O	-3.643282	-2.789666	0.008463
C	-4.270062	-3.336270	-1.147729
H	-5.128048	-2.734126	-1.453634
H	-4.611304	-4.328913	-0.863603
H	-3.559947	-3.420025	-1.973015
C	-3.584304	-0.538480	-0.930127
H	-4.478053	-0.239491	-0.380235
H	-3.863360	-0.869395	-1.930406
H	-2.905467	0.304950	-1.014335
C	-2.784862	-2.045095	2.364728
H	-2.618561	-3.107663	2.201976
H	-3.851731	-1.867730	2.499979
H	-2.251957	-1.731867	3.264197
H	0.804709	-0.526706	2.084596
C	2.471087	0.385239	-0.391018
C	3.646758	-0.389398	-0.423866
C	3.513514	2.017538	0.943590
H	1.484088	2.074737	0.260325
C	4.791488	0.097070	0.262302
C	4.689132	1.333647	0.953765
H	3.364558	2.960843	1.448999
H	5.541945	1.731136	1.488305
H	1.551798	0.082676	-0.881457
N	2.434572	1.524180	0.268860
C	5.981618	-0.668008	0.230135
C	6.010041	-1.855362	-0.454583
H	6.922083	-2.439369	-0.478122
C	4.863806	-2.339367	-1.132666
H	4.917750	-3.283881	-1.658990
C	3.697468	-1.623678	-1.120242
H	2.802041	-1.978766	-1.614761
O	-0.593781	1.087553	-1.079262
C	-0.651027	2.186588	-0.538855
O	0.195973	2.782649	0.176295
C	-1.956762	3.007581	-0.700486
9	-1.702481	4.290571	-0.985084
9	-2.748746	2.537976	-1.669079
9	-2.666583	2.989413	0.439898
H	6.859107	-0.302990	0.750063

Structure **4_TS-coup_*Re*·TFA**

O	-1.889349	-0.960632	-2.063138
O	-1.640276	-2.464105	1.052219
O	0.393799	-1.665491	1.601578
N	-0.897269	-0.695778	-0.050661
C	-2.514203	-2.375664	-0.130092
C	-0.604670	-1.619966	0.944125
C	-0.760780	-0.094712	-2.258098
H	-0.168565	-0.479284	-3.086680
H	-1.105319	0.917193	-2.500702
C	-2.193276	-0.955033	-0.688671
C	-0.004681	-0.146538	-0.948196
O	-3.815821	-2.401111	0.295961
C	-4.280199	-3.642733	0.824844
H	-4.427664	-4.376474	0.030336
H	-5.234100	-3.426963	1.298966
H	-3.582242	-4.030463	1.569338
C	-2.115864	-3.495726	-1.067492
H	-2.794104	-3.516481	-1.920732
H	-2.149146	-4.449788	-0.542762
H	-1.102472	-3.330777	-1.432998
C	-3.235332	0.095771	-0.365544
H	-3.376643	0.183912	0.712071
H	-4.180785	-0.183634	-0.827989
H	-2.907392	1.056438	-0.767498
H	1.041137	-0.426381	-0.881734
C	0.577646	1.845988	-0.089425
C	-0.645873	2.584889	-0.147517
C	-1.464520	2.627405	1.008379
C	0.223711	1.396722	2.209439
H	1.982000	0.954730	1.160272
C	-1.007876	1.974695	2.193830
H	0.647481	0.923835	3.084158
H	-1.620942	1.971411	3.084992
H	1.331312	1.914944	-0.865239
C	-2.688702	3.327531	0.953101
C	-3.075464	3.953434	-0.208691
H	-4.017240	4.486972	-0.246260
C	-2.254618	3.915705	-1.354796
H	-2.569093	4.424734	-2.257020
C	-1.054747	3.243163	-1.326118
H	-0.408851	3.224586	-2.196760
N	1.012171	1.400167	1.103086
O	3.109063	0.296038	-1.017953
C	3.739076	0.083794	0.022474
O	3.428668	0.322415	1.207071
C	5.117348	-0.591913	-0.190410
9	5.816915	-0.743013	0.936431
9	5.875186	0.120381	-1.038255
9	4.966111	-1.811506	-0.732571
H	-3.316118	3.360239	1.836060

Structure **4_TS-coup_*Si*·TFA**

O	-1.737399	-0.528184	1.946103
O	-2.586198	-1.494676	-1.250069
O	-0.553063	-1.306835	-2.202400
N	-0.825359	-1.402768	0.086088
C	-3.171667	-1.377418	0.096724
C	-1.247708	-1.399492	-1.229543
C	-0.322144	-0.352164	2.047963
H	-0.126863	0.667066	2.375863
H	0.105675	-1.041312	2.786369
C	-1.936161	-1.574052	1.025017
C	0.199002	-0.660889	0.660226
O	-4.027854	-2.436240	0.275150
C	-5.224684	-2.405781	-0.500553
H	-5.913896	-1.644811	-0.129542
H	-5.677553	-3.387624	-0.389950
H	-4.997986	-2.225095	-1.553078
C	-3.815465	-0.015489	0.225980
H	-4.401418	0.011691	1.146105
H	-4.459725	0.183095	-0.630217
H	-3.049821	0.754606	0.281210
C	-1.884353	-2.930098	1.704876
H	-1.972820	-3.728826	0.970034
H	-2.707485	-3.010013	2.413721
H	-0.935857	-3.034236	2.233267
H	1.229592	-0.928534	0.451354
C	0.603546	1.242439	-0.368481

C	-0.649370	1.916369	-0.576396
C	-1.053814	2.895079	0.367843
C	1.099885	2.704320	1.435118
H	2.476740	1.380040	0.558325
C	-0.149740	3.248861	1.415836
H	1.858993	2.993183	2.149176
H	-0.427087	3.995756	2.147311
H	1.053220	0.649173	-1.157458
C	-2.286915	3.556530	0.181983
C	-3.048555	3.298887	-0.934821
H	-3.984540	3.823862	-1.081404
C	-2.612998	2.372090	-1.901537
H	-3.209564	2.201158	-2.788849
C	-1.438410	1.677442	-1.720385
H	-1.099460	0.963442	-2.460396
N	1.469919	1.764149	0.529907
O	3.915313	0.820990	0.563802
C	3.928160	-0.182009	-0.186263
O	3.008372	-0.706671	-0.813377
C	5.333201	-0.822046	-0.312566
9	5.806817	-1.185171	0.889445
9	6.211536	0.049614	-0.832666
9	5.342557	-1.907353	-1.088544
H	-2.604463	4.291359	0.912280

Structure **4D_*R*·TFA**

O	-0.188823	-2.570747	0.488672
O	2.150588	-1.671161	-1.264766
O	0.889161	-0.458068	-2.659141
N	0.386915	-0.574203	-0.402260
C	2.172665	-1.986363	0.139651
C	1.129444	-0.845019	-1.552025
C	-1.428248	-1.908173	0.258327
H	-2.087850	-2.593498	-0.268197
H	-1.896774	-1.619816	1.205863
C	0.774704	-1.536395	0.633899
C	-1.061196	-0.668639	-0.599106
O	3.082171	-1.150528	0.771009
C	4.437948	-1.279230	0.358457
H	4.890229	-2.184867	0.769479
H	4.951826	-0.405002	0.750861
H	4.514023	-1.281335	-0.730405
C	2.462263	-3.461999	0.291490
H	2.547910	-3.707619	1.351141
H	3.396367	-3.713161	-0.210691
H	1.653515	-4.040623	-0.149561
C	0.791614	-1.011109	2.056976
H	1.344915	-0.077867	2.109647
H	1.264123	-1.758117	2.696933
H	-0.224582	-0.853130	2.416377
H	-1.253255	-0.859530	-1.655523
C	-1.822132	0.640323	-0.261406
C	-3.302819	0.409354	-0.405931
C	-4.135780	0.377713	0.737022
C	-2.243148	1.126051	2.087962
H	-0.439292	1.536056	1.156040
C	-3.578130	0.706815	2.003099
H	-1.827751	1.485596	3.022609
H	-4.187576	0.696831	2.896026
H	-1.462211	1.388002	-0.975600
C	-5.506254	0.076927	0.592938
C	-6.032823	-0.175709	-0.656713
H	-7.084644	-0.405878	-0.767365
C	-5.206535	-0.123154	-1.784548
H	-5.623600	-0.307418	-2.766689
C	-3.851688	0.166266	-1.658056
H	-3.224891	0.210266	-2.541519
H	-6.134909	0.056589	1.475265
N	-1.442532	1.128091	1.053155
O	0.683336	2.495955	-0.946618
C	1.346135	2.243792	0.054188
O	0.976428	2.059003	1.240184
C	2.871147	2.108259	-0.192540
9	3.579764	1.865494	0.912393
9	3.119255	1.105467	-1.053977
9	3.370742	3.224973	-0.747060

Structure **4D_*S*·TFA**

O	0.467707	-1.832039	1.612899
O	1.599518	-2.399378	-1.130448
O	0.853424	-0.533895	-2.096967
N	1.599137	-0.520436	0.110869
C	2.174652	-2.789256	0.135001
C	1.300119	-1.090362	-1.131479
C	-0.384061	-0.732572	1.273856
H	-1.079853	-1.028659	0.481946
H	-0.936065	-0.422018	2.159328
C	1.770743	-1.618328	1.063018
C	0.587410	0.336648	0.753289
O	3.555908	-2.744858	0.023169
C	4.141476	-3.723627	-0.831649
H	4.138724	-4.708712	-0.360591
H	5.168330	-3.405692	-0.994535
H	3.619182	-3.766174	-1.789661
C	1.613492	-4.138970	0.516635
H	2.088962	-4.480775	1.437598
H	1.805737	-4.862626	-0.274955
H	0.540901	-4.056928	0.672837
C	2.757378	-1.341449	2.176723
H	3.734668	-1.094787	1.769676
H	2.843599	-2.231715	2.802099
H	2.393498	-0.524438	2.799772
H	1.083783	0.825171	1.595965
C	-0.032369	1.459144	-0.112219
C	0.997846	2.521296	-0.417191
C	-1.100411	3.079554	1.361791
H	-2.062766	1.366341	0.719276
C	1.037909	3.701585	0.366157
C	0.000810	3.946087	1.305419
H	-1.970765	3.317048	1.962725
H	0.007348	4.835323	1.920320
H	-0.454368	1.024436	-1.019581
N	-1.152847	1.971907	0.671014
C	2.061363	4.648885	0.154793
C	3.012235	4.436519	-0.821525
H	3.796579	5.164292	-0.985649
C	2.950776	3.282308	-1.607805
H	3.687343	3.122169	-2.385458
C	1.953867	2.330859	-1.405165
H	2.078082	5.546590	0.761501
H	1.914887	1.442112	-2.020960
O	-2.639521	-0.139901	-1.230768
C	-3.426280	-0.059177	-0.294385
O	-3.320993	0.573661	0.788570
C	-4.749825	-0.856409	-0.392397
9	-5.814159	-0.061124	-0.209908

9	-4.901897	-1.463201	-1.570449
9	-4.800938	-1.805273	0.556615

Structure **4_TS-deprot_*R*·TFA**

O	0.697539	-2.720961	-1.028861
O	3.019030	-0.673796	-0.914329
O	1.765672	1.124559	-1.364519
N	0.907919	-0.648094	-0.151009
C	2.873987	-1.934372	-0.224878
C	1.886651	0.047168	-0.852084
C	-0.607829	-2.162688	-1.112852
H	-0.973351	-2.310271	-2.126345
H	-1.289430	-2.650072	-0.408437
C	1.345120	-2.034506	0.028075
C	-0.430005	-0.656465	-0.754400
O	3.474141	-1.822854	1.019093
C	4.892401	-1.682579	1.012980
H	5.379973	-2.631224	0.779033
H	5.168472	-1.374786	2.018479
H	5.204518	-0.915932	0.300932
C	3.443960	-3.023311	-1.105393
H	3.412847	-3.974731	-0.571818
H	4.475901	-2.791200	-1.366649
H	2.855020	-3.101655	-2.015970
C	0.988095	-2.645386	1.370729
H	1.366051	-2.029902	2.183954
H	1.439872	-3.636011	1.439147
H	-0.092590	-2.750232	1.466418
H	-0.400679	-0.037348	-1.651710
C	-1.514481	-0.093289	0.182190
C	-2.905094	-0.374105	-0.205226
C	-3.875955	-0.648717	0.790358
C	-2.152988	-0.517830	2.494214
H	-0.284711	-0.019465	1.823872
C	-3.467809	-0.703838	2.162123
H	-1.798965	-0.540270	3.515857
H	-4.193568	-0.900721	2.939045
H	-1.328382	1.163658	0.025838
C	-5.213156	-0.873840	0.410944
C	-5.580888	-0.826769	-0.916586
H	-6.610764	-1.000620	-1.202047
C	-4.619918	-0.550464	-1.900109
H	-4.911570	-0.510023	-2.942208
C	-3.301303	-0.325448	-1.549761
H	-2.574895	-0.101768	-2.322322
H	-5.946228	-1.084745	1.180950
N	-1.225332	-0.303503	1.550077
O	-0.870597	2.413922	-0.234642
C	0.199742	2.564134	0.430914
O	0.602800	1.941048	1.400686
C	1.060424	3.752329	-0.055181
9	0.646438	4.881291	0.552189
9	2.348967	3.592909	0.248513
9	0.970146	3.959468	-1.369063

Structure **4_TS-deprot_*S*·TFA**

O	-0.830218	-2.466400	-1.227850
O	-2.511096	-1.436771	1.052168
O	-0.760259	-0.795359	2.268585
N	-0.786259	-0.524863	-0.043911
C	-2.894054	-1.460661	-0.341725
C	-1.290380	-0.904066	1.191101
C	0.525880	-2.283791	-0.787481
H	0.706975	-2.918477	0.084211
H	1.212717	-2.552118	-1.589939
C	-1.545668	-1.236555	-1.063282
C	0.616149	-0.789103	-0.425317
O	-3.658931	-0.337812	-0.612369
C	-4.902719	-0.247947	0.074491
H	-5.637753	-0.936743	-0.347834
H	-5.245895	0.774875	-0.062185
H	-4.773445	-0.445665	1.140308
C	-3.576049	-2.778001	-0.623470
H	-3.929932	-2.787072	-1.655568
H	-4.425191	-2.911562	0.046504
H	-2.871164	-3.592757	-0.474742
C	-1.670314	-0.506937	-2.385351
H	-2.037345	0.503712	-2.223197
H	-2.366264	-1.050554	-3.026492
H	-0.704086	-0.473163	-2.888685
H	0.745755	-0.217766	-1.340332
C	1.737912	-0.310735	0.502501
C	3.015175	-0.006467	-0.181565
C	3.056808	-1.421144	2.240879
H	1.025601	-1.217831	2.217036
C	4.247164	-0.427040	0.383594
C	4.243289	-1.149064	1.617512
H	2.999985	-1.934254	3.191239
H	5.173048	-1.469058	2.067116
H	1.221207	0.862253	0.834626
N	1.887156	-1.067495	1.692914
C	5.458195	-0.118900	-0.268518
C	5.459127	0.604967	-1.439417
H	6.392480	0.839309	-1.935816
C	4.244620	1.057088	-1.976156
H	4.244872	1.653004	-2.880601
C	3.044469	0.762522	-1.356783
H	6.387560	-0.457313	0.174977
H	2.122520	1.165386	-1.757229
O	0.454064	1.891310	1.072009
C	-0.232124	2.183196	0.036748
O	0.088253	2.159424	-1.136137
C	-1.673859	2.620498	0.394976
9	-2.448102	2.729187	-0.684094
9	-1.664998	3.813783	1.009417
9	-2.262916	1.753697	1.229777

Structure **4E'_*R*·TFA**

O	1.341990	-1.522871	1.079463
O	3.182420	-0.542712	-1.081526
O	1.651840	-0.685431	-2.711133
N	1.060817	-0.012871	-0.573458
C	3.214758	-0.171686	0.314955
C	1.934943	-0.436390	-1.575168
C	-0.018684	-1.688509	0.685335
H	-0.147254	-2.728325	0.389526
H	-0.694789	-1.453115	1.511064
C	1.721412	-0.207995	0.715311
C	-0.237539	-0.693899	-0.510123
O	3.612190	1.153080	0.411380
C	4.962500	1.422738	0.043740
H	5.655398	1.079725	0.814830
H	5.038538	2.502929	-0.053299
H	5.206043	0.955002	-0.912401
C	4.099962	-1.153775	1.046758
H	4.195522	-0.847149	2.089764

H	5.088574	-1.181284	0.589462
H	3.658313	-2.146176	1.000294
C	1.321111	0.792814	1.780367
H	1.532717	1.808284	1.453792
H	1.884367	0.586024	2.691572
H	0.256025	0.695844	1.999322
H	-0.379274	-1.245521	-1.444560
C	-1.367919	0.279279	-0.305249
C	-2.698366	-0.127078	-0.119630
C	-3.719107	0.867900	0.065745
C	-2.025498	2.589982	-0.208276
H	-0.111733	1.884741	-0.573462
C	-3.314271	2.261166	0.020731
H	-1.677308	3.611841	-0.270491
H	-4.051714	3.041208	0.155302
C	-5.036407	0.477474	0.269017
C	-5.398402	-0.874041	0.289684
H	-6.429919	-1.160516	0.450776
C	-4.417933	-1.845644	0.092407
H	-4.691134	-2.894793	0.093723
C	-3.092309	-1.492831	-0.111841
H	-2.358019	-2.271347	-0.281893
H	-5.790482	1.244980	0.409163
N	-1.069518	1.627741	-0.378046

Structure **4E'_*S*·TFA**

O	1.359089	-0.240405	-1.818207
O	1.729013	0.886817	1.085907
O	-0.059194	-0.024753	2.081624
N	0.951752	-1.045988	0.262591
C	2.698534	0.515768	0.089853
C	0.774382	-0.068963	1.222496
C	0.240617	-1.102792	-1.991250
H	-0.545877	-0.539143	-2.488174
H	0.511987	-1.963647	-2.605343
C	2.027098	-0.677988	-0.644891
C	-0.178863	-1.574474	-0.556815
O	3.817420	0.004333	0.735510
C	4.561155	0.935176	1.515700
H	5.151942	1.598454	0.880176
H	5.230338	0.343065	2.135478
H	3.900239	1.523004	2.155889
C	2.981092	1.723474	-0.776429
H	3.771676	1.482837	-1.489204
H	3.299208	2.560580	-0.155570
H	2.083447	2.007545	-1.320967
C	2.961182	-1.830624	-0.965277
H	3.408098	-2.221153	-0.054903
H	3.750565	-1.484765	-1.633977
H	2.404970	-2.627081	-1.462642
H	-0.126703	-2.663998	-0.524394
C	-1.550636	-1.164018	-0.133194
C	-2.094233	0.138048	-0.238516
C	-3.446480	0.368281	0.180182
C	-3.608618	-1.957380	0.854920
H	-1.920928	-3.081393	0.559302
C	-4.193221	-0.748144	0.727158
H	-4.110901	-2.818307	1.274164
H	-5.214883	-0.606291	1.052969
C	-4.002641	1.639796	0.069845
C	-3.265171	2.712441	-0.431503
H	-3.713334	3.694910	-0.510676
C	-1.941086	2.505189	-0.814819
H	-1.350371	3.334134	-1.187965
C	-1.357083	1.250846	-0.719077
H	-0.320115	1.136135	-1.008980
H	-5.029015	1.785879	0.389785
N	-2.321341	-2.162088	0.449704

Structure **6B_*S***

O	-0.239988	1.272448	-0.963269
O	1.663419	0.434660	1.196877
O	0.052390	0.186547	2.736928
N	-0.299519	-0.420279	0.529418
C	1.818148	0.202789	-0.224498
C	-2.602766	-1.087148	0.016702
C	0.437495	0.075530	1.609862
O	-3.714821	-0.958562	-0.421586
C	-1.625709	1.188887	-0.614198
H	-2.211105	0.910607	-1.494944
C	0.358728	0.014167	-0.701263
C	-1.678945	0.032904	0.439538
O	2.446248	-1.016833	-0.408466
C	3.808703	-1.082590	0.006719
H	4.458368	-0.555169	-0.694576
H	4.069560	-2.137861	0.015425
H	3.928873	-0.670944	1.010847
C	2.540741	1.388665	-0.819712
H	2.729998	1.202222	-1.878144
H	3.489902	1.544508	-0.307999
H	1.927752	2.280205	-0.712538
C	0.181162	-0.938254	-1.864693
H	0.527475	-1.933598	-1.598311
H	0.752150	-0.571010	-2.718457
H	-0.870400	-0.985442	-2.156178
O	-2.085286	-2.292053	0.179042
H	-2.021585	0.378312	1.418385
C	-2.082379	2.523782	-0.070669
H	-3.141923	2.475327	0.185951
H	-1.510136	2.781547	0.823006
H	-1.939067	3.301212	-0.821319

Structure **6C_*S***

O	-1.101257	-1.281926	-0.025751
O	1.324907	1.200023	-0.195619
O	-0.070254	2.973846	-0.141222
N	-0.728593	0.870433	0.546245
C	1.140605	-0.254568	-0.255633
C	0.142561	1.808803	0.048089
C	-2.404298	-0.682389	0.163761
H	-2.868809	-1.145886	1.043500
C	-0.142233	-0.465722	0.600508
C	-2.105475	0.778585	0.406853
O	2.202527	-0.855799	0.373667
C	3.460812	-0.754432	-0.289508
H	3.496104	-1.402473	-1.167496
H	4.206700	-1.082224	0.430195
H	3.663320	0.278719	-0.578739
C	0.959397	-0.630586	-1.711412
H	0.897266	-1.714775	-1.806283
H	1.794001	-0.252485	-2.300821
H	0.035352	-0.195171	-2.091784
C	0.122881	-0.914153	2.024799
H	0.839258	-0.249733	2.507439
H	0.525273	-1.926148	2.020400
H	-0.817243	-0.896119	2.579362

H	−2.660246	1.614911	0.008762
C	−3.252215	−0.947613	−1.064484
H	−3.360467	−2.021869	−1.219281
H	−4.244655	−0.513951	−0.930584
H	−2.785292	−0.502524	−1.944823

Structure **6_TS-*epi***

O	−1.115697	−1.266115	−0.001127
O	1.362815	1.180679	−0.174214
O	0.005823	2.986008	−0.186457
N	−0.721462	0.904420	0.503299
C	1.137296	−0.268233	−0.246399
C	0.185507	1.818575	0.022122
C	−2.412403	−0.650199	0.167112
H	−2.868170	−1.048552	1.084774
C	−0.152317	−0.439020	0.603503
C	−2.087840	0.811336	0.295997
O	2.179077	−0.904834	0.380448
C	3.440047	−0.838749	−0.282286
H	3.455020	−1.482141	−1.164277
H	4.175459	−1.193949	0.435159
H	3.674297	0.189408	−0.565300
C	0.944412	−0.629648	−1.704651
H	0.854734	−1.711218	−1.806595
H	1.786926	−0.268571	−2.293482
H	0.030464	−0.168634	−2.080596
C	0.099793	−0.839624	2.044148
H	0.825508	−0.169873	2.505348
H	0.485911	−1.857487	2.077329
H	−0.841445	−0.786317	2.594316
H	−2.736231	1.668110	0.246413
C	−3.280853	−0.999888	−1.025586
H	−3.409536	−2.081263	−1.090372
H	−4.263822	−0.538837	−0.915566
H	−2.816866	−0.634830	−1.943215

Structure **6C_*R***

O	−1.125582	−1.253084	0.008503
O	1.382488	1.168375	−0.165357
O	0.052984	2.991504	−0.203268
N	−0.717602	0.923596	0.484018
O	2.165020	−0.931213	0.385660
C	1.134815	−0.277610	−0.241715
C	0.087308	−0.805048	2.051281
H	0.817528	−0.133758	2.503247
H	0.465774	−1.825190	2.100927
H	−0.853644	−0.735743	2.599793
C	−2.076253	0.844809	0.197958
C	0.212775	1.823375	0.012886
C	−2.413464	−0.620698	0.165144
H	−2.855393	−0.953782	1.115593
C	−0.158876	−0.424954	0.603962
C	3.427349	−0.886665	−0.276509
H	4.156018	−1.255772	0.440771
H	3.679752	0.137567	−0.558131
H	3.431147	−1.529064	−1.159327
C	0.937656	−0.632529	−1.701076
H	0.835323	−1.712706	−1.805802
H	1.783982	−0.279683	−2.289432
H	0.028644	−0.160073	−2.075528
H	−2.751596	1.662683	0.392913
C	−3.306312	−1.034225	−0.987233
H	−3.446432	−2.116026	−0.983194
H	−4.282475	−0.556397	−0.889479
H	−2.854681	−0.730623	−1.932966

Structure **6_TS-coup_*Re***

O	1.014520	1.384014	−1.259283
O	2.690425	−0.562085	1.118210
O	1.325648	−0.177037	2.875364
N	0.711696	0.327174	0.708460
C	2.782229	−0.041777	−0.265685
C	1.562436	−0.144826	1.704180
C	−0.169785	1.945150	−0.656203
H	−1.046630	1.568819	−1.199757
C	1.282230	0.156098	−0.635442
C	−0.142839	1.390401	0.759027
O	3.313145	−1.021264	−1.055667
C	4.698596	−1.309015	−0.860918
H	5.323807	−0.507612	−1.258128
H	4.891947	−2.225830	−1.411291
H	4.914153	−1.465711	0.197625
C	3.563595	1.252957	−0.211490
H	3.721260	1.624352	−1.224123
H	4.522454	1.090915	0.279176
H	3.006097	2.002908	0.350342
C	0.679747	−1.008908	−1.391808
H	0.857238	−1.944591	−0.861907
H	1.138498	−1.066433	−2.377694
H	−0.395128	−0.854070	−1.499500
H	−0.131627	2.019543	1.642773
C	−2.278687	0.674359	1.277926
C	−2.274298	−0.603944	0.649138
C	−3.741560	1.518561	−0.413246
H	−3.171742	2.505834	1.295640
C	−2.975438	−0.765197	−0.572980
C	−3.699297	0.350162	−1.100886
H	−4.296661	2.387814	−0.732971
H	−4.237111	0.258856	−2.034593
H	−1.969543	0.789175	2.307774
N	−3.073899	1.640431	0.775689
C	−2.956556	−2.025058	−1.205292
C	−2.271261	−3.072731	−0.632902
H	−2.262868	−4.039463	−1.120913
C	−1.579530	−2.906900	0.583919
H	−1.048580	−3.745122	1.016778
C	−1.572110	−1.686393	1.217697
H	−3.490301	−2.154874	−2.138964
H	−1.040417	−1.543818	2.152169
C	−0.108792	3.453837	−0.733944
H	−0.041328	3.764905	−1.776293
H	−1.011826	3.886589	−0.299884
H	0.761745	3.824895	−0.190835

Structure **6_TS-coup_*Si***

O	−1.080792	1.326274	−1.152611
O	−2.819269	−0.829255	0.956042
O	−1.197508	−1.123922	2.489488
N	−1.156426	0.621672	0.978576
C	−2.905349	−0.151828	−0.360000
C	−1.685020	−0.500664	1.583508
C	0.136663	1.763088	−0.532793
H	0.052732	2.829191	−0.278579
C	−2.004387	1.090318	−0.127204
C	0.167286	1.007552	0.800040
O	−4.195072	0.253713	−0.548487

C	−5.150693	−0.779342	−0.794312
H	−5.020188	−1.203279	−1.791385
H	−6.125519	−0.304104	−0.727384
H	−5.072962	−1.562797	−0.038249
C	−2.363125	−1.105705	−1.402120
H	−2.531861	−0.688338	−2.395218
H	−2.850779	−2.075949	−1.317658
H	−1.289230	−1.231442	−1.266404
C	−2.775931	2.339176	0.254417
H	−3.449227	2.130801	1.085421
H	−3.363817	2.669666	−0.600678
H	−2.079313	3.125638	0.546384
H	0.690644	1.459778	1.636929
C	1.395881	−0.745286	1.084946
C	2.677857	−0.303219	0.610339
C	1.288776	−2.340793	−0.702579
H	0.056235	−2.272203	0.945030
C	3.188869	−0.853990	−0.591397
C	2.438440	−1.870595	−1.256084
H	0.706547	−3.148112	−1.122345
H	2.798812	−2.296587	−2.182623
H	1.134677	−0.651375	2.131513
N	0.830524	−1.817521	0.469619
C	4.419307	−0.380884	−1.091575
C	5.108638	0.596425	−0.412813
H	6.050405	0.962136	−0.802289
C	4.606533	1.123836	0.794640
H	5.171211	1.880521	1.324097
C	3.408158	0.679767	1.303893
H	4.806827	−0.795337	−2.014366
H	3.025295	1.076765	2.237060
C	1.265843	1.573468	−1.519210
H	1.084745	2.217913	−2.379275
H	2.224182	1.847548	−1.075259
H	1.296547	0.538091	−1.857720

Structure **6D_*R***

O	−1.176281	1.102861	1.580225
O	−2.753223	0.108805	−0.916666
O	−1.501594	1.171564	−2.441547
N	−0.567746	0.395450	−0.479030
C	−2.563201	−0.518976	0.373696
C	−1.602343	0.604988	−1.389592
C	0.023299	1.830382	1.300285
H	0.840017	1.412834	1.895170
C	−1.132445	−0.071464	0.791626
C	0.261159	1.571099	−0.212634
O	−2.510909	−1.888958	0.182154
C	−3.726806	−2.506050	−0.234154
H	−4.436489	−2.572661	0.592830
H	−3.458557	−3.508139	−0.559347
H	−4.173799	−1.961514	−1.068286
C	−3.667387	−0.049558	1.293180
H	−3.585669	−0.567768	2.250157
H	−4.639523	−0.262933	0.850132
H	−3.576856	1.021642	1.455471
C	−0.335026	−1.166739	1.474960
H	−0.178848	−2.002624	0.797208
H	−0.889891	−1.515142	2.347272
H	0.630850	−0.786630	1.808005
H	−0.127629	2.412621	−0.792264
C	1.735823	1.370101	−0.682240
C	2.419189	0.202035	−0.032381
C	2.335460	−1.071973	−0.643772
C	1.579234	0.004343	−2.697205
H	1.464582	2.008014	−2.694274
C	1.828892	−1.164446	−1.967250
H	1.353648	−0.021320	−3.755278
H	1.732204	−2.120861	−2.461954
H	2.282863	2.294932	−0.489254
C	2.844429	−2.202109	0.027574
C	3.449261	−2.058877	1.260283
H	3.840914	−2.925106	1.777484
C	3.581377	−0.788251	1.828720
H	4.085809	−0.673732	2.779894
C	3.070707	0.337008	1.183881
H	3.183782	1.315716	1.636529
H	2.767671	−3.174259	−0.444558
N	1.654265	1.186380	−2.129714
C	−0.183140	3.287529	1.643642
H	−0.391040	3.393623	2.708322
H	0.716679	3.859741	1.409131
H	−1.021189	3.692325	1.073757

Structure **6D_*S***

O	1.298489	−1.714176	0.709522
O	2.839787	0.349426	−1.105708
O	1.057443	1.348639	−2.007213
N	0.981095	0.484025	0.139085
C	3.215848	−0.241378	0.168972
C	1.576996	0.777808	−1.078798
C	0.051059	−1.643266	0.002281
H	−0.651967	−2.297772	0.518318
C	1.849517	−0.411723	0.898619
C	−0.343694	−0.157346	0.182087
O	3.933392	0.700352	0.880723
C	5.223913	1.025369	0.366711
H	5.937405	0.223386	0.564826
H	5.537104	1.924405	0.891351
H	5.175557	1.228632	−0.704885
C	3.954857	−1.527977	−0.115030
H	4.295337	−1.962567	0.826352
H	4.815447	−1.333729	−0.754189
H	3.292085	−2.231782	−0.613034
C	1.898380	−0.109828	2.381693
H	2.250435	0.903302	2.556424
H	2.575964	−0.814038	2.866848
H	0.903932	−0.240357	2.809226
H	−0.718047	−0.011461	1.198420
C	−1.411334	0.415757	−0.781759
C	−2.775926	−0.069414	−0.347165
C	−3.603234	0.778086	0.432847
C	−2.115308	2.648757	−0.047366
H	−0.529780	2.257134	−1.259858
C	−3.229384	2.132632	0.624825
H	−1.883504	3.705888	−0.038616
H	−3.840965	2.798813	1.216852
H	−1.195105	0.110230	−1.806944
C	−4.825581	0.289110	0.939488
C	−5.224472	−1.002302	0.664257
H	−6.162808	−1.377423	1.051319
C	−4.417892	−1.820604	−0.130200
H	−4.736414	−2.828547	−0.364195
C	−3.202036	−1.356029	−0.630270
H	−2.601161	−2.011702	−1.246485
H	−5.446820	0.948762	1.533245

N	−1.326322	1.873340	−0.749861
C	0.270339	−2.103204	−1.432410
H	0.959006	−1.448894	−1.972159
H	0.712225	−3.099727	−1.405822
H	−0.656512	−2.164705	−2.002849

Structure **8B_*S***

O	0.126592	−1.373834	1.121327
O	−1.608149	1.213133	−0.470454
O	0.120974	2.581516	−0.877514
N	0.436699	0.343870	−0.368092
C	−1.800138	−0.070462	0.199366
C	2.714785	−0.478891	−0.401405
C	−0.297695	1.497015	−0.590127
O	3.725711	−0.978193	0.016335
C	1.204079	−0.586543	1.611753
H	0.872626	0.074168	2.418056
H	1.986648	−1.249086	1.979654
C	−0.432815	−0.788843	−0.039145
C	1.668074	0.265396	0.417637
C	2.272528	1.599241	0.845439
H	1.542894	2.170485	1.419205
H	2.582145	2.187817	−0.016344
H	3.138793	1.390266	1.474289
O	−2.786627	−0.763485	−0.459073
C	−4.107662	−0.237676	−0.345577
H	−4.525878	−0.434130	0.643531
H	−4.700633	−0.754252	−1.096072
H	−4.116503	0.834769	−0.550034
C	−2.091705	0.207534	1.659573
H	−2.289947	−0.729790	2.179143
H	−2.951906	0.870169	1.748067
H	−1.239952	0.699267	2.130025
C	−0.485265	−1.837079	−1.132134
H	−0.884687	−1.406834	−2.049991
H	−1.128926	−2.658009	−0.819114
H	0.522065	−2.212150	−1.310922
O	2.455406	−0.505129	−1.696261

Structure **8C_*S***

O	0.506877	−1.734793	0.561660
O	−0.903455	1.375351	−0.098104
O	1.007173	2.553284	−0.304837
N	0.961987	0.243195	−0.441719
C	−1.241560	−0.006218	0.250030
C	0.433342	1.498639	−0.277048
C	1.934728	−1.590826	0.563522
H	2.274401	−1.584173	1.605224
H	2.397340	−2.442986	0.053805
C	−0.044490	−0.803871	−0.343306
C	2.219638	−0.275787	−0.124052
C	3.378271	0.598922	0.183143
H	3.248904	1.115284	1.144750
H	3.511347	1.360763	−0.584914
H	4.286843	−0.001713	0.247050
O	−2.389550	−0.358083	−0.418420
C	−3.583885	0.291953	0.009748
H	−3.917082	−0.092708	0.975798
H	−4.334463	0.069033	−0.744454
H	−3.436640	1.372248	0.068502
C	−1.327887	−0.090388	1.760065
H	−1.660629	−1.085687	2.054920
H	−2.021654	0.660511	2.136723

H	−0.344747	0.090584	2.195157
C	−0.325181	−1.455722	−1.683215
H	−0.728155	−0.727785	−2.387127
H	−1.046312	−2.261879	−1.555285
H	0.610721	−1.860147	−2.073825

Structure **8_TS-*epi***

O	0.508142	−1.695387	0.632895
O	−0.939631	1.374959	−0.089383
O	0.961135	2.587406	−0.156094
N	0.964529	0.284872	−0.368291
C	−1.266044	−0.016036	0.232203
C	0.402236	1.524979	−0.192800
C	1.937296	−1.581359	0.596560
H	2.311294	−1.722577	1.614337
H	2.364165	−2.371320	−0.037541
C	−0.025029	−0.784837	−0.304684
C	2.201182	−0.202315	0.061200
C	3.475897	0.541232	−0.041267
H	3.504337	1.410096	0.625898
H	3.637563	0.911347	−1.058791
H	4.307105	−0.114006	0.222627
O	−2.371122	−0.386708	−0.495990
C	−3.599301	0.234882	−0.125325
H	−3.973791	−0.164926	0.819217
H	−4.305075	0.003004	−0.919068
H	−3.478170	1.317456	−0.050877
C	−1.427727	−0.111176	1.735434
H	−1.751119	−1.115812	2.008604
H	−2.158161	0.620799	2.078480
H	−0.472849	0.090712	2.220864
C	−0.229113	−1.449134	−1.652277
H	−0.611564	−0.732299	−2.378410
H	−0.940348	−2.268168	−1.553644
H	0.730325	−1.837980	−1.999334

Structure **8C_*R***

O	0.518008	−1.691609	0.629563
O	−0.968385	1.366257	−0.114442
O	0.904642	2.620296	−0.111169
N	0.973061	0.318765	−0.316085
O	−2.359016	−0.430979	−0.511688
C	−1.268606	−0.029823	0.220228
C	−0.169743	−1.399974	−1.667919
H	−0.543218	−0.667234	−2.383056
H	−0.875364	−2.227572	−1.607041
H	0.800151	−1.769422	−2.005909
C	2.183742	−0.110111	0.256643
C	0.370881	1.547253	−0.170608
C	1.943163	−1.552644	0.610636
H	2.324063	−1.818911	1.597688
H	2.384855	−2.238627	−0.128524
C	−0.004396	−0.768806	−0.298947
C	−3.604789	0.161267	−0.150822
H	−4.300607	−0.094412	−0.945982
H	−3.511805	1.247071	−0.083611
H	−3.972732	−0.241540	0.794965
C	−1.436266	−0.113198	1.723508
H	−1.745373	−1.120242	2.004101
H	−2.179250	0.610568	2.056902
H	−0.486650	0.107623	2.211858
H	3.772149	0.156091	−1.155265
C	3.491773	0.469026	−0.139496

H	4.275168	0.136763	0.543017
H	3.450636	1.558827	−0.123987

Structure 8**_TS–coup_*Re***

O	−0.998924	−0.906261	−1.779082
O	−2.676005	0.174691	1.095425
O	−1.332754	−0.738694	2.656374
N	−0.705115	−0.580391	0.434736
C	−2.756964	0.137388	−0.380427
C	−1.556808	−0.420365	1.525114
C	0.173649	−1.626994	−1.387097
H	0.117239	−2.633056	−1.800408
H	1.069852	−1.127730	−1.773249
C	−1.255133	0.045800	−0.778719
C	0.129495	−1.634688	0.136326
O	−3.263566	1.331664	−0.809896
C	−4.645665	1.566169	−0.536039
H	−5.280936	0.953016	−1.177451
H	−4.817713	2.616816	−0.753782
H	−4.868899	1.369222	0.514164
C	−3.559275	−1.086727	−0.765468
H	−3.713955	−1.093067	−1.844535
H	−4.520021	−1.080277	−0.252258
H	−3.021151	−1.993278	−0.487863
C	−0.630960	1.386408	−1.102018
H	−0.793433	2.092621	−0.288072
H	−1.089712	1.778437	−2.008365
H	0.440424	1.261789	−1.262778
C	2.242907	−1.088824	0.801498
C	2.251813	0.331366	0.661471
C	3.715441	−1.288424	−1.076814
H	3.163480	−2.805303	0.184974
C	2.957377	0.908450	−0.424623
C	3.674995	0.047026	−1.313651
H	4.269151	−1.991375	−1.681100
H	4.212358	0.458848	−2.156872
H	1.972145	−1.547470	1.742908
N	3.049647	−1.816499	−0.005666
C	2.951881	2.310353	−0.571275
C	2.276903	3.095798	0.335780
H	2.279409	4.172617	0.220806
C	1.583567	2.516954	1.417210
H	1.062092	3.152745	2.121348
C	1.562076	1.150649	1.577387
H	3.489020	2.755127	−1.400223
H	1.028608	0.693022	2.403448
C	0.022153	−2.916210	0.904748
H	0.820197	−3.601540	0.618895
H	0.045506	−2.749984	1.980567
H	−0.930112	−3.398908	0.653373

Structure **8_TS–coup_*Si***

O	1.034542	−1.433843	0.974064
O	2.720801	0.934048	−0.907574
O	1.132852	2.525340	−0.815308
N	1.213745	0.791550	0.704016
C	2.851219	−0.458225	−0.421265
C	1.648255	1.528578	−0.378551
C	−0.066890	−0.835030	1.656739
H	−0.968092	−1.408943	1.454867
H	0.099129	−0.825235	2.741389
C	2.015795	−0.424286	0.891695
C	−0.104516	0.590775	1.136424
O	4.162205	−0.680113	−0.105430
C	5.076672	−0.750120	−1.200792
H	4.959862	−1.686504	−1.748785
H	6.069267	−0.709900	−0.760522
H	4.939880	0.099816	−1.871790
C	2.288890	−1.368524	−1.490864
H	2.438714	−2.407415	−1.195400
H	2.782327	−1.177408	−2.442650
H	1.220937	−1.198958	−1.616616
C	2.859557	−0.346228	2.149470
H	3.572034	0.473934	2.073453
H	3.406938	−1.279354	2.274551
H	2.217076	−0.178854	3.014134
C	−1.266410	0.571719	−0.753093
C	−2.305461	−0.395453	−0.556240
C	−3.608754	0.061357	−0.233905
C	−2.872076	2.334589	−0.577997
H	−0.899339	2.531572	−1.143202
C	−3.859530	1.466719	−0.223222
H	−3.009720	3.403799	−0.644485
H	−4.843247	1.844774	0.018947
H	−0.368922	0.289073	−1.288452
C	−4.625509	−0.887647	0.005000
C	−4.353461	−2.230946	−0.100893
H	−5.138233	−2.955502	0.076850
C	−3.063280	−2.679458	−0.450809
H	−2.871177	−3.740624	−0.543623
C	−2.049858	−1.776242	−0.671626
H	−1.051903	−2.116497	−0.927203
H	−5.620170	−0.540726	0.257870
N	−1.633624	1.872576	−0.890354
C	−0.809992	1.662753	1.899489
H	−0.699798	2.633700	1.418234
H	−1.868438	1.424555	2.017414
H	−0.359087	1.726368	2.896007

Structure **8D_*R***

O	0.986255	−0.851661	1.734995
O	2.706028	0.073271	−1.114710
O	1.289727	−0.805814	−2.627157
N	0.647430	−0.411892	−0.451526
C	2.774219	0.152650	0.347489
C	1.521992	−0.433400	−1.508125
C	−0.303000	−1.360209	1.418481
H	−0.409675	−2.335658	1.888376
H	−1.080567	−0.689079	1.797375
C	1.269396	0.125438	0.758102
C	−0.323168	−1.451641	−0.133520
O	3.304727	1.371857	0.690525
C	4.682727	1.567196	0.377789
H	5.323827	1.001748	1.056698
H	4.870562	2.630104	0.506289
H	4.888629	1.283462	−0.656131
C	3.563703	−1.043287	0.836268
H	3.702348	−0.974410	1.915536
H	4.532505	−1.076127	0.339146
H	3.026677	−1.963634	0.612102
C	0.721498	1.484504	1.156066
H	0.892981	2.207862	0.359076
H	1.224353	1.826600	2.059439
H	−0.352005	1.415417	1.341944
C	−1.731595	−1.107361	−0.760767
C	−2.114204	0.337465	−0.612748

C	−3.610002	−1.603552	0.743005
H	−2.719865	−2.964297	−0.429087
C	−3.033194	0.735960	0.389483
C	−3.745711	−0.259662	1.103512
H	−4.240513	−2.373441	1.168264
H	−4.459321	0.005827	1.870979
H	−1.665597	−1.363247	−1.821869
N	−2.730015	−1.986894	−0.158161
C	−3.286710	2.109936	0.600129
C	−2.668885	3.053643	−0.192335
H	−2.862561	4.106817	−0.035966
C	−1.808771	2.646048	−1.218851
H	−1.347448	3.387623	−1.858976
C	−1.531512	1.296794	−1.425542
H	−3.983432	2.402486	1.376471
H	−0.857737	0.994634	−2.218848
C	0.131176	−2.832773	−0.603896
H	−0.581579	−3.599754	−0.296971
H	0.247841	−2.864322	−1.686480
H	1.087025	−3.074822	−0.136027

Structure **8D_*S***

O	1.355222	−1.559351	1.038427
O	2.836882	0.821262	−1.024377
O	1.024631	2.036487	−1.487281
N	1.084802	0.598001	0.328798
C	3.148496	−0.382005	−0.251930
C	1.581805	1.217695	−0.792358
C	0.071762	−1.458466	0.434100
H	0.113574	−1.788524	−0.607851
H	−0.616866	−2.091048	0.992399
C	2.065486	−0.346024	0.878971
C	−0.276127	0.043312	0.525620
O	4.388154	−0.222487	0.314968
C	5.500531	−0.233551	−0.578596
H	5.714402	−1.245324	−0.927747
H	6.346652	0.135949	−0.004982
H	5.321292	0.427341	−1.428939
C	3.034492	−1.572237	−1.180960
H	3.303561	−2.481125	−0.643036
H	3.695354	−1.439860	−2.036783
H	2.017428	−1.671585	−1.557362
C	2.638006	0.079126	2.216852
H	3.195046	1.008320	2.107421
H	3.317466	−0.691999	2.578402
H	1.833755	0.220047	2.935145
C	−1.271586	0.490916	−0.593900
C	−2.584285	−0.253234	−0.504357
C	−3.710017	0.370527	0.091010
C	−2.529933	2.501699	0.035746
H	−0.673870	2.487842	−0.810938
C	−3.646786	1.748889	0.419666
H	−2.510285	3.579822	0.131000
H	−4.487850	2.250683	0.877081
H	−0.807659	0.302917	−1.565286
C	−4.905056	−0.358335	0.264040
C	−4.990561	−1.663900	−0.173315
H	−5.907767	−2.223271	−0.043474
C	−3.895172	−2.253509	−0.808916
H	−3.969544	−3.267049	−1.182166
C	−2.702688	−1.550820	−0.975786
H	−1.875552	−2.026213	−1.487831
H	−5.754001	0.130464	0.726748
N	−1.473406	1.933972	−0.491782
C	−0.796185	0.394276	1.918123
H	−0.752222	1.468913	2.096130
H	−1.822124	0.043969	2.042142
H	−0.192753	−0.107528	2.672861

14. Kinetic Isotopic Experiments

14.1. Characterization of compound 5-D

(3*R*,7*R*,7a*S*)-3-(isoquinolin-1-yl-d6)-7-methoxy-7,7a-dimethyltetrahydro-5*H*-oxazolo[4,3-b] oxazol-5-one

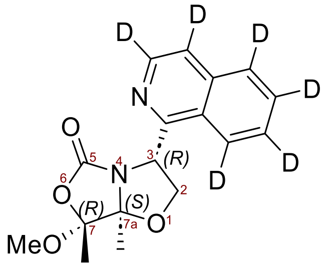

5D

Following the general experimental procedure for the Minisci reaction using D conditions, the activated ester **4** was treated with isoquinoline-d7 affording product **5-D** as a colorless oil (26.8 mg, 0.085 mmol, 85% yield). $[\alpha]^{20}_{D}$ -93.2 (c 1.0, $CHCl_3$). HRMS (ESI) m/z: $[M + H]^+$ Calcd for $C_{17}H_{13}D_6N_2O_4$: 321.1721; Found 321.1714. ^1H NMR ($CDCl_3$, 400 MHz): 6.06 (dd, *J* = 8.0, 4.8 Hz, 1H, C$\underline{H}^{\alpha}$), 5.44 (dd, *J* = 8.2, 4.8 Hz, 1H, 1C$\underline{H}_2^{\beta}$), 4.32 (t, *J* = 8.0 Hz, 1H, 1C$\underline{H}_2^{\beta}$), 3.40 (s, 3H, OC$\underline{H}_3$), 1.64 (s, 3H, C$\underline{H}_3^{7}$), 1.21 (s, 3H, C$\underline{H}_3^{7a}$). $^{13}C\{^1H\}$ NMR ($CDCl_3$, 100 MHz): δ 162.0 (CO), 155.4 (C^{*Ar}), 140.4 (t, C^{Ar}), 136.6 (C^{*Ar}), 129.9 (t, C^{Ar}), 127.8 (C^{Ar}), 127.7 (t, C^{Ar}), 126.8 (t, C^{Ar}), 125.2 (t, C^{Ar}), 121.4 (t, C^{Ar}), 107.4 (C^7), 101.4 (C^{7a}), 66.1 (C^{β}), 60.8 (C^{α}), 51.1 (O$\underline{C}H_3$), 17.6 ($\underline{C}H_3^{7a}$), 15.7 ($\underline{C}H_3^{7}$).

^1H NMR in $CDCl_3$ (400 MHz) compound 5-D

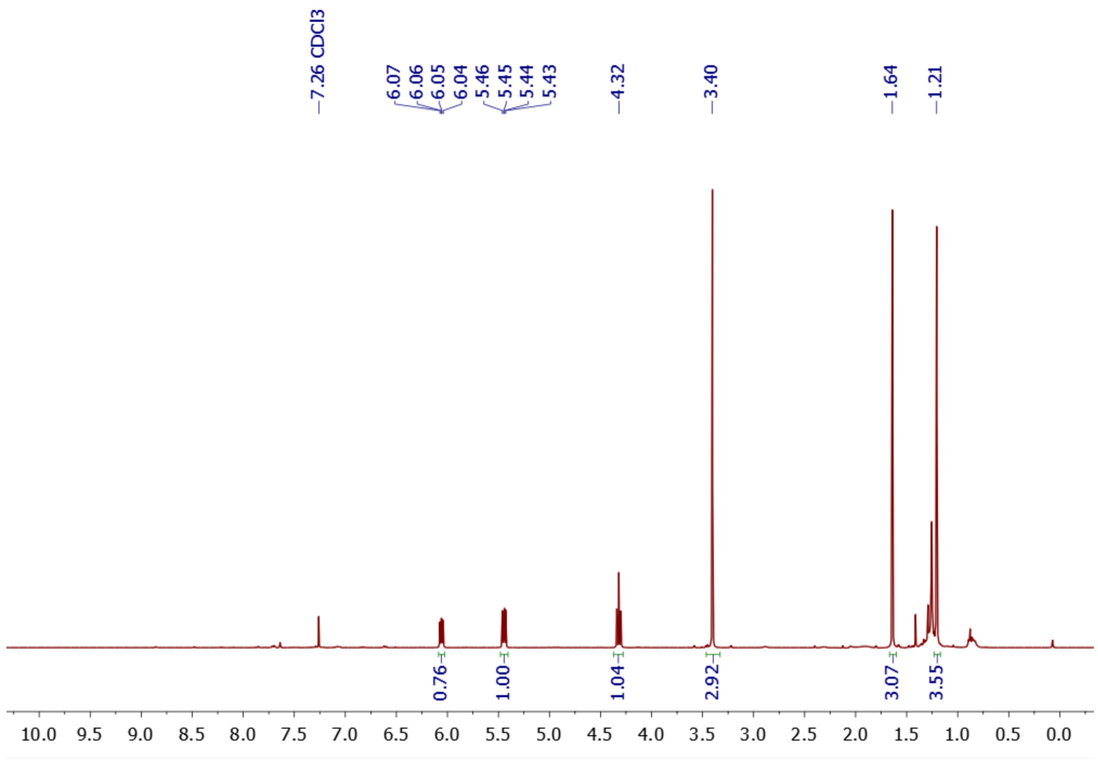

^{13}C {^1H} NMR in $CDCl_3$ (100 MHz) compound 5-D

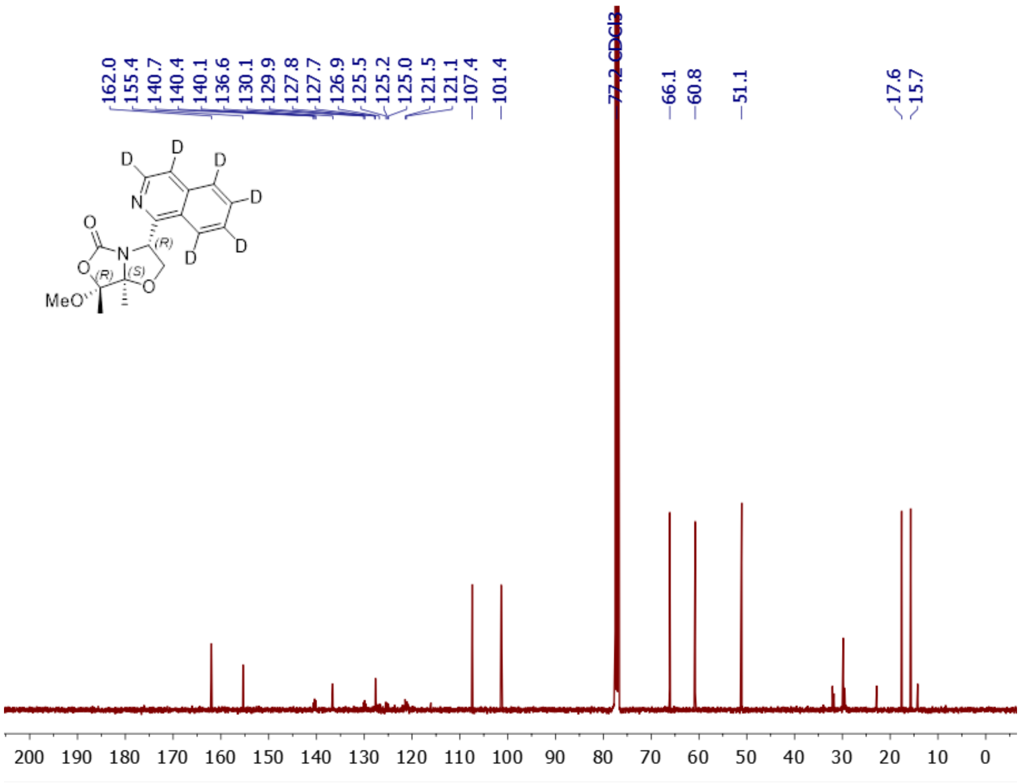

COSY in $CDCl_3$ (400 MHz) compound 5-D

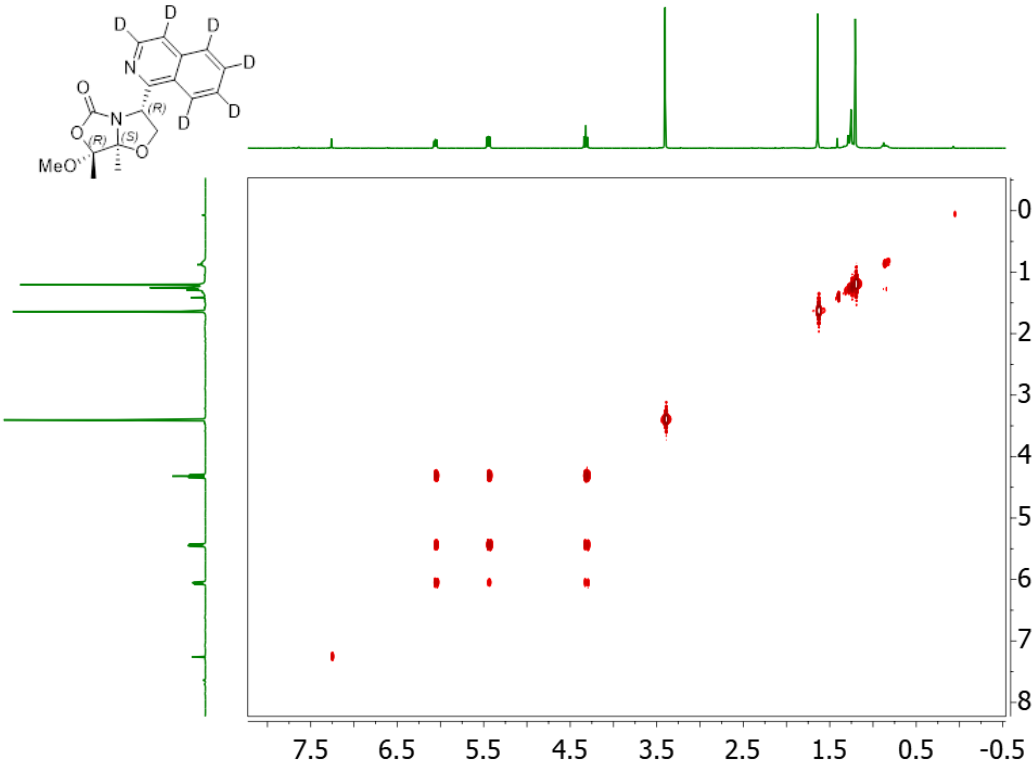

HSQC in $CDCl_3$ (400 MHz) compound 5-D

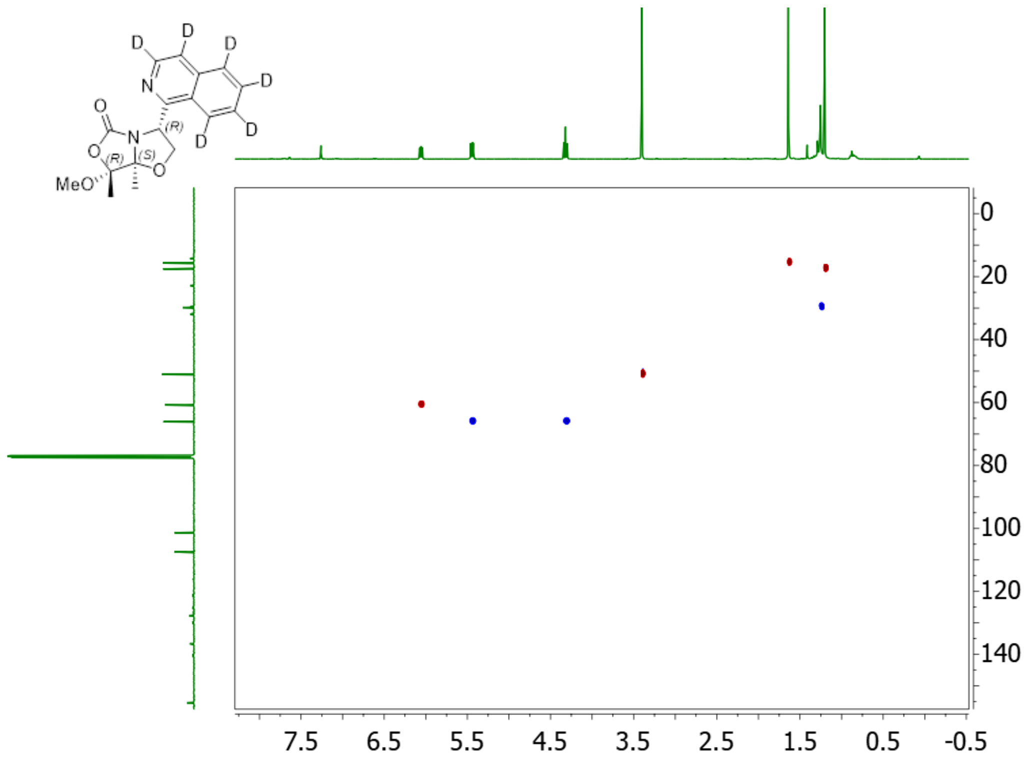

NOESY in $CDCl_3$ (400 MHz) compound 5-D

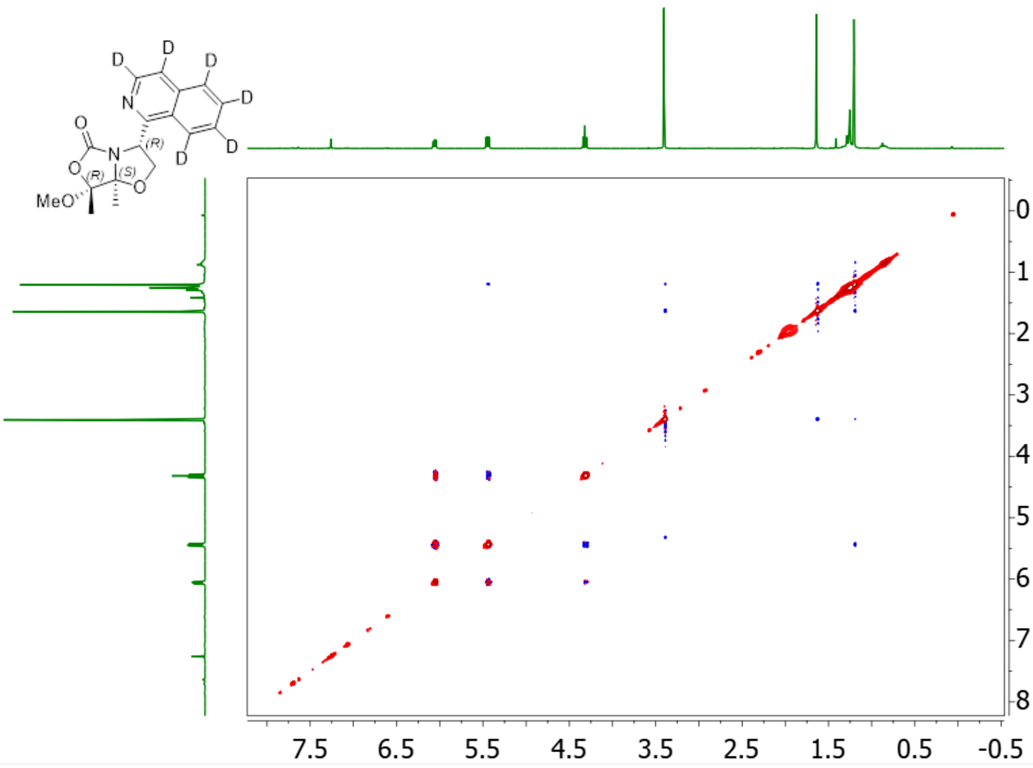

14.2. Competitive photocatalytic Minisci reaction to investigate the Kinetic Isotopic Effect (KIE)

A dried vial equipped with a Teflon septum was charged with 4-CzIPN (1 mg, 0.01 mmol, 0.01 equiv), isoquinoline (3 mg, 0.025 mmol, 0.5 equiv), isoquinoline-d7 (3.1 mg, 0.025 mmol, 0.5 equiv) and TFA (5 µL, 0.06 mmol, 1.2 equiv), and vacuum was created. Then DMF-d7 (0.5 mL) were added to the vial. The resulting mixture was transferred to an NMR tube equipped with a Teflon septum under inert atmosphere. Finally, the freshly synthesised activated ester **4** (19 mg, 0.05 mmol, 1 equiv) was added into the NMR tube. The reaction crude was analysed by ^{1}H NMR using a 400 MHz NMR before the reaction started. Then, the tube was irradiated with a blue LED (30 W, λ = 450 nm) while remaining in constant agitation. The reaction was monitored by ^{1}H NMR at one-minute intervals for ten minutes and then at longer intervals (at 15, 20, 30 and 45 minutes) until the reaction was completed.

Before to perform the kinetic isotopic experiments, we assigned the two Hβ protons by NOESY experiments in compounds **5** and **5-D**. See section 12 of this Supp. Inf. (pages S93 and S94)

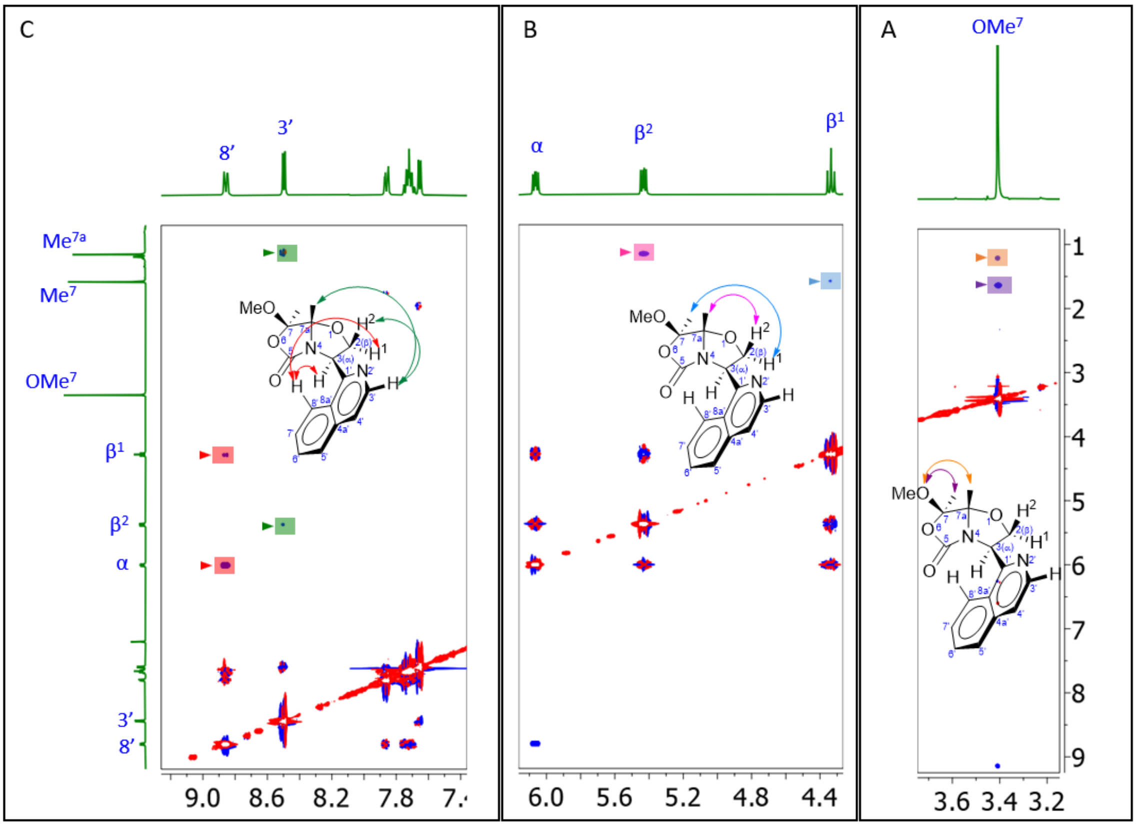

Figure S8. 2D-NOESY experiments were recorded on a 400 MHz spectrometer with TMS as the internal standard at 298 K. The experiments were conducted using phase-sensitive ge-2D-NOESY spectra. The number of scans used was 16, and the mixing time was 800 ms. NOTE: H^2 and H^1 stand $H^{\beta2}$ and $H^{\beta1}$ or Hβ and Hβ', respectively, in all figures. As an example, this figure represents the zoom of important NOESY contacts in compound **5**.

The kinetic for the KIE was measured by monitoring the integral variations in the ^{1}H NMR signals corresponding to the Hβ from compounds **4** (4.3 ppm), **5** (5.3 ppm) and **5-D** (5.3 ppm) using MestreNova software.

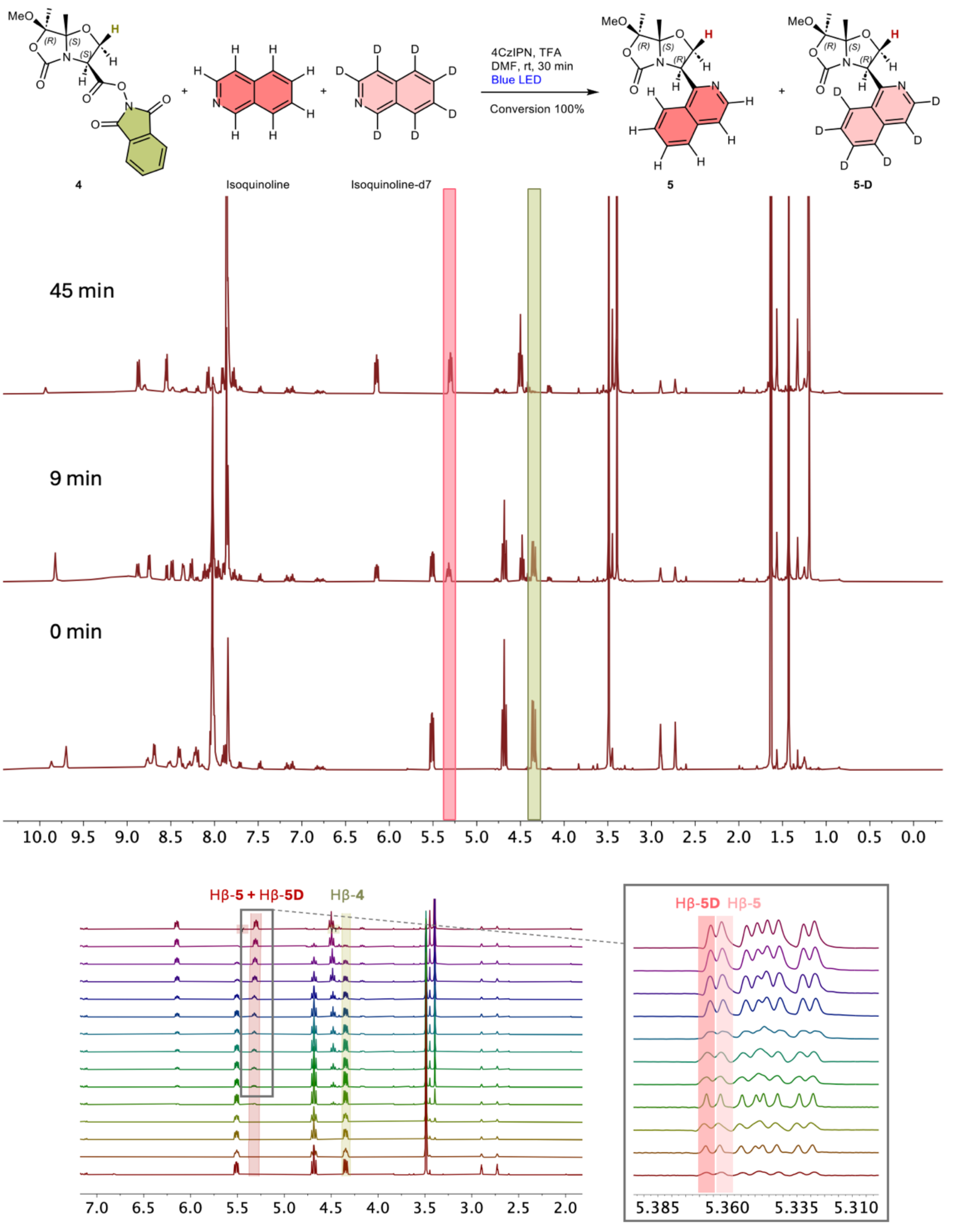

t (min)	Signal loss Hβ-4	Disappearence Hβ-4	Signal conversion Hβ-5D + Hβ-5	Conversion Hβ-5D + Hβ-5	% 5D	% 5	Conversion Hβ-5D	Conversion Hβ-5
0,00	5127,93	1,00	-40,5983	-0,01	n.d	n.d	n.d	n.d
1,00	5254,55	1,02	-30,2248	-0,01	n.d	n.d	n.d	n.d
2,00	5164,98	1,01	85,9512	0,02	n.d	n.d	n.d	n.d
3,00	5064,45	0,99	244	0,05	n.d	n.d	n.d	n.d
4,00	4957,72	0,97	481	0,09	0,47	0,53	0,04	0,05
5,00	4727,90	0,92	783	0,15	0,48	0,52	0,07	0,08
6,00	4362,16	0,85	1.140	0,22	0,48	0,52	0,11	0,12
7,00	4019,85	0,78	1.345	0,26	0,49	0,51	0,13	0,13
8,00	3679,35	0,72	1.645	0,32	0,48	0,52	0,15	0,17
9,00	3437,81	0,67	1.872	0,37	0,48	0,52	0,18	0,19
10,00	3368,33	0,66	1.958	0,38	0,49	0,51	0,19	0,19
15,00	2124,59	0,41	2.959	0,58	0,48	0,52	0,28	0,30
20,00	1611,55	0,31	3.382	0,66	0,48	0,52	0,32	0,34
30,00	830,54	0,16	4.053	0,79	0,48	0,52	0,38	0,41
45,00	333,77	0,07	4.411	0,86	0,48	0,52	0,41	0,45

Figure S9. Monitoring the integral variations in the ^{1}H NMR signals corresponding to the Hβ (or Hβ2) from compounds **4** (4.3 ppm), **5** (5.3 ppm) and **5-D** (5.3 ppm) using MestreNova software.

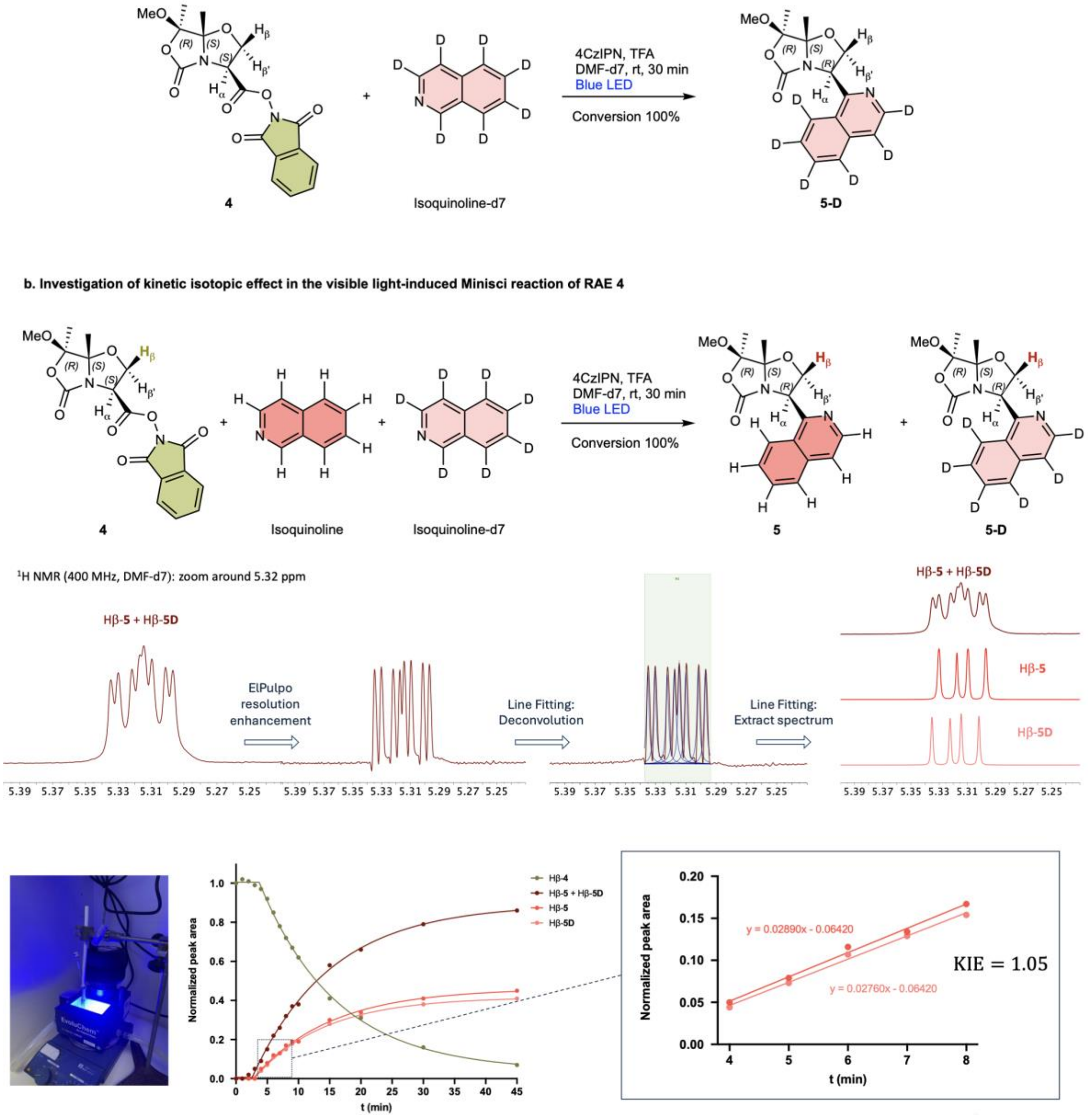

Figure S10. This figure corresponds to Figure 2 of the manuscript and provides a detailed explanation of the simplified captions presented therein: **Kinetic study of the photoredox Minisci reaction.** **(a)** Reaction of RAE **4** with fully deuterated isoquinoline to give compound **5-D**. **(b)** Kinetic isotope effect (KIE) determined from an intermolecular competition reaction of RAE **4** with equimolecular amounts of isoquinoline and isoquinoline-d7 to give products **5** and **5-D**, respectively. In the ^{1}H NMR spectra of the products, all signals match in chemical shift except for the doublet of doublets corresponding to the Hβ protons marked in bold. These peaks could be integrated after deconvolution of the spectra with MestReNova software. The data points in the graphs represent the normalized peak area of the corresponding signals associated with the Hβ protons of compounds **4** (decay, in green), **5** (formation, in red), and **5-D** (formation, in light red) during the competition reaction described above, which was carried out in a 5 mm diameter NMR tube using deuterated DMF as solvent and monitored at various reaction times. To calculate the kinetic isotope effect (KIE = 1.05) from this intermolecular competition reaction, five data points corresponding to 4 to 8 minutes of reaction time, during which the conversion ranged from 9% to 30%. Lower conversion values were excluded due to signal integration errors. Note: Although the reaction conditions employed in the NMR tube were identical to those used in conventional

reaction vials, the photoredox Minisci reaction proceeds significantly faster (86% conversion in 45 min), likely due to more efficient irradiation resulting from the smaller diameter of the reaction vessel.

15. References

S1. Anderson, Z. J.; Fox, D. J. Total synthesis of the azolemycins. *Org. Biomol. Chem.* **2016**, 14, 1450-1454.

S2. Ayurini, M.; Chandler, P. G.; O'Leary, P. D.; Wang, R.; Rudd, D.; Milewska, K. D.; Malins, L. R.; Buckle, A. M.; Hooper, J. F. Polymer End Group Control through a Decarboxylative Cobalt-Mediated Radical Polymerization: New Avenues for Synthesizing Peptide, Protein, and Nanomaterial Conjugates. *JACS Au* **2022**, *2*, 169–177.

S3. (a) Aydillo, C. Jiménez-Osés, G.; Busto J. H.; Peregrina, J. M.; Zurbano, M. M.; Avenoza, A. Theoretical Evidence for Pyrami-dalized Bicyclic Serine Enolates in Highly Diastereoselective Alkylations. *Chem. Eur. J.* **2007**, *13*, 4840–4848. (b) Jiménez-Osés, G.; Aydillo, C.; Busto, J. H.; Zurbano, M. M.; Peregrina, J. M.; Avenoza, A. Role of the Countercation in Diastereoselective Alkylations of Pyramidalized Bicyclic Serine Enolates. An Easy Approach to α-Benzylserine. *J. Org. Chem.* **2007**, *72*, 5399-5402. (c) Oroz, P.; Navo, C. D.; Avenoza, A.; Busto, J. H.; Corzana, F.; Jiménez-Osés, G.; Peregrina, J. M. Towards Enantiomerically Pure Unnatural α-Amino Acids via Photoredox Catalytic 1,4-Additions to a Chiral Dehydroalanine. *J. Org. Chem.* **2022**, *87*, 14308–14318.

S4. (a) Aydillo, C.; Avenoza, A.; Busto, J. H.; Jiménez-Osés, G.; Peregrina, J. M.; Zurbano, M. M. α-Alkylation versus retro-O-Michael/γ-alkylation of bicyclic *N,O*-acetals: an entry to α-methylthreonine, *Tetrahedron: Asymmetry* **2008**, *19*, 2829-2834. (b) Jiménez-Osés, G.; Aydillo, C.; Busto, J. H.; Zurbano, M. M.; Peregrina, J. M.; Avenoza, A. Influence of Amino Acid Stereocenters on the Formation of Bicyclic *N,O*-Acetals. *J. Org. Chem.* **2014**, *79*, 2556-2563.

S5. Blessing, R. H. An empirical correction for absorption anisotropy. *Acta Crystallogr.* **1995**, *A51*, 33-38.

S6. Farrugia, L. J. WinGX and ORTEP for Windows: an update. *J. Appl. Crystallogr*. **2012**, *45*, 849-854.

S7. Sheldrick, G. Crystal structure refinement with SHELXL. *Acta Crystallogr., Sect. C* **2015**, *71*, 3-8.

S8. Cheng, W.-M.; Shang, R.; Fu, Y. Photoredox/Brønsted Acid Co-Catalysis Enabling Decarboxylative Coupling of Amino Acid and Peptide Redox-Active Esters with N-Heteroarenes. *ACS Catal.* **2017**, *7*, 907−911.

S9. Gaussian 16, Revision C.01, Frisch, M. J.; Trucks, G. W.; Schlegel, H. B.; Scuseria, G. E.; Robb, M. A.; Cheeseman, J. R.; Scalmani, G.; Barone, V.; Petersson, G. A.; Nakatsuji, H.; Li, X.; Caricato, M.; Marenich, A. V.; Bloino, J.; Janesko, B. G.; Gomperts, R.; Mennucci, B.; Hratchian, H. P.; Ortiz, J. V.; Izmaylov, A. F.; Sonnenberg, J. L.; Williams-Young, D.; Ding, F.; Lipparini, F.; Egidi, F.; Goings, J.; Peng, B.; Petrone, A.; Henderson, T.; Ranasinghe, D.; Zakrzewski, V. G.; Gao, J.; Rega, N.; Zheng, G.; Liang, W.; Hada, M.; Ehara, M.; Toyota, K.; Fukuda, R.; Hasegawa, J.; Ishida, M.; Nakajima, T.; Honda, Y.; Kitao, O.; Nakai, H.; Vreven, T.; Throssell, K.; Montgomery, J. A., Jr.; Peralta, J. E.; Ogliaro, F.; Bearpark, M. J.; Heyd, J. J.; Brothers, E. N.; Kudin, K. N.; Staroverov, V. N.; Keith, T. A.; Kobayashi, R.; Normand, J.; Raghavachari, K.; Rendell, A. P.; Burant, J. C.; Iyengar, S. S.; Tomasi, J.; Cossi, M.; Millam, J. M.; Klene, M.; Adamo, C.; Cammi, R.; Ochterski, J. W.; Martin, R. L.; Morokuma, K.; Farkas, O.; Foresman, J. B.; Fox, D. J. Gaussian, Inc., Wallingford CT, 2016.

S10. Zhao, Y.; Truhlar, D. G. The M06 Suite of Density Functionals for Main Group Thermochemistry, Thermochemical Kinetics, Non-covalent Interactions, Excited States, and Transition Elements: Two New Functionals and Systematic Testing of Four M06-Class Functionals and 12 Other Functionals. *Theor. Chem. Acc.* **2008**, *120*, 215–241.

S11. Scalmani, G.; Frisch, M. J. Continuous Surface Charge Polarizable Continuum Models of Solvation. I. General Formalism. *J. Chem. Phys.* **2010**, *132*, 114110.

S12. Ribeiro, R. F.; Marenich, A. V.; Cramer, C. J. &; Truhlar, D. G. Use of Solution-Phase Vibrational Frequencies in Continuum Models for the Free Energy of Solvation. *J. Phys. Chem. B* **2011**, *115*, 14556–14562.

S13. (a) Hratchian, H. P.; Schlegel, H. B. Accurate reaction paths using a Hessian based predictor-corrector integrator. *J. Chem. Phys.* **2004**, *120*, 9918–9924. (b) Hratchian, H. P.; Schlegel, H. B. Using Hessian updating to increase the efficiency of a Hessian based predictor-corrector reaction path following method. *J. Chem. Theory Comput.* **2005**, *1*, 61–69.

S14. (a) Johnson, E. R; Keinan, S; Mori-Sanchez, P; Contreras-Garcia, J; Cohen, A. J.; Yang, W. *J. Am. Chem. Soc.* **2010**, *132*, 6498–6506. (b) Contreras-Garcia, J.; Johnson, E. R.; Keinan, S.; Chaudret, R.; Piquemal, J-P.; Beratan, D. N.; Yang. W. *J. Chem. Theory Comput.* **2011**, 7, 625–632.
